# Gold(I)-Catalyzed Intermolecular Aryloxyvinylation with Acetylene Gas

**DOI:** 10.1021/acscatal.3c02461

**Published:** 2023-08-01

**Authors:** Tania Medina-Gil, Anna Sadurní, L. Anders Hammarback, Antonio M. Echavarren

**Affiliations:** Institute of Chemical Research of Catalonia (ICIQ), Barcelona Institute of Science and Technology (BIST), Av. Països Catalans 16, 43007 Tarragona, Spain; Departament de Química Orgànica i Analítica, Universitat Rovira i Virgili (URV), C/Marcel·lí Domingo s/n, 43007 Tarragona, Spain

**Keywords:** gold catalysis, acetylene, aryloxycyclization, chromanes, enantioselective catalysis

## Abstract

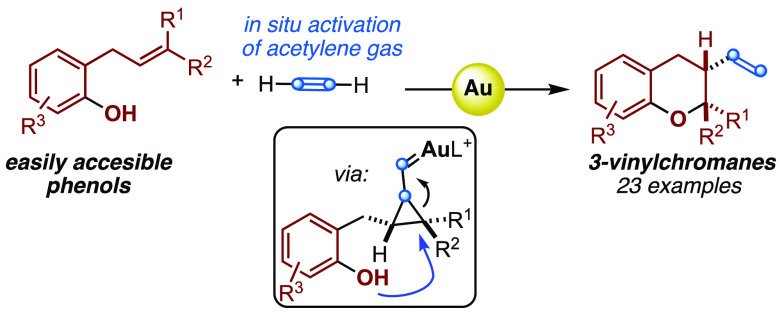

Acetylene gas is an important feedstock for chemical production, although it is underutilized in organic synthesis. We have developed an intermolecular gold(I)-catalyzed alkyne/alkene reaction of *o*-allylphenols with acetylene gas that gives rise to chromanes by a stereospecific aryloxycyclization through the nucleophilic regioselective opening of cyclopropyl gold(I)-carbene intermediates. The synthetic application of this method was demonstrated in the late-stage functionalization of the natural product lapachol.

Acetylene is one of the most important feedstocks in chemical industry due to its ready availability and high reactivity.^1,2^ Acetylene can be produced by several well-established methods such as the reaction of calcium carbide with water^2^ or the partial combustion of hydrocarbons.^3^ The importance of acetylene-based chemistry is best illustrated by its remarkable production market that reached 1.9 million tones in 2020 and is expected to continue growing until 2030.^2^ Despite this, so far, chemical applications of acetylene have been mainly limited to noncatalyzed vinylation reactions^4^ or hydrochlorination processes,^5^ whereas its use in catalytic reactions has been less explored.^2,6−8^

Homogeneous gold(I) complexes are highly efficient catalysts for the electrophilic activation of alkynes.^9^ Although gold(I)-catalyzed cyclizations of 1,n-enynes,^9^ such as the alkoxycyclizations by intramolecular alkyne/alkene reactions (Scheme 1a),^10^ have been widely explored, broad scope intermolecular reactions between alkynes and alkenes are less common.^11−13^ Besides the possible polymerization of the alkenes,^14^ the main hurdle is that products of intermolecular reactions of alkynes with alkenes are also alkenes, which can react further with the alkyne leading to the formation of oligomers. Indeed, we recently reported that acetylene gas reacts with *trans*-stilbene in the presence of gold(I) catalysts to form (*Z*,*Z*)-1,3-dienes, along with oligomers that result from the formal insertion of C_2_ units (Scheme 1b, pathway 1).^15^ By using a NHC-gold(I) catalyst, biscyclopropyl products were also obtained, which was applied to the first total synthesis of the sesquiterpene waitziacuminone in a single step.^15^ (Scheme 1b, pathway 2).

**Scheme 1:**
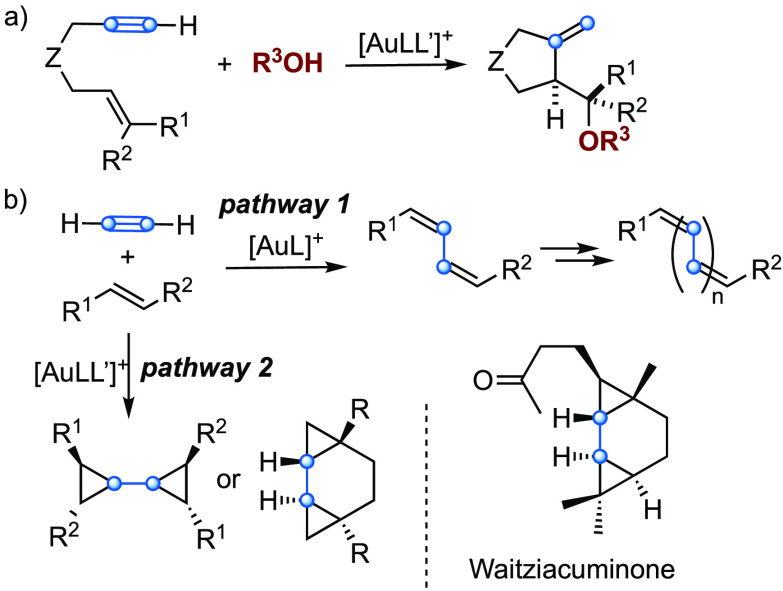
(a) Gold(I)-Catalyzed Alkoxycyclization by Intramolecular Alkyne/Alkene Reaction^9,10^ and (b) Activation of Acetylene Gas^15^

Although alkoxycyclizations of 1,n-enynes are well-known (Scheme 1a),^10,16^ the intermolecular version has not yet been developed. We reasoned that using acetylene gas as an intermolecular in the gold(I)-catalyzed reaction with an alkene would give rise to products with a terminal vinyl group, which are less reactive in subsequent reactions with acetylene, thus minimizing the problem of oligomerization. Here, we report the realization of this concept by developing an intermolecular alkyne/alkene gold(I)-catalyzed reaction from *o*-allylphenols **1** and acetylene gas that gives rise stereospecifically to chromanes **2** (Scheme 2). In this aryloxyvinylation reaction, the initial acetylene gold(I) complex is the electrophile that reacts with the alkene to form cyclopropyl gold(I)-carbene **IntA**, which reacts regioselectively with the phenol at C-3 of the allyl chain to form a 6-membered ring. The resulting chromanes are important heterocyclic scaffolds present in a wide variety of natural products, agrochemical, and pharmaceutical compounds,^17^ such as α-tocopherol,^18^ (+)-catechin,^19^ deguelin,^20^ and cromakalim.^21^

**Scheme 2:**
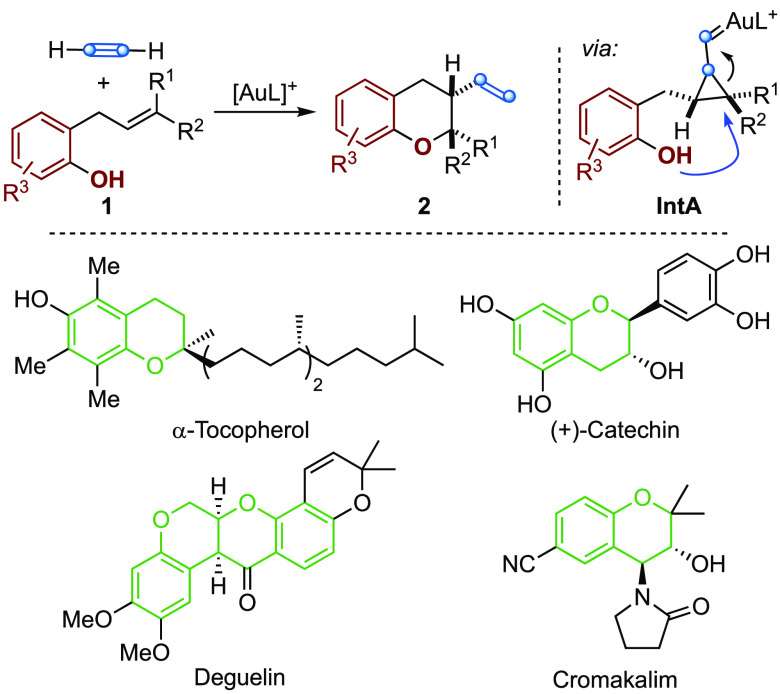
Aryloxyvinylation by Gold(I)-Catalyzed Intermolecular Alkyne/Alkene Reaction

The reaction of 2-cinnamyl phenol (**1a**) with acetylene gas in the presence of commercially available JohnPhosAuCl (**A**) as a catalyst and NaBAr^F^_4_ as a halide scavenger gave the desired vinylated chromane **2a** in 78% yield (Table 1, entry 1). Gold(I) catalysts **B**, **C**, and **D** led to **2a** in lower yields (Table 1, entries 2–4). Using CHCl_3_ instead of CH_2_Cl_2_ as solvent with catalyst **A** improved the yield of **2a** to 89% (Table 1, entry 5). Aromatic solvents gave comparable yields, toluene being the best one, providing **2a** in 81% yield (Table 1, entry 6). Changing NaBAr^F^_4_ to AgSbF_6_ as a chloride abstractor led to a drop of the yield (49%) (Table 1, entry 7). Finally, **2a** was obtained in 91% yield by increasing the concentration (Table 1, entry 9).^22^

**Table 1:**
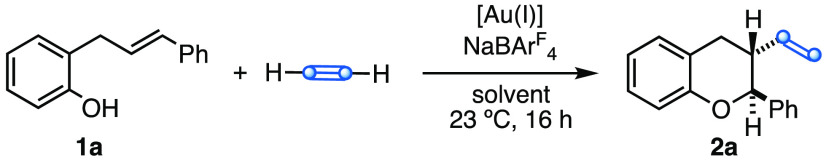
Gold(I)-Catalyzed Reaction of **1a** with Acetylene Gas to Form Chromane **2a**^a^

entry	[Au(I)] (mol %)	solvent	yield (%)^b^
1	**A** (6)	CH_2_Cl_2_	78
2	**B** (6)	CH_2_Cl_2_	71
3	**C** (6)	CH_2_Cl_2_	53
4	**D** (6)	CH_2_Cl_2_	50
5	**A** (6)	CHCl_3_	89
6	**A** (6)	toluene	81
7^c^	**A** (6)	CHCl_3_	49
8	**A** (4)	CHCl_3_	69
9^d^	**A** (6)	CHCl_3_	91

a Reactions were carried out using 0.10 mmol scale of substrate **1a** at 0.2 M concentration.

b Yields determined by ^1^H NMR using 1,1,2,2-tetrachloroethane as internal standard.

c AgSbF_6_ instead of NaBAr^F^_4_.

d 0.4 M concentration.

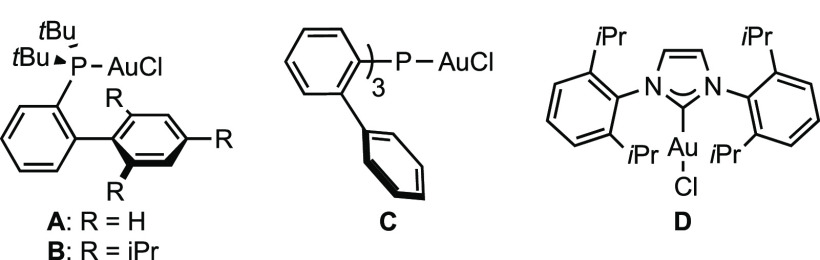

The optimized reaction conditions were applied to the synthesis of a variety of 3-vinyl chromane derivates **2a**–**w** (Scheme 3). First, the influence of the substituents on the phenol ring was investigated. Substrates with electron-donating groups gave the corresponding products **2b**–**d**, **2h**, and **2j**–**l** in moderate to good yields, whereas **2i** could only be isolated in 29% yield. An allyl phenol with a Br substituent in the *para*-position led to chromane **2e** in good yield, whereas substitution with more strongly electron-withdrawing ester or CF_3_ groups led to **2f** and **2g** in 20% and 33% yield, respectively, presumably because of the decreased nucleophilicity of the corresponding phenols. Substrates with different substituents on the phenyl ring of the cinnamyl chain led to products **2m**–**t** in gold yields, except for **2p** and **2r** with a *p*-CF_3_ or *o*-Br, which were isolated in 21% and 30% yields, respectively (Scheme 3). Other *o*-allyl phenols with different substituents at the alkene gave chromanes **2u**–**w** in 37–74% yields.

**Scheme 3:**
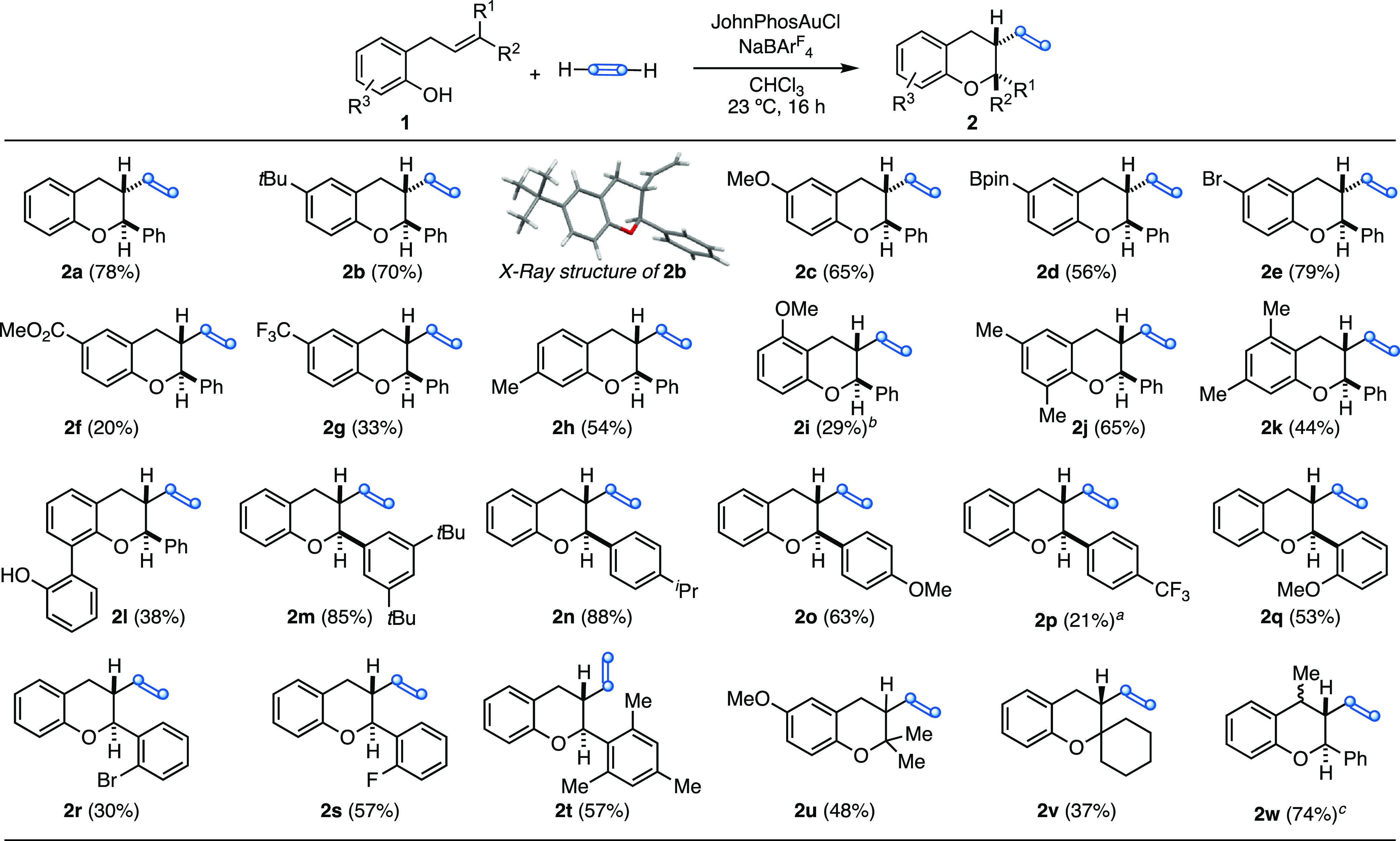
Synthesis of Chromanes **2** by Gold(I)-Catalyzed Aryloxyvinylation of *o*-Allylphenols **1** with Acetylene Gas 48 h reaction time. 0 °C for 3 h. 1:1 mixture of stereoisomers

The observed *anti*-stereochemistry and excusive 6-*endo*-*trig* regioselectivity is identical to that found in similar formation of chromanes by halocyclization of the same substrates.^23^ However, in our case, the cyclization is induced by the addition of acetylene as a C2 equiv of the halonium electrophile.

Since *o*-allylphenols are ubiquitous in nature, this aryloxyvinylation could be used for the late-stage modification of this class of natural products.^24^ As a preliminary demonstration of this concept, we have applied this new reaction to the natural product lapachol (**3**), a derivate of vitamin K,^25^ leading to 3-vinyl-α-lapachone **4** in 50% yield (Scheme 4a). Vinyl chromane **2d** was converted into **2x** by Suzuki cross-coupling with bromobenzene (Scheme 4b). The vinyl group provides a versatile handle for diversification. Thus, **2a** led to **5** by Wacker oxidation, whereas reaction with *m*CPBA gave **6** (Scheme 4c). Furthermore, metathesis of **2a** with methyl acrylate afforded **7**, and the hydroboration with HBpin provided **8**. A monocationic catalyst generated in situ from JosiPhos-type digold(I) complex (*R,S*_*P*_)-**E**^12d^ proved to be highly active leading to **2a**, **2d**, **2e**, and **2m** (Scheme 4d). Although the achieved enantioselectivities are still moderate, these are the first examples of enantioselective activation of acetylene in gold(I) catalysis.

**Scheme 4:**
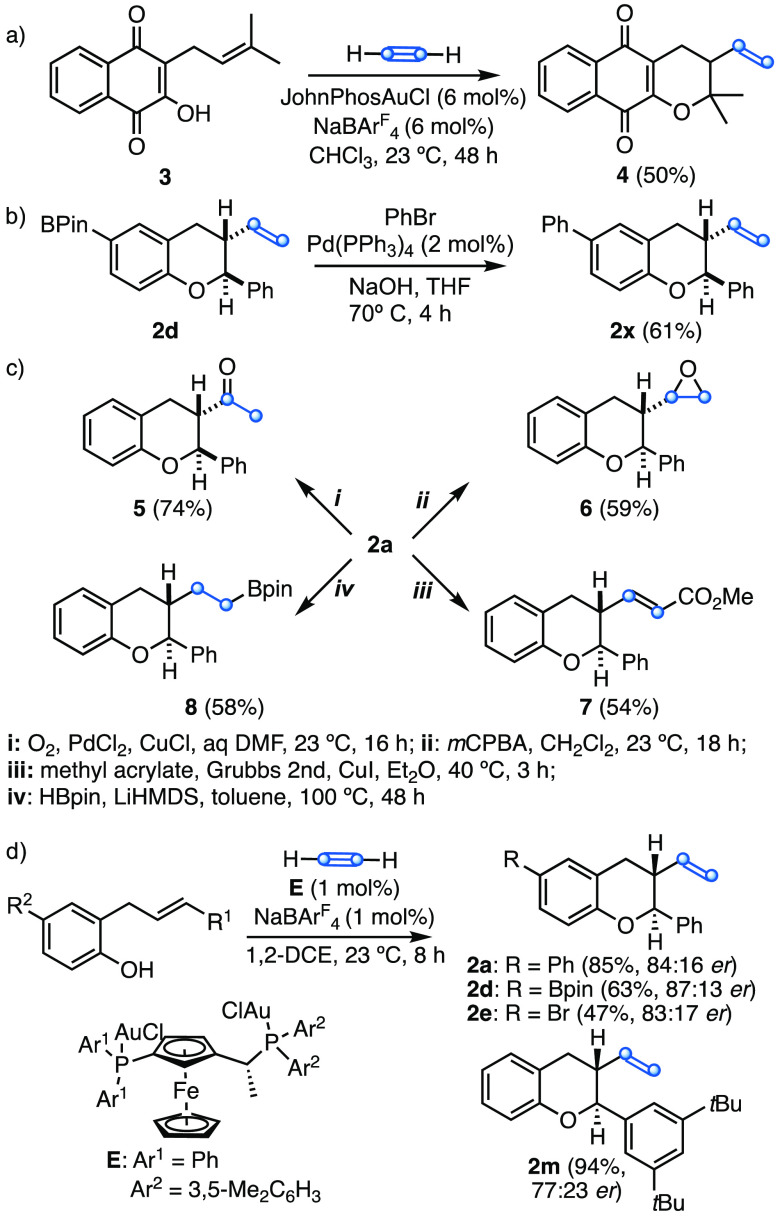
(a) Vinylation of Lapachol (**3**); (b) Derivatization of **2d**; (c) Derivatization of **2a**; (d) Enantioselective Aryloxycyclization

In summary, we have developed a gold(I)-catalyzed intermolecular reaction between acetylene gas and readily available *o*-allylphenols as a novel approach for the synthesis of 3-vinylchromanes. This stereoselective intermolecular aryloxyvinylation leads to chromanes in moderate to excellent yields, showing good functional group tolerance. The applicability of this method was demonstrated by the late*-*stage functionalization of the natural product lapachol (**3**) and with the diversification at the aryl or vinyl of the resulting chromanes. This new methodology combines the use of common feedstock reagents such as acetylene gas and phenols with the employment of gold(I) catalysis to obtain scaffolds widely abundant in natural products and pharmacologically active compounds.

## Supporting Information Available

Experimental procedures, characterization data, NMR data, UPC2 and HPLC traces, computational details, and X-ray data (PDF)
