# Supplementary material for: Gold(I)-Catalyzed Intermolecular Aryloxyvinylation with Acetylene Gas
Source: ACS Catal. 2023 Aug 1;13(16):10751–5. doi: 10.1021/acscatal.3c02461 (PMC10442918; doi:10.1021/acscatal.3c02461)
Supplement: Supplementary file 1 — cs3c02461_si_001.pdf [file cs3c02461_si_001.pdf]

Supporting Information

Gold(I)-Catalyzed Intermolecular Aryloxyvinylation with Acetylene Gas

Tania Medina-Gil, Anna Sadurní, L. Anders Hammarback, and Antonio M. Echavarren*

Institute of Chemical Research of Catalonia (ICIQ) Barcelona Institute of Science and Technology (BIST), Av. Paisos Catalans 16, 43007 Tarragona (Spain)

Department de Química Orgànica i Analítica Universitat Rovira I Virgili (URV), C/ Marcel·lí Domingo s/n, 43007 Tarragona (Spain)

Email: *aechavarren@iciq.es*

Table of Contents

1. General Remarks 3

2. General Procedures 4

2.1 General Procedure GP1: Synthesis of *o*-allylphenols 4

2.2 General Procedure GP2: Synthesis of phenyl substituted *o*-allylphenols 4

2.3 General Procedure GP3: Gold(I)-catalyzed aryloxyvinylation using acetylene gas 5

2.4 General Procedure GP4: Enantioselective gold(I)-catalyzed aryloxyvinylation using acetylene gas 5

2.5 HEL reactor 6

3. Optimization of the reaction conditions 7

4. Procedures and characterization 11

4.1 Synthesis of the Starting Materials (1) 11

4.2 Synthesis of 2-vinyl chromanes (2) 20

4.3 Functionalization of Lapachol (3) 33

4.4 Suzuki coupling of product 2d 34

4. 5 Diversification of product 2a 34

4.6 Synthesis of chiral catalyst (E) 36

5. NMR spectra 38

5.1 Starting materials 38

5.2 Products 51

5.3 Suzuki coupling of compound 2d 77

5.4 Late stage functionalization of natural product lapachol 78

5.5 Diversfication of the product 2a 79

5.6 Chiral Catalyst E 83

6. X-Ray structures 85

6.1 6-(*tert*-Butyl)-2-phenyl-3-vinylchromane (Product 2b) 85

6.2 (2*S*,3*R*)-4,4,5,5-Tetramethyl-2-(2-phenyl-3-vinylchroman-6-yl)-1,3,2-dioxaborolane (Product 2d) 93

7. SFC 99

8. References 105

1. General Remarks

Unless otherwise stated, reactions were performed with magnetic stirring. Compound names were generated using ChemDraw. Chemicals were obtained from commercial suppliers and used as received. Anhydrous solvents were dried by passing through an activated alumina column on a PureSolv™ Solvent Purification System or taken from commercial bottles equipped with septa and molecular sieves. Solutions were evaporated using a Büchi rotary evaporator under reduced pressure at T ≤ 40 °C. Yields refer to chromatographically and spectroscopically pure homogenous material, unless otherwise stated. Analytical thin-layer chromatography (TLC) was carried out using aluminum sheets coated with 0.2 mm of silica gel (fluorescent-treated Merck Kieselgel 60 F254). Visualization was accomplished under UV light at 254 nm and by staining with an alkaline aqueous potassium permanganate solution, ninhydrin, or vanillin staining dips. Flash column chromatography (FCC) was carried out manually using PanReac Silica Gel 60 (40–63 μm) or employing the automated flash column chromatographer CombiFlash Companion with disposable pre-packed normal phase silica gel columns (Teledyne Isco). Preparative TLC was performed on 20 cm × 20 cm silica gel plates (2.0 mm or 1.0 mm silica thickness, Analtech). Reactions with acetylene were performed in a HEL CAT24 multireactor using an acetylene 2.6 cylinder B50 Messer with regulator BT2000. Enantiomeric excesses were determined by SFC analysis using the chiral stationary phase columns, eluents and conditions specified in the individual procedures and by comparing the sample with the appropriate racemic mixture. SFC analyses were performed on an Agilent Technologies 1260 Infinity II or on a Waters ACQUITY UPC2 instrument.

NMR spectra were recorded at 298 K on Bruker Ultrashield NMR spectrometers operating at ^{1}H resonances of 300, 400 or 500 MHz (in the latter case, with optional cryoprobe for enhanced sensitivity). Proton and carbon chemical shifts (δ) are given in parts per million (ppm) downfield from tetramethylsilane, using the solvent resonance as reference.[1] ^{1}H NMR spectra are reported as follows: chemical shift (multiplicity, coupling constant, number of protons). The following abbreviations are used: s = singlet, d = doublet, t = triplet, q = quartet, quint = quintet, m = multiplet, br = broad signal, app = apparent. ^{13}C, ^{31}P and ^{19}F NMR spectra were always acquired with proton decoupling, even when not explicitly written. ^{1}H, ^{13}C{^{1}H}, ^{31}P{^{1}H} and ^{19}F{^{1}H} NMR spectra for all novel compounds and for all methodology products (both known and novel ones) are attached. Two-dimensional NMR spectroscopy experiments (COSY, HSQC and HMBC) were used to assist in the assignment of signals in ^{1}H and ^{13}C spectra and data are not reported. High-resolution mass spectra (HRMS) were recorded by ICIQ mass spectrometry staff on MaXis Impact, MicrOTOF II and AutoFlex spectrometers equipped with ESI, APCI or MALDI sources, all by Bruker Daltonics. Melting points were measured using a Mettler Toledo MP70 Melting Point apparatus. Single-crystal X-ray diffraction (XRD) data were collected and refined by ICIQ XRD staff either on a Rigaku MicroMax-007HF diffractometer, equipped with a Pilatus 200 K area detector, a Rigaku MicroMax-007HF microfocus rotating anode with MoK$_{\alpha}$ radiation, confocal Max Flux optics and an Oxford Cryostream 700 plus, or on a Bruker Apex II DUO diffractometer, equipped with APEX DII 4K CCD area detector, Mo and Cu X-ray sources and an Oxford Cryostream 700 plus.

2. General Procedures

2.1 General Procedure GP1: Synthesis of *o*-allylphenols

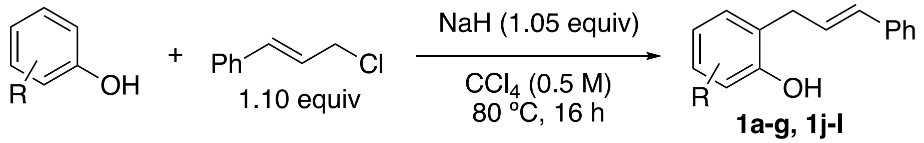

A microwave vial under argon atmosphere was charged with NaH (1.05 equiv, 60% Wt) in dry CCl_4 (0.5 M). To this mixture, the corresponding phenol **1** (1.0 equiv) was slowly added at 0 ºC and the reaction was stirred for 30 min at 0 ºC. Then, (*E*)-cinnamyl chloride (1.10 equiv) was added in one portion and the mixture was heated to 80 ºC and stirred for 16 h. After completion of the reaction, the mixture was cooled down to room temperature and acidified until pH<1 with an aqueous solution of HCl 1M. Then, it was diluted with water (10 mL) and extracted three times with CH_2Cl_2 (3 x 10 mL). The combined organic layers were washed with brine (10 mL), dried over $MgSO_4$, filtered, and concentrated under reduced pressure. The crude product was purified by flash column chromatography (SiO_2, cyclohexane/EtOAc) to afford the o-allylphenols **1.**

2.2 General Procedure GP2: Synthesis of phenyl substituted *o*-allylphenols

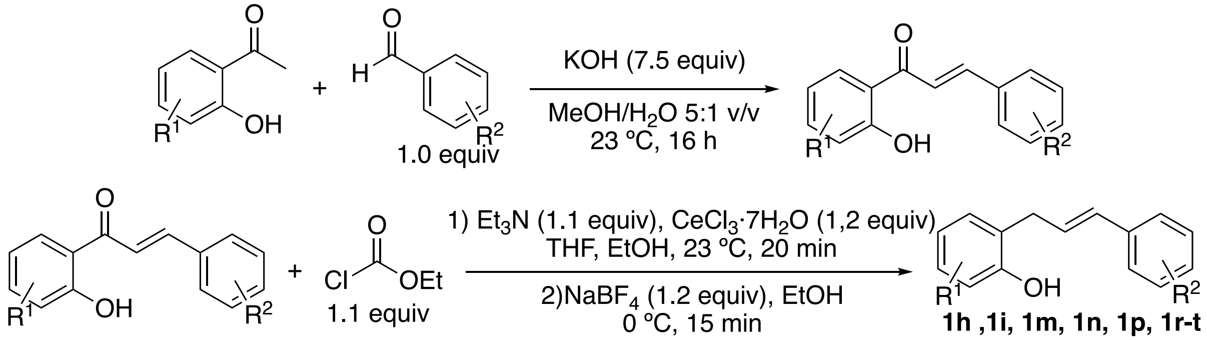

Adapted from a literature procedure.[1,2] To a round-bottomed flask was added the corresponding aldehyde (1.0 equiv) and KOH (7.5 equiv) dissolved in MeOH/H_2O (50%, 5:1, v/v). Following stirring for 10 minutes, the corresponding 2-hydroxyacetophenone (1.0 equiv) was added slowly. The reaction mixture was stirred at room temperature overnight afterwhich the reaction was diluted with water (10 mL) and extracted three times with EtOAc (3 x 15 mL). The organic layer was washed with brine (15 mL), dried over $MgSO_4$, filtered, and concentrated under reduced pressure. The crude material was recrystallised from CH_2Cl_2/MeOH and used without further purification.

To a round bottomed flask was added starting chalcone (1.0 equiv), triethylamine (1.1 equiv) and THF (2 ml $mmol^{-1}$). Ethyl chloroformate (1.1 equiv) in THF (1 ml $mmol^{-1}$) was added dropwise and the resulting mixture was stirred for 30 min. Thereafter, the insoluble amine salt was removed *via* filtration and washed with a further portion of THF (2 ml $mmol^{-1}$). The filtrate was added to a solution of $CeCl_3 \cdot 7H_2O$ (1.2 equiv) in EtOH (5 ml $mmol^{-1}$) and stirred for 20 minutes. $NaBF_4$ (1.2 equiv) in EtOH (5 ml $mmol^{-1}$) was added dropwise at 0 °C and the mixture stirred for 15 minutes before being dilluted with water (10 mL). The pH was adjusted to 3 – 4 using HCl (10% in H_2O) and extracted three times with CH_2Cl_2 (3 x 15 mL). The organic layer was washed with water and brine (15 mL) before being dried over $MgSO_4$ and concentrated *in vacuo*. The crude material was purified by flash column chromatography (SiO_2, cyclohexane/EtOAc) to yield the desired product **1**.

2.3 General Procedure GP3: Gold(I)-catalyzed aryloxyvinylation using acetylene gas

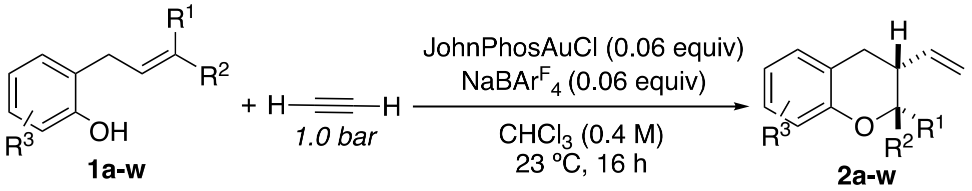

A test tube was charged with phenol **1** (1.0 equiv), JohnPhosAuCl (0.06 equiv) and $NaBAr^F{}_4$ (0.06 equiv) in HPLC grade $CHCl_3$ (0.4 M). The tube was introduced in a HEL reactor, which after proper closure, was pressurized with 1.0 bar of acetylene gas. The reaction mixture was stirred at 23 ºC for 16 h and after emptying the remaining gas, the crude was quenched by the addition of 3 drops of NEt_3 and concentrated under reduced pressure. The crude product was purified as described in the single procedures yielding the corresponding products **2**.

2.4 General Procedure GP4: Enantioselective gold(I)-catalyzed aryloxyvinylation using acetylene gas

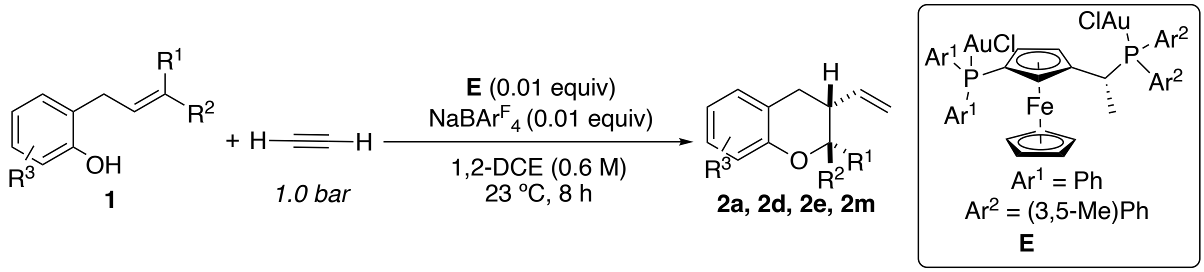

A test tube was charged with phenol **1** (1.0 equiv), chiral catalyst **E** (0.01equiv) and $NaBAr^F{}_4$ (0.01 equiv) in HPLC grade 1,2-DCE (0.6 M). The tube was introduced in a HEL reactor that, after proper closure, was pressurized with 1.0 bar of acetylene gas. The reaction mixture was stirred at 23 ºC for 8 h and after emptying the remaining gas, the crude was quenched by the addition of 3 drops of NEt_3 and concentrated under reduced pressure. The crude product was purified as described in the single procedures yielding the corresponding products **2**.

2.5 HEL reactor

Figure S-1 shows the set up of the reactions using acetylene gas. At lower reaction temperatures, the reactions can also be performed through sparging the reaction solution using a balloon filled with acetylene inside a capped microwave vial.

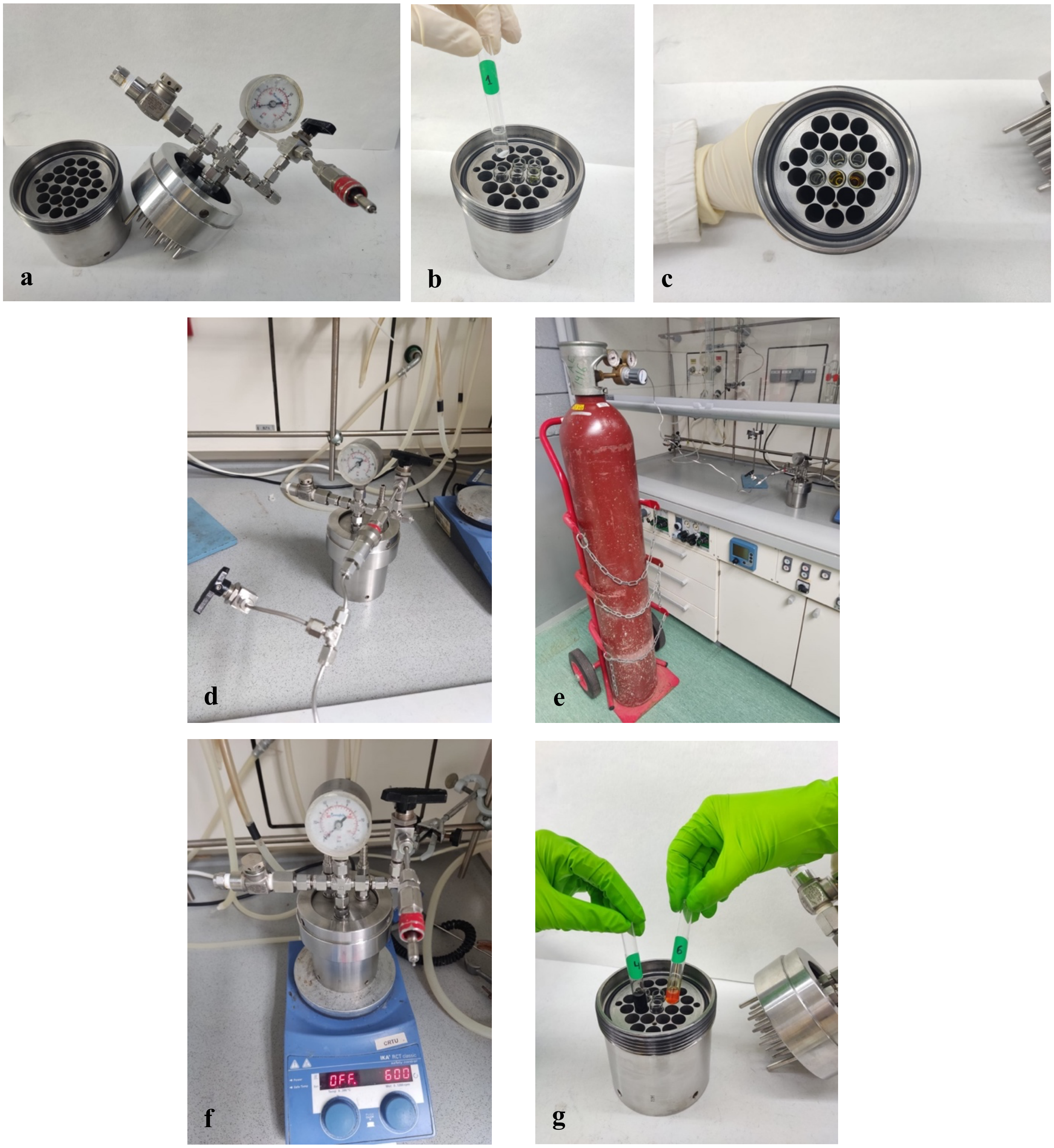

Figure S-1. a) HEL reactor; b) and c) the reactions are placed in the reactor; d) the reactor is closed and connected to the acetylene cylinder; e) the reactor is charged with acetylene (1 bar); f) the cylinder is disconected and the reaction is stirred at 600 rpm for 16 h; g) the acetylene gas is released when the valve of the reactor is opened.

3. Optimization of the reaction conditions

All the reactions from the optimization were carried out in a 0.100 mmol scale of starting phenol **1a** and in a HEL reactor charged with 1 bar of acetylene gas.

Table S1. Catalyst screening.

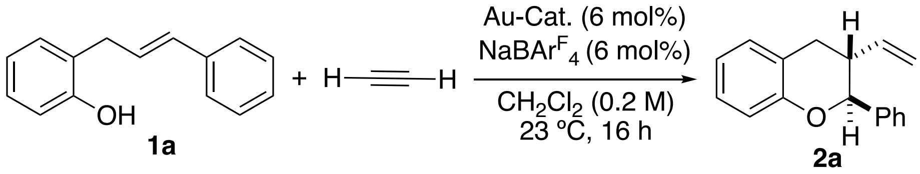

Entry	Catalyst	Yield **2a** (%)[a]
**1**	PPh_3AuCl	n.r
**2**	PMe_3AuCl	n.r
**3**	JohnPhosAuCl	78
**4**	[1, 1'-biphenyl]$_3$AuCl	53
**5**	IPrAuCl	50
**6**	IMesAuCl	n.r
**7**	*t*BuXPhosAuCl	71
**8**[b]	[IPrAuNCPh]BAr^F_4	48
**9**[b]	[*t*BuXPhosAuNCMe]BAr^F_4	75
**10**	none	n.r

[a]Yield determined by ^{1}H NMR spectroscopy using 1,1,2,2-tetrachloroethane as internal standard. [b]Cationic gold(I) complex used without scavenger. n.r no reaction

Table S2. Solvent screening.

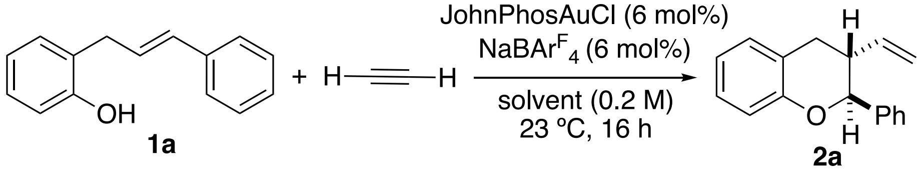

Entry	Solvent	Yield **2a** (%)[a]
**1**	CH_2Cl_2	78
**2**	$CHCl_3$	89
**3**	CCl_4	57
**4**	1,2-Dichloethane	71
**5**	EtOAc	34
**6**	Acetone	12
**7**	THF	8
**8**	Hexane	68
**9**	1,4-Dioxane	44
**10**	Toluene	81
**11**	Mesitylene	26
**12**	Chlorobenzene	80
**13**	α, α, α-Trifluorotoluene	78
**14**	Anisole	56
**15**	Xylene	52
**16**	Nitrobenzene	n.r

[a]Yield determined by ^1H NMR spectroscopy using 1,1,2,2-tetrachloroethane as internal standard.
n.r no reaction

Table S3. Scavenger screening using toluene as solvent.

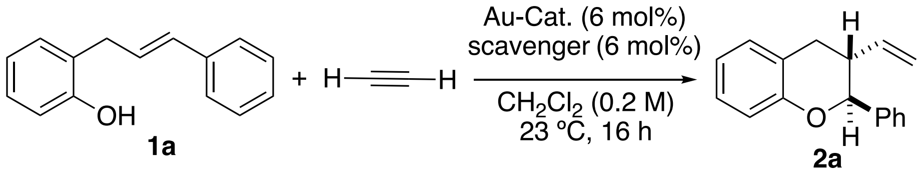

Entry	Scavenger	Yield **2a** (%)[a]
**1**	$NaBAr^F_4$	80
**2**	$AgSbF_6$	40
**3**	$AgNTf_2$	32
**4**	$NaBF_4$	n.r
**5**	none	n.r

[a]Yield determined by ^{1}H NMR spectroscopy using 1,1,2,2-tetrachloroethane as internal standard.
n.r no reaction

Table S4. Catalyst loading and concentration screening using toluene as solvent.

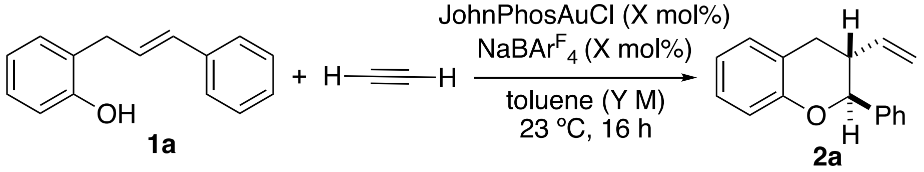

Entry	Catalyst (mol%)	Concentration (M)	Yield **2a** (%)[a]
**1**	6	0.2	80
**2**	4	0.2	69
**3**	2	0.2	47
**4**	6	0.1	60
**5**	6	0.07	n.r
**6**	6	0.4	69

[a]Yield determined by ^{1}H NMR spectroscopy using 1,1,2,2-tetrachloroethane as internal standard.
n.r no reaction

Table S5. Scavenger screening using $CHCl_3$ as solvent.

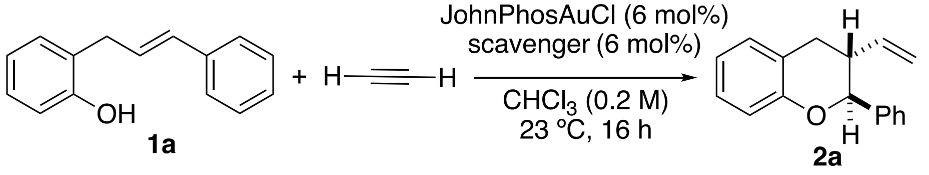

Entry	Scavenger	Yield **2a** (%)[a]
**1**	$NaBAr^F{}_4$	89
**2**	$AgSbF_6$	49

[a]Yield determined by ^{1}H NMR spectroscopy using 1,1,2,2-tetrachloroethane as internal standard.

Table S6. Catalyst loading and concentration screening using $CHCl_3$ as solvent.

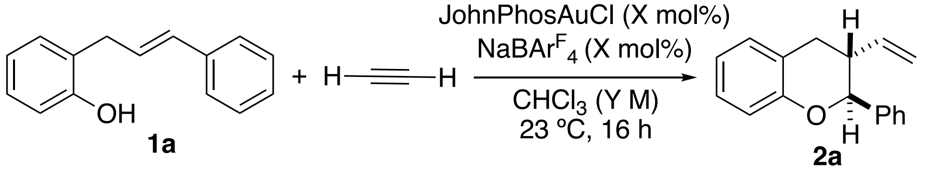

Entry	Catalyst (mol%)	Concentration (M)	Yield **2a** (%)[a]
**1**	8	0.2	75
**2**	6	0.2	89
**3**	4	0.2	69
**4**	6	0.1	80
**5**	6	0.4	91

[a]Yield determined by ^{1}H NMR spectroscopy using 1,1,2,2-tetrachloroethane as internal standard.

4. Procedures and characterization

4.1 Synthesis of the Starting Materials (1)

2-Cynnamylphenol (1a)

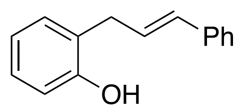

Prepared following the general procedure **GP1** using phenol (3.05 g, 20.0 mmol, 1.0 equiv), NaH (840 mg, 21.0 mmol, 1.05 equiv) and (*E)*-cinnamyl chloride (1.78 mL, 22.0 mmol, 1.10 equiv) in CCl_4 (40.0 mL, 0.5 M) at 80 ºC for 16 h. The crude product was purified by flash column chromatography (SiO_2, cyclohexane/EtOAc 100:0 to 85:15, *v/v*) and the product **1a** was obtained as a yellow solid (3.30 g, 16.0 mmol, 78% yield).

^{1}H NMR (400 MHz, $CDCl_3$) δ 7.39–7.34 (m, 2H), 7.30 (ddd, *J* = 7.8, 6.8, 1.3 Hz, 2H), 7.24–7.11 (m, 3H), 6.92 (td, *J* = 7.4, 1.2 Hz, 1H), 6.83 (dd, *J* = 8.0, 1.2 Hz, 1H), 6.52 (dt, *J* = 15.9, 1.5 Hz, 1H), 6.40 (dt, *J* = 15.9, 6.4 Hz, 1H), 4.94 (s, 1H), 3.58 (dd, *J* = 6.4, 1.4 Hz, 2H). The characterization data matches those reported in the literature.[3]

4-(*tert*-Butyl)-2-cinnamylphenol (1b)

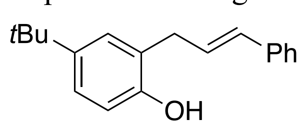

Prepared following the general procedure **GP1** using 4-*tert*-butylphenol (751 mg, 5.00 mmol, 1.0 equiv), NaH (210 mg, 5.25 mmol, 1.05 equiv) and (*E)*-cinnamyl chloride (0.766 mL, 5.50 mmol, 1.10 equiv) in CCl_4 (10.0 mL, 0.5 M) at 80 ºC for 16 h. The crude product was purified by flash column chromatography (SiO_2, cyclohexane/EtOAc 100:0 to 80:20, *v/v*) and the product **1b** was obtained as a white solid (573 mg, 2.15 mmol, 43% yield).

^{1}H NMR (300 MHz, $CDCl_3$) δ 7.41–7.27 (m, 4H), 7.25–7.12 (m, 3H), 6.76 (d, *J* = 9.1 Hz, 1H), 6.51 (d, *J* = 15.9 Hz, 1H), 6.42 (dt, *J* = 15.8, 6.3 Hz, 1H), 4.80 (s, 1H), 3.63–3.50 (m, 2H), 1.30 (s, 9H). The characterization data matches those reported in the literature.[4]

4-Methoxy-2-cinnamylphenol (1c)

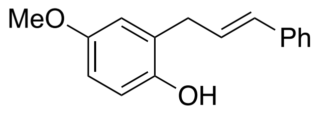

Prepared following the general procedure **GP1** using 4-methoxyphenol (621 mg, 5.00 mmol, 1.0 equiv), NaH (210 mg, 5.25 mmol, 1.05 equiv) and (*E)*-cinnamyl chloride (0.766 mL, 5.50 mmol, 1.10 equiv) in CCl_4 (10.0 mL, 0.5 M) at 80 ºC for 16 h. The crude product was purified by flash column chromatography (SiO_2, cyclohexane/EtOAc 100:0 to 80:20, *v/v*) and the product **1c** was obtained as a white solid (377 mg, 1.57 mmol, 32% yield).

^{1}H NMR (300 MHz, $CDCl_3$) δ 7.39–7.27 (m, 4H), 7.25–7.17 (m, 1H), 6.82–6.73 (m, 2H), 6.73–6.65 (m, 1H), 6.51 (dt, *J* = 15.9, 1.4 Hz, 1H), 6.37 (dt, *J* = 15.9, 6.3 Hz, 1H), 4.63 (s, 1H), 3.77 (s, 3H), 3.54 (dd, *J* = 6.4, 1.3 Hz, 2H). The characterization data matches those reported in the literature.[5]

2-Cinnamyl-4-(4,4,5,5-tetramethyl-1,3,2-dioxaborolan-2-yl)phenol (1d)

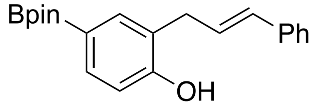

Prepared following the general procedure **GP1** using 4-(4,4,5,5-tetramethyl-1,3,2-dioxaborolan-2-yl)phenol (1.10 g, 5.00 mmol, 1.0 equiv), NaH (210 mg, 5.25 mmol, 1.05 mmol, 1.05 equiv) and (*E)*-cinnamyl chloride (0.766 mL, 5.50 mmol, 1.10 equiv) in CCl_4 (10.0 mL, 0.5 M) at 80 ºC for 16 h. The crude product was purified by flash column chromatography (SiO_2,

cyclohexane/EtOAc 100:0 to 60:40, *v/v*) and the product **1d** was obtained as a white sticky solid (655 mg, 1.95 mmol, 39% yield).

R*f* 0.42 (cyclohexane/EtOAc, 8:2, *v/v*). **^{1}H NMR** (500 MHz, $CDCl_3$) δ 7.68–7.59 (m, 2H), 7.38–7.32 (m, 2H), 7.28 (dd, *J* = 8.5, 6.8 Hz, 2H), 7.24–7.18 (m, 1H), 6.81 (d, *J* = 7.8 Hz, 1H), 6.50 (dt, *J* = 15.8, 1.6 Hz, 1H), 6.42–6.34 (m, 1H), 3.58 (dd, *J* = 6.4, 1.5 Hz, 2H), 1.34 (s, 12H). **^{13}C NMR** (126 MHz, $CDCl_3$) δ 157.1, 137.6, 137.3, 135.3, 131.5, 128.6, 128.1, 127.4, 126.4, 125.1, 115.5, 83.8, 34.4, 27.1, 25.0. **HRMS** (ESI –) calculated for [$C_{21}H_{24}O_3B$] [M–H]$^-$ 335.1824 *m/z*; found 335.1826 *m/z.*

4-Bromo-2-cinnamylphenol (1e)

Prepared following the general procedure **GP1** using methyl 4-bromophenol (865 mg, 5.00 mmol, 1.0 equiv), NaH (210 mg, 5.25 mmol, 1.05 equiv) and (*E)*-cinnamyl chloride (0.766 mL, 5.50 mmol, 1.10 equiv) in CCl_4 (10.0 mL, 0.5 M) at 80 ºC for 16 h. The crude product was purified by flash column chromatography (SiO_2, cyclohexane/EtOAc 100:0 to 80:20, *v/v*) and the product **1e** was obtained as a yellow oil (1.01 g, 3.49 mmol, 70% yield).

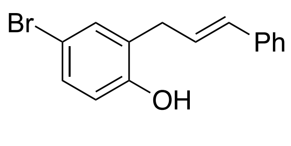

^{1}H NMR (400 MHz, $CDCl_3$) δ 7.39–7.21 (m, 7H), 6.70 (d, *J* = 8.5 Hz, 1H), 6.51 (dt, *J* = 15.8, 1.6 Hz, 1H), 6.34 (dt, *J* = 15.9, 6.7 Hz, 1H), 4.95 (s, 1H), 3.53 (dd, *J* = 6.6, 1.5 Hz, 2H). The characterization data matches those reported in the literature.[6]

Methyl 3-Cinnamyl-4-hydroxybenzoate (1f)

Prepared following the general procedure **GP1** using methyl 4-hydroxybenzoate (122 mg, 0.800 mmol, 1.0 equiv), NaH (33.6 mg, 0.840 mmol, 1.05 equiv) and (*E)*-cinnamyl chloride (0.123 mL, 0.880 mmol, 1.10 equiv) in CCl_4 (1.6 mL, 0.5 M) at 80 ºC for 16 h. The crude product was purified by flash column chromatography (SiO_2, cyclohexane/EtOAc 100:0 to 50:50, *v/v*) and the product **1f** was obtained as a white solid (106 mg, 0.395 mmol, 50% yield).

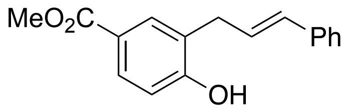

R*f* 0.23 (cyclohexane/EtOAc, 8:2, *v/v*). **M.p.** 122–131 ºC. **^{1}H NMR** (500 MHz, $CDCl_3$) δ 7.90–7.84 (m, 2H), 7.38–7.33 (m, 2H), 7.32–7.28 (m, 2H), 7.24–7.20 (m, 1H), 6.85 (dd, *J* = 8.6, 7.4 Hz, 1H), 6.55–6.49 (m, 1H), 6.37 (dt, *J* = 15.9, 6.6 Hz, 1H), 3.88 (d, *J* = 1.6 Hz, 3H), 3.60 (dd, *J* = 6.6, 1.6 Hz, 2H). **^{13}C NMR** (126 MHz, $CDCl_3$) δ 167.4, 158.6, 137.1, 132.5, 132.1, 130.2, 128.7, 127.5, 127.4, 126.1, 122.8, 115.7, 115.4, 52.1, 34.0. **HRMS** (ESI –) calculated for [$C_{17}H_{15}O_3$] [M–H]$^-$ 267.1027 *m/z*; found 267.1028 *m/z.*

2-Cinnamyl-4-(trifluoromethyl)phenol (1g)

Prepared following the general procedure **GP1** using methyl 4-(trifluroromethyl)phenol (324 mg, 2.00 mmol, 1.0 equiv), NaH (84.0 mg, 2.10 mmol, 1.05 equiv) and (*E*)-cinnamyl chloride (0.306 mL, 2.20 mmol, 1.10 equiv) in CCl_4 (4.0 mL, 0.5 M) at 80 ºC for 16 h. The crude product was purified by flash column chromatography (SiO_2, cyclohexane/EtOAc 100:0 to 50:50, *v/v*) and the product **1g** was obtained as a orange solid (236 mg, 0.848 mmol, 42% yield).

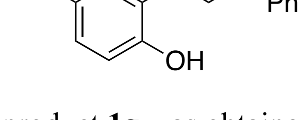

^{1}H NMR (500 MHz, $CDCl_3$) δ 7.47–7.34 (m, 4H), 7.33–7.28 (m, 2H), 7.26–7.20 (m, 1H), 6.88 (d, *J* = 8.5 Hz, 1H), 6.54 (dt, *J* = 15.8, 1.6 Hz, 1H), 6.36 (dt, *J* = 15.9, 6.6 Hz, 1H), 5.36 (s, 1H), 3.60 (dd, *J* = 6.6, 1.6 Hz, 2H). The characterization data matches those reported in the literature.[8]

2-Cinnamyl-5-methylphenol (1h)

Prepared following the general procedure **GP2** using (*E*)-1-(2-hydroxy-4-methylphenyl)-3-phenylpropenone (0.72 g, 3.0 mmol, 1.0 equiv), Et_3N (0.46 mL, 0.33 g, 3.3 mmol, 1.1 equiv), ethyl chloroformate (0.32 mL, 0.36 g, 3.3 mmol, 1.1 equiv), $CeCl_3•7H_2O$ (1.34 g, 3.6 mmol, 1.2 equiv) and $NaBF_4$ (0.14 g, 3.6 mmol, 1.2 equiv.) in THF (15 mL, 0.2 M) and EtOH (30 mL, 0.1 M). The crude product was purified by flash column chromatography (SiO_2, cyclohexane/EtOAc 9:1, *v/v*) and the product **1h** was obtained as a white solid (311 mg, 1.4 mmol, 46% yield).

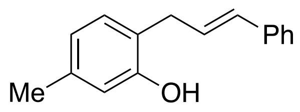

^{1}H NMR (500 MHz, $CDCl_3$) δ 7.37–7.33 (m, 2H), 7.31–7.27 (m, 2H), 7.23–7.19 (m, 1H), 7.05 (d, *J* = 7.6 Hz, 1H), 6.73 (d, *J* = 7.6 Hz, 1H), 6.65 (s, 1H), 6.50 (dt, *J* = 15.8, 1.6 Hz, 1H), 6.38 (dt, *J* = 15.8, 6.5 Hz, 1H), 4.86 (s, 1H), 3.53 (dd, *J* = 6.6, 1.7 Hz, 2H), 2.30 (s, 3H). The characterization data matches those reported in the literature.[7]

2-Cinammyl-3-methoxyphenol (1i)

Prepared following the general procedure **GP2** using (*E*)-1-(2-hydroxy-6-methoxyphenyl)-3-phenylpropenone (1.53 g, 6.00 mmol, 1.0 equiv), Et_3N (0.92 mL, 0.67 g, 6.6 mmol, 1.1 equiv), ethyl chloroformate (0.63 mL, 0.72 g, 6.6 mmol, 1.1 equiv), $CeCl_3•7H_2O$ (2.68 g, 7.20 mmol, 1.2 equiv) and $NaBF_4$ (0.27 g, 7.2 mmol, 1.2 equiv) in THF (30 mL, 0.2 M) and EtOH (60 mL, 0.1 M). The crude product was purified by flash column chromatography (SiO_2, cyclohexane/EtOAc 9:1, *v/v*) and the product **1i** was obtained as a white solid (812 mg, 3.4 mmol, 56% yield).

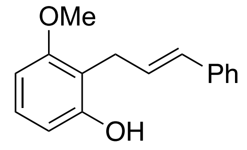

^{1}H NMR (500 MHz, $CDCl_3$) δ 7.35–7.32 (m, 2H), 7.29–7.25 (m, 2H), 7.21–7.16 (m, 1H), 7.10 (dd, *J* = 8.2, 8.2 Hz, 1H), 6.54–6.45 (m, 3H), 6.35 (dt, *J* = 15.9, 6.3, 1H), 4.99 (s, 1H), 3.84 (s, 3H), 3.62 (dd, *J* = 6.4, 1.6 Hz, 2H). The characterization data matches those reported in the literature. [8]

2-Cinnamyl-4,6-dimethylphenol (1j)

Prepared following the general procedure **GP1** using 2,4-dimethylphenol (611 mg, 5.00 mmol, 1.0 equiv), NaH (210 mg, 5.25 mmol, 1.05 equiv) and (*E)*-cinnamyl chloride (0.766 mL, 5.50 mmol, 1.10 equiv) in CCl_4 (10.0 mL, 0.5 M) at 80 ºC for 16 h. The crude product was purified by flash column chromatography (SiO_2, cyclohexane/EtOAc 100:0 to 80:20, *v/v*) and the product **1j** was obtained as a yellow solid (615 mg, 2.58 mmol, 52% yield).

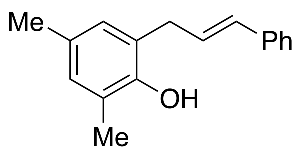

^{1}H NMR (400 MHz, $CDCl_3$) δ 7.44–7.38 (m, 2H), 7.38–7.31 (m, 2H), 7.30–7.23 (m, 1H), 6.95–6.84 (m, 2H), 6.62–6.53 (m, 1H), 6.48–6.36 (m, 1H), 4.85 (s, 1H), 3.58 (dd, *J* = 6.6, 1.5 Hz, 2H), 2.30 (d, *J* = 0.7 Hz, 3H), 2.27 (d, *J* = 0.7 Hz, 3H). The spectroscopic data matches those reported in the literature.[8]

2-Cinnamyl-3,5-dimethylphenol (1k)

Prepared following the general procedure **GP1** using 3,5-dimethylphenol (0.44 g, 3.6 mmol, 1.1 equiv), NaH (0.14 g, 3.4 mmol, 1.05 equiv), cinnamyl chloride (0.46 mL, 3.3 mmol, 1.0 equiv) in CCl_4 (6 mL, 0.5 M). The crude product was purified by flash column chromatography (SiO_2, cyclohexane/EtOAc

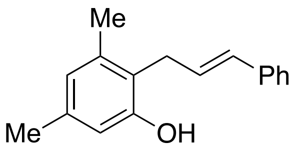

98:2, *v/v*) and the product **1k** was obtained as a white solid (561 mg, 2.40 mmol, 72% yield).

^{1}H NMR (400 MHz, $CDCl_3$) δ 7.33–7.29 (m, 2H), 7.29–7.23 (m, 2H), 7.20–7.15 (m, 1H), 6.63 (s, 1H), 6.52 (s, 1H), 6.41–6.27 (m, 2H), 5.75 (s, 1H), 3.54 (d, *J* = 5.4 Hz, 2H), 2.29 (s, 3H), 2.26 (s, 3H). The characterization data matches those reported in the literature.[9]

3-Cinnamyl-(1,1'-biphenyl)-2,2'-diol (1l)

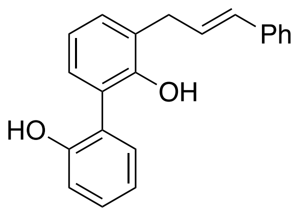

Prepared following the general procedure **GP1** using (1,1'-biphenyl)-2,2'-diol (931 mg, 5.00 mmol, 1.0 equiv), NaH (210 mg, 5.25 mmol, 1.05 equiv) and *(E)*-cinnamyl chloride (0.766 mL, 5.50 mmol, 1.10 equiv) in CCl_4 (10.0 mL, 0.5 M) at 80 ºC for 16 h. The crude product was purified by flash column chromatography (SiO_2, cyclohexane/EtOAc 100:0 to 60:40, *v/v*) and the product **1l** was obtained as a white sticky solid (1.05 g, 3.47 mmol, 70% yield).

R_f 0.37 (cyclohexane/EtOAc, 8:2, *v/v*). **^{1}H NMR** (500 MHz, $CDCl_3$) δ 7.40–7.25 (m, 8H), 7.23–7.14 (m, 2H), 7.08–6.98 (m, 2H), 6.55 (d, *J* = 15.9 Hz, 1H), 6.44 (dt, *J* = 15.8, 6.7 Hz, 1H), 5.52–5.35 (br, 2H), 3.65 (dd, *J* = 6.7, 1.3 Hz, 2H). **^{13}C NMR** (126 MHz, $CDCl_3$) δ 153.3, 151.4, 137.4, 131.7, 131.4, 130.8, 130.2, 129.4, 128.7, 128.2, 127.7, 127.4, 126.3, 123.4, 123.3, 121.7, 121.5, 116.7, 34.2. **HRMS** (ESI –) calculated for [$C_{21}H_{17}O_2$] $[M–H]^–$ 301.1234 *m/z*; found 301.1237 *m/z.*

(*E*)-2-(3-(3,5-Di-*tert*-butylphenyl)allyl)phenol (1m)

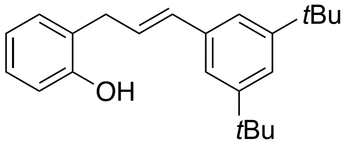

Prepared following the general procedure **GP2** using 1-(2-hydroxyphenyl)-3-(3,5-di-*tert*-butylphenyl)propenone (1.01 g, 3.00 mmol, 1.0 equiv), Et_3N (0.46 mL, 0.33 g, 3.3 mmol, 1.1 equiv), ethyl chloroformate (0.32 mL, 3.3 mmol, 1.1 equiv), $CeCl_3•7H_2O$ (1.34 g, 3.6 mmol, 1.2 equiv) and $NaBF_4$ (0.25 g, 6.6 mmol, 2.2 equiv) in THF (15 mL, 0.2 M) and EtOH (30 mL, 0.1 M). The crude product was purified by flash column chromatography (SiO_2, cyclohexane/EtOAc 95:5, *v/v*) and the product **1m** was obtained as a white solid (264 mg, 0.800 mmol, 27% yield).

R_f 0.31 (cyclohexane/EtOAc, 9:1, *v/v*). **M.p.** 103–106 ºC. **^{1}H NMR** (400 MHz, $CDCl_3$) δ 7.31 (dd, *J* = 7.9, 7.9 Hz, 1H), 7.22–7.14 (m, 4H), 6.92 (ddd, *J* = 7.4, 7.4, 1.2 Hz, 1H), 6.84 (dd, *J* = 8.0, 1.2 Hz, 1H), 6.55 (dt, *J* = 15.9, 1.6 Hz, 1H), 6.38 (dt, *J* = 15.9, 6.6 Hz, 1H), 5.00 (s, 1H), 3.59 (dd, *J* = 6.6, 1.6 Hz, 2H), 1.32 (s, 18H). **^{13}C NMR** (101 MHz, $CDCl_3$) δ 154.4, 151.1, 136.3, 132.8, 130.7, 128.1, 127.1, 125.9, 121.9, 121.1, 120.7, 116.1, 35.0, 34.5, 31.6. **HRMS** (ESI –) calculated for [$C_{23}H_{29}O$] $[M–H]^–$ 321.2224 *m/z*; found 321.2211 *m/z.*

(*E*)-2-(3-(4-*iso*-Propylphenyl)allyl)phenol (1n)

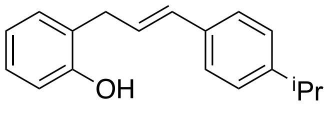

Prepared following the general procedure **GP2** using 1-(2-hydroxyphenyl)-3-(4-*iso*-propylphenyl)propenone (0.80 g, 3.0 mmol, 1.0 equiv), Et_3N (0.46 mL, 0.33 g, 3.3 mmol, 1.1 equiv), ethyl chloroformate (0.32 mL, 3.3 mmol, 1.1 equiv), $CeCl_3•7H_2O$ (1.34 g, 3.6 mmol, 1.2 equiv) and $NaBF_4$ (0.25 g, 6.6 mmol, 2.2 equiv) in THF (15 mL, 0.2 M) and EtOH (30 mL, 0.1 M). The crude product was purified by flash column chromatography (SiO_2, cyclohexane/EtOAc 95:5, *v/v*) and the product **1n** was obtained as a white solid (259 mg, 1.0 mmol, 34% yield).

Rf 0.25 (cyclohexane/EtOAc, 9:1, *v/v*). **M.p.** 48–50 ºC. **^{1}H NMR** (500 MHz, $CDCl_3$) δ 7.30 (d, *J* = 8.1 Hz, 2H), 7.20–7.13 (m, 4H), 6.93–6.89 (m, 1H), 6.83 (d, *J* = 7.9 Hz, 1H), 6.52 (d, *J* = 15.8 Hz, 1H), 6.35 (dt, *J* = 15.8, 6.6 Hz, 1H), 5.00–4.98 (m, 1H), 3.58 (d, *J* = 6.6 Hz, 2H), 2.89 (p, *J* = 6.9 Hz, 1H), 1.25 (dd, *J* = 6.9, 1.0 Hz, 6H). **^{13}C NMR** (126 MHz, $CDCl_3$) δ 154.3, 148.4, 134.8, 131.6, 130.6, 128.0, 127.0, 126.7, 126.3, 125.9, 121.1, 116.0, 34.3, 34.0, 24.1. **HRMS** (ESI –) calculated for [$C_{18}H_{19}O$] [M–H]$^-$ 251.1441 *m/z*; found 251.1443 *m/z.*

Synthesis of 2-(3-(4-methoxyphenyl)allyl)phenol (1o)

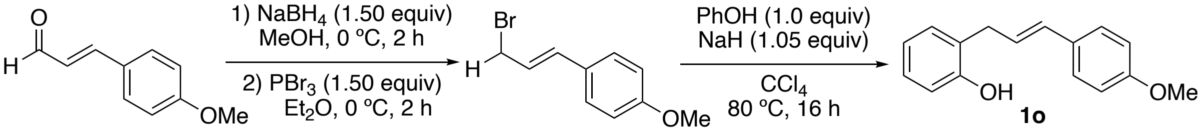

$NaBH_4$ (284 mg, 7.50 mmol, 1.5 equiv) was slowly added to a solution of 3-(4-methoxyphenyl-2-propenal (811 mg, 5.00 mmol, 1.0 equiv) in MeOH (13 mL, 0.4 M) at 0 º C and the solution was stirred for 2 h. The crude mixture was quenched by the addition of a saturated NH_4Cl aqueous solution (15 mL) and extracted three times with EtOAc (3 x 15 mL). The combined organic layers were washed with brine (15 mL), dried over $MgSO_4$, filtered and concentrated under reduced pressure. Then, the crude alcohol was dissolved in dry Et_2O (50 mL, 0.1 M) at 0 ºC and PBr_3 (1.29 mL, 7.50 mmol, 1.50 equiv) was slowly added to the mixture. The reaction was stirred for 2 h at 0 ºC and quenched by the addition of a saturated NH_4Cl aqueous solution (50 mL) and extracted three times with CH_2Cl_2 (3 x 50 mL). The combined organic layers were washed with brine (50 mL), dried over $MgSO_4$, filtered, and concentrated under reduced pressure. The crude bromide was directly used without further purification.

In a 250 mL round-bottom flash under argon atmosphere phenol (527 mg, 5.60 mmol, 1.0 equiv) was dissolved in dry CCl_4 (12.3 mL, 0.5 M) and, to this mixture, NaH (259 mg, 60% Wt, 6.47 mmol, 1.05 equiv) was added in one portion at 0 ºC. The reaction was stirred at 0 ºC for 30 min and the previously synthetized crude bromide (1.5 equiv) was added to the mixture, which was further stirred at 80 ºC for 16 h. After completion of the reaction, the mixture was cooled down to room temperature and acidified until pH<1 with an aqueous solution of HCl 1M. Then, it was diluted with water (20 mL) and extracted three times with CH_2Cl_2 (3 x 20 mL). The combined organic layers were washed with brine (20 mL), dried over $MgSO_4$, filtered and concentrated under reduced pressure. The crude product was purified by flash column chromatography (SiO_2, cyclohexane/EtOAc 100:0 to 60:40, *v/v*) to afford the product **1o** as a yellow oil (361 mg, 1.50 mmol, 24% yield over 3 steps).

Rf 0.53 (cyclohexane/EtOAc, 8:2, *v/v*). **M.p.** 87–93 ºC. **^{1}H NMR** (400 MHz, $CDCl_3$) δ 7.32–7.27 (m, 2H), 7.19–7.12 (m, 2H), 6.91 (td, *J* = 7.5, 1.2 Hz, 1H), 6.87–6.80 (m, 3H), 6.47 (dt, *J* = 15.8, 1.6 Hz, 1H), 6.24 (dt, *J* = 15.8, 6.6 Hz, 1H), 4.97 (s, 1H), 3.80 (s, 3H), 3.55 (dd, *J* = 6.6, 1.6 Hz, 2H). **^{13}C NMR** (101 MHz, $CDCl_3$) δ 159.2, 154.3, 131.2, 130.6, 130.0, 128.0, 127.5, 125.9, 125.7, 121.1, 116.0, 114.1, 55.4, 34.4. **HRMS** (ESI –) calculated for [$C_{16}H_{15}O_2$] [M–H]$^-$ 239.1078 *m/z*; found 239.1079 *m/z.*

2-(3-(4-(Trifluoromethyl)phenyl)allyl)phenol (1p)

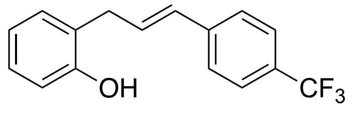

Prepared following the general procedure **GP2** using (*E*)-1-(2-hydroxyphenyl)-3-(4-(trifluoromethyl)phenyl)prop-2-en-1-one (0.56 g, 5.0 mmol, 1.0 equiv), Et_3N (0.77 mL, 0.56 g, 5.5 mmol, 1.1 equiv), ethyl chloroformate (0.53 mL, 0.60 g, 5.5 mmol, 1.1 equiv), $CeCl_3$•$7H_2O$

(2.24 g, 6.00 mmol, 1.2 equiv) and $NaBF_4$ (0.23 g, 6.0 mmol, 1.2 equiv) in THF (15 mL) and EtOH (30 ml). The crude product was purified by flash column chromatography (SiO_2, cyclohexane/EtOAc 9:1, *v/v*) and the product **1p** was obtained as a white solid (644 mg, 2.30 mmol, 46% yield).

^{1}H NMR (500 MHz, $CDCl_3$) δ 7.54 (d, *J* = 8.2 Hz, 2H), 7.44 (d, *J* = 8.1 Hz, 2H), 7.19–7.13 (m, 2H), 6.92 (ddd, *J* = 7.5, 7.5, 1.3 Hz, 1H), 6.81 (dd, *J* = 8.0, 1.2 Hz, 1H), 6.54–6.46 (m, 2H), 4.83 (s, 1H), 3.59 (d, *J* = 4.8 Hz, 2H). The characterization data matches those reported in the literature.[2]

Synthesis of 2-(3-(2-Methoxyphenyl)allyl)phenol (1q)

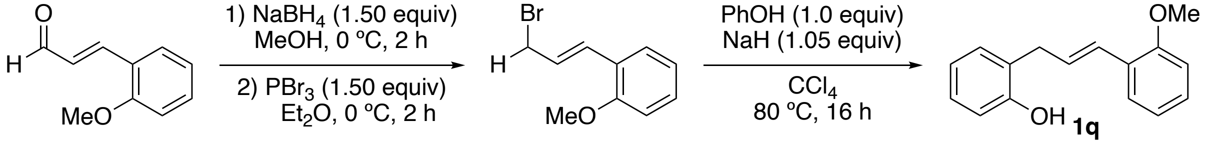

$NaBH_4$ (426 mg, 11.3 mmol, 1.5 equiv) was slowly added to a solution of (*E*)-3-(2-methoxyphenyl)acrylaldehyde (1.10 g, 7.50 mmol, 1.0 equiv) in MeOH (20 mL, 0.4 M) at 0 ºC and the solution was stirred for 2 h. The crude mixture was quenched by the addition of a saturated NH_4Cl aqueous solution (20 mL) and extracted three times with EtOAc (3 x 20 mL). The combined organic layers were washed with brine (30 mL), dried over $MgSO_4$, filtered, and concentrated under reduced pressure. Then, the crude alcohol was dissolved in dry Et_2O (75 mL, 0.1 M) at 0 ºC and PBr_3 (1.13 mL, 11.3 mmol, 1.50 equiv) was slowly added to the mixture. The reaction was stirred for 2 h at 0 ºC and quenched by the addition of a saturated NH_4Cl aqueous solution (50 mL) and extracted three times with CH_2Cl_2 (3 x 50 mL). The combined organic layers were washed with brine (50 mL), dried over $MgSO_4$, filtered, and concentrated under reduced pressure. The crude bromide was directly used without further purification.

In a 250 mL round-bottom flash under argon atmosphere phenol (527 mg, 5.60 mmol, 1.0 equiv) was dissolved in dry CCl_4 (14 mL, 0.5 M) and, to this mixture, NaH (298 mg, 60% Wt, 7.46 mmol, 1.05 equiv) was added in one portion at 0 ºC. The reaction was stirred at 0 ºC for 30 min and the previously synthetized crude bromide (1.50 equiv) was added to the mixture, which was further stirred at 80 ºC for 16 h. After completion of the reaction, the mixture was cooled down to room temperature and acidified until pH<1 with an aqueous solution of HCl 1M. Then, it was diluted with water (20 mL) and extracted three times with CH_2Cl_2 (3 x 20 mL). The combined organic layers were washed with brine (20 mL), dried over $MgSO_4$, filtered and concentrated under reduced pressure. The crude product was purified by flash column chromatography (SiO_2, cyclohexane/EtOAc 100:0 to 60:40, *v/v*) to afford the product **1q** as a yellow oil (335 mg, 1.49 mmol, 21% yield over 3 steps).

R_f 0.59 (cyclohexane/EtOAc, 8:2, *v/v*). **^{1}H NMR** (400 MHz, $CDCl_3$) δ 7.41 (dd, *J* = 7.6, 1.7 Hz, 1H), 7.25–7.12 (m, 3H), 6.95–6.81 (m, 5H), 6.38 (dt, *J* = 16.0, 6.9 Hz, 1H), 5.10 (s, 1H), 3.85 (s, 3H), 3.60 (dd, *J* = 6.9, 1.6 Hz, 2H). **^{13}C NMR** (101 MHz, $CDCl_3$) δ 156.6, 154.4, 130.5, 128.8, 128.6, 128.0, 126.9, 126.8, 126.2, 126.0, 121.1, 120.8, 116.0, 111.0, 55.6, 35.1. **HRMS** (ESI –) calculated for $[C_{16}H_{15}O_2]$ $[M–H]^-$ 239.1078 *m/z*; found 239.1080 *m/z.*

(*E*)-2-(3-(2-Bromophenyl)allyl)phenol (1r)

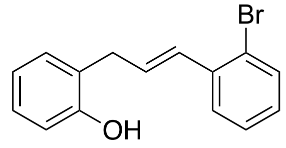

Prepared following the general procedure **GP2** using 1-(2-hydroxyphenyl)-3-(2-bromophenyl)propenone (0.91 g, 3.0 mmol, 1.0 equiv), Et_3N (0.46 mL, 0.33 g, 3.3 mmol, 1.1 equiv), ethyl chloroformate (0.32 mL, 3.3 mmol, 1.1 equiv), $CeCl_3•7H_2O$ (1.34 g, 3.60 mmol, 1.2 equiv) and $NaBF_4$ (0.25 g, 6.6 mmol, 2.2 equiv) in THF (15 mL) and EtOH (30 ml). The crude product was purified by flash column chromatography (SiO_2, cyclohexane/EtOAc 95:5, *v/v*) and the product **1r** was obtained as a white solid (400 mg, 1.40 mmol, 46% yield).

R_f 0.20 (cyclohexane/EtOAc, 9:1, *v/v*). **M.p.** 77–78 ºC. **^{1}H NMR** (500 MHz, $CDCl_3$) δ 7.53 (dd, *J* = 8.0, 1.3 Hz, 1H), 7.49 (dd, *J* = 7.8, 1.7 Hz, 1H), 7.25–7.21 (m, 1H), 7.20–7.13 (m, 2H), 7.07 (ddd, *J* = 7.7, 7.7, 1.7 Hz, 1H), 6.91 (ddd, *J* = 7.5, 7.5, 1.2 Hz, 1H), 6.86 (dt, *J* = 15.7, 1.8 Hz, 1H), 6.82 (dd, *J* = 8.0, 1.2 Hz, 1H), 6.31 (dt, *J* = 15.7, 6.8 Hz, 1H), 4.89 (s, 1H), 3.61 (dd, *J* = 6.8, 1.6 Hz, 2H). **^{13}C NMR** (126 MHz, $CDCl_3$) δ 154.1, 137.3, 133.0, 131.4, 130.6, 130.6, 128.7, 128.1, 127.6, 127.2, 125.7, 123.5, 121.2, 115.9, 34.4. **HRMS** (ESI –) calculated for [$C_{15}H_{13}BrO$] $[M–H]^-$ 287.0077 *m/z*; found 287.0067 *m/z.*

(*E*)-2-(3-(2-Fluorophenyl)allyl)phenol (1s)

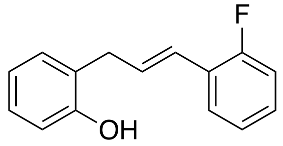

Prepared following the general procedure **GP2** using 1-(2-hydroxyphenyl)-3-(2-fluorophenyl)propenone (0.73 g, 3.0 mmol, 1.0 equiv), Et_3N (0.46 mL, 0.33 g, 3.3 mmol, 1.1 equiv), ethyl chloroformate (0.32 mL, 3.3 mmol, 1.1 equiv), $CeCl_3•7H_2O$ (1.34 g, 3.60 mmol, 1.2 equiv) and $NaBF_4$ (0.25 g, 6.6 mmol, 2.2 equiv) in THF (15 mL) and EtOH (30 ml). The crude product was purified by flash column chromatography (SiO_2, cyclohexane/EtOAc 95:5, *v/v*) and the product **1s** was obtained as a white solid (205 mg, 0.90 mmol, 30% yield).

R_f 0.20 (cyclohexane/EtOAc, 9:1, *v/v*). **M.p.** 43–46 ºC. **^{1}H NMR** (500 MHz, $CDCl_3$) δ 7.43 (ddd, *J* = 7.7, 7.7, 1.8 Hz, 1H), 7.21–7.12 (m, 3H), 7.09–6.99 (m, 2H), 6.91 (ddd, *J* = 7.5, 7.5, 1.2 Hz, 1H), 6.81 (dd, *J* = 8.0, 1.2 Hz, 1H), 6.67 (dt, *J* = 16.0, 1.7 Hz, 1H), 6.47 (dt, *J* = 16.0, 6.7 Hz, 1H), 4.88 (s, 1H), 3.60 (dd, *J* = 6.8, 1.6 Hz, 2H). **^{13}C NMR** (126 MHz, $CDCl_3$) δ 160.2 (d, $J_{C–F}$ = 249.0 Hz), 154.0, 130.9 (d, $J_{C–F}$ = 4.9 Hz), 130.6, 128.6 (d, $J_{C–F}$ = 8.4 Hz), 128.1, 127.4 (d, $J_{C–F}$ = 3.8 Hz), 125.7, 125.1 (d, $J_{C–F}$ = 12.3 Hz), 124.2 (d, $J_{C–F}$ = 3.6 Hz), 124.0 (d, $J_{C–F}$ = 3.5 Hz), 121.2, 115.8, 115.8 (d, $J_{C–F}$ = 22.2 Hz), 34.4. **^{19}F NMR** (471 MHz, $CDCl_3$) δ –118.40. **HRMS** (ESI –) calculated for [$C_{15}H_{13}FO$] $[M–H]^-$ 227.0878 *m/z*; found 227.0872 *m/z.*

(*E*)-2-(3-Mesitylallyl)phenol (1t)

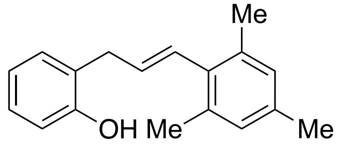

Prepared following the general procedure **GP2** using 1-(2-hydroxyphenyl)-3-mesitylpropenone (0.80 g, 3.0 mmol, 1.0 equiv), Et_3N (0.46 mL, 0.33 g, 3.3 mmol, 1.1 equiv), ethyl chloroformate (0.32 mL, 3.3 mmol, 1.1 equiv), $CeCl_3•7H_2O$ (1.34 g, 3.60 mmol, 1.2 equiv) and $NaBF_4$ (0.25 g, 6.6 mmol, 2.2 equiv) in THF (15 mL) and EtOH (30 ml). The crude product was purified by flash column chromatography (SiO_2, cyclohexane/EtOAc 95:5, *v/v*) and the product **1t** was obtained as a white solid (300 mg, 1.20 mmol, 40% yield).

***R**f* 0.27 (cyclohexane/EtOAc, 9:1, *v/v*). **M.p.** 65–66 ºC. **^{1}H NMR** (400 MHz, $CDCl_3$) δ 7.22–7.12 (m, 2H), 6.91 (ddd, *J* = 7.4, 7.4, 1.2 Hz, 1H), 7.87–7.81 (m, 3H), 6.48 (d, *J* = 16.1, Hz, 1H), 5.85 (dt, *J* = 16.2, 6.6 Hz, 1H), 5.02 (s, 1H), 3.60 (dd, *J* = 6.6, 1.7 Hz, 2H), 2.26 (s, 9H). **^{13}C NMR** (101 MHz, $CDCl_3$) δ 154.3, 136.2, 136.0, 134.0, 132.5, 130.5, 129.6, 128.7, 128.0, 125.9, 121.1, 115.8, 34.8, 21.0. **HRMS** (ESI –) calculated for $[C_{18}H_{19}O]$ $[M–H]^-$ 251.1441 *m/z*; found 251.1433 *m/z.*

Synthesis of 4-methoxy-2-(3-methylbut-2-en-1-yl)phenol (1u)

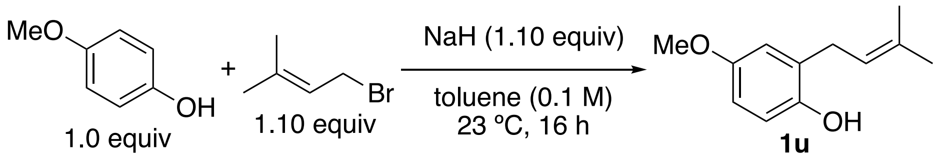

In a 100 mL round-bottom flash under argon atmosphere 4-methoxyphenol (621 mg, 5.00 mmol, 1.0 equiv) was dissolved in dry toluene (50 mL, 0.1 M) and, to this mixture, NaH (220 mg, 60% Wt, 5.50 mmol, 1.10 equiv) was added in one portion at 0 ºC. The reaction was stirred at 0 ºC for 30 min and prenyl bromide (0.639 mL, 5.50 mmol, 1.10 equiv) was added to the mixture which was further stirred at 23 ºC for 16 h. After completion of the reaction it was quenched with water (30 mL) and extracted three times with Et_2O (3 x 30 mL). The combined organic layers were washed with brine (30 mL), dried over $MgSO_4$, filtered and concentrated under reduced pressure. The crude product was purified by flash column chromatography (SiO_2, cyclohexane/EtOAc 100:0 to 60:40, *v/v*) to afford the product **1u** as a white solid (498 mg, 2.59 mmol, 52% yield).

***R**f* 0.44 (cyclohexane/EtOAc, 8:2, *v/v*). **M.p.** 61–63 ºC. **^{1}H NMR** (400 MHz, $CDCl_3$) δ 6.77–6.61 (m, 3H), 5.35–5.28 (m, 1H), 4.94 (s, 1H), 3.76 (s, 3H), 3.33 (d, *J* = 7.2 Hz, 2H), 1.87–1.66 (m, 6H). The spectroscopic data matches those reported in the literature.[10]

Synthesis of 2-(2-cyclohexylideneethyl)phenol (1v)

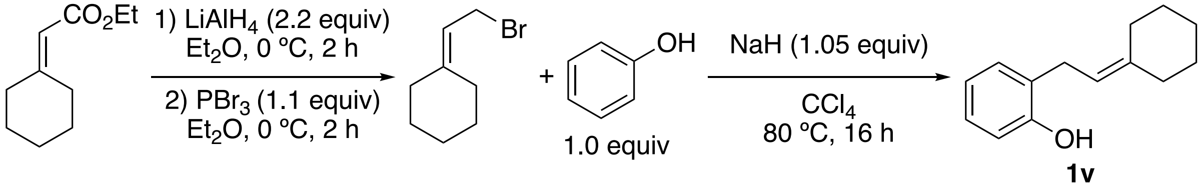

$LiAlH_4$ (32.7 mL, 1.0 M, 32.7 mmol, 2.2 equiv) was slowly added to a solution of 2-cyclohexylidenenacetate (2.50 g, 14.9 mmol, 1.0 equiv) in Et_2O (149 mL, 0.1 M) at 0 ºC and the solution was stirred for 2 h. The crude mixture was quenched by the addition of a saturated NH_4Cl aqueous solution (80 mL) and extracted three times with EtOAc (3 x 80 mL). The combined organic layers were washed with brine (100 mL), dried over $MgSO_4$, filtered and concentrated under reduced pressure. Then, the crude alcohol (expected 13.5 mmol) was dissolved in dry Et_2O (130 mL, 0.1 M) at 0 ºC and PBr_3 (1.48 mL, 14.8 mmol, 1.1 equiv) was slowly added to the mixture. The reaction was stirred for 2 h at 0 ºC and quenched by the addition of a saturated NH_4Cl aqueous solution (50 mL) and extracted three times with CH_2Cl_2 (3 x 50 mL). The combined organic layers were washed with brine (50 mL), dried over $MgSO_4$, filtered, and concentrated under reduced pressure. The crude bromide was directly used without further purification.

In a 250 mL round-bottom flash under argon atmosphere phenol (1.27 g, 13.5 mmol, 1.0 equiv) was dissolved in dry CCl_4 (25 mL, 0.5 M) and, to this mixture, NaH (566 mg, 60% Wt, 14.1 mmol, 1.05 equiv) was added in one portion at 0 ºC. The reaction was stirred at 0 ºC for 30 min and the

previously synthetized crude bromide (1.1 equiv) was added to the mixture which was further stirred at 80 ºC for 16 h. After completion of the reaction the mixture was cooled down to room temperature and acidified until pH<1 with an aqueous solution of 1M HCl. Then, it was diluted with water (20 mL) and extracted three times with CH_2Cl_2 (3 x 20 mL). The combined organic layers were washed with brine (20 mL), dried over $MgSO_4$, filtered, and concentrated under reduced pressure. The crude product was purified by flash column chromatography (SiO_2, cyclohexane/EtOAc 100:0 to 60:40, *v/v*) to afford the product **1v** as an orange oil (427 mg, 13.5 mmol, 16% yield over 3 steps).

***R**$_f$* 0.56 (cyclohexane/EtOAc, 8:2, *v/v*). **^{1}H NMR** (500 MHz, $CDCl_3$) δ 7.17–7.10 (m, 2H), 6.89 (td, *J* = 7.4, 1.2 Hz, 1H), 6.82 (dt, *J* = 8.5, 1.1 Hz, 1H), 5.30 (tdd, *J* = 7.3, 2.4, 1.3 Hz, 1H), 3.40 (d, *J* = 7.4 Hz, 2H), 2.17 (t, *J* = 5.3 Hz, 2H), 2.33 (t, *J* = 5.3 Hz, 2H), 1.60 (hept, *J* = 4.9 Hz, 6H). **^{13}C NMR** (126 MHz, $CDCl_3$) δ 154.5, 143.0, 130.1, 127.6, 127.0, 120.9, 118.5, 115.9, 37.3, 29.1, 28.8, 28.7, 27.8, 26.9. **HRMS** (ESI –) calculated for [$C_{14}H_{17}O$] $[M–H]^-$ 201.1285 *m/z*; found 201.1285 *m/z.*

Synthesis of 2-(4-phenylbut-3-en-2-yl)phenol (1w)

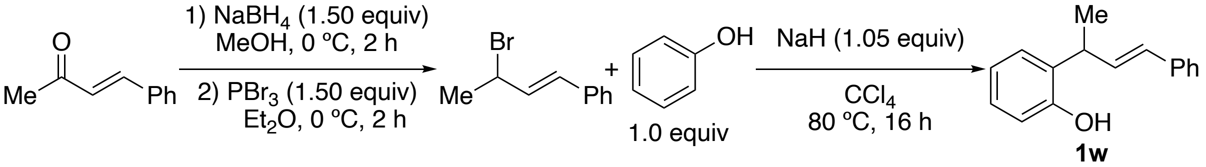

$NaBH_4$ (426 mg, 11.3 mmol, 1.5 equiv) was slowly added to a solution of 4-phenyl-3-buten-2-one (1.10 g, 7.50 mmol, 1.0 equiv) in MeOH (20 mL, 0.4 M) at 0 ºC and the solution was stirred for 2 h. The crude mixture was quenched by the addition of a saturated NH_4Cl aqueous solution (20 mL) and extracted three times with EtOAc (3 x 20 mL). The combined organic layers were washed with brine (30 mL), dried over $MgSO_4$, filtered, and concentrated under reduced pressure. Then, the crude alcohol was dissolved in dry Et_2O (75 mL, 0.1 M) at 0 ºC and PBr_3 (1.13 mL, 11.3 mmol, 1.50 equiv) was slowly added to the mixture. The reaction was stirred for 2 h at 0 ºC and quenched by the addition of a saturated NH_4Cl aqueous solution (50 mL) and extracted three times with CH_2Cl_2 (3 x 50 mL). The combined organic layers were washed with brine (50 mL), dried over $MgSO_4$, filtered, and concentrated under reduced pressure. The crude bromide was directly used without further purification.

In a 250 mL round-bottom flash under argon atmosphere phenol (526 mg, 5.60 mmol, 1.0 equiv) was dissolved in dry CCl_4 (14 mL) and, to this mixture, NaH (298 mg, 60% Wt, 7.46 mmol, 1.05 equiv) was added in one portion at 0 ºC. The reaction was stirred at 0 ºC for 30 min and the previously synthetized crude bromide (1.50 equiv) was added to the mixture, which was further stirred at 80 ºC for 16 h. After completion of the reaction the mixture was cooled down to room temperature and acidified until pH<1 with an aqueous solution of HCl 1M. Then, it was diluted with water (20 mL) and extracted three times with CH_2Cl_2 (3 x 20 mL). The combined organic layers were washed with brine (20 mL), dried over $MgSO_4$, filtered and concentrated under reduced pressure. The crude product was purified by flash column chromatography (SiO_2, cyclohexane/EtOAc 100:0 to 60:40, *v/v*) to afford the product **1w** as a yellow oil (335 mg, 1.49 mmol, 21% yield over 3 steps).

***R**f* 0.59 (cyclohexane/EtOAc, 8:2, *v/v*). **^{1}H NMR** (400 MHz, $CDCl_3$) δ 7.40–7.34 (m, 2H), 7.34–7.27 (m, 2H), 7.25–7.19 (m, 2H), 7.14 (ddd, *J* = 8.0, 7.4, 1.7 Hz, 1H), 6.94 (td, *J* = 7.5, 1.2 Hz, 1H), 6.81 (dd, *J* = 8.0, 1.3 Hz, 1H), 6.52 (dd, *J* = 16.1, 1.0 Hz, 1H), 6.44 (dd, *J* = 16.0, 5.9 Hz, 1H), 5.00 (s, 1H), 3.91 (p, *J* = 6.5 Hz, 1H), 1.50 (d, *J* = 7.1 Hz, 3H). **^{13}C NMR** (101 MHz, $CDCl_3$) δ 153.7, 137.3, 134.1, 129.5, 128.7, 128.7, 128.1, 127.8, 127.5, 126.4, 121.2, 116.2, 36.9, 19.6. **HRMS** (ESI –) calculated for $[C_{16}H_{15}O]$ $[M–H]^-$ 223.1128 *m/z*; found 223.1126 *m/z.*

4.2 Synthesis of 2-vinyl chromanes (2)

2-Phenyl-3-vinylchromane

*Racemic product **2a***

Prepared following the general procedure **GP3** using 2-cinnamylphenol **1a** (52.6 mg, 0.250 mmol, 1.0 equiv), JohnPhosAuCl (8.0 mg, 15 µmol, 0.06 equiv), $NaBAr^F_4$ (13.3 mg, 15.0 µmol, 0.06 equiv) and 1 bar of acetylene gas in $CHCl_3$ (0.63 mL, 0.4 M) at 23 ºC for 16 h. The crude product was purified by flash column chromatography (SiO_2, cyclohexane/EtOAc 100:0 to 95:5, *v/v*) and the product **2a** was obtained as a yellow solid (46.0 mg, 0.190 mmol, 78% yield).

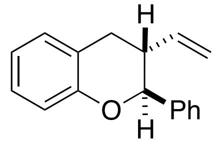

R_f 0.69 (cyclohexane/EtOAc, 8:2, *v/v*). **M.p.** 69–72 ºC. **^{1}H NMR** (400 MHz, $CDCl_3$) δ 7.42–7.32 (m, 5H), 7.17 (ddt, *J* = 8.2, 1.6, 0.7 Hz, 1H), 7.15–7.12 (m, 1H), 6.95–6.89 (m, 2H), 5.61 (ddd, *J* = 17.4, 10.5, 7.1 Hz, 1H), 5.03 (dt, *J* = 10.2, 1.4 Hz, 1H), 5.01–4.99 (m, 1H), 4.81 (d, *J* = 8.9 Hz, 1H), 2.99–2.90 (m, 2H), 2.89–2.79 (m, 1H). **^{13}C NMR** (126 MHz, $CDCl_3$) δ 154.8, 140.1, 137.7, 129.5, 128.5, 128.3, 127.6, 127.4, 121.5, 120.6, 116.7, 116.5, 82.2, 42.6, 31.0. **HRMS** (ESI +) calculated for [$C_{17}H_{17}O$] $[M+H]^+$ 237.1274 *m/z*; found 237.1270 *m/z.*

*Enantioenriched product **2a***

Prepared following the general procedure **GP4** using 2-cinnamylphenol **1a** (52.6 mg, 0.250 mmol, 1.0 equiv**),** **E** (2.8 mg, 2.5 μmol, 0.01 equiv), $NaBAr^F_4$ (2.2 mg, 2.50 μmol, 0.01 equiv) and 1 bar of acetylene gas in 1,2-DCE (0.42 mL, 0.6 M) at 23 ºC for 8 h. The crude product was purified by flash column chromatography (SiO_2, cyclohexane/EtOAc 100:0 to 95:5, *v/v*) and the product **2a** was obtained as a yellow solid (50.2 mg, 0.212 mmol, 85% yield).

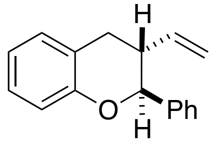

Characterization data matches the racemic product **2a**. **[α]$^{25.0}_D$** +17.9. (c 1.0, CH_2Cl_2, sample with 84:16 er). **SFC** (IG (100 × 3 mm, 3 μm), CO_2:MeOH 80:20, 1.2 mL/min, 25 ºC, BPR 150 bar, 210 nm): en1 (minor, 16%) min 0.90, en2 (major, 84%) min 0.98.

6-(*tert*-Butyl)-2-phenyl-3-vinylchromane (2b)

Prepared following the general procedure **GP3** using 4-(*tert*-butyl)-2-cinnamylphenol **1b** (66.6 mg, 0.250 mmol, 1.0 equiv), JohnPhosAuCl (8.0 mg, 15 μmol, 0.06 equiv), $NaBAr^F_4$ (13.3 mg, 15.0 μmol, 0.06 equiv) and 1 bar of acetylene gas in $CHCl_3$ (0.63 mL, 0.4 M) at 2 ºC for 16 h. The crude product was purified by flash column chromatography (SiO_2, cyclohexane/EtOAc 100:0 to 95:5, *v/v*) and the product **2b** was obtained as a yellow solid (51.0 mg, 0.170 mmol, 70% yield).

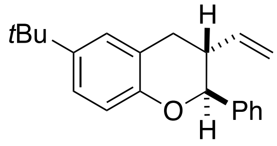

R_f 0.81 (cyclohexane/EtOAc, 8:2, *v/v*). **M.p.** 127–130 ºC. **^{1}H NMR** (500 MHz, $CDCl_3$) δ 7.43–7.30 (m, 5H), 7.21–7.16 (m, 1H), 7.14 (dt, *J* = 2.1, 0.9 Hz, 1H), 6.86 (d, *J* = 8.5 Hz, 1H), 5.58 (ddd, *J* = 17.5, 10.5, 7.2 Hz, 1H), 5.01 (dt, *J* = 11.3, 1.4 Hz, 1H), 4.98 (dt, *J* = 4.5, 1.4 Hz, 1H), 4.76 (d, *J* = 9.3 Hz, 1H), 2.98–2.90 (m, 2H), 2.89–2.79 (m, 1H), 1.33 (s, 9H). **^{13}C NMR** (126 MHz, $CDCl_3$) δ 152.6, 143.3, 140.2, 137.8, 128.5, 128.3, 127.5, 126.2, 124.6, 120.6, 116.4, 116.2, 82.3, 42.9, 34.2, 31.7, 31.5. **HRMS** (ESI +) calculated for [$C_{21}H_{25}O$] $[M+H]^+$ 293.1900 *m/z*; found 293.1892 *m/z.*

6-Methoxy-2-phenyl-3-vinylchromane (2c)

Prepared following the general procedure **GP3** using 2-cinnamyl-4-methoxyphenol **1c** (60.1 mg, 0.250 mmol, 1.0 equiv), JohnPhosAuCl (8.0 mg, 15 μmol, 0.06 equiv), $NaBAr^F_4$ (13.3 mg, 15.0 μmol, 0.06 equiv) and 1 bar of acetylene gas in $CHCl_3$ (0.63 mL, 0.4 M) at 23 ºC for 16 h. The crude product was purified by flash column chromatography (SiO_2, cyclohexane/EtOAc 100:0 to 95:5, *v/v*) and the product **2c** was obtained as a yellow solid (43.0 mg, 0.160 mmol, 65% yield).

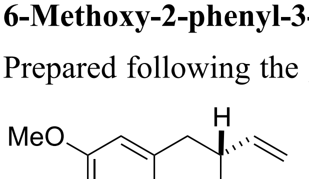

R_f 0.70 (cyclohexane/EtOAc, 8:2, *v/v*). **M.p.** 102–104 ºC. **^{1}H NMR** (500 MHz, $CDCl_3$) δ 7.41–7.30 (m, 5H), 6.85 (d, *J* = 8.9 Hz, 1H), 6.73 (dd, *J* = 8.9, 3.0 Hz, 1H), 6.69–6.65 (m, 1H), 5.59 (ddd, *J* = 17.4, 10.5, 7.1 Hz, 1H), 5.02 (dt, *J* = 11.9, 1.3 Hz, 1H), 4.99 (dt, *J* = 5.2, 1.3 Hz, 1H),

4.75 (d, J = 8.9 Hz, 1H), 3.78 (s, 3H), 2.89 (d, J = 1.4 Hz, 2H), 2.88–2.79 (m, 1H). **^{13}C NMR** (126 MHz, $CDCl_3$) δ 153.6, 148.9, 140.2, 137.8, 128.5, 128.5, 128.2, 127.4, 122.0, 117.3, 116.5, 114.0, 113.6, 55.9, 42.7, 31.3. **HRMS** (ESI +) calculated for [$C_{18}H_{18}NaO_2$] $[M+Na]^+$ 289.1199 *m/z*; found 289.1202 *m/z.*

4,4,5,5-Tetramethyl-2-(2-phenyl-3-vinylchroman-6-yl)-1,3,2-dioxaborolane

Racemic product ***2d***

Prepared following the general procedure **GP3** using 2-cinnamyl-4-(4,4,5,5-tetramethyl-1,3,2-dioxaborolan-2-yl)phenol **1d** (84.1 mg, 0.250 mmol, 1.0 equiv), JohnPhosAuCl (8.0 mg, 15 µmol, 0.06 equiv), $NaBAr^F{}_4$ (13.3 mg, 15.0 µmol, 0.06 equiv) and 1 bar of acetylene gas in $CHCl_3$ (0.63 mL, 0.4 M) at 23 ºC for 16 h. The crude product was purified by flash column chromatography (SiO_2, cyclohexane/EtOAc 100:0 to 95:5, *v/v*) and the product **2d** was obtained as a sticky yellow solid (51.0 mg, 0.140 mmol, 56% yield).

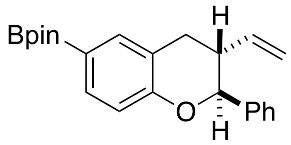

Rf 0.76 (cyclohexane/EtOAc, 8:2, *v/v*). **^{1}H NMR** (500 MHz, $CDCl_3$) δ 7.62–7.58 (m, 2H), 7.41–7.30 (m, 5H), 6.90 (dd, J = 8.2, 3.1 Hz, 1H), 5.58 (dd, J = 10.4, 7.0 Hz, 1H), 5.01 (dt, J = 9.7, 1.3 Hz, 1H), 5.00–4.96 (m, 1H), 4.84 (d, J = 8.6 Hz, 1H), 2.89 (d, J = 7.6 Hz, 2H), 2.84–2.77 (m, 1H), 1.35 (s, 12H). **^{13}C NMR** (126 MHz, $CDCl_3$) δ 157.6, 140.0, 137.7, 136.6, 134.5, 128.5, 128.3, 127.3, 120.8, 116.6, 116.2, 83.7, 82.3, 42.5, 30.5, 27.1, 25.0. **HRMS** (ESI +) calculated for [$C_{23}H_{27}NaBO_3$] $[M+Na]^+$ 385.1945 *m/z*; found 385.1949 *m/z.*

Enantioenriched product ***2d***

Prepared following the general procedure **GP4** using 2-cinnamyl-4-(4,4,5,5-tetramethyl-1,3,2-dioxaborolan-2-yl)phenol **1d** (84.1 mg, 0.250 mmol, 1.0 equiv), **E** (2.8 mg, 2.5 µmol, 0.01 equiv), $NaBAr^F{}_4$ (2.2 mg, 2.50 µmol, 0.01 equiv) and 1 bar of acetylene gas in 1,2-DCE (0.42 mL, 0.6 M) at 23 ºC for 8 h. The crude product was purified by flash column chromatography (SiO_2, cyclohexane/EtOAc 100:0 to 95:5, *v/v*) and the product **2d** was obtained as a sticky yellow solid (57.0 mg, 0.160 mmol, 63% yield).

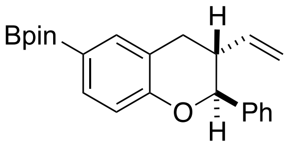

Characterization data matches the racemic product **2d**. **$[\alpha]^{25.0}_D$** –6.0. (c 1.1, CH_2Cl_2, sample with 87:13 er). **SFC** (IG (100 × 3 mm, 3 µm), CO_2:MeOH 80:20, 1.2 mL/min, 25 ºC, BPR 150 bar, 210 nm): en1 (minor, 16%) min 0.90, en2 (major, 84%) min 0.98.

2-Phenyl-3-vinyl-6-bromochromane

Racemic product ***2e***

Prepared following the general procedure **GP3** using 4-bromo-2-cinnamylphenol **1e** (72.3 mg, 0.250 mmol, 1.0 equiv), JohnPhosAuCl (8.0 mg, 15 µmol, 0.06 equiv), $NaBAr^F{}_4$ (13.3 mg, 15.0 µmol, 0.06 equiv) and 1 bar of acetylene gas in $CHCl_3$ (0.63 mL, 0.4 M) at 23 ºC for 16 h. The crude product was purified by flash column chromatography (SiO_2, cyclohexane/EtOAc 100:0 to 95:5, *v/v*) and the product **2e** was obtained as a yellow solid (62.0 mg, 0.197 mmol, 79% yield).

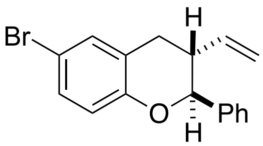

Rf 0.50 (cyclohexane/EtOAc, 9:1, *v/v*). **M.p.** 97–100 ºC. **^{1}H NMR** (500 MHz, $CDCl_3$) δ 7.40–7.30 (m, 5H), 7.24–7.20 (m, 2H), 6.78 (d, J = 8.4 Hz, 1H), 5.61–5.53 (m, 1H), 5.03–5.01 (m, 1H), 5.00–

4.98 (m, 1H), 4.78 (d, J = 8.7 Hz, 1H), 2.91–2.84 (m, 2H), 2.83–2.75 (m, 1H). **^{13}C NMR** (126 MHz, $CDCl_3$) δ 154.0, 139.7, 137.2, 132.0, 130.5, 128.6, 128.5, 127.3, 123.7, 118.5, 116.9, 112.6, 82.2, 42.2, 30.6. **HRMS** (ESI +) calculated for [$C_{17}H_{15}BrNaO$] $[M+Na]^+$ 337.0198 *m/z*; found 337.0182 *m/z*.

*Enantioenriched product **2e***

Prepared following the general procedure **GP4** using 4-bromo-2-cinnamylphenol**1e** **1e** (84.1 mg, 0.250 mmol, 1.0 equiv), **E** (2.8 mg, 2.5 μmol, 0.01 equiv), NaBAr$^F{}_4$ (2.2 mg, 2.50 μmol, 0.01 equiv) and 1 bar of acetylene gas in 1,2-DCE (0.42 mL, 0.6 M) at 23 ºC for 8 h. The crude product was purified by flash column chromatography (SiO_2, cyclohexane/EtOAc 100:0 to 95:5, *v/v*) and the product **2d** was obtained as a sticky yellow solid (57.0 mg, 0.160 mmol, 63% yield).

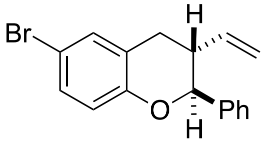

Characterization data matches the racemic product **2e**. **[α]$^{25.0}_D$** 32.8 (c 0.99, CH_2Cl_2, sample with 87:13 er). **SFC** (IB-N (100 × 3 mm, 3 μm), CO_2:MeOH 95:5, 1.2 mL/min, 25 ºC, BPR 150 bar, 210 nm): en1 (major, 87%) min 2.42, en2 (minor, 13%) min 2.60.

Methyl-2-phenyl-3-vinylchromane-6-carboxylate (2f)

Prepared following the general procedure **GP3** using methyl 3-cinnamyl-4-hydroxybenzoate **1f** (67.1 mg, 0.250 mmol, 1.0 equiv), JohnPhosAuCl (8.0 mg, 15 μmol, 0.06 equiv), NaBAr$^F{}_4$ (13.3 mg, 15.0 μmol, 0.06 equiv) and 1 bar of acetylene gas in $CHCl_3$ (0.63 mL, 0.4 M) at 23 ºC for 16 h. The crude product was purified by flash column chromatography (SiO_2, cyclohexane/EtOAc 100:0 to 95:5, *v/v*) and the product **2f** was obtained as a white solid (15.0 mg, 0.051 mmol, 20% yield).

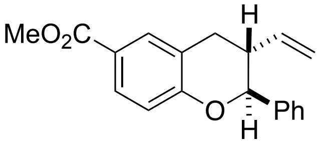

R$_f$ 0.61 (cyclohexane/EtOAc, 8:2, *v/v*). **M.p.** 108–112 ºC. **^{1}H NMR** (500 MHz, $CDCl_3$) δ 7.87–7.80 (m, 2H), 7.42–7.35 (m, 3H), 7.37–7.30 (m, 2H), 6.91 (d, *J* = 8.5 Hz, 1H), 5.63–5.54 (m, 1H), 5.04 (dt, *J* = 7.1, 1.2 Hz, 1H), 5.01 (d, *J* = 1.1 Hz, 1H), 4.86 (d, *J* = 8.8 Hz, 1H), 3.89 (s, 3H), 2.92 (d, *J* = 7.6 Hz, 2H), 2.86–2.77 (m, 1H). **^{13}C NMR** (126 MHz, $CDCl_3$) δ 167.1, 158.8, 139.5, 137.1, 131.7, 129.5, 128.6, 128.5, 127.3, 122.5, 121.3, 117.0, 116.7, 82.6, 52.0, 42.2, 30.6. **HRMS** (ESI +) calculated for [$C_{19}H_{18}NaO_3$] [M+Na]$^+$ 317.1148 *m/z*; found 317.1158 *m/z.*

2-Phenyl-6-(trifluoromethyl)-3-vinylchromane-6-carboxylate (2g)

Prepared following the general procedure **GP3** using methyl 2-cinnamyl-(4-trifluroromethyl)phenol **1g** (140.0 mg, 0.503 mmol, 1.0 equiv), JohnPhosAuCl (16.0. mg, 30.2 μmol, 0.06 equiv), NaBAr$^F{}_4$ (26.8 mg, 30.2 μmol, 0.06 equiv) and 1 bar of acetylene gas in $CHCl_3$ (1.26 mL, 0.4 M) at 23 ºC for 16 h. The crude product was purified by flash column chromatography (SiO_2, cyclohexane/EtOAc 100:0 to 95:5, *v/v*) and the product **2g** was obtained as a white solid (51.0 mg, 0.170 mmol, 33% yield).

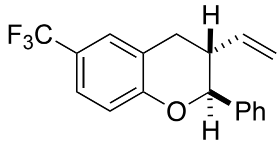

R$_f$ 0.89 (cyclohexane/EtOAc, 8:2, *v/v*).**M.p.** 101–106 ºC. **^{1}H NMR (**400 MHz, $CDCl_3$) δ 7.42–7.31 (m, 8H), 6.96 (d, *J* = 9.1 Hz, 1H), 5.58 (ddd, *J* = 17.4, 10.2, 7.1 Hz, 1H), 5.04 (dt, *J* = 4.7, 1.2 Hz, 1H), 5.01 (p, *J* = 1.3 Hz, 1H), 4.85 (d, *J* = 8.8 Hz, 1H), 2.92 (d, *J* = 7.9 Hz, 2H), 2.87–2.78 (m, 1H). **^{13}C NMR** (126 MHz, $CDCl_3$) δ 157.4, 139.4, 137.0, 128.6, 128.6, 127.3, 126.9 (q, $J_{C–F}$ = 3.8 Hz), 124.9 (q, $J_{C–F}$ = 3.7 Hz) 124.7 (q, $J_{C–F}$ = 271.5 Hz), 122.9 (q, $J_{C–F}$ = 31.6 Hz), 121.8, 117.1, 117.0, 82.5, 42.1, 30.7. **^{19}F NMR** (376 MHz, $CDCl_3$) δ –61.6. **HRMS** (ESI +) calculated for [$C_{18}H_{16}F_3O$] [M+H]$^+$ 305.1148 *m/z*; found 305.1146 *m/z.*

2-Phenyl-3-vinyl-7-methylchromane (2h)

Prepared following the general procedure **GP3** using 2-cinnamyl-5-methylphenol **1h** (60.1 mg, 0.250 mmol, 1.0 equiv), JohnPhosAuCl (8.0 mg, 15 µmol, 0.06 equiv), $NaBAr^F_4$ (13.3 mg, 15.0 µmol, 0.06 equiv) and 1 bar of acetylene gas in $CHCl_3$ (0.63 mL, 0.4 M) at 23 °C for 16 h. The crude product was purified by flash column chromatography (SiO_2, cyclohexane/EtOAc, 100:0 to 95:5, *v/v*) and the product **2h** was obtained as a yellow oil (34.0 mg, 0.135 mmol, 54% yield).

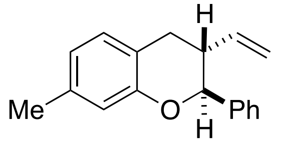

R_f 0.44 (cyclohexane/EtOAc, 9:1, *v/v*). **^{1}H NMR** (400 MHz, $CDCl_3$) δ 7.41–7.32 (m, 6H), 7.03 (d, *J* = 7.5 Hz, 1H), 6.78–6.72 (m, 2H), 5.61 (ddd, *J* = 17.3, 10.5, 6.9 Hz, 1H), 5.06–4.98 (m, 2H), 4.80 (d, *J* = 8.6 Hz, 1H), 2.92–2.77 (m, 3H), 2.33 (s, 3H). **^{13}C NMR** (101 MHz, $CDCl_3$) δ 154.6, 140.2, 137.9, 137.5, 129.3, 128.5, 128.2, 127.4, 121.6, 118.3, 117.1, 116.5, 82.1, 42.8, 30.7, 21.3. **HRMS** (APCI +) calculated for [$C_{18}H_{19}O$] $[M+H]^+$ 251.1430 *m/z*; found 251.1429 *m/z.*

2-Phenyl-3-vinyl-5-methoxychromane (2i)

Prepared following the general procedure **GP3** 10 ml microwave vial equipped with a stirrer bar was added phenol **1i** (56.1 mg, 0.250 mmol, 1.0 equiv), JohnPhosAuCl (8.0 mg, 15 µmol, 0.06 equiv), $NaBAr^F_4$ (13.3 mg, 15.0 µmol, 0.06 equiv) and $CHCl_3$ (0.63 mL, 0.4 M). The resulting solution was stirred at 0 °C for 15 min before acetylene gas was added *via* balloon. The reaction solution was stirred at 0 °C for 3 h before being quenched by addition of Et_3N. The reaction mixture was concentrated *in vacuo* and subsequently analysed by ^{1}H NMR spectroscopy (1,1,2,2-tetrachloroethane as internal standard). The crude product was purified by flash column chromatography (SiO_2, cyclohexane/DCM, 8:2 to 1:1, *v/v*) and the product **2i** was obtained as a white solid (19.6 mg, 0.074 mmol, 29% yield).

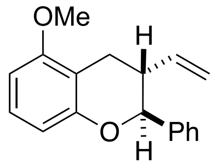

R_f 0.48 (cyclohexane/EtOAc, 9:1, *v/v*). **M.p.** 111–112 °C. **^{1}H NMR** (500 MHz, $CDCl_3$) δ 7.40–7.30 (m, 5H), 7.10 (d, *J* = 8.2 Hz, 1H), 6.57 (d, *J* = 8.3 Hz, 1H), 6.47 (d, *J* = 8.2 Hz, 1H), 5.59 (ddd, *J* = 17.5, 10.5, 7.3 Hz, 1H), 5.02 (d, *J* = 17.3 Hz, 1H), 4.98 (d, *J* = 10.5 Hz, 1H), 4.72 (d, *J* = 9.2 Hz, 1H), 3.85 (s, 3H), 2.95 (dd, *J* = 16.8, 5.1 Hz, 1H), 2.80–2.72 (m, 1H), 2.64 (dd, *J* = 16.8, 10.6 Hz, 1H). **^{13}C NMR** (126 MHz, $CDCl_3$) δ 158.0, 155.7, 140.1, 137.9, 128.5, 128.3, 127.5, 127.2, 116.5, 110.6, 109.6, 102.2, 81.8, 55.7, 42.3, 25.6. **HRMS** (ESI +) calculated for [$C_{18}H_{18}NaO_2$] $[M+Na]^+$ 289.1199 *m/z*; found 289.1194 *m/z.*

6,8-Dimethyl-2-phenyl-3-vinylchromane (2j)

Prepared following the general procedure **GP3** using methyl 2-cinnamyl-4,6-dimethylphenol **1j** (56.6 mg, 0.250 mmol, 1.0 equiv), JohnPhosAuCl (8.0 mg, 15 µmol, 0.06 equiv), $NaBAr^F_4$ (13.3 mg, 15.0 µmol, 0.06 equiv) and 1 bar of acetylene gas in $CHCl_3$ (0.63 mL, 0.4 M) at 23 °C for 16 h. The crude product was purified by flash column chromatography (SiO_2, cyclohexane/EtOAc 100:0 to 95:5, *v/v*) and the product **2j** was obtained as a yellow oil (43.0 mg, 0.160 mmol, 45% yield).

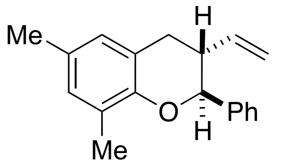

R_f 0.73 (cyclohexane/EtOAc, 8:2, *v/v*). **^{1}H NMR** (500 MHz, $CDCl_3$) δ 7.42–7.31 (m, 5H), 6.86 (d, *J* = 2.2 Hz, 1H), 6.80–6.78 (m, 1H), 5.68–5.60 (m, 1H), 5.04–5.02 (m, 1H), 5.01–4.99 (m, 1H), 4.82 (d, *J* = 8.8 Hz, 1H), 2.93–2.83 (m, 2H), 2.83–2.72 (m, 1H), 2.29 (s, 3H), 2.20 (s, 3H). **^{13}C NMR** (126 MHz, $CDCl_3$) δ 150.7, 140.8, 138.1, 129.5, 129.0, 128.3, 128.0, 127.3, 127.2, 125.6,

120.5, 116.3, 81.9, 43.1, 31.0, 20.6, 16.1. **HRMS** (ESI +) calculated for [$C_{19}H_{21}O$] [M+H]$^+$ 265.1587 *m/z*; found 265.1581 *m/z.*

2-Phenyl-3-vinyl-5,7-dimethylchromane (2k)

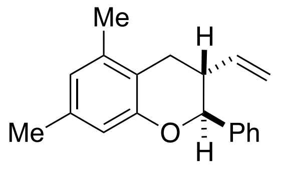

Prepared following the general procedure **GP3** using 2-cinnamyl-3,5-dimehtylphenol **1k** (59.6 mg, 0.250 mmol, 1.0 equiv), JohnPhosAuCl (8.0 mg, 15 µmol, 0.06 equiv), NaBAr$^F{}_4$ (13.3 mg, 15.0 µmol, 0.06 equiv.) and 1 bar of acetylene gas in $CHCl_3$ (0.63 mL, 0.4 M) at 23 ºC for 16 h. The crude product was purified by flash column chromatography (SiO_2, cyclohexane/$CHCl_3$, 100:0 to 8:2, *v/v*) and the product **2k** was obtained as a white solid (29.0 mg, 0.110 mmol, 44% yield).

R$_f$ 0.38 (cyclohexane/$CHCl_3$, 8:2, *v/v*). **M.p.** 91–93 ºC. **^{1}H NMR** (500 MHz, $CDCl_3$) δ 7.39–7.29 (m, 5H), 6.62 (s, 1H), 6.61 (s, 1H), 5.60 (ddd, *J* = 17.4, 10.5, 7.4 Hz, 1H), 5.02–4.95 (m, 2H), 4.70 (d, *J* = 8.9 Hz, 1H), 2.84–2.75 (m, 2H), 2.69–2.61 (m, 1H), 2.26 (s, 3H), 2.22 (s, 3H). **^{13}C NMR** (126 MHz, $CDCl_3$) δ 154.8, 140.2, 138.1, 137.2, 136.9, 128.5, 128.2, 127.5, 123.2, 117.1, 116.4, 114.9, 81.5, 43.1, 28.8, 21.1, 19.2. **HRMS** (APCI +) calculated for [$C_{19}H_{21}O$] [M+H]$^+$ 265.1587 *m/z*; found 165.1582 *m/z.*

2-(2-Phenyl-3-vinylchroman-8-yl)phenol (2l)

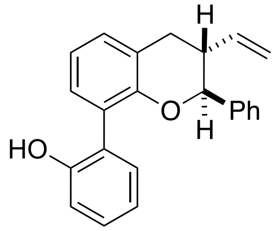

Prepared following the general procedure **GP3** using 3-cinnamyl-(1,1'-biphenyl)-2,2'-diol **1l** (75.6 mg, 0.250 mmol, 1.0 equiv), JohnPhosAuCl (8.0 mg, 15 µmol, 0.06 equiv), NaBAr$^F{}_4$ (13.3 mg, 15.0 µmol, 0.06 equiv) and 1 bar of acetylene gas in $CHCl_3$ (0.63 mL, 0.4 M) at 23 ºC for 16 h. The crude product was purified by flash column chromatography (SiO_2, cyclohexane/EtOAc 100:0 to 95:5, *v/v*) and the product **2l** was obtained as a yellow sticky solid (34.0 mg, 0.094 mmol, 38% yield).

R$_f$ 0.55 (cyclohexane/EtOAc, 8:2, *v/v*). **^{1}H NMR** (500 MHz, $CDCl_3$) δ 7.3–7.27 (m, 7H), 7.25 –7.16 (m, 2H), 7.06 (t, *J* = 7.5 Hz, 1H), 6.99 (td, *J* = 7.5, 1.3 Hz, 1H), 6.94 (dd, *J* = 8.1, 1.3 Hz, 1H), 6.44 (s, 1H), 5.65 (ddd, *J* = 17.2, 10.5, 6.7 Hz, 1H), 5.11–5.07 (m, 1H), 5.07–5.04 (m, 1H), 4.98 (d, *J* = 8.0 Hz, 1H), 3.03–2.98 (m, 2H), 2.98–2.93 (m, 1H). **^{13}C NMR** (126 MHz, $CDCl_3$) δ 154.0, 150.8, 139.0, 137.2, 131.4, 130.8, 129.5, 129.1, 128.6, 128.4, 127.0, 126.6, 126.5, 122.0, 121.7, 121.0, 117.7, 117.0, 82.9, 42.1, 30.5. **HRMS** (ESI –) calculated for [$C_{23}H_{19}O_2$] [M–H]$^-$ 327.1391 *m/z*; found 327.1389 *m/z.*

2-(3,5-Di-*tert*-butylphenyl)-3-vinylchromane (2m)

*Racemic product (**2m**)*

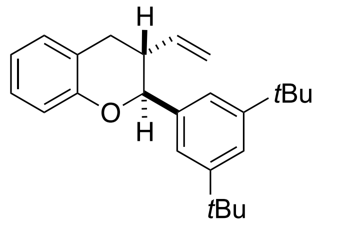

Prepared following the general procedure **GP3** using 2-cinnamyl-3,5-dimehtylphenol **1m** (80.6 mg, 0.250 mmol, 1.0 equiv), JohnPhosAuCl (8.0 mg, 15 µmol, 0.06 equiv), NaBAr$^F{}_4$ (13.3 mg, 15.0 µmol, 0.06 equiv) and 1 bar of acetylene gas in $CHCl_3$ (0.63 mL, 0.4 M) at 23 ºC for 16 h. The crude product was purified by flash column chromatography (SiO_2, cyclohexane/EtOAc, 100:0 to 95:5, *v/v*) and the product **2m** was obtained as a yellow oil (74.0 mg, 0.212 mmol, 85% yield).

R$_f$ 0.61 (cyclohexane/EtOAc, 9:1, *v/v*). **^{1}H NMR** (500 MHz, $CDCl_3$) δ 7.40–7.39 (m, 1H), 7.22–7.20 (m, 2H), 7.18–7.12 (m, 2H), 6.97–6.89 (m, 2H), 5.59 (ddd, *J* = 17.3, 10.5, 6.9 Hz, 1H), 5.05–

4.97 (m, 2H), 4.78 (d, *J* = 9.1 Hz, 1H), 3.00–2.83 (m, 3H), 1.35 (s, 18H). **^{13}C NMR** (126 MHz, $CDCl_3$) δ 155.1, 150.8, 138.8, 138.0, 129.5, 127.5, 122.2, 121.8, 121.6, 120.5, 116.8, 116.1, 83.0, 42.3, 35.0, 31.6, 31.1. **HRMS** (APCI +) calculated for [$C_{25}H_{33}O$] $[M+H]^+$ 349.2526 *m/z*; found 349.2523 *m/z*.

*Enantioenriched product (**2m**)*

Prepared following the general procedure **GP4** using 2-cinnamyl-3,5-dimehtylphenol **1m** (80.6 mg, 0.250 mmol, 1.0 equiv), **E** (2.8 mg, 2.50 μmol, 0.01 equiv), $NaBAr^F_4$ (2.2 mg, 2.5 μmol, 0.01 equiv) and 1 bar of acetylene gas in 1,2-DCE (0.42 mL, 0.6 M) at 23 ºC for 8 h. The crude product was purified by flash column chromatography (SiO_2, cyclohexane/EtOAc, 100:0 to 95:5, *v/v*) and the product **2m** was obtained as a yellow oil (81.5 mg, 0.234 mmol, 94% yield).

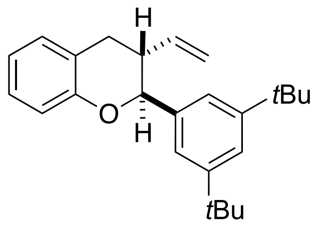

Characterization data matches the racemic product **2m**. $[\alpha]^{25.0}_D$ +12.1. (c 1.0, CH_2Cl_2, sample with 77:23 er). **SFC** (OD (100 × 3 mm, 3 μm), CO_2:i-PrOH 95:5, 1.2 mL/min, 35 ºC, BPR 150 bar, 210 nm): en1 (minor, 77%) min 0.96, en2 (major, 23%) min 1.12.

2-(4-*iso*-Propylphenyl)-3-vinylchromane (2n)

Prepared following the general procedure **GP3** using (*E*)-2-(3-(4-*iso*-propylphenyl)allyl)phenol **1n** (63.1 mg, 0.250 mmol, 1.0 equiv), JohnPhosAuCl (8.0 mg, 15 μmol, 0.06 equiv), $NaBAr^F_4$ (13.3 mg, 15.0 μmol, 0.06 equiv) and 1 bar of acetylene gas in $CHCl_3$ (0.63 mL, 0.4 M) at 23 ºC for 16 h. The crude product was purified by flash column chromatography (SiO_2, cyclohexane/EtOAc, 100:0 to 95:5, *v/v*) and the product **2n** was obtained as a white solid (61.0 mg, 0.219 mmol, 88% yield).

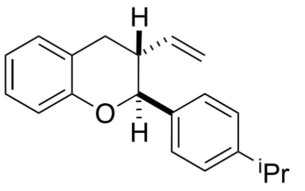

Rf 0.53 (cyclohexane/EtOAc, 9:1, *v/v*). **M.p.** 85–88 ºC. **^{1}H NMR** (500 MHz, $CDCl_3$) δ 7.30–7.27 (m, 2H), 7.25–7.21 (m, 2H), 7.15–7.09 (m, 2H), 6.91–6.87 (m, 2H), 5.60 (ddd, *J* = 17.4, 10.5, 6.9 Hz, 1H), 5.06–4.98 (m, 2H), 4.76 (d, *J* = 9.0 Hz, 1H), 2.96–2.79 (m, 4H), 1.26 (d, *J* = 6.9 Hz, 6H). **^{13}C NMR** (126 MHz, $CDCl_3$) δ 154.9, 149.0, 137.9, 137.4, 129.5, 127.6, 127.4, 126.6, 121.5, 120.5, 116.7, 116.3, 82.1, 42.3, 34.0, 31.0, 24.1. **HRMS** (APCI +) calculated for $[C_{20}H_{23}O]$ $[M+H]^+$ 279.1743 *m/z*; found 279.1740 *m/z.*

2-(4-Methoxyphenyl)-3-vinylchromane (2o)

Prepared following the general procedure **GP3** using 2-(3-(4-methoxyphenyl)allyl)phenol **1o** (60.1 mg, 0.250 mmol, 1.0 equiv), JohnPhosAuCl (8.0 mg, 15 μmol, 0.06 equiv), $NaBAr^F_4$ (13.3 mg, 15.0 μmol, 0.06 equiv) and 1 bar of acetylene gas in $CHCl_3$ (0.63 mL, 0.4 M) at 23 ºC for 16 h. The crude product was purified by flash column chromatography (SiO_2, cyclohexane/EtOAc 100:0 to 95:5, *v/v*) and a second flash column chromatography was needed (SiO_2, cyclohexane/toluene 100:0 to 30:10, *v/v*) to obtain the product **2o** as a colorless oil (42.0 mg, 0.160 mmol, 63% yield).

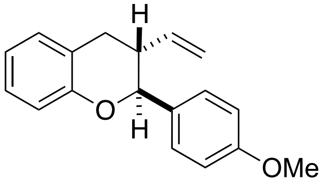

Rf 0.62 (cyclohexane/EtOAc, 8:2, *v/v*).**^{1}H NMR** (500 MHz, $CDCl_3$) δ 7.31–7.27 (m, 2H), 7.15–7.09 (m, 2H), 6.93–6.89 (m, 2H), 6.88 (dd, *J* = 7.8, 1.6 Hz, 2H), 5.61–5.50 (m, 1H), 5.05–4.95 (m, 2H), 4.73 (d, *J* = 9.3 Hz, 1H), 3.82 (s, 3H), 2.95–2.87 (m, 2H), 2.85–2.76 (m, 1H). **^{13}C NMR** (126 MHz, $CDCl_3$) δ 159.6, 155.0, 137.9, 132.2, 129.5, 128.7, 127.5, 121.5, 120.5, 116.7, 116.4, 114.0, 81.9, 55.4, 42.6, 31.3. **HRMS** (ESI +) calculated for $[C_{18}H_{18}NaO_2]$ $[M+Na]^+$ 289.1199 *m/z*; found 289.1199 *m/z.*

2-(4-Trifluoromethylphenyl)-3-vinylchromane (2p)

Prepared following the general procedure **GP3** using (*E*)-2-(3-(4-(trifluoromethyl)phenyl)allyl)phenol **1p** (69.6 mg, 0.250 mmol, 1.0 equiv), JohnPhosAuCl (8.0 mg, 15 μmol, 0.06 equiv), $NaBAr^F_4$ (13.3 mg, 15.0 μmol, 0.06 equiv) and 1 bar of acetylene gas in $CHCl_3$ (0.63 mL, 0.4 M) at 23 ºC for 16 h. The crude product was purified by flash column chromatography (SiO_2, cyclohexane/CH_2Cl_2, 100:0 to 9:1, *v/v*) and the product **2p** was obtained as a white solid (46.0 mg, 0.053 mmol, 21% yield).

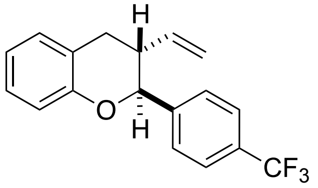

Rf 0.69 (cyclohexane/CH_2Cl_2, 9:1, *v/v*). **M.p.** 91–94 ºC. **^{1}H NMR** (500 MHz, $CDCl_3$) δ 7.63 (d, *J* = 8.1 Hz, 2H), 7.48 (d, *J* = 8.0 Hz, 2H), 7.17–7.10 (m, 2H), 6.94–6.88 (m, 2H), 5.57 (ddd, *J* = 17.1, 10.6, 7.5 Hz, 1H), 5.03–4.97 (m, 2H), 4.81 (d, *J* = 9.0 Hz, 1H), 2.97–2.85 (m, 2H), 2.82–2.74 (m, 1H). **^{13}C NMR** (126 MHz, $CDCl_3$) δ 154.3, 144.0, 137.0, 130.3 (q, $J_{C\text{–}F}$ = 32.3 Hz), 129.5, 129.1, 127.5, 125.4 (q, $J_{C\text{–}F}$ = 3.7 Hz), 124.1 (q, $J_{C\text{–}F}$ = 272.1 Hz), 121.1, 120.8, 117.2, 116.6, 81.3, 42.8, 30.8. **^{19}F NMR** (471 MHz, $CDCl_3$) δ –62.55. **HRMS** (APCI +) calculated for $[C_{18}H_{16}F_3O]$ $[M+H]^+$ 305.1148 *m/z*; found 305.1145 *m/z.*

2-(2-Methoxyphenyl)-3-vinylchromane (2q)

Prepared following the general procedure **GP3** using 2-(3-(2-methoxyphenyl)allyl)phenol **1q** (60.1 mg, 0.250 mmol, 1.0 equiv), JohnPhosAuCl (8.0 mg, 15 μmol, 0.06 equiv), $NaBAr^F_4$ (13.3 mg, 15.0 μmol, 0.06 equiv) and 1 bar of acetylene gas in $CHCl_3$ (0.63 mL, 0.4 M) at 23 ºC for 16 h. The crude product was purified by flash column chromatography (SiO_2, cyclohexane/EtOAc 100:0 to 95:5, *v/v*) and the product **2q** was obtained as a yellow solid (35.0 mg, 0.130 mmol, 53% yield).

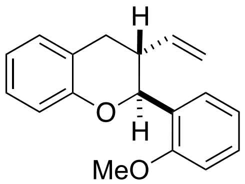

Rf 0.69 (cyclohexane/EtOAc, 8:2, *v/v*). **M.p.** 88–95 ºC. **^{1}H NMR** (500 MHz, $CDCl_3$) δ 7.40 (dd, *J* = 7.6, 1.8 Hz, 1H), 7.29 (ddd, *J* = 8.2, 7.4, 1.8 Hz, 1H), 7.16 – 7.10 (m, 2H), 6.99 (td, *J* = 7.5, 1.1 Hz, 1H), 6.92 – 6.86 (m, 3H), 5.72–5.64 (m, 1H), 5.40–5.36 (m, 1H), 4.98 (dt, *J* = 17.3, 1.4 Hz, 1H), 4.94 (ddd, *J* = 10.4, 1.7, 0.8 Hz, 1H), 3.83 (s, 3H), 2.95 – 2.87 (m, 2H), 2.87–2.82 (m, 1H).**^{13}C NMR** (126 MHz, $CDCl_3$) δ 156.9, 155.3, 138.1, 129.6, 129.0, 128.8, 127.7, 127.5, 121.6, 121.0, 120.4, 116.7, 116.0, 110.8, 75.4, 55.6, 42.5, 31.2. **HRMS** (ESI +) calculated for $[C_{18}H_{18}NaO_2]$ $[M+Na]^+$ 289.1199 *m/z*; found 289.1201 *m/z.*

2-(2-Bromophenyl)-3-vinylchromane (2r)

Prepared following the general procedure **GP3** using (*E*)-2-(3-(2-bromophenyl)allyl)phenol **1r** (72.3 mg, 0.250 mmol, 1.0 equiv), JohnPhosAuCl (8.0 mg, 15 μmol, 0.06 equiv), $NaBAr^F_4$ (13.3 mg, 15.0 μmol, 0.06 equiv) and 1 bar of acetylene gas in $CHCl_3$ (0.63 mL, 0.4 M) at 23 ºC for 16 h. The crude product was purified by flash column chromatography (SiO_2, cyclohexane/$CHCl_3$, 100:0 to 8:2, *v/v*) and the product **2r** was obtained as a white solid (24.0 mg, 0.076 mmol, 30% yield).

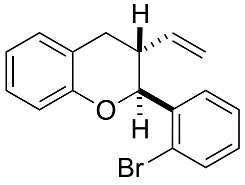

Rf 0.43 (cyclohexane/$CHCl_3$, 8:2, *v/v*). **M.p.** 58–61 ºC. **^{1}H NMR** (500 MHz, $CDCl_3$) δ 7.56 (dd, *J* = 8.1, 1.2 Hz, 1H), 7.46 (dd, *J* = 7.8, 1.8 Hz, 1H), 7.34 (ddd, *J* = 7.6, 7.5, 1.2 Hz, 1H), 7.19–7.10 (m, 3H), 6.93–6.87 (m, 2H), 5.72 (ddd, *J* = 16.9, 10.7, 7.4 Hz, 1H), 5.39 (d, *J* = 8.3, 1H), 5.01–4.96 (m, 2H), 2.98–2.82 (m, 3H). **^{13}C NMR** (126 MHz, $CDCl_3$) δ 154.8, 139.6, 137.1, 132.8,

129.6, 129.6, 128.7, 127.9, 127.7, 123.5, 121.3, 120.8, 117.0, 116.7, 80.0, 43.1, 30.9. **HRMS** (APCI +) calculated for [$C_{17}H_{16}BrO$] $[M+H]^+$ 315.0379 *m/z*; found 315.0373 *m/z*.

2-(2-Fluorophenyl)-3-vinylchromane (2s)

Prepared following the general procedure **GP3** using (*E*)-2-(3-(2-fluorophenyl)allyl)phenol **1s** (57.1 mg, 0.250 mmol, 1.0 equiv), JohnPhosAuCl (8.0 mg, 15 μmol, 0.06 equiv), $NaBAr^F_4$ (13.3 mg, 15.0 μmol, 0.06 equiv) and 1 bar of acetylene gas in $CHCl_3$ (0.63 mL, 0.4 M) at 23 ºC for 16 h. The crude product was purified by flash column chromatography (SiO_2, cyclohexane/EtOAc, 100:0 to 95:5, *v/v*) and the product **2s** was obtained as a colourless oil (36.0 mg, 0.142 mmol, 57% yield).

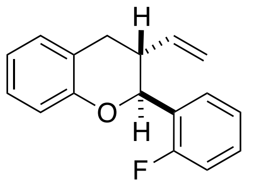

Rf 0.49 (cyclohexane/EtOAc, 9:1, *v/v*). **^{1}H NMR** (500 MHz, $CDCl_3$) δ 7.46 (ddd, *J* = 7.4, 7.4, 1.8 Hz, 1H), 7.33–7.27 (m, 1H), 7.21–7.10 (m, 3H), 7.08–7.03 (m, 1H), 6.93–6.88 (m, 2H), 5.69–5.61 (m, 1H), 5.20 (d, *J* = 9.1 Hz, 1H), 5.01–4.99 (m, 1H), 4.98–4.97 (m, 1H), 2.98–2.80 (m, 3H). **^{13}C NMR** (126 MHz, $CDCl_3$) δ 160.3 (d, $J_{C–F}$ = 246.8 Hz), 154.8, 137.1, 129.7 (d, $J_{C–F}$ = 8.3 Hz), 129.6, 128.5 (d, $J_{C–F}$ = 4.1 Hz), 127.6, 127.5 (d, $J_{C–F}$ =12.9 Hz), 124.5 (d, $J_{C–F}$ = 3.7 Hz), 121.4, 120.8, 117.1. 116.7, 115.4 (d, $J_{C–F}$ = 22.5 Hz), 75.3 (d, $J_{C–F}$ = 2.3 Hz), 42.9 (d, $J_{C–F}$ = 1.8 Hz), 31.4. **^{19}F NMR** (471 MHz, $CDCl_3$) δ −117.92. **HRMS** (APCI +) calculated for $[C_{17}H_{16}FO]$ $[M+H]^+$ 255.1180 *m/z*; found 255.1175 *m/z.*

2-Mesityl-3-vinylchromane (2t)

Prepared following the general procedure **GP3** using (*E*)-2-(3-mesitylallyl)phenol **1t** (63.1 mg, 0.250 mmol, 1.0 equiv), JohnPhosAuCl (8.0 mg, 15 μmol, 0.06 equiv), $NaBAr^F_4$ (13.3 mg, 15.0 μmol, 0.06 equiv) and 1 bar of acetylene gas in $CHCl_3$ (0.63 mL, 0.4 M) at 23 ºC for 16 h. The crude product was purified by flash column chromatography (SiO_2, cyclohexane/EtOAc 100:0 to 95:5, *v/v*) and the product **2t** was obtained as a yellow oil (40.0 mg, 0.144 mmol, 57% yield).

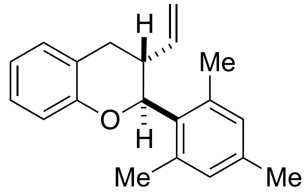

Rf 0.55 (cyclohexane/EtOAc, 9:1, *v/v*). **^{1}H NMR** (500 MHz, $CDCl_3$) δ 7.16–7.10 (m, 2H), 7.91–7.84 (m, 4H), 5.58 (ddd, *J* = 17.2, 10.5, 6.7 Hz, 1H), 5.21 (d, *J* = 10.6 Hz, 1H), 5.05 (dd, *J* = 17.3, 1.4 Hz, 1H), 4.97 (dd, *J* = 10.5, 1.3 Hz, 1H), 3.27–3.19 (m, 1H), 3.03–2.89 (m, 2H), 2.37 (s, 6H), 2.27 (s, 3H). **^{13}C NMR** (126 MHz, $CDCl_3$) δ 154.9, 137.5, 137.2, 137.1, 132.1, 130.4, 129.7, 127.6, 121.8, 120.4, 116.8, 116.1, 78.9, 39.0, 31.8, 21.2, 21.0. **HRMS** (APCI +) calculated for $[C_{20}H_{23}O]$ $[M+H]^+$ 279.1743 *m/z*; found 279.1743 *m/z.*

6-Methoxy-2,2-dimethyl-3-vinylchromane (2u)

Prepared following the general procedure **GP3** using 4-methoxy-2-(3-methylbut-2-en-1-yl)phenol **1u** (48.1 mg, 0.250 mmol, 1.0 equiv), JohnPhosAuCl (8.0 mg, 15 μmol, 0.06 equiv), $NaBAr^F_4$ (13.3 mg, 15.0 μmol, 0.06 equiv) and 1 bar of acetylene gas in $CHCl_3$ (0.63 mL, 0.4 M) at 23 ºC for 16 h. The crude product was purified by flash column chromatography (SiO_2, cyclohexane/EtOAc 100:0 to 95:5, *v/v*) and the product **2u** was obtained as a yellow oil (26.0 mg, 0.120 mmol, 48% yield).

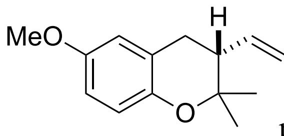

Rf 0.73 (cyclohexane/EtOAc, 8:2, *v/v*). **^{1}H NMR** (400 MHz, $CDCl_3$) δ 6.71 (s, 1H), 6.71 – 6.66 (m, 1H), 6.62 – 6.58 (m, 1H), 5.74 (ddd, *J* = 17.1, 10.3, 8.5 Hz, 1H), 5.18 (ddd, *J* = 17.1, 1.8, 1.0 Hz, 1H), 5.13 (ddd, *J* = 10.3, 1.8, 0.7 Hz, 1H), 3.75 (s, 3H), 2.78 (dd, *J* = 16.8, 5.9 Hz, 1H), 2.68 (ddt, *J* = 16.8, 9.8, 0.9 Hz, 1H), 2.49 – 2.40 (m, 1H), 1.36 (s, 3H), 1.17 (s, 3H). **^{13}C NMR** (101

MHz, $CDCl_3$) δ 153.2, 147.5, 138.4, 121.4, 117.8, 117.0, 113.9, 113.6, 55.8, 46.1, 29.2, 27.7, 21.4. **HRMS** (ESI –) calculated for [$C_{14}H_{19}O_2$] $[M+H]^+$ 219.1380 *m/z*; found 219.1377 *m/z*.

3-Vinyl-*spiro*[chromane-2,1'-cyclohexane] (2v)

Prepared following the general procedure **GP3** using 2-(2-cyclohexylideneethyl)phenol **1v** (50.6 mg, 0.250 mmol, 1.0 equiv), JohnPhosAuCl (8.0 mg, 15 μmol, 0.06 equiv), $NaBAr^F_4$ (13.3 mg, 15.0 μmol, 0.06 equiv) and 1 bar of acetylene gas in $CHCl_3$ (0.63 mL, 0.4 M) at 23 ºC for 16 h. The crude product was purified by flash column chromatography (SiO_2, cyclohexane/EtOAc 100:0 to 95:5, *v/v*) and the product **2v** was obtained as a colorless oil (21.0 mg, 0.092 mmol, 37% yield).

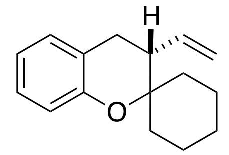

Rf 0.84 (cyclohexane/EtOAc, 8:2, *v/v*). **^{1}H NMR** (500 MHz, $CDCl_3$) δ 7.13–7.08 (m, 2H), 7.04 (dd, *J* = 7.5, 1.5 Hz, 1H), 6.88–6.81 (m, 1H), 5.76 (ddd, *J* = 17.1, 10.3, 9.0 Hz, 1H), 5.14 (ddd, *J* = 17.1, 1.9, 0.9 Hz, 1H), 5.10 (ddd, *J* = 10.2, 1.9, 0.5 Hz, 1H), 2.85 (dd, *J* = 16.8, 5.9 Hz, 1H), 2.69 (dd, *J* = 16.8, 8.4 Hz, 1H), 2.44 (td, *J* = 8.7, 5.8 Hz, 1H), 1.84–1.41 (m, 6H), 1.34–1.18 (m, 4H). **^{13}C NMR** (126 MHz, $CDCl_3$) δ 153.3, 138.5, 129.5, 127.4, 121.1, 120.0, 117.3, 116.8, 45.6, 34.9, 30.2, 28.5, 26.0, 21.6, 21.4. **HRMS** (ESI +) calculated for [$C_{16}H_{21}O$] $[M+H]^+$ 229.1587 *m/z*; found 229.1588 *m/z*.

4-Methyl-2-phenyl-3-vinylchromane (2w)

Prepared following the general procedure **GP3** using 2-(4-phenylbut-3-en-2-yl)phenol **1w** (56.1 mg, 0.250 mmol, 1.0 equiv), JohnPhosAuCl (8.0 mg, 15 μmol, 0.06 equiv), $NaBAr^F_4$ (13.3 mg, 15.0 μmol, 0.06 equiv) and 1 bar of acetylene gas in $CHCl_3$ (0.63 mL, 0.4 M) at 23 ºC for 16 h. The crude product was purified by flash column chromatography (SiO_2, cyclohexane/EtOAc 100:0 to 95:5, *v/v*) and the product **2w** was obtained as a yellow oil (46.0 mg, 0.180 mmol, 74% yield) in a 1:1 mixture of isomers.

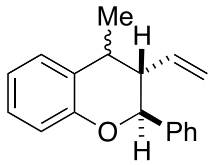

Rf 0.81 (cyclohexane/EtOAc, 8:2, *v/v*). **^{1}H NMR** (500 MHz, $CDCl_3$) δ 7.42–7.29 (m, 10H), 7.16 (ddddd, *J* = 9.8, 7.2, 5.4, 1.7, 0.7 Hz, 4H), 7.01–6.88 (m, 4H), 5.69 (ddd, *J* = 17.1, 10.3, 9.2 Hz, 1H), 5.44 (ddd, *J* = 17.1, 10.4, 9.1 Hz, 1H), 5.18 (d, *J* = 7.8 Hz, 1H), 5.09–4.97 (m, 3H), 4.83 (ddd, *J* = 17.1, 1.7, 0.7 Hz, 1H), 4.77 (d, *J* = 10.2 Hz, 1H), 3.01–2.91 (m, 2H), 2.90–2.83 (m, 2H), 1.37 (d, *J* = 6.8 Hz, 3H), 1.33 (d, *J* = 7.1 Hz, 3H). **^{13}C NMR** (126 MHz, $CDCl_3$) δ 154.7, 153.8, 140.9, 140.2, 137.1, 136.2, 128.5, 128.5, 128.4, 128.3, 128.0, 127.9, 127.7, 127.7, 127.4, 127.3, 126.9, 126.8, 121.0, 120.5, 118.6, 117.9, 117.0, 116.6, 81.8, 78.1, 52.1, 47.9, 34.8, 32.8, 18.7, 18.3. **HRMS** (ESI +) calculated for [$C_{18}H_{19}O$] $[M+H]^+$ 250.1352 *m/z*; found 250.1349 *m/z*.

4.3 Functionalization of Lapachol (3)

2,2-Dimethyl-3-vinyl-3,4-dihydro-2*H*-benzo[*g*]chromene-5,10-dione-2-hydroxy-3-(3-methylbut-2-en-1-yl) naphthalene-1,4-dione-ethyne (4)

A test tube was charged with Lapachol (**3**) (60.6 mg, 0.250 mmol, 1.0 equiv), JohnPhosAuCl (8.0 mg, 15 μmol, 0.06 equiv) and $NaBAr^F_4$ (13.3 mg, 15 μmol, 0.06 equiv) in HPLC grade $CHCl_3$ (0.63 mL, 0.4 M). The tube was introduced in a HEL reactor that, after proper closure, was pressurized with 1.0 bar of acetylene gas. The reaction mixture was stirred at 23 ºC for 16 h and after emptying the remaining gas, the crude was quenched by the addition of 3 drops of NEt_3 and concentrated under reduced pressure. The crude product was purified by flash column chromatography (SiO_2, cyclohexane/EtOAc 100:0 to 80:20, *v/v*) and the product **4** was obtained as a orange sticky solid (33.0 mg, 0.120 mmol, 50% yield).

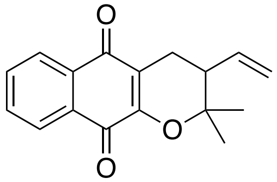

Rf 0.32 (cyclohexane/EtOAc, 8:2, *v/v*). **^{1}H NMR** (400 MHz, $CDCl_3$) δ 8.06 (ddd, *J* = 7.6, 1.4, 0.6 Hz, 1H), 7.80 (ddd, *J* = 7.8, 1.3, 0.5 Hz, 1H), 7.64 (td, *J* = 7.7, 1.4 Hz, 1H), 7.52 (dd, *J* = 7.6, 1.3 Hz, 1H), 5.73 (ddd, *J* = 17.1, 10.3, 7.9 Hz, 1H), 5.25 (ddd, *J* = 17.1, 1.5, 1.0 Hz, 1H), 5.20 (ddd, *J* = 10.3, 1.5, 0.7 Hz, 1H), 2.79–2.69 (m, 1H), 2.49–2.42 (m, 1H), 2.37 (dd, *J* = 16.9, 10.2 Hz, 1H), 1.56–1.50 (m, 3H), 1.29 (s, 3H). **^{13}C NMR** (101 MHz, $CDCl_3$) δ 179.9, 178.6, 161.7, 136.4, 135.0, 132.5, 130.9, 130.3, 128.8, 124.3, 118.5, 112.6, 81.8, 45.0, 27.4, 22.3, 21.6. **HRMS** (ESI +) calculated for $[C_{17}H_{17}O_3]$ $[M+H]^+$ 269.1172 *m/z*; found 269.1182 *m/z*.

4.4 Suzuki coupling of product 2d

2,6-Diphenyl-3-vinylchromane (2x)

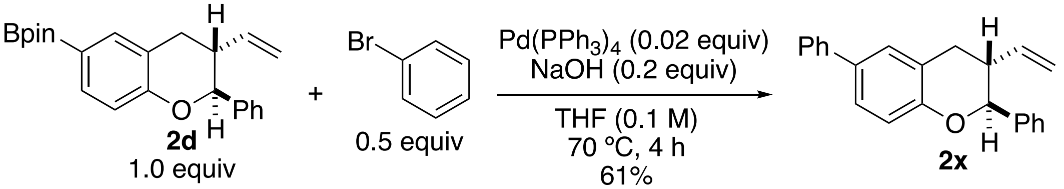

A flame and dried MW vial was charged inside the glovebox with $Pd(PPh_3)_4$ (2.7 mg, 0.002 mmol, 0.02 equiv) in dry THF (1.2 mL, 0.1 M) and then, bromobenzene (6.10 µL, 0.06 mmol, 0.5 equiv), 4,4,5,5-tetramethyl-2-(2-phenyl-3-vinylchroman-6-yl)-1,3,2-dioxaborolane **2d** (42.0 mg, 0.12 mmol, 1.0 equiv) and 1 M NaOH (0.23 mL, 0.02 mmol, 0.2 equiv) were sequentially added. The mixture was stirred for 4 h at 70 ºC, cooled down to room temperature, quenched with water and extracted with Et_2O (3 x 6 mL). The combined organic layers were washed with brine (10 mL), dried over $MgSO_4$, and concentrated in vacuo. The crude was purified by flash column chromatography (SiO_2, cyclohexane/EtOAc 100:0 to 90:10, *v/v*) to afford the desired product **2x** as a white solid (22.0 mg, 0.07 mmol, 61% yield).

Rf 0.84 (cyclohexane/EtOAc, 8:2, *v/v*). **M.p.** 120–127 ºC. **^{1}H NMR** (500 MHz, $CDCl_3$) δ 7.59–7.54 (m, 2H), 7.45–7.41 (m, 2H), 7.40–7.29 (m, 8H), 6.98 (d, *J* = 8.4 Hz, 1H), 5.62 (ddd, *J* = 17.5, 10.5, 7.2 Hz, 1H), 5.08–5.03 (m, 1H), 5.02–5.00 (m, 1H), 4.84 (d, *J* = 9.0 Hz, 1H), 3.00–2.94 (m, 2H), 2.91–2.82 (m, 1H). **^{13}C NMR** (126 MHz, $CDCl_3$) δ 154.5, 141.1, 140.0, 137.6, 133.8, 128.8, 128.6, 128.4, 128.2, 127.4, 126.9, 126.8, 126.4, 121.7, 117.1, 116.7, 82.4, 42.7, 31.1. **HRMS** (ESI +) calculated for $[C_{23}H_{20}NaO_3]$ $[M+Na]^+$ 335.1406 *m/z*; found 335.1595 *m/z*.

4. 5 Diversification of product 2a

1-(2-Phenylchroman-3-yl)ethan-1-one (5)

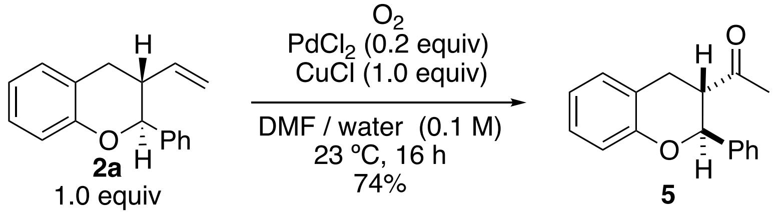

$PdCl_2$ (3.8 mg, 0.021 mmol, 0.2 equiv) and CuCl (10.5 mg, 0.106 mmol, 1.0 equiv) were dissolved in a 7:1 mixture of DMF (0.67 mL) and water (0.10 mL) and a balloon of O_2 was placed through one hour (a homogeneous green solution was formed). Then, 2-phenyl-3-vinylchromane **2a** (25.0 mg, 0.106 mmol, 1.0 equiv) was dissolved in DMF (0.42 mL, 0.1 M) and added to the previously prepared mixture under O_2 atmosphere. The reaction mixture was stirred at room temperature for

16 h and quenched by the addition of an aqueous HCl 1 M solution (5 mL) and extracted three times with Et_2O (3 x 5 mL). The combined organic layers were washed with brine (10 mL), dried over $MgSO_4$, filtered, and concentrated under reduced pressure. The crude product was purified by flash column chromatography (SiO_2, cyclohexane/EtOAc 100:0 to 60:40, *v/v*) to afford the product **5** as a white solid (11.0 mg, 0.044 mmol, 74% yield).

R$_f$ 0.56 (cyclohexane/EtOAc, 8:2, *v/v*). **M.p.** 71–76 ºC. **^{1}H NMR** (400 MHz, $CDCl_3$) δ 7.43–7.31 (m, 5H), 7.19–7.10 (m, 2H), 6.96–6.88 (m, 2H), 4.97 (dd, *J* = 7.8, 1.6 Hz, 1H), 3.24 (dt, *J* = 9.7, 1.4 Hz, 2H), 2.93–2.81 (m, 1H), 1.79 (s, 3H). **^{13}C NMR** (101 MHz, $CDCl_3$) δ 209.4, 154.4, 139.1, 129.5, 128.9, 127.8, 127.1, 121.0, 120.7, 116.9, 79.4, 52.3, 31.6, 29.9, 28.7. **HRMS** (ESI +) calculated for [$C_{17}H_{16}NaO_2$] $[M+Na]^+$ 270.1043 *m/z*; found 270.1048 *m/z*.

3-(Oxiran-2-yl)-2-phenylchromane (6)

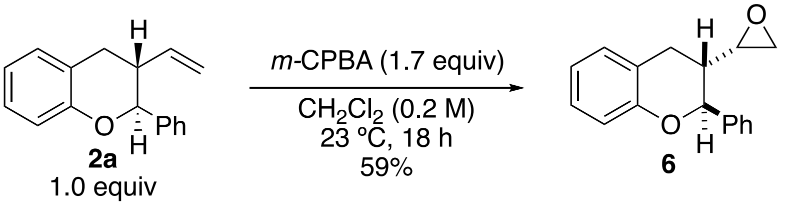

m-CPBA (37.2 mg, 0.216 mmol, 1.7 equiv) was slowly added, under argon atmosphere, to a solution of 2-phenyl-3-vinylchromane **2a** (30.0 mg, 0.127 mmol, 1.0 equiv) in dry CH_2Cl_2 (0.65 mL, 0.2 M) at 0 ºC. The reaction was warmed to room temperature and stirred for 18 h. The crude mixture was quenched with a saturated $NaHSO_3$ aqueous solution (5 mL) and extracted three times with CH_2Cl_2 (3 x 5 mL). The combined organic layers were washed with brine (10 mL), dried over $MgSO_4$, filtered, and concentrated under reduced pressure. The crude product was purified by flash column chromatography (SiO_2, cyclohexane/EtOAc 100:0 to 60:40, *v/v*) to afford the product **6** as a colorless oil (19.0 mg, 0.127 mmol, 59% yield).

R$_f$ 0.56 (cyclohexane/EtOAc, 8:2, *v/v*). **^{1}H NMR** (400 MHz, $CDCl_3$) δ 7.4–7.32 (m, 5H), 7.19–7.10 (m, 2H), 6.94–6.88 (m, 2H), 4.95 (d, *J* = 9.0 Hz, 1H), 2.89 (d, *J* = 7.8 Hz, 2H), 2.74 (ddd, *J* = 6.5, 4.0, 2.7 Hz, 1H), 2.52 (dd, *J* = 4.7, 4.0 Hz, 1H), 2.31 (dd, *J* = 4.7, 2.7 Hz, 1H), 2.06 (dddd, *J* = 9.1, 8.2, 7.4, 6.4 Hz, 1H). **^{13}C NMR** (126 MHz, $CDCl_3$) δ 154.6, 139.8, 129.8, 128.8, 128.6, 127.7, 126.9, 120.9, 120.8, 116.8, 79.9, 52.9, 46.4, 41.0, 27.2. **HRMS** (ESI +) calculated for [$C_{17}H_{17}O_2$] $[M+H]^+$ 253.1223 *m/z*; found 253.1224 *m/z*.

Methyl-(*E*)-3-(2-phenylchroman-3-yl)acrylate (7)

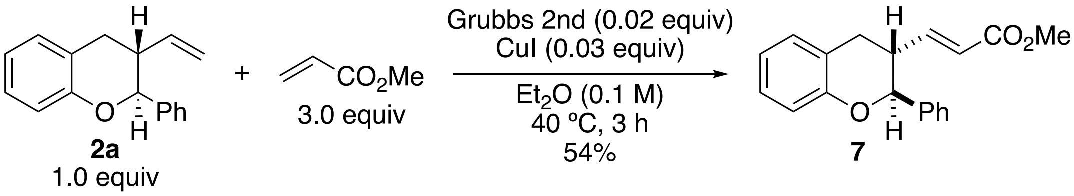

A dried MW vial was charged with 2-phenyl-3-vinylchromane **2a** (30.0 mg, 0.127 mmol, 1.0 equiv), methyl acrylate (34.3 µL, 0.381 mmol, 3.0 equiv), Grubbs 2nd generation catalyst (2.2 mg, 0.003 mmol, 0.02 equiv) and CuI (0.73 µg, 0.004 mmol, 0.03 equiv) under argon atmosphere. Dry Et_2O (1.3 mL, 0.1 M) was added, and the reaction was heated at 40 ºC for 3 h. After cooling down to room temperature the mixture was concentrated under reduced pressure and the residue was

purified by flash column chromatography (SiO_2, cyclohexane/EtOAc 100:0 to 70:30, *v/v*) to afford the product **7** as a white solid (20.0 mg, 0.070 mmol, 54% yield).

R_f 0.53 (cyclohexane/EtOAc, 8:2, *v/v*). **M.p.** 136–139 ºC. **^{1}H NMR** (500 MHz, $CDCl_3$) δ 7.41–7.30 (m, 5H), 7.19–7.13 (m, 1H), 7.12–7.09 (m, 1H), 6.95–6.88 (m, 2H), 6.78–6.71 (m, 1H), 5.76 (dd, *J* = 15.7, 1.0 Hz, 1H), 4.89 (d, *J* = 8.4 Hz, 1H), 3.67 (s, 3H), 3.03–2.95 (m, 2H), 2.95–2.88 (m, 1H). **^{13}C NMR** (126 MHz, $CDCl_3$) δ 166.5, 154.6, 147.4, 139.4, 129.5, 128.8, 128.6, 127.9, 127.1, 122.7, 120.9, 120.3, 116.9, 81.0, 51.7, 41.9, 30.7. **HRMS** (ESI +) calculated for [$C_{19}H_{18}NaO_3$] $[M+Na]^+$ 317.1148 *m/z*; found 317.1159 *m/z*.

4,4,5,5-Tetramethyl-2-(2-2-phenylchroman-3-yl)ethyl)-1,3,2-dioxaborolane (8)

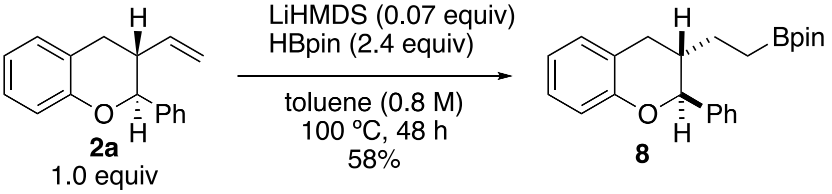

In an argon-purged Schlenk tube containing a magnetic stirring bar, dry toluene (0.10 mL), pinacolatoborane (58.9 µL, 0.406 mmol, 2.4 equiv) and LiHMDS (2.4 µL, 0.013 mmol, 0.07 equiv) were added sequentially at room temperature. The mixture was stirred for 5 min, 2-phenyl-3-vinylchromane **2a** (40.0 mg, 0.169 mmol, 1.0 equiv) was added dropwise in dry toluene (0.12 mL, 0.8 M) and the reaction was stirred at 100 ºC for 48 h. After completion of the reaction, the crude was allowed to cool down to room temperature and quenched by the addition of an aqueous HCl 1 M solution (1 mL) and extracted three times with EtOAc (3 x 1 mL). The combined organic layers were washed with brine (2 mL), dried over $MgSO_4$, filtered and concentrated under reduced pressure. The crude product was purified by flash column chromatography (SiO_2, cyclohexane/EtOAc 100:0 to 60:40, *v/v*) to afford the product **8** as a white solid (18.0 mg, 0.049 mmol, 58% yield).

R_f 0.68 (cyclohexane/EtOAc, 8:2, *v/v*). **M.p.** 66–70 ºC. **^{1}H NMR** (400 MHz, $CDCl_3$) δ 7.40–7.29 (m, 5H), 7.14–7.05 (m, 2H), 6.87 (d, *J* = 7.7 Hz, 2H), 4.77 (d, *J* = 8.3 Hz, 1H), 2.91 (dd, *J* = 16.3, 5.1 Hz, 1H), 2.57 (dd, *J* = 16.4, 9.6 Hz, 1H), 2.17–2.02 (m, 1H), 1.40 (ddt, *J* = 13.5, 8.2, 3.0 Hz, 1H), 1.31–1.21 (m, 1H), 1.21 (s, 12H), 0.95–0.77 (m, 1H), 0.77–0.66 (m, 1H). **^{13}C NMR** (126 MHz, $CDCl_3$) δ 154.9, 140.8, 129.7, 128.6, 128.1, 127.4, 127.3, 122.0, 120.4, 116.5, 83.2, 82.7, 39.3, 30.0, 26.2, 25.0, 24.9. **HRMS** (ESI +) calculated for [$C_{23}H_{29}NaBO_3$] $[M+Na]^+$ 387.2102 *m/z*; found 387.2108 *m/z*.

4.6 Synthesis of chiral catalyst (E)

Prepared using the following procedure: To a 10 ml round-bottomed flask was added DMSAuCl (124 mg, 0.420 mmol, 2.1 equiv), phosphine ligand (128 mg, 0.200 mmol, 1.0 equiv) and DCM (2.2 mL, 0.1 M). The resulting solution was stirred at room temperature for 1 hour. Following concentration *in vacuo*, the crude product was purified by flash column chromatography (SiO_2, cyclohexane/EtOAc, 4:1, *v/v*) and Au-complex **E** was obtained as an organge solid (198.0 mg, 0.179 mmol, 90% yield).

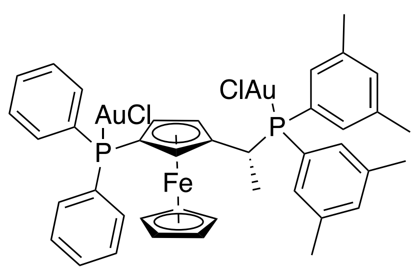

***R**f* 0.27 (cyclohexane/EtOAc, 4:1, *v/v*). **^{1}H NMR** (500 MHz, $CDCl_3$) δ 7.79–7.73 (m, 2H), 7.56–7.52 (m, 1H), 7.51–7.46 (m, 3H), 7.45 (s, 1H), 7.38–7.33 (m, 1H), 7.23–7.18 (m, 2H), 7.17–7.13 (m, 3H), 7.11–7.05 (m, 2H), 6.56 (s, 1H), 5.27–5.18 (m, 1H), 5.11–5.07 (m, 1H), 4.73 (dd, *J* = 2.7, 2.7 Hz, 1H), 4.27–4.25 (m, 1H), 3.98 (s, 5H), 2.39 (s, 6H), 2.07 (s, 6H), 1.77 (dd, *J* = 17.8, 7.1 Hz, 3H). **^{13}C NMR** (126 MHz, $CDCl_3$) δ 139.6 (d, $J_{C–P}$ = 12.0 Hz), 138.8 (d, $J_{C–P}$ = 12.1 Hz), 135.4 (d, $J_{C–P}$ = 15.0 Hz), 134.2 (d, $J_{C–P}$ = 2.7 Hz), 134.1 (d, $J_{C–P}$ = 2.7 Hz), 132.8 (d, $J_{C–P}$ = 13.4 Hz), 132.5 (d, $J_{C–P}$ = 68.2 Hz), 132.4 (d, $J_{C–P}$ = 2.6 Hz), 132.3 (d, $J_{C–P}$ = 13.3 Hz), 131.9 (d, $J_{C–P}$ = 13.3 Hz), 131.4 (d, $J_{C–P}$ = 63.0 Hz), 131.0 (d, $J_{C–P}$ = 2.7 Hz), 129.23 (d, $J_{C–P}$ = 12.1 Hz), 129.0 (d, $J_{C–P}$ = 11.8 Hz), 128.8 (d, $J_{C–P}$ = 55.2 Hz), 127.9 (d, $J_{C–P}$ = 54.9 Hz), 96.7 (dd, $J_{C–P}$ = 17.5, 7.8 Hz), 73.4 (dd, $J_{C–P}$ = 7.5, 7.5 Hz), 72.9 (d, $J_{C–P}$ = 5.1 Hz), 72.2 (d, $J_{C–P}$ = 7.7 Hz), 71.2, 66.7 (d, $J_{C–P}$ = 68.6 Hz), 31.7 (dd, $J_{C–P}$ = 32.2, 4.3 Hz), 22.8 (d, $J_{C–P}$ = 5.9 Hz), 21.5, 21.4. **^{31}P NMR** (202 MHz, CD_2Cl_2) δ 54.69, 22.80. **HRMS** (ESI +) calculated for [$C_{40}H_{40}Au_2ClP_2Fe$] $[M+H]^+$ 1067.0969 *m/z*; found 1067.0980 *m/z.*

5. NMR spectra

5.1 Starting materials

Compound 1d ^{1}H NMR (500 MHz, $CDCl_3$, 298 K)

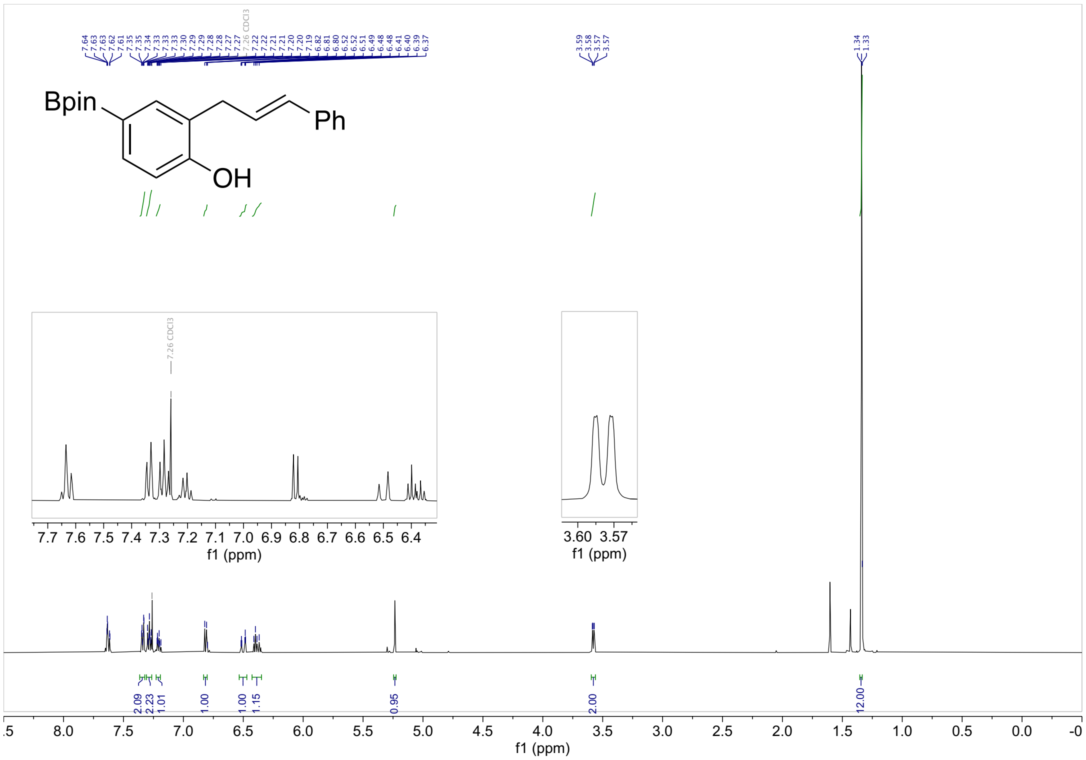

Compound 1d $^{13}C\{^1H\}$ NMR (126 MHz, $CDCl_3$, 298 K)

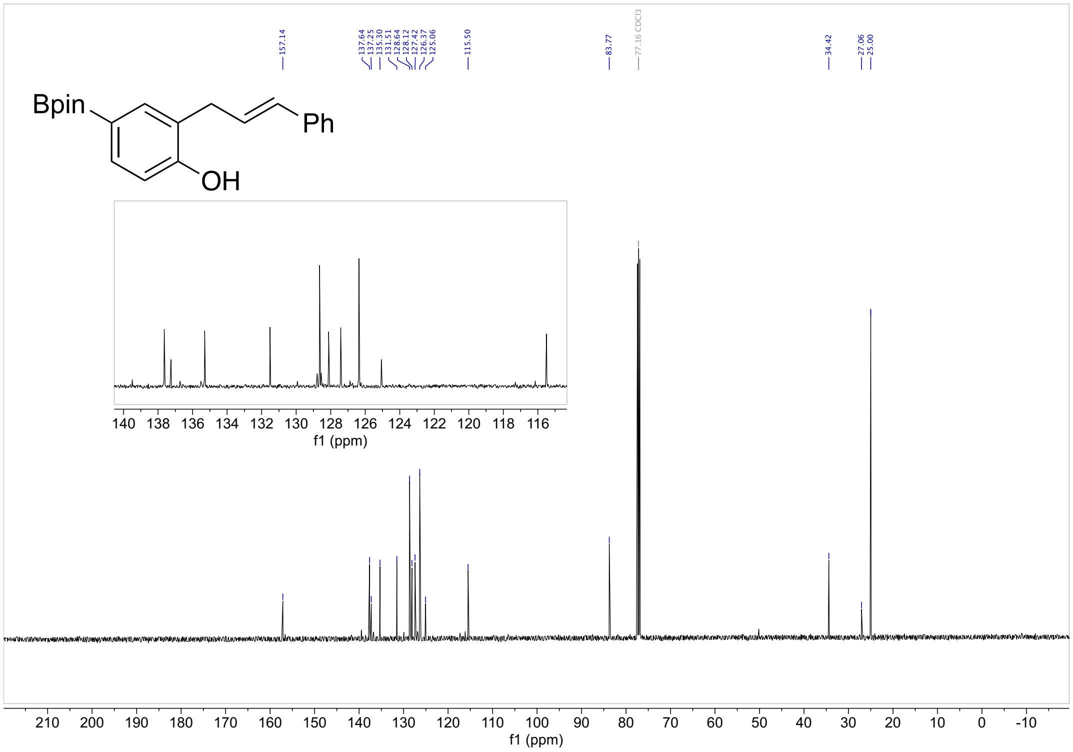

Compound 1f ^{1}H NMR (500 MHz, $CDCl_3$, 298 K)

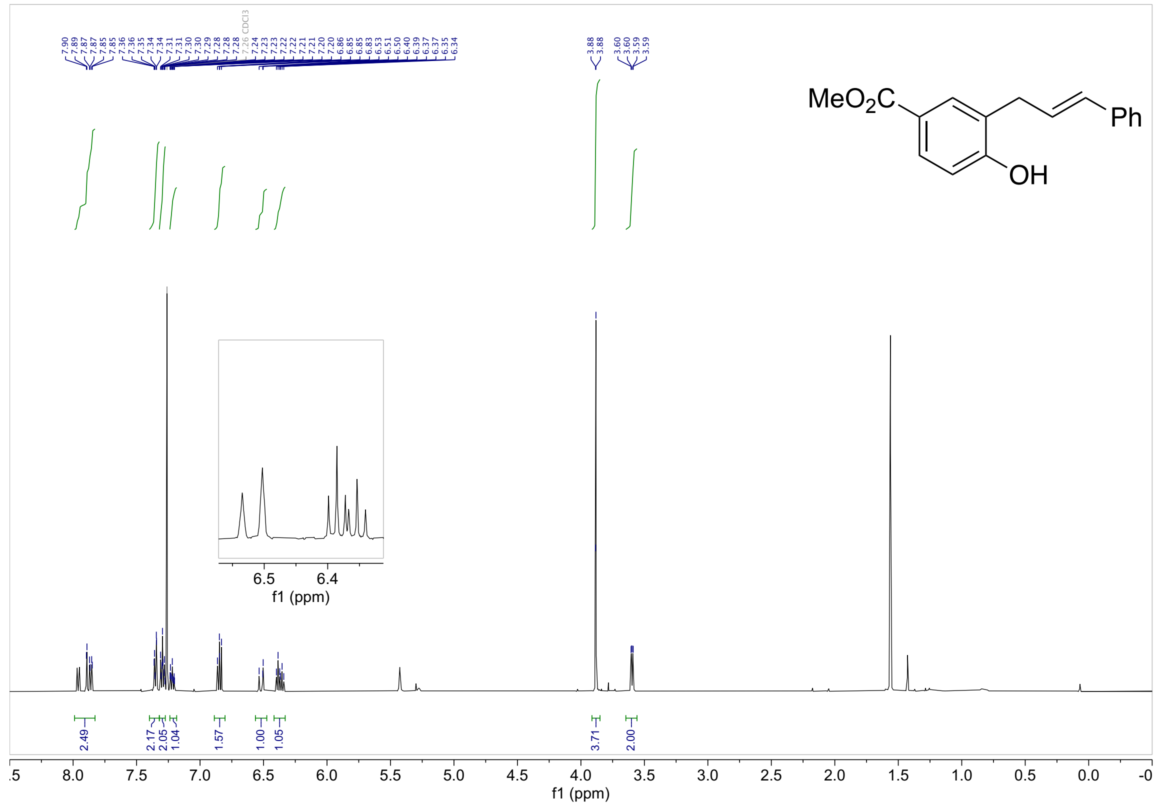

Compound 1f $^{13}C\{^{1}H\}$ NMR (126 MHz, $CDCl_3$, 298 K)

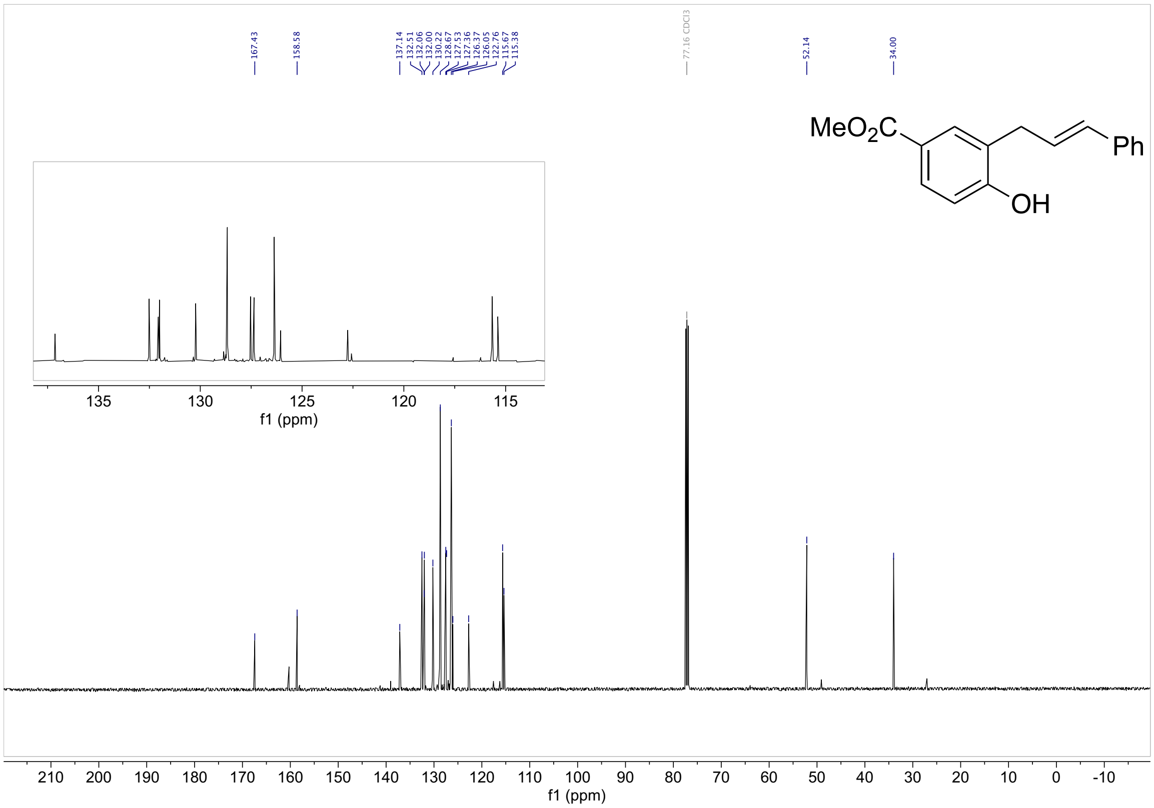

Compound 1l ^{1}H NMR (500 MHz, $CDCl_3$, 298 K)

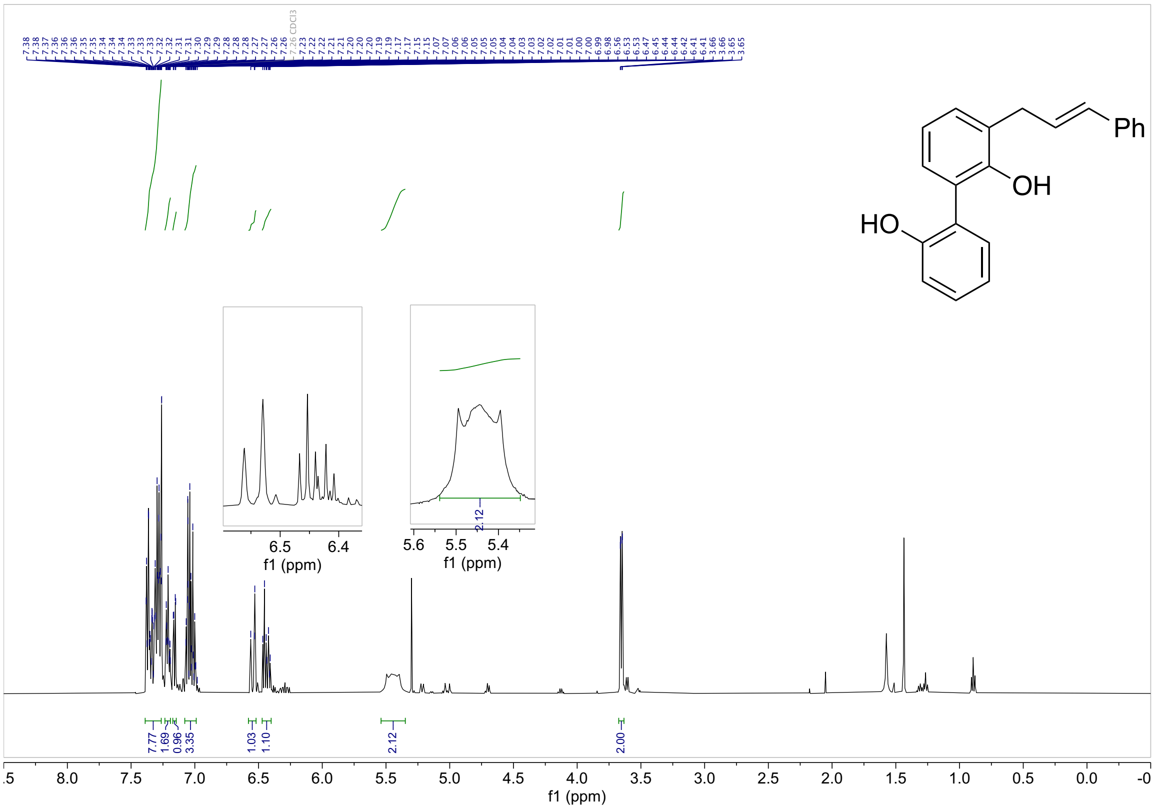

Compound 1l ^{13}C{^{1}H} NMR (126 MHz, $CDCl_3$, 298 K)

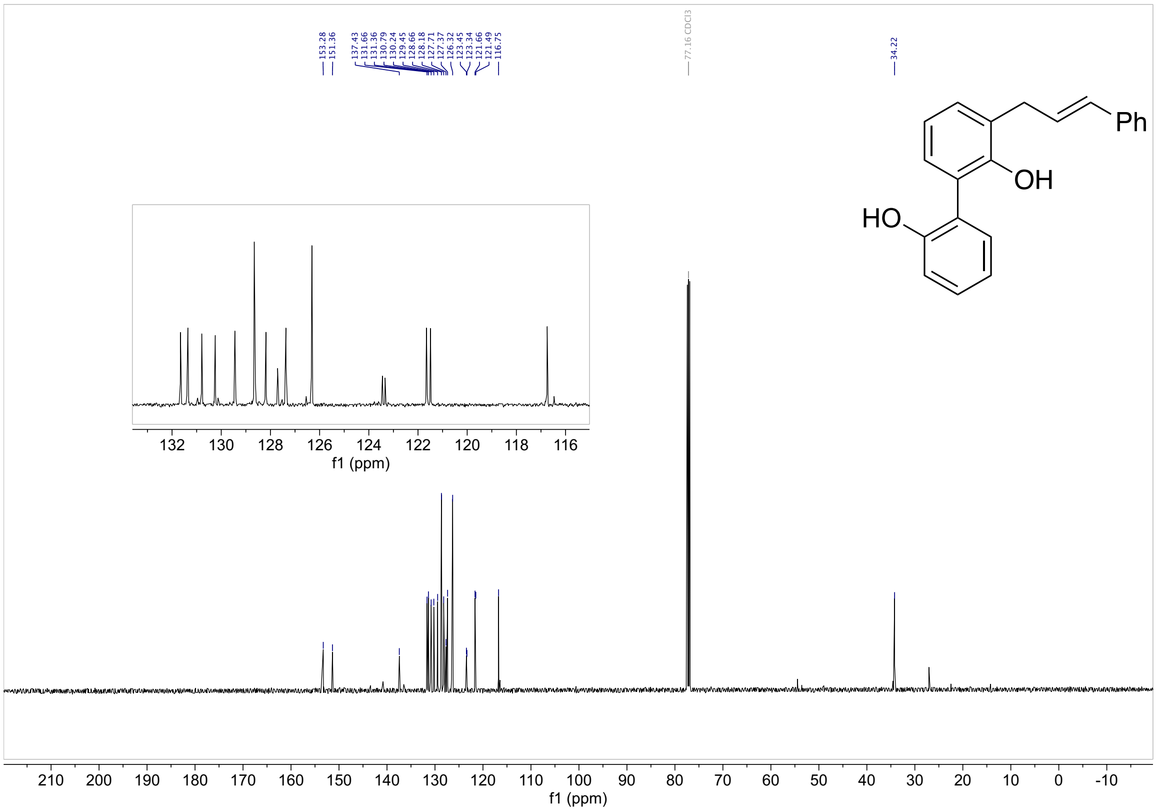

Compound 1m ^{1}H NMR (400 MHz, $CDCl_3$, 298 K)

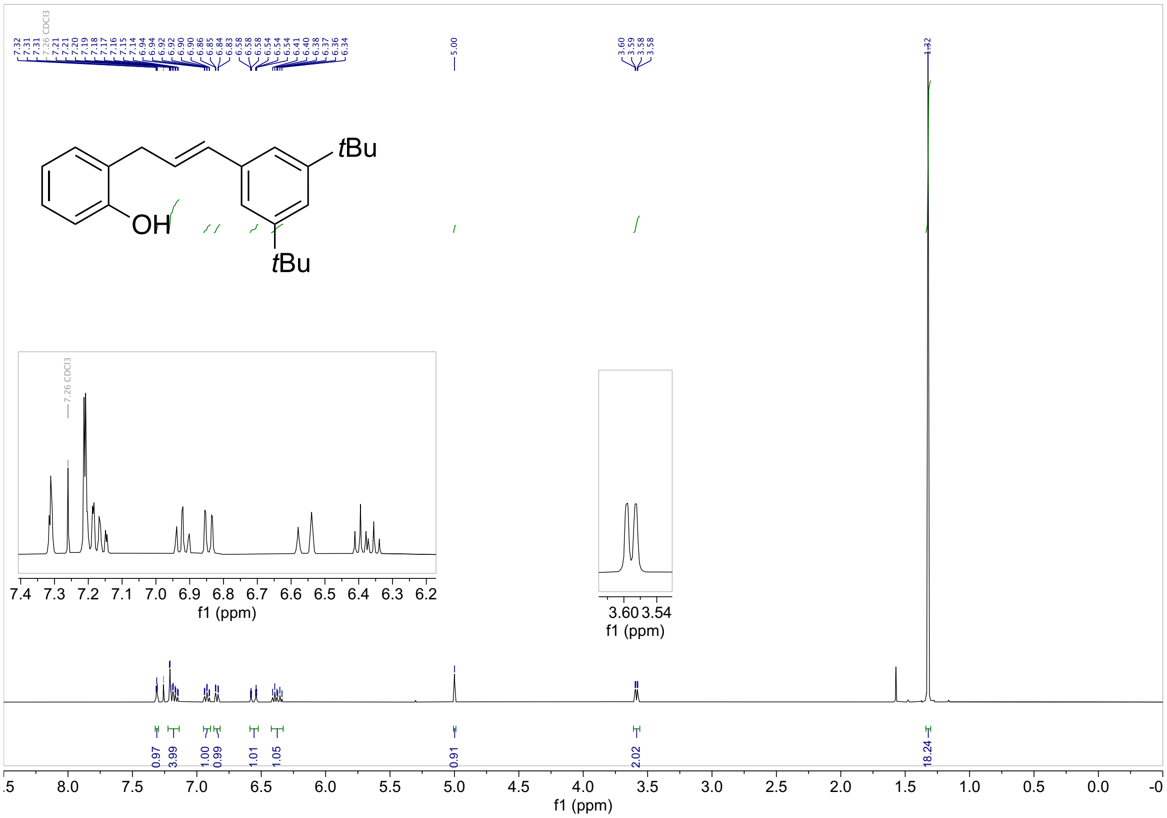

Compound 1m ^{13}C{1H} NMR (101 MHz, $CDCl_3$, 298 K)

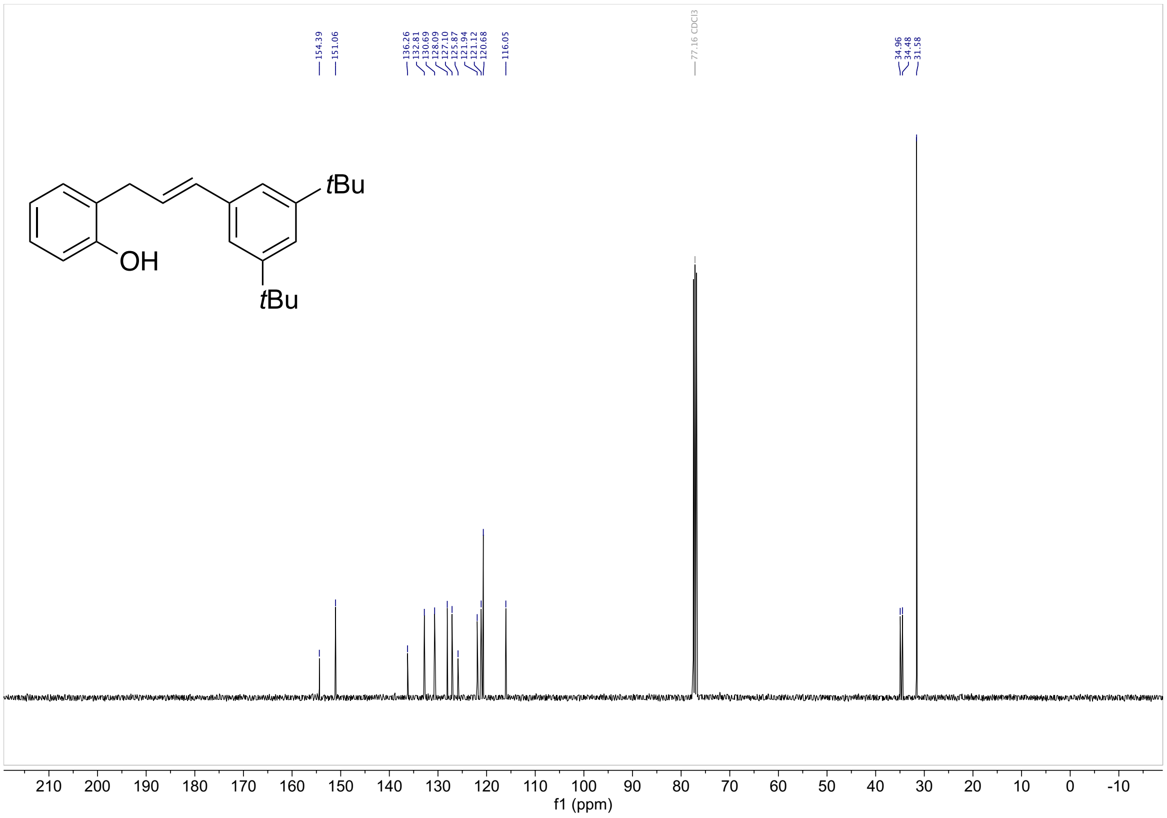

Compound 1n ^{1}H NMR (500 MHz, $CDCl_3$, 298 K)

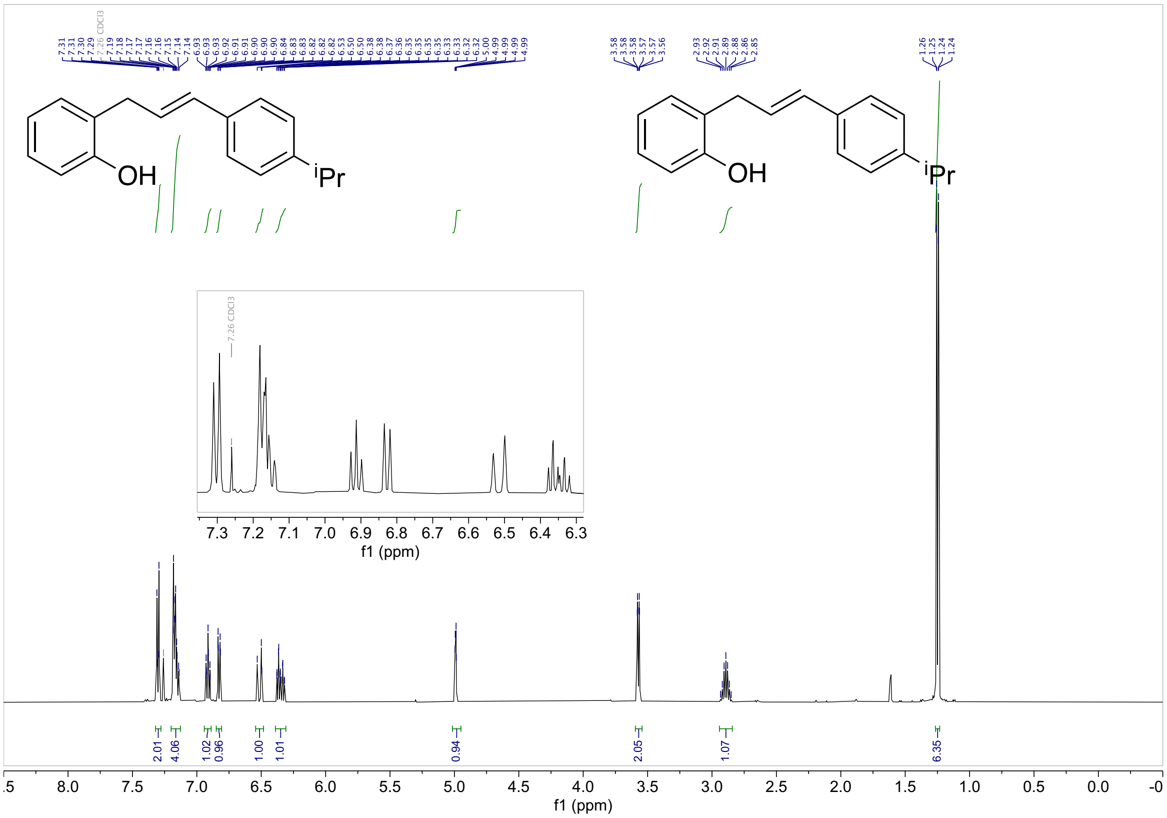

Compound 1n ^{13}C{1H} NMR (126 MHz, $CDCl_3$, 298 K)

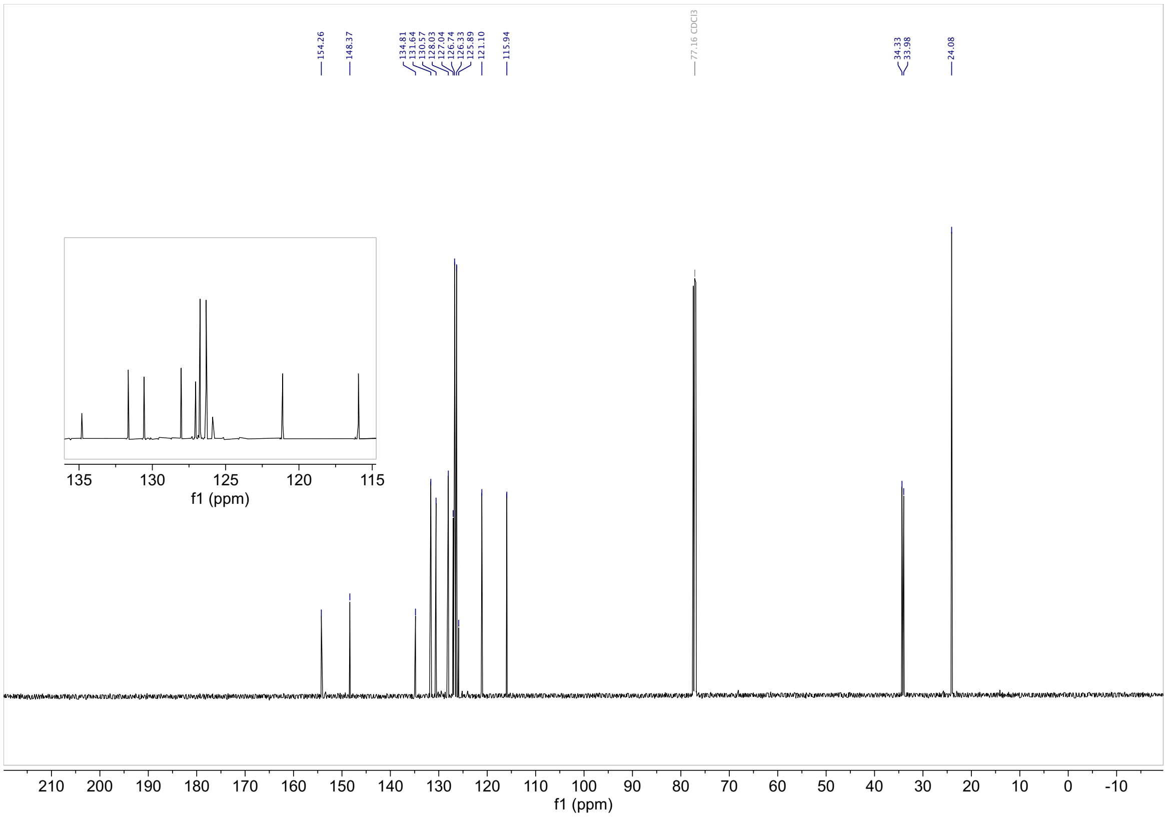

Compound 1o ^{1}H NMR (400 MHz, $CDCl_3$, 298 K)

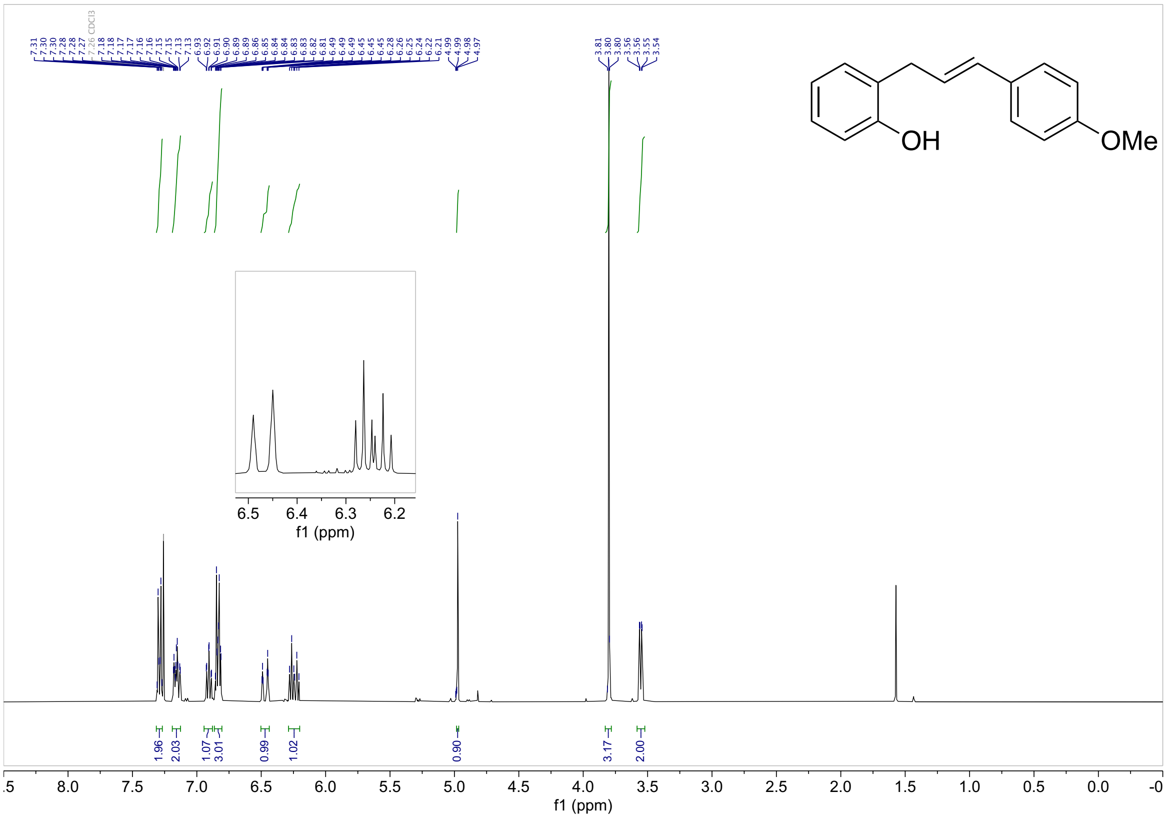

Compound 1o ^{13}C{1H} NMR (101 MHz, $CDCl_3$, 298 K)

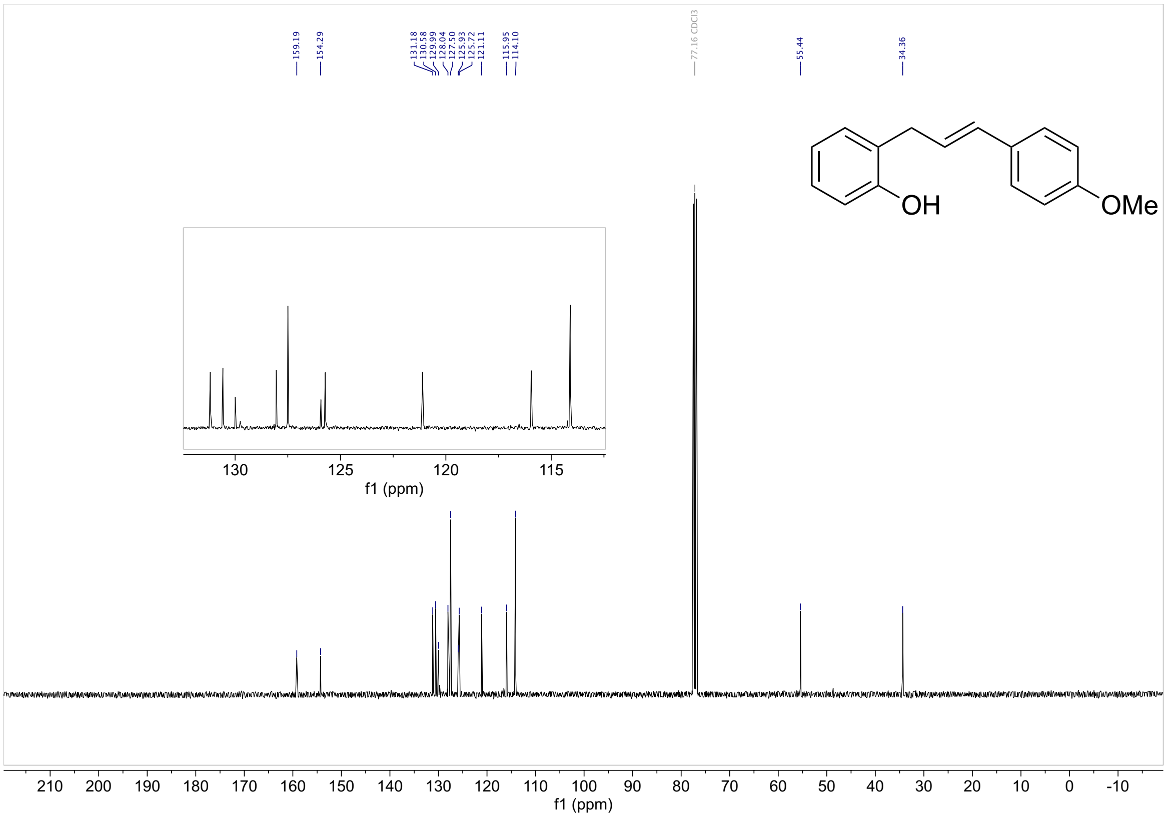

Compound 1q ^{1}H NMR (400 MHz, $CDCl_3$, 298 K)

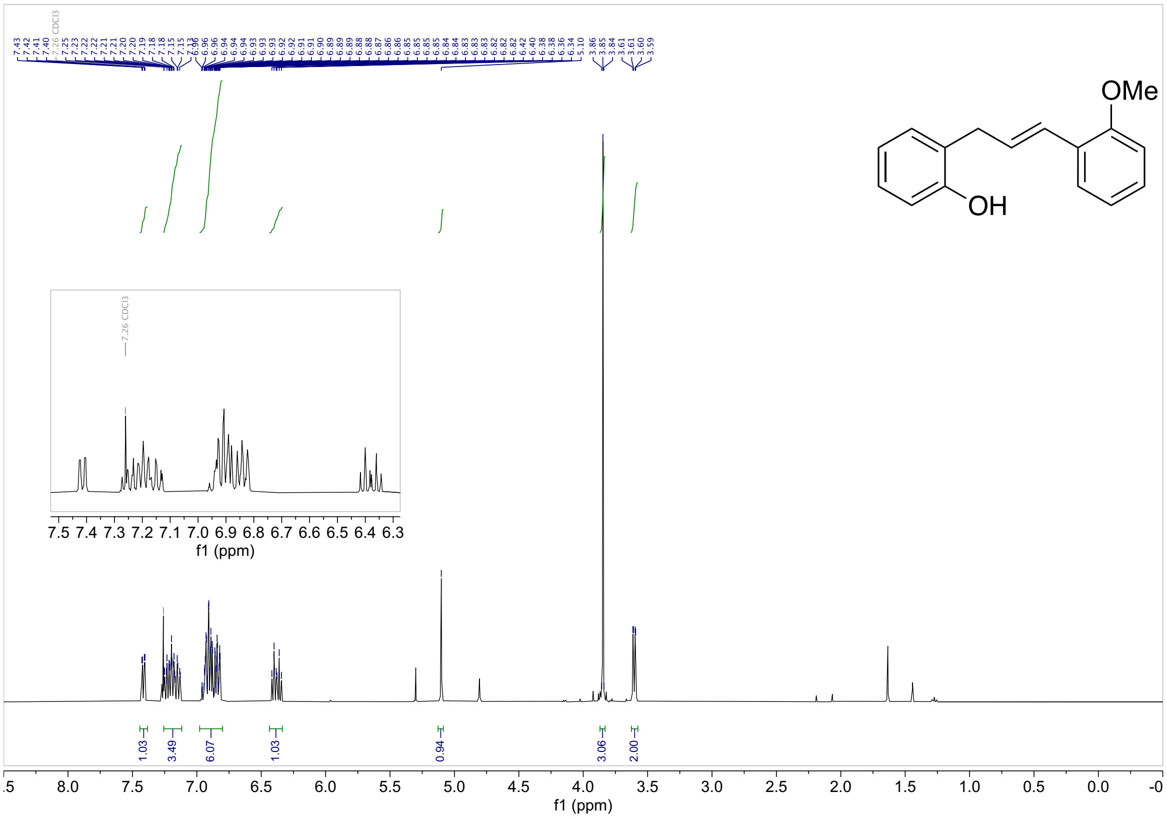

Compound 1q ^{13}C{1H} NMR (101 MHz, $CDCl_3$, 298 K)

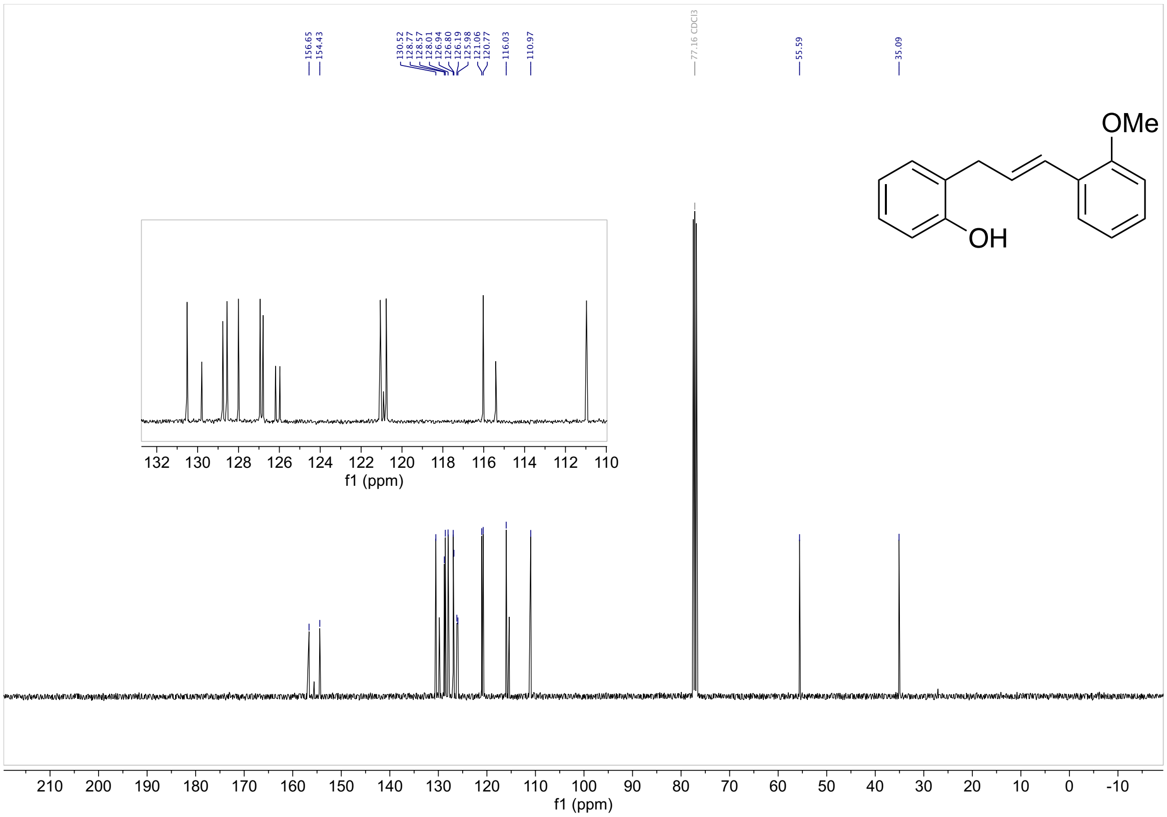

Compound 1r ^{1}H NMR (500 MHz, $CDCl_3$, 298 K)

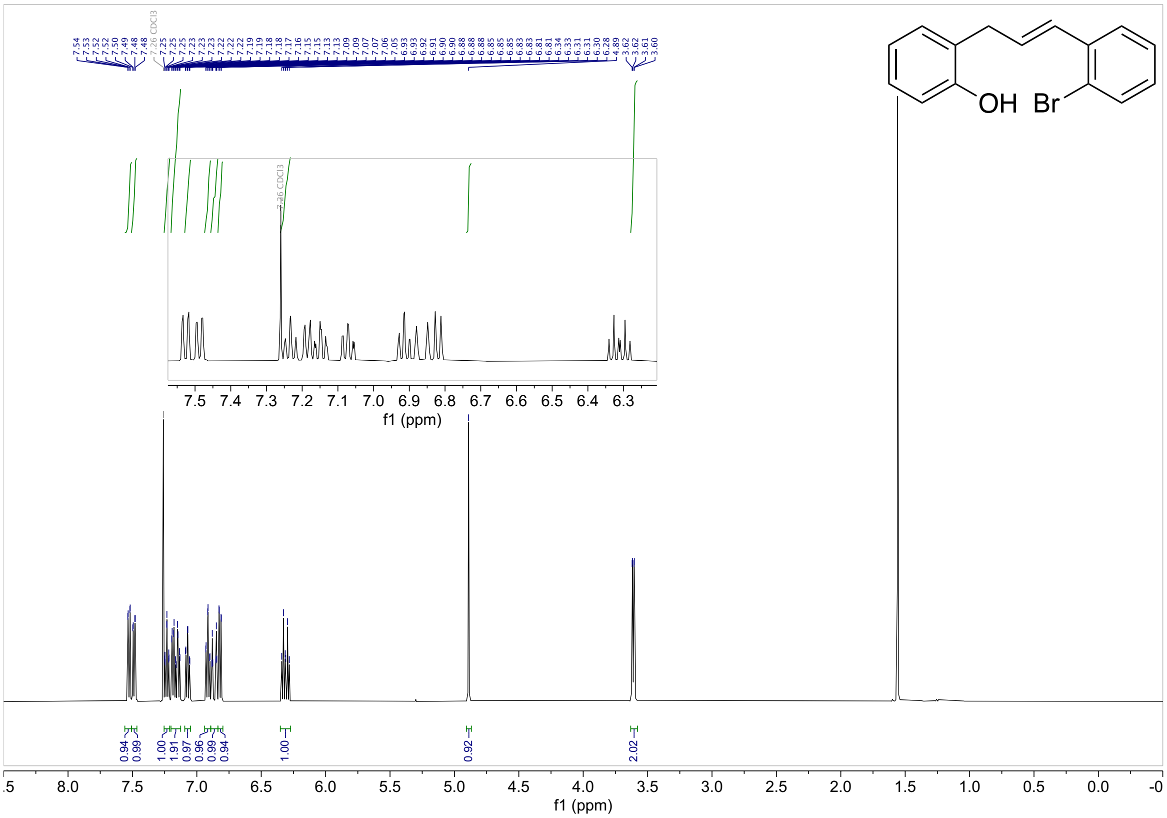

Compound 1r $^{13}C\{^{1}H\}$ NMR (126 MHz, $CDCl_3$, 298 K)

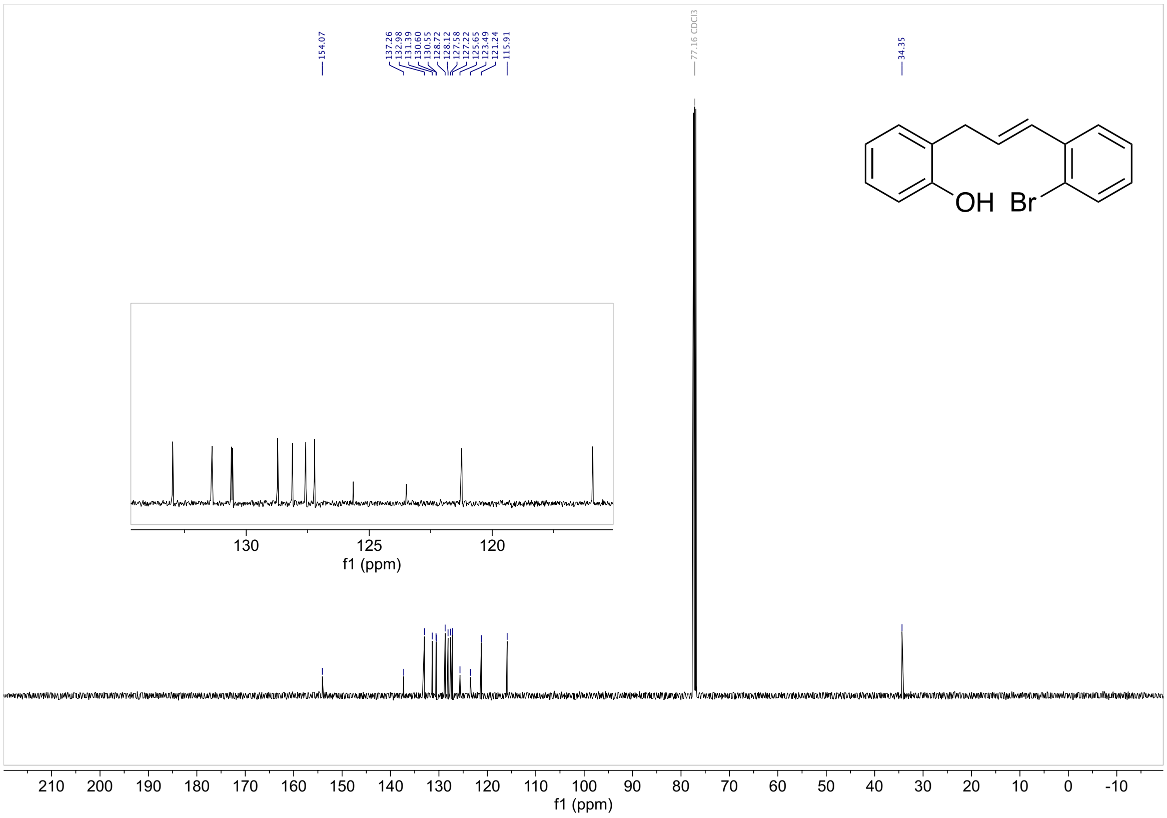

Compound 1s ^{1}H NMR (500 MHz, $CDCl_3$, 298 K)

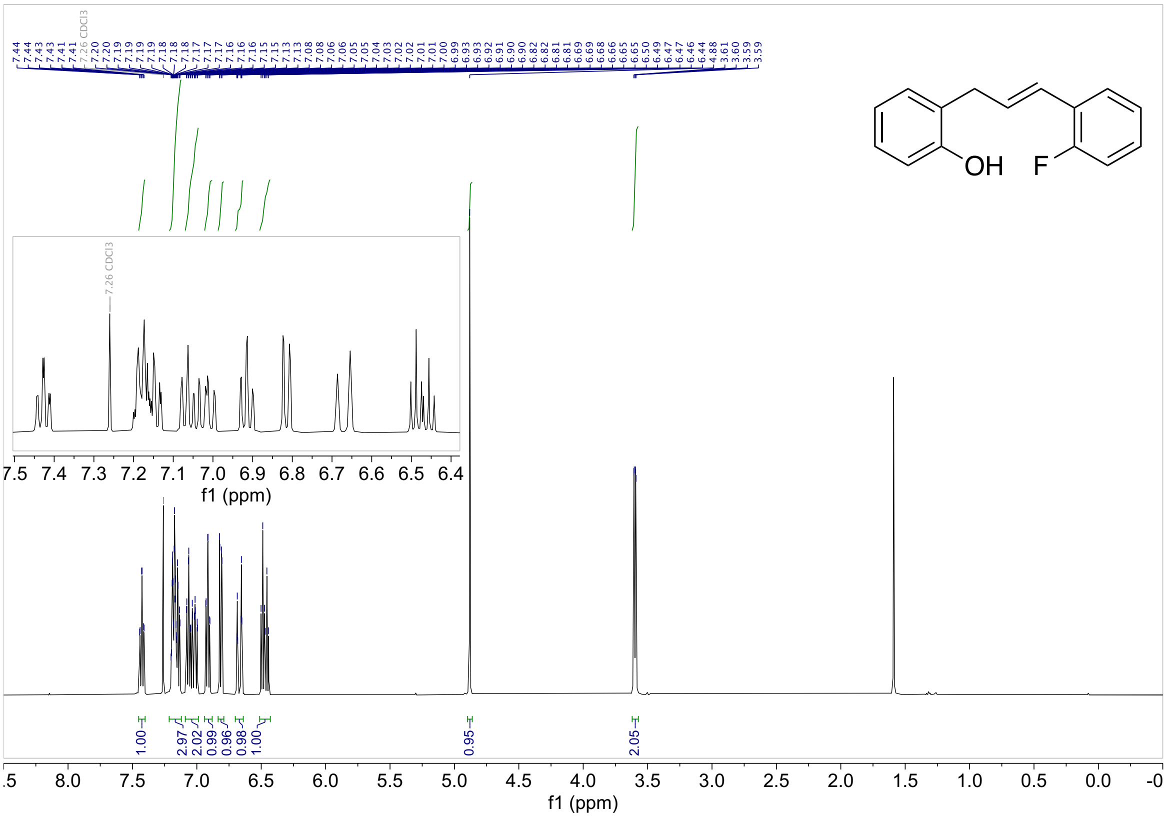

Compound 1s $^{13}C\{^{1}H\}$ NMR (126 MHz, $CDCl_3$, 298 K)

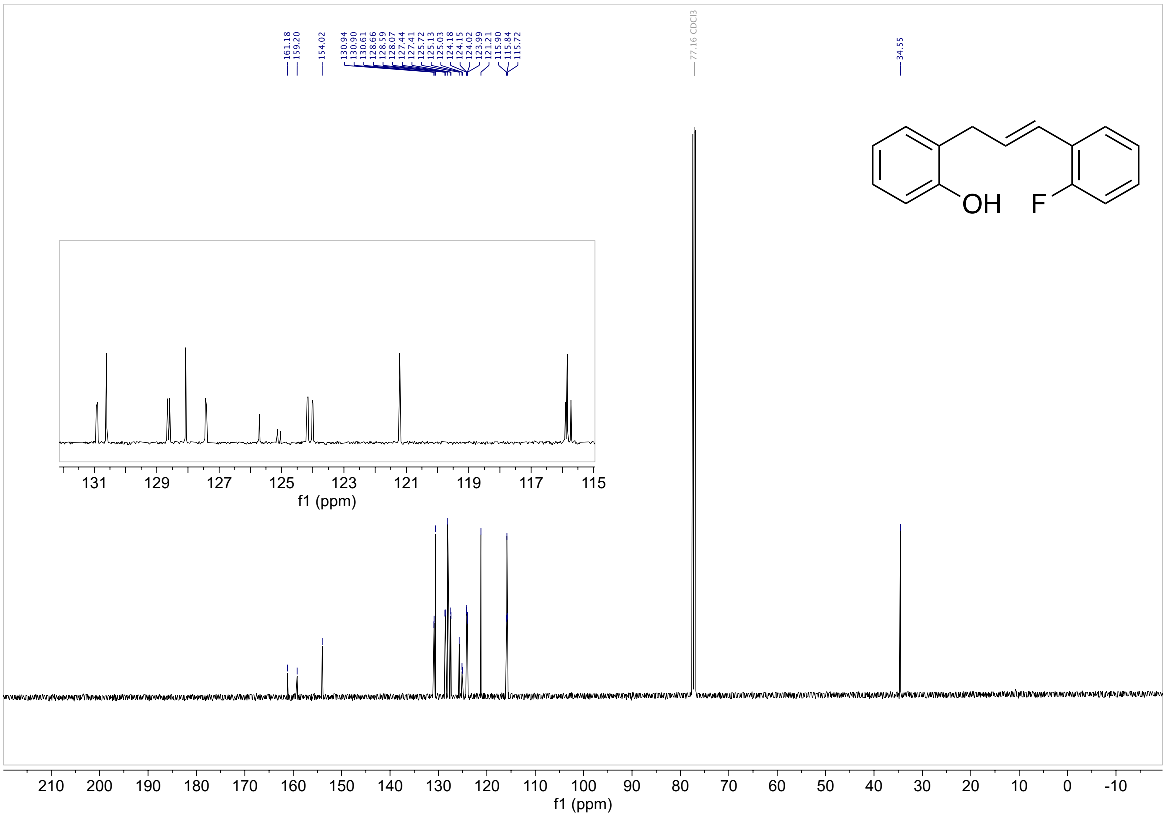

Compound 1s $^{19}F\{^1H\}$ NMR (126 MHz, $CDCl_3$, 298 K)

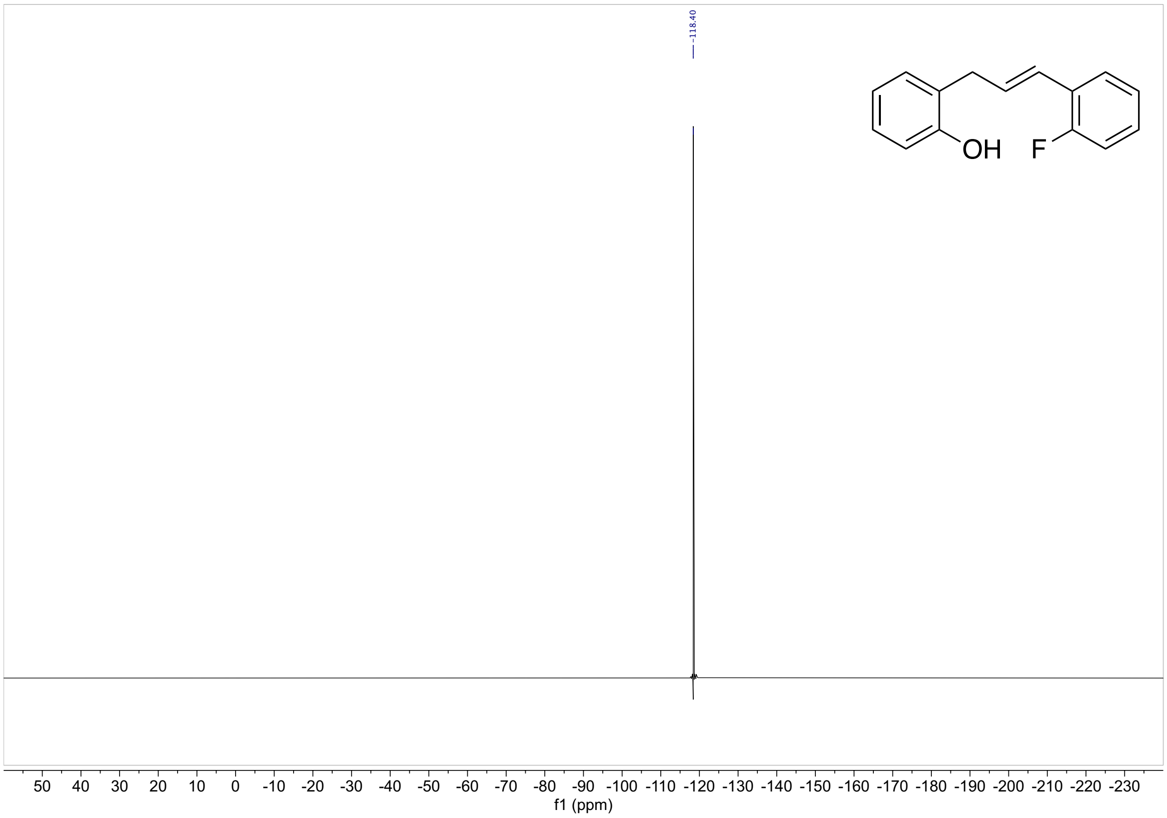

Compound 1t ^{1}H NMR (400 MHz, $CDCl_3$, 298 K)

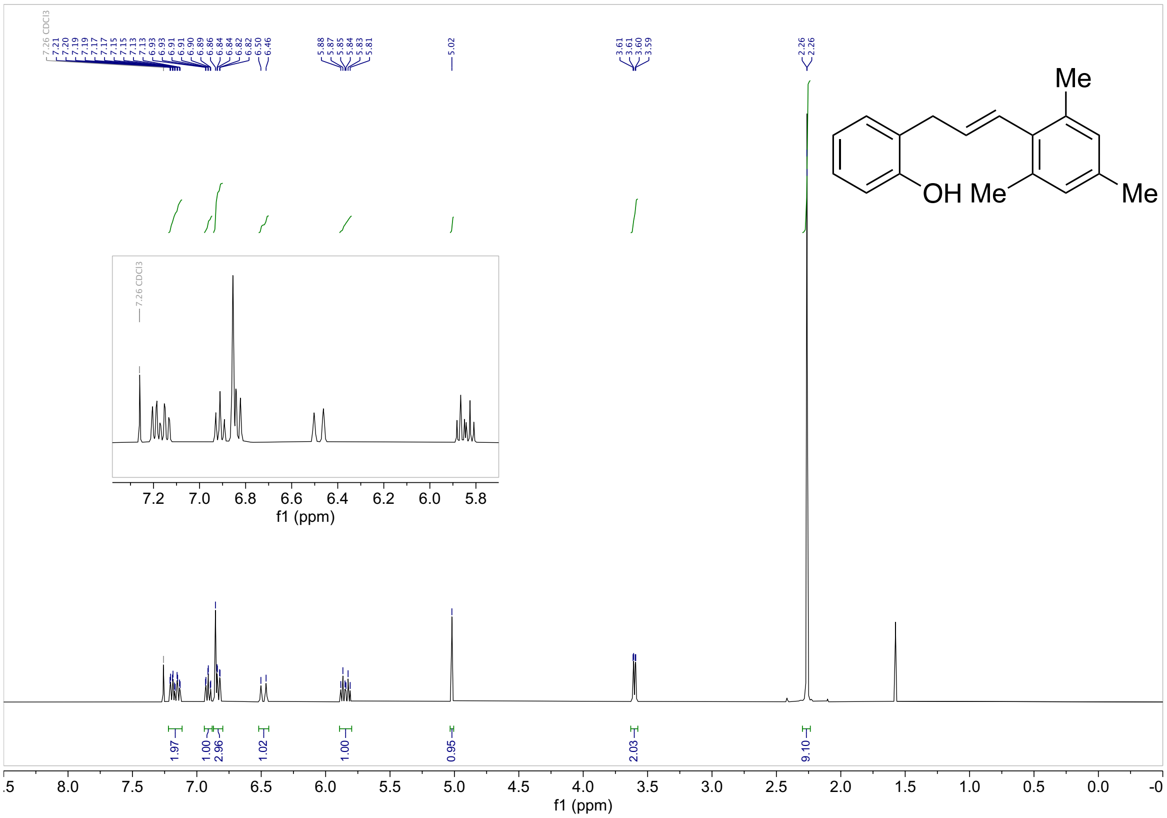

Compound 1t $^{13}C\{^1H\}$ NMR (101 MHz, $CDCl_3$, 298 K)

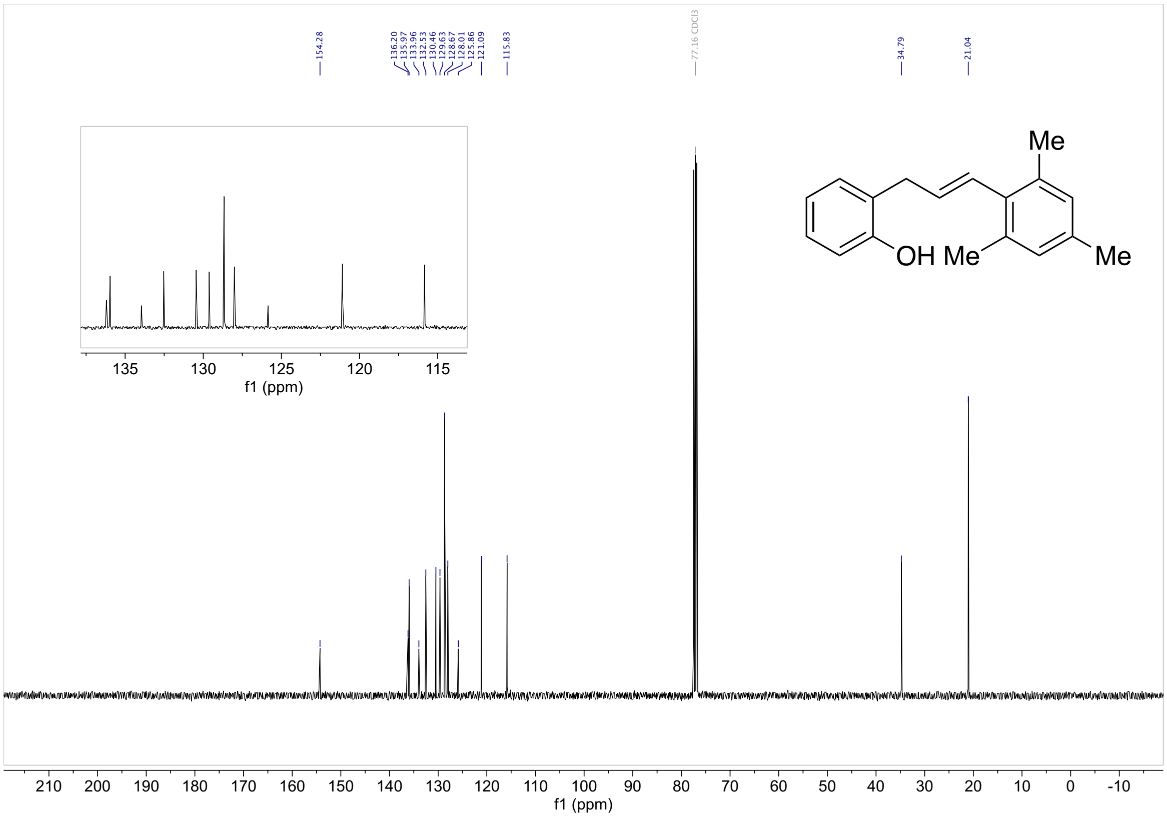

Compound 1v ^{1}H NMR (500 MHz, $CDCl_3$, 298 K)

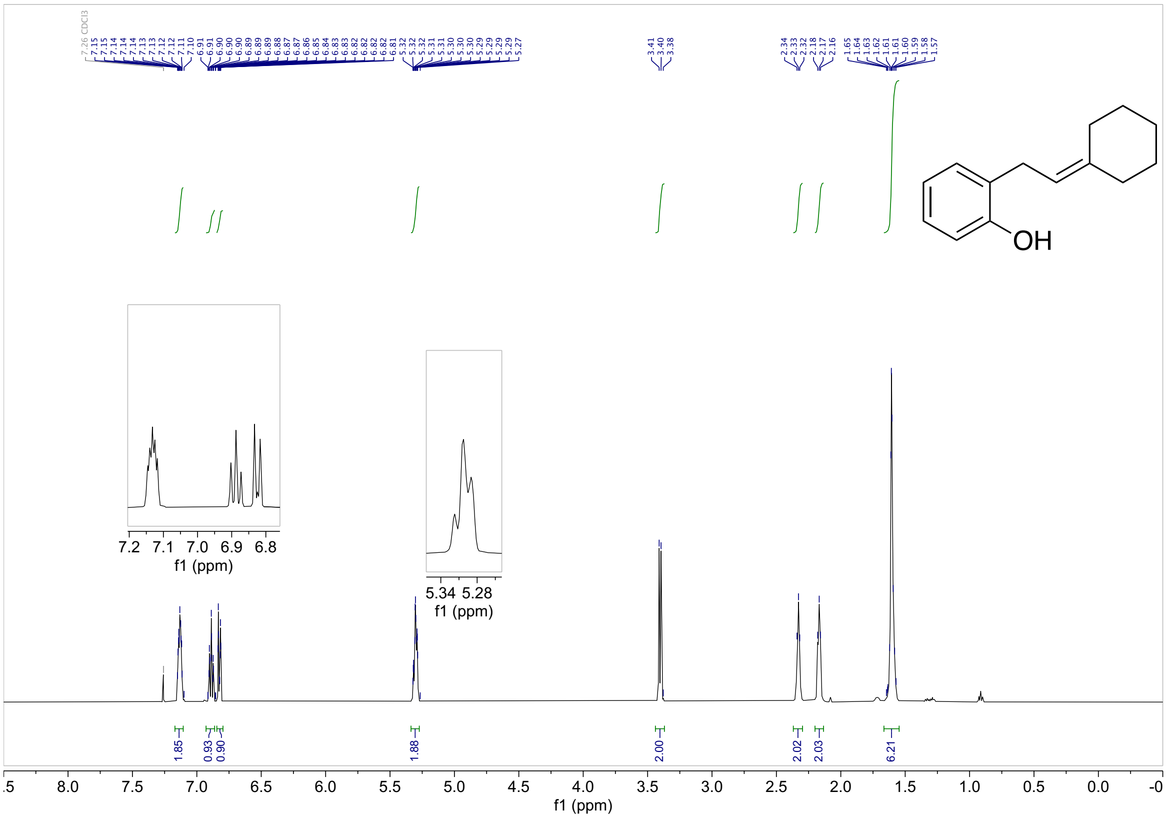

Compound 1v $^{13}C\{^{1}H\}$ NMR (126 MHz, $CDCl_3$, 298 K)

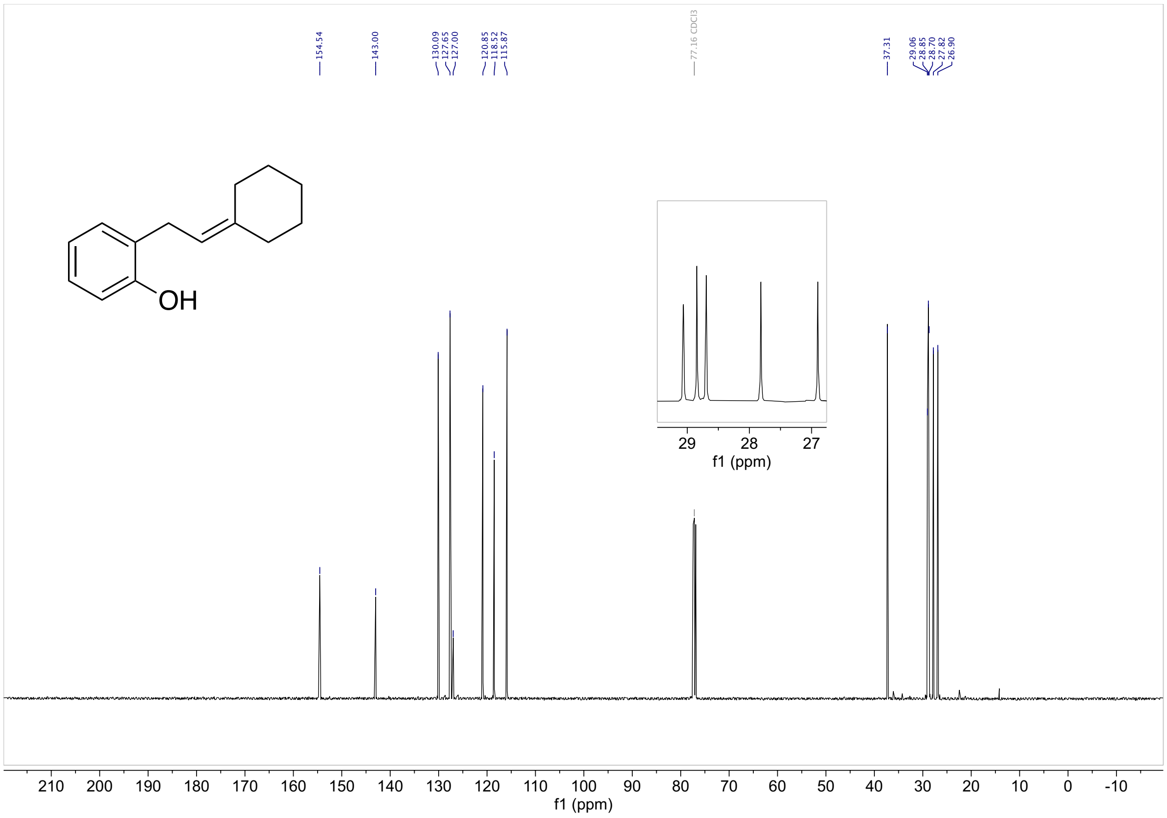

Compound 1w ^{1}H NMR (400 MHz, $CDCl_3$, 298 K)

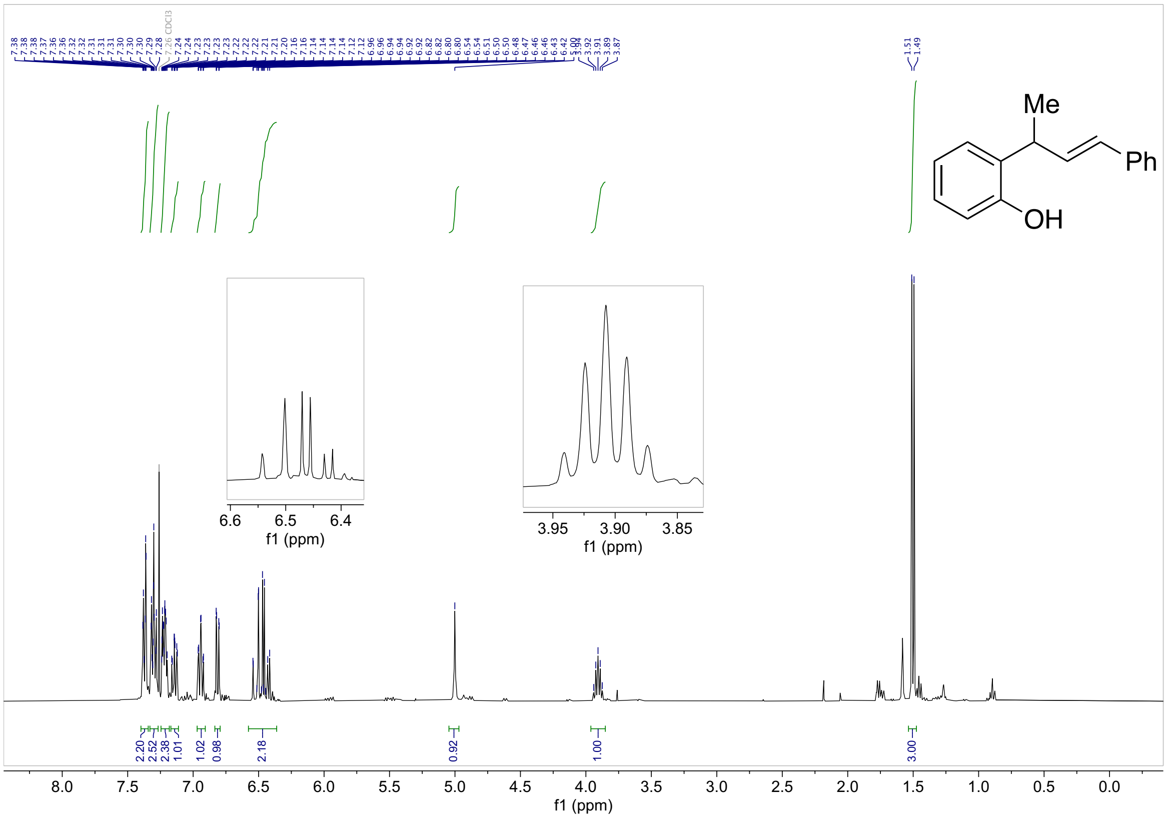

Compound 1w ^{13}C{^{1}H} NMR (101 MHz, $CDCl_3$, 298 K)

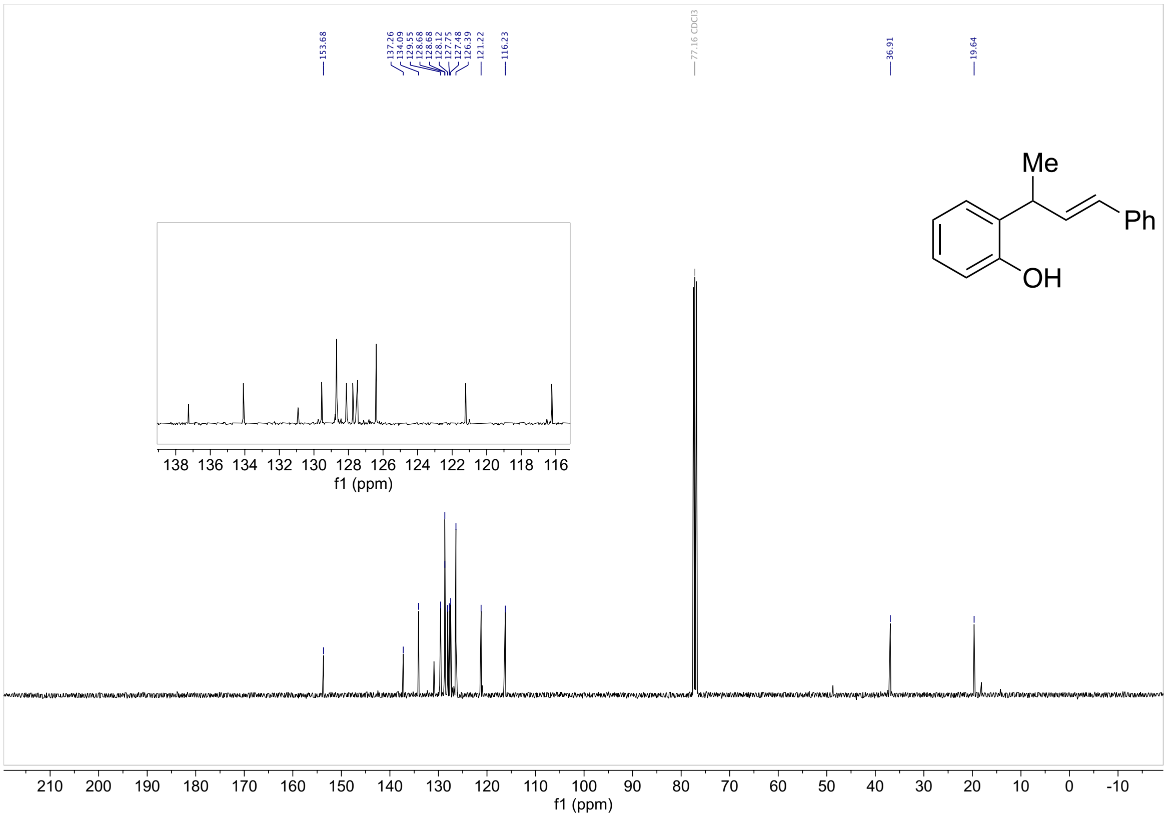

5.2 Products

Compound 2a ^{1}H NMR (400 MHz, $CDCl_3$, 298 K)

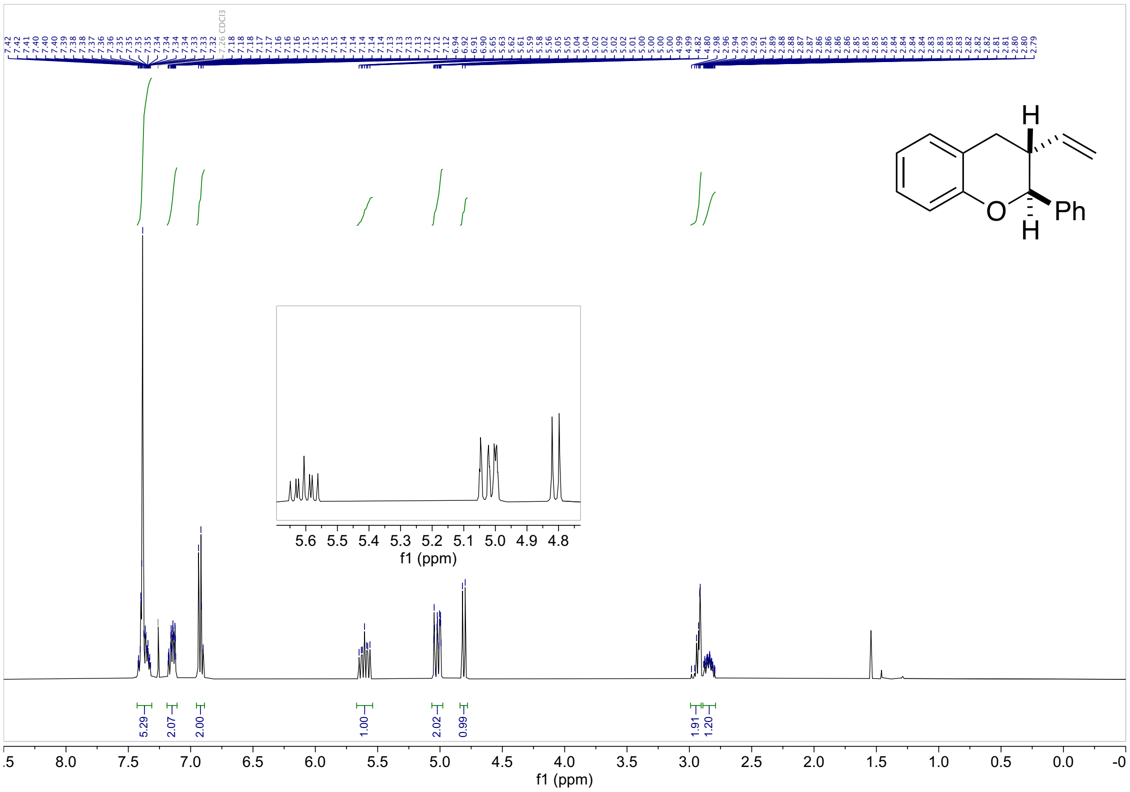

Compound 2a $^{13}C\{^{1}H\}$ NMR (126 MHz, $CDCl_3$, 298 K)

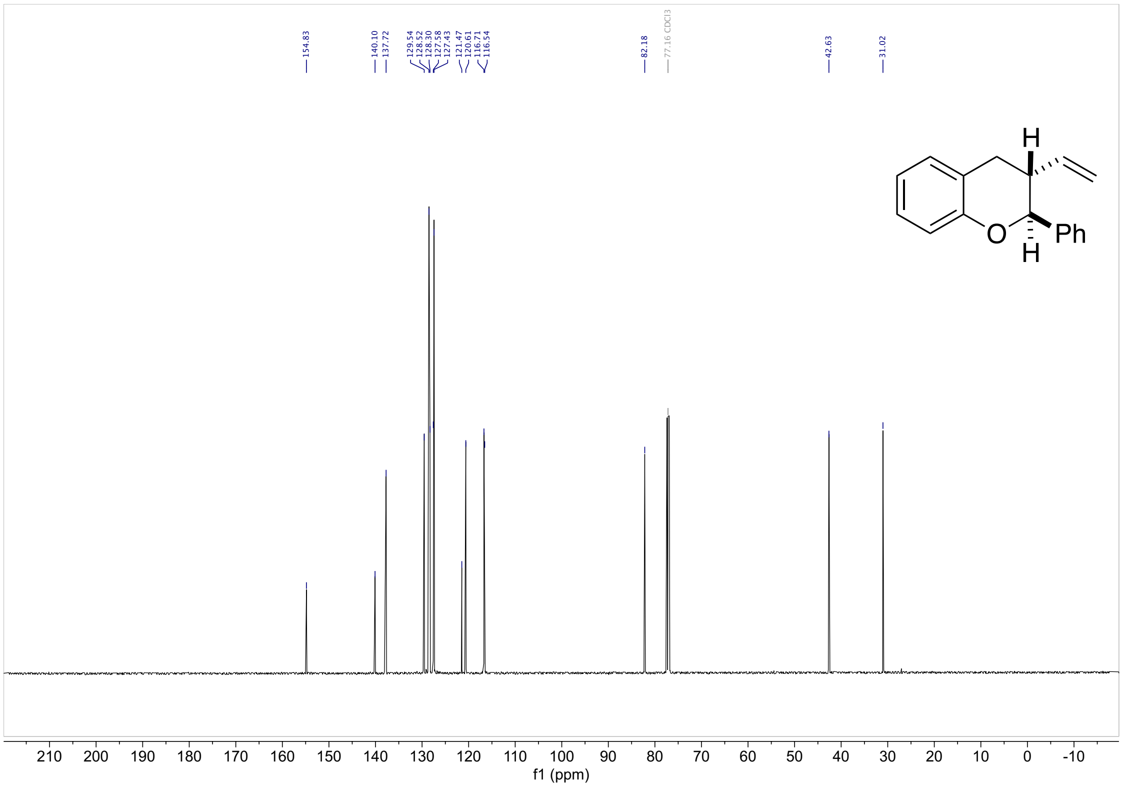

Compound 2b ¹H NMR (500 MHz, $CDCl_3$, 298 K)

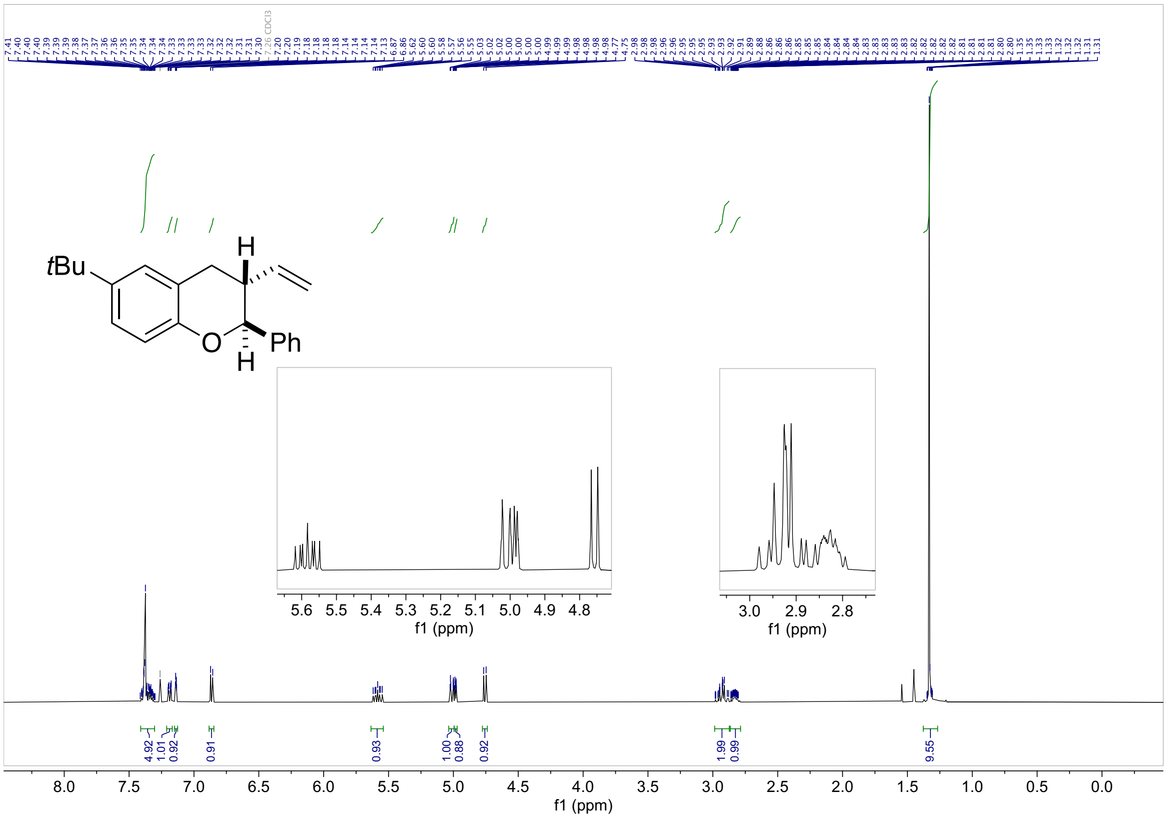

Compound 2b $^{13}C\{^1H\}$ NMR (126 MHz, $CDCl_3$, 298 K)

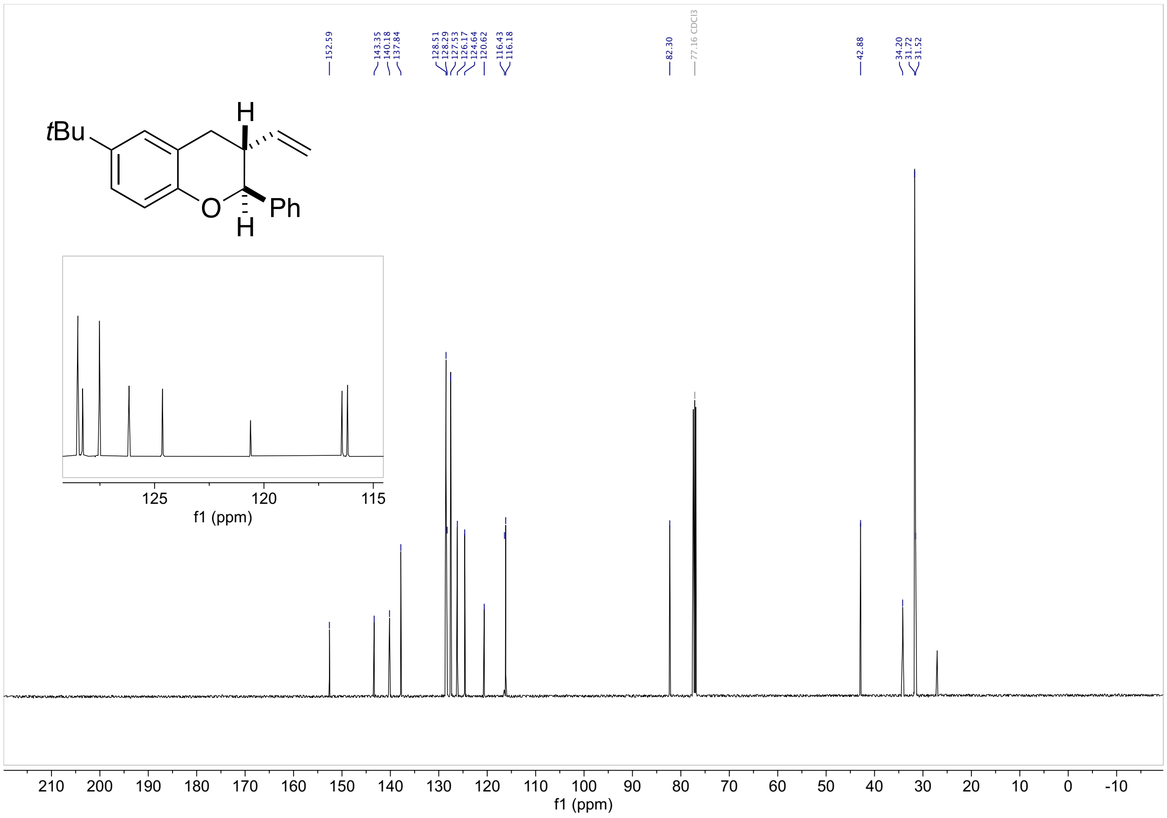

Compound 2c ^{1}H NMR (500 MHz, $CDCl_3$, 298 K)

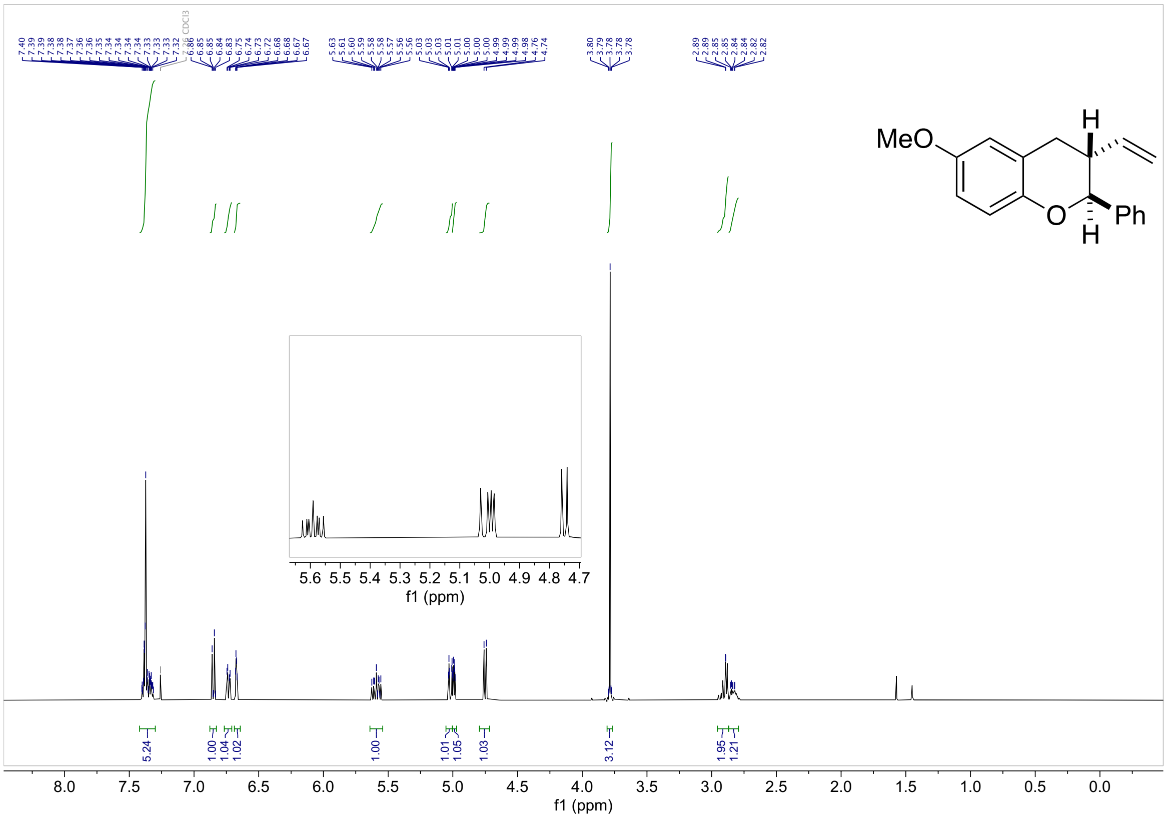

Compound 2c ^{13}C{^{1}H} NMR (126 MHz, $CDCl_3$, 298 K)

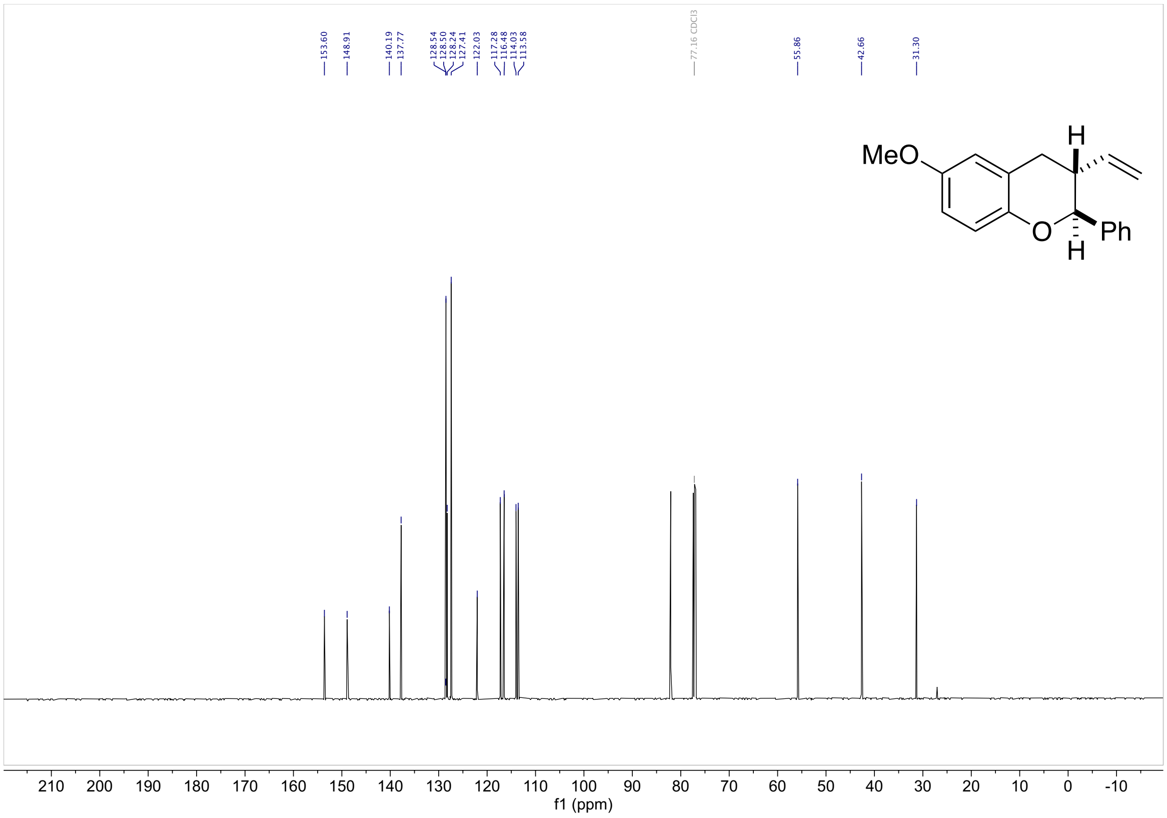

Compound 2d ^{1}H NMR (500 MHz, $CDCl_3$, 298 K)

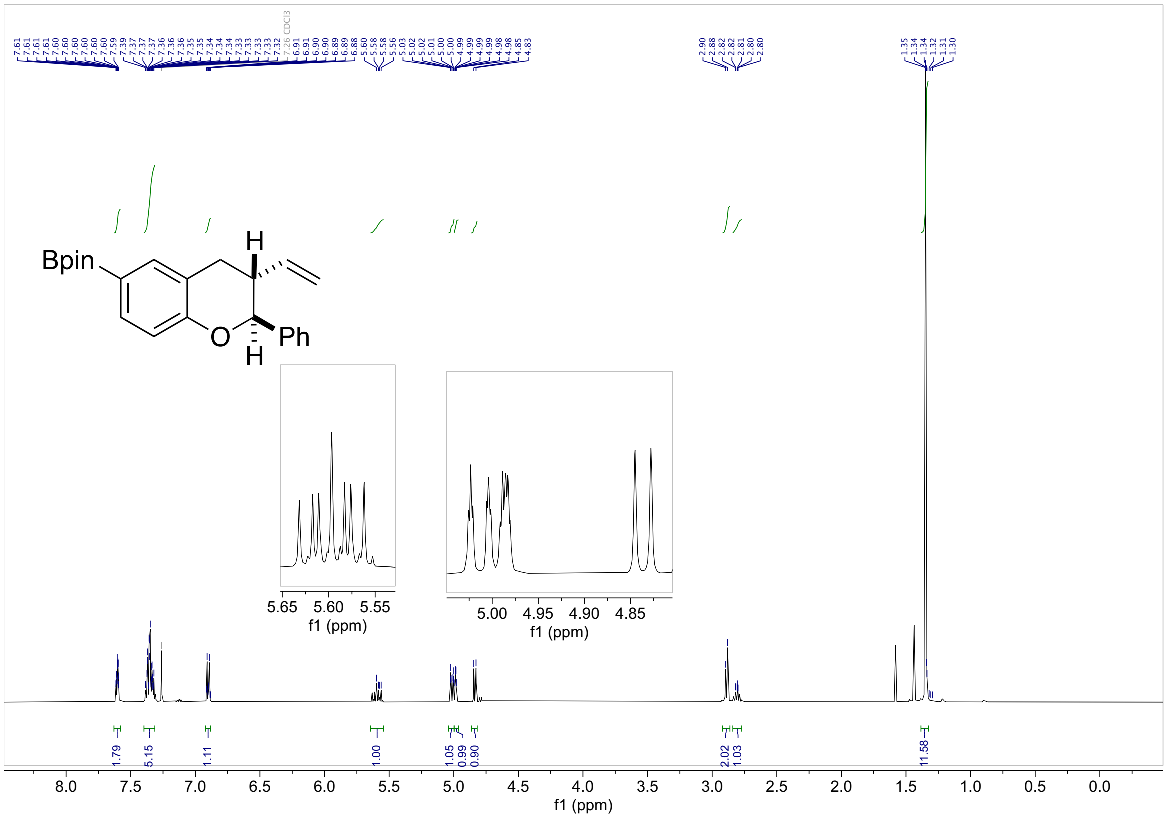

Compound 2d $^{13}C\{^{1}H\}$ NMR (126 MHz, $CDCl_3$, 298 K)

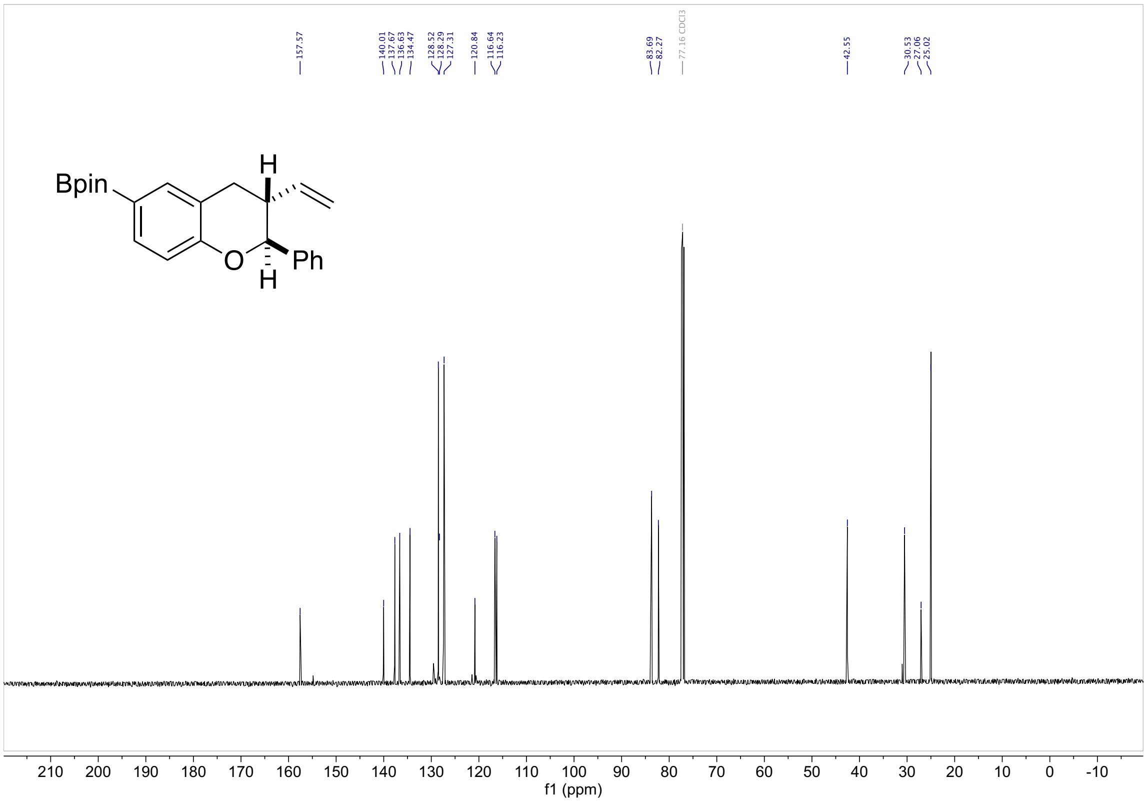

Compound 2e ^{1}H NMR (500 MHz, $CDCl_3$, 298 K)

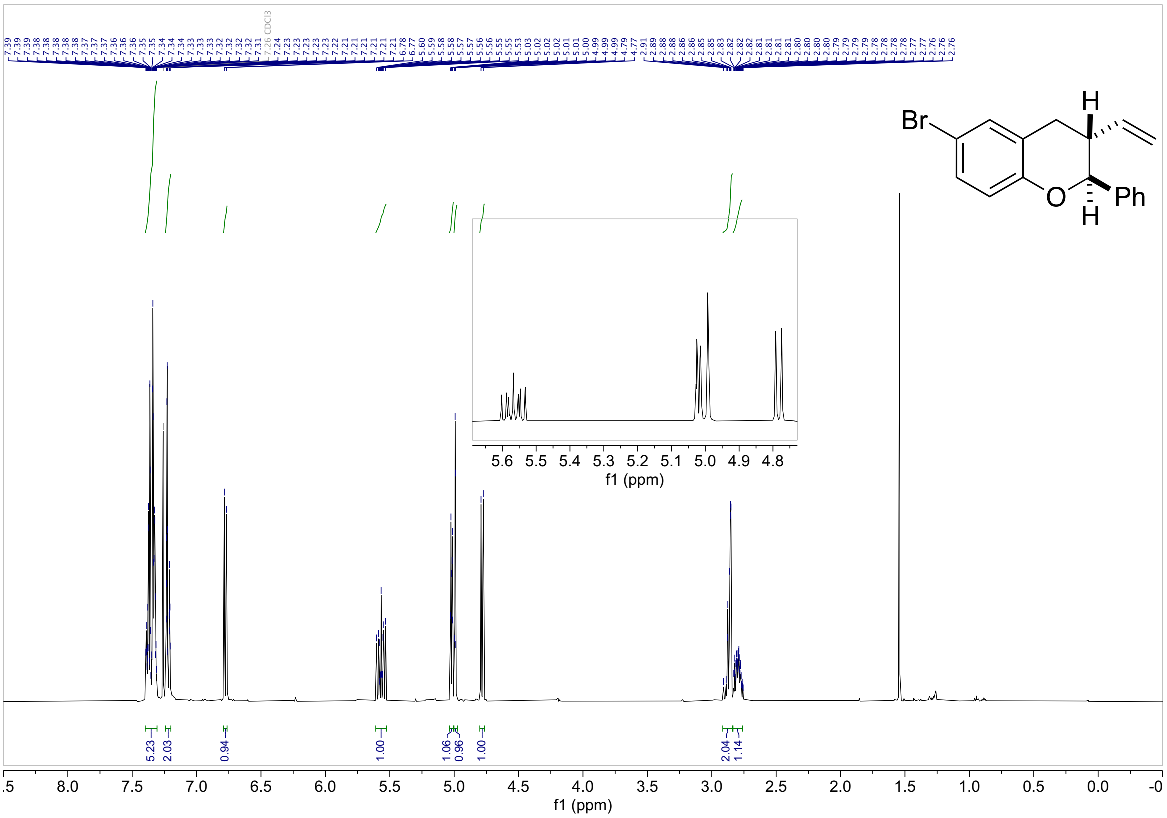

Compound 2e ^{13}C{1H} NMR (126 MHz, $CDCl_3$, 298 K)

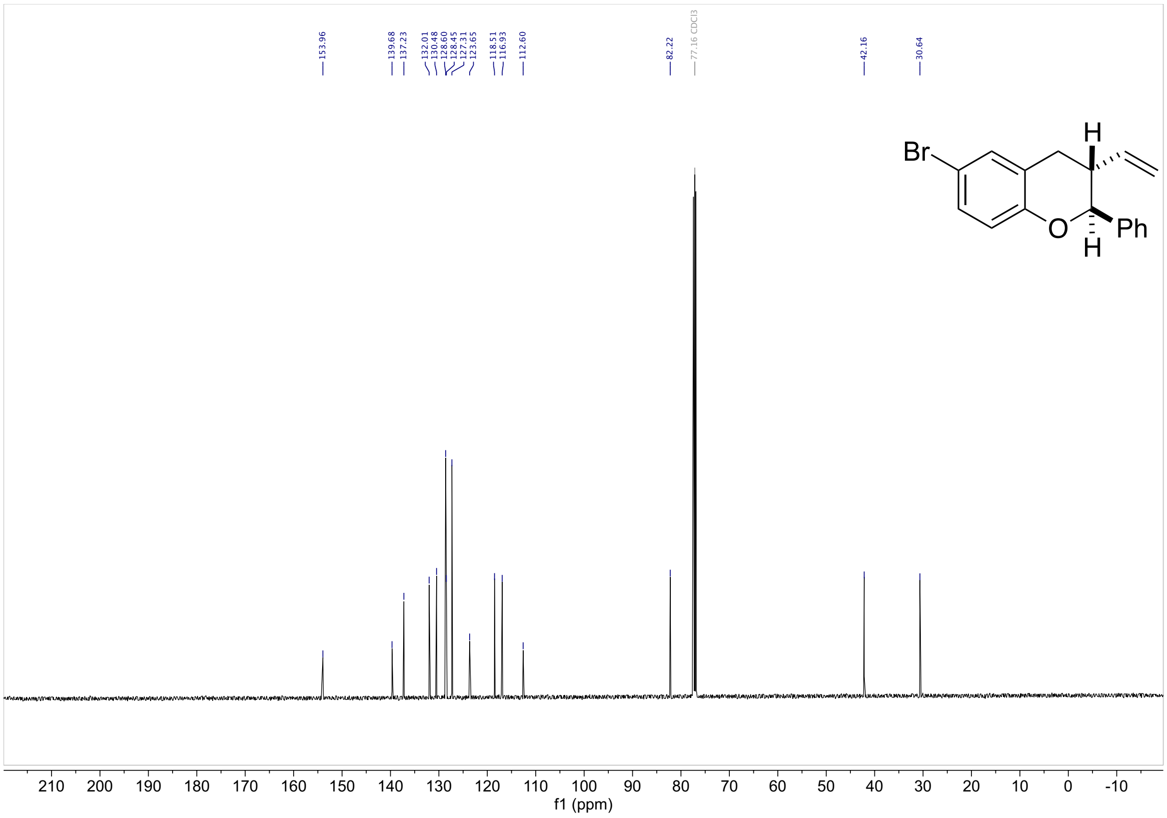

Compound 2f ^{1}H NMR (500 MHz, $CDCl_3$, 298 K)

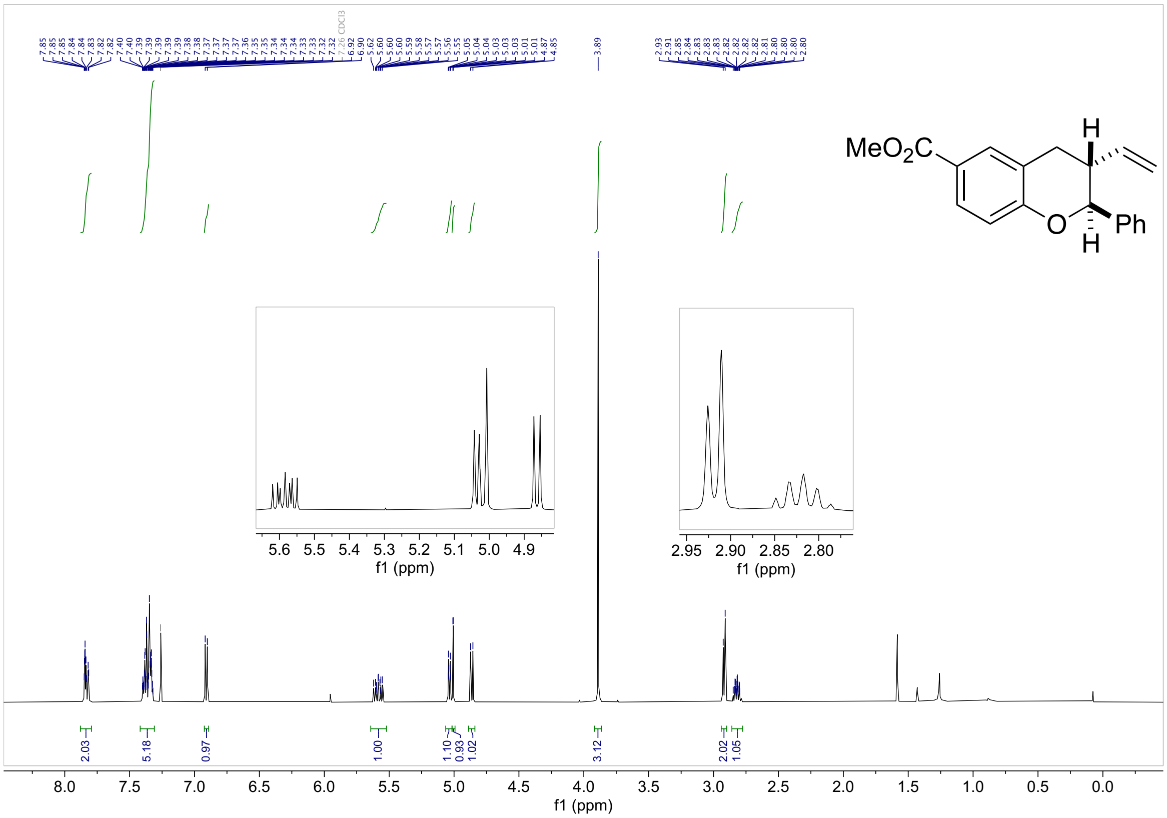

Compound 2f ^{13}C{1H} NMR (126 MHz, $CDCl_3$, 298 K)

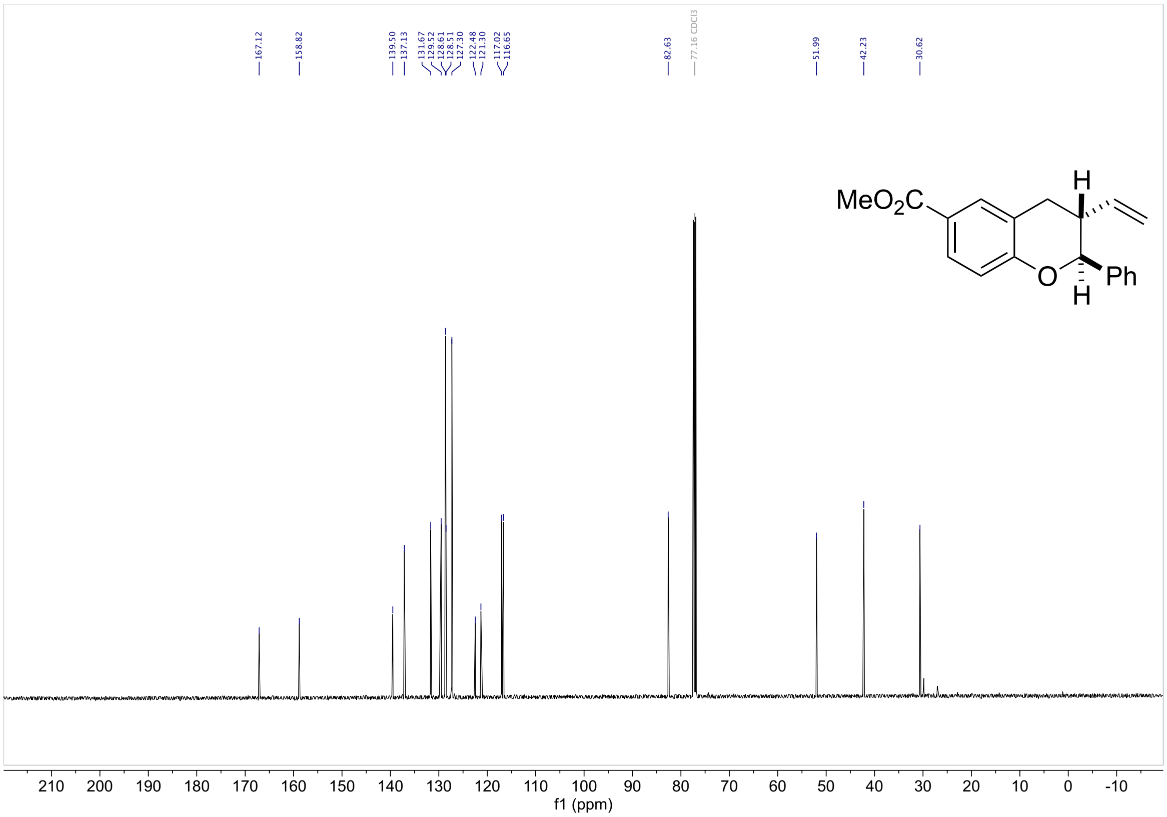

Compound 2g ^{1}H NMR (400 MHz, $CDCl_3$, 298 K)

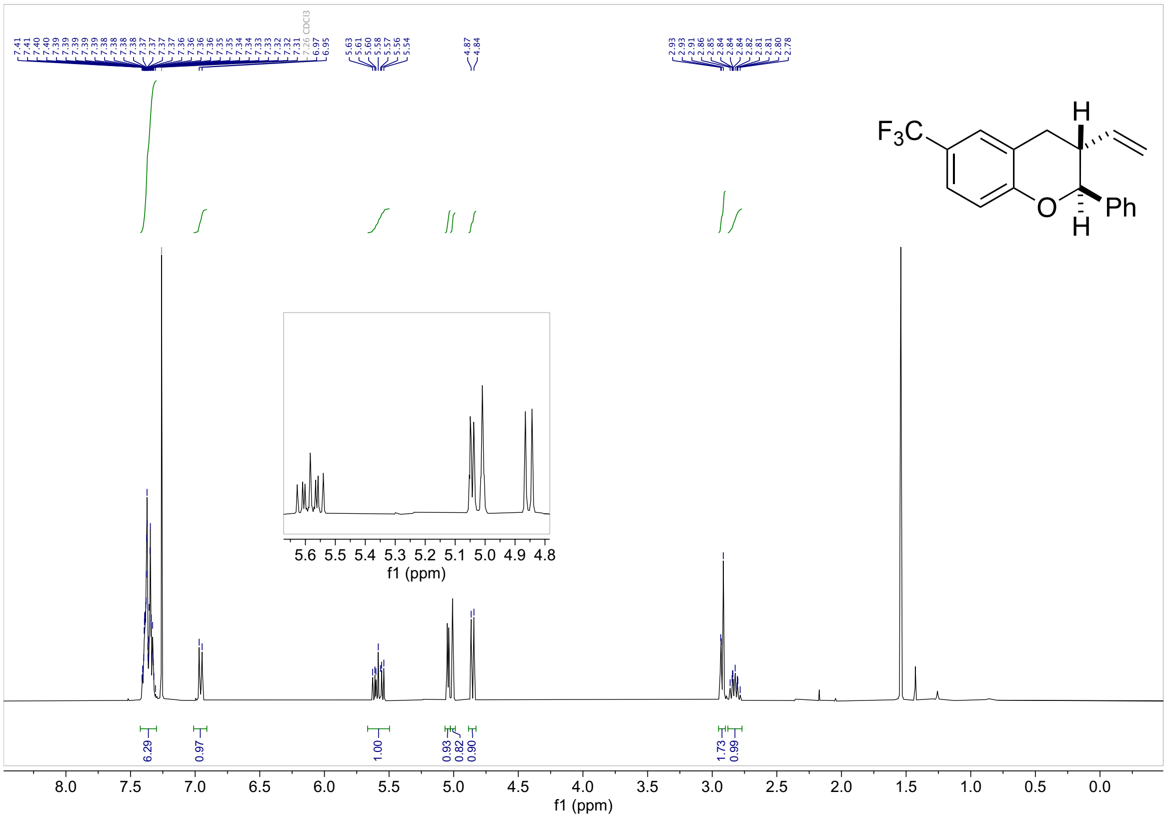

Compound 2g ^{13}C{1H} NMR (126 MHz, $CDCl_3$, 298 K)

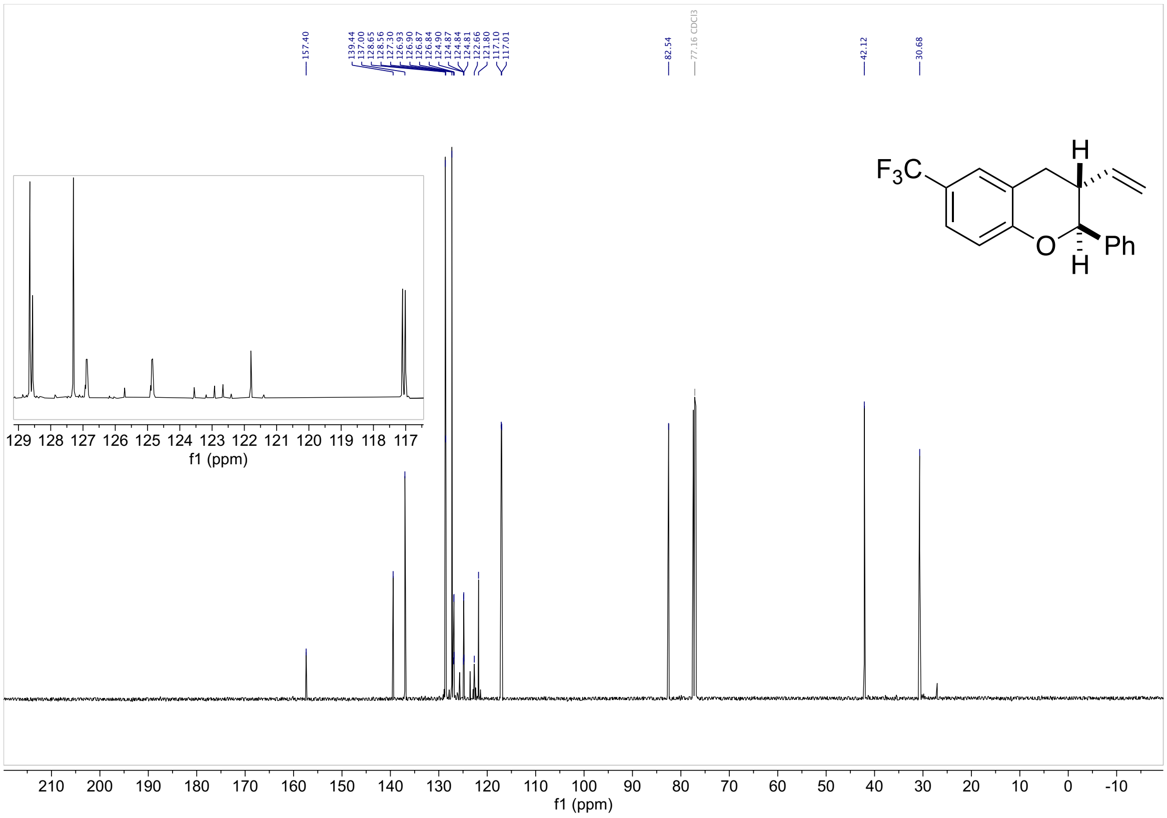

Compound 2g $^{19}F\{1H\}$ NMR (376 MHz, $CDCl_3$, 298 K)

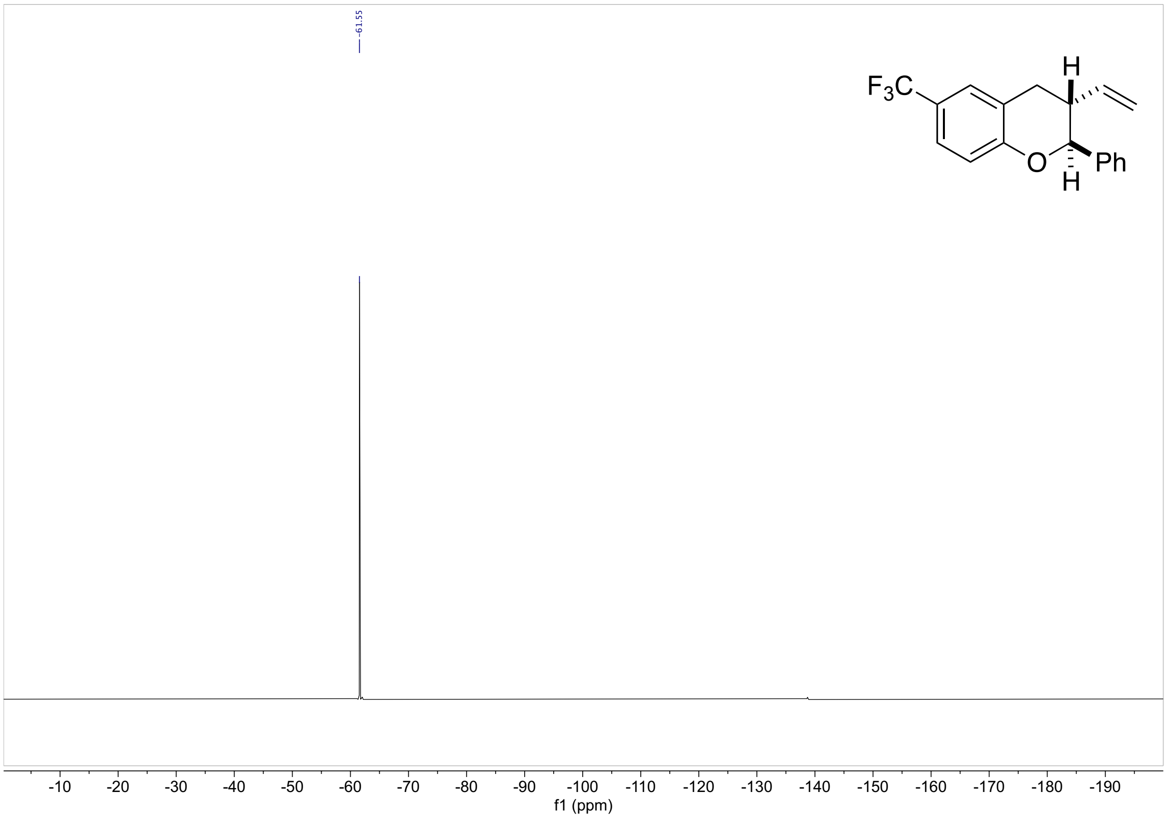

Compound 2h ^{1}H NMR (400 MHz, $CDCl_3$, 298 K)

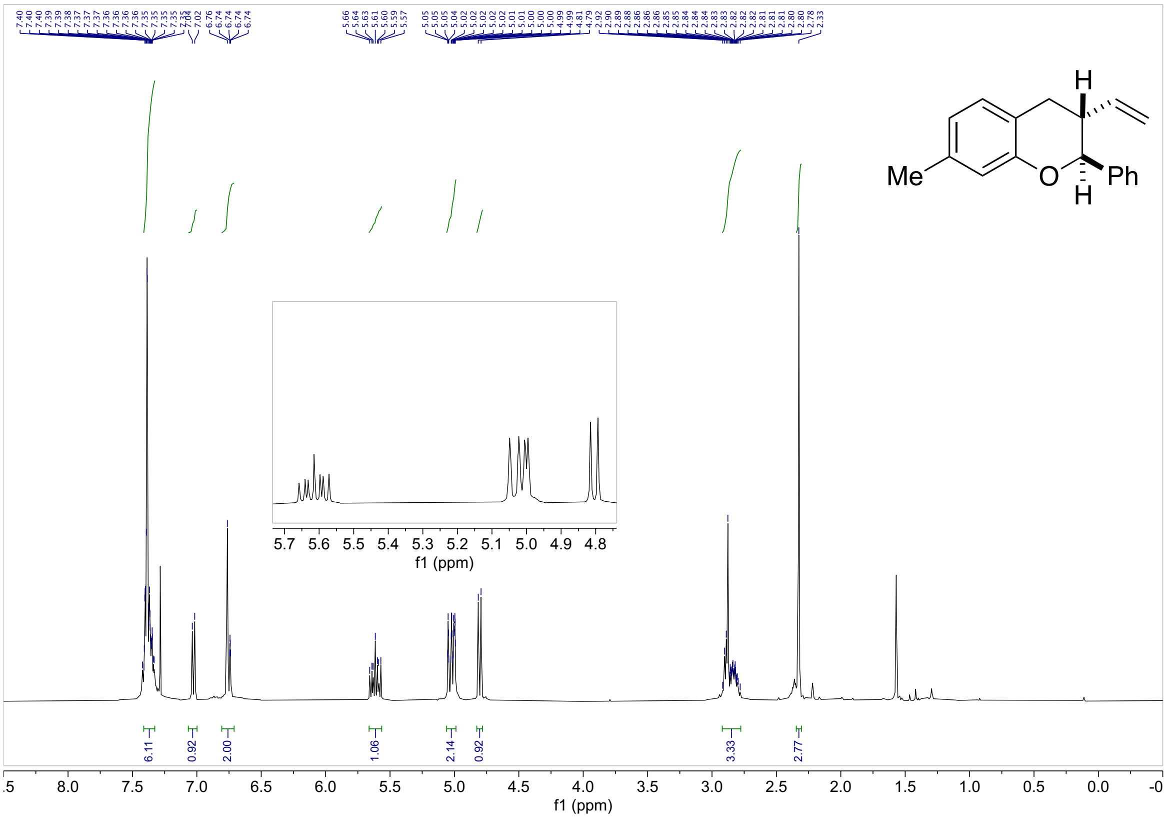

Compound 2h ^{13}C{^{1}H} NMR (400 MHz, $CDCl_3$, 298 K)

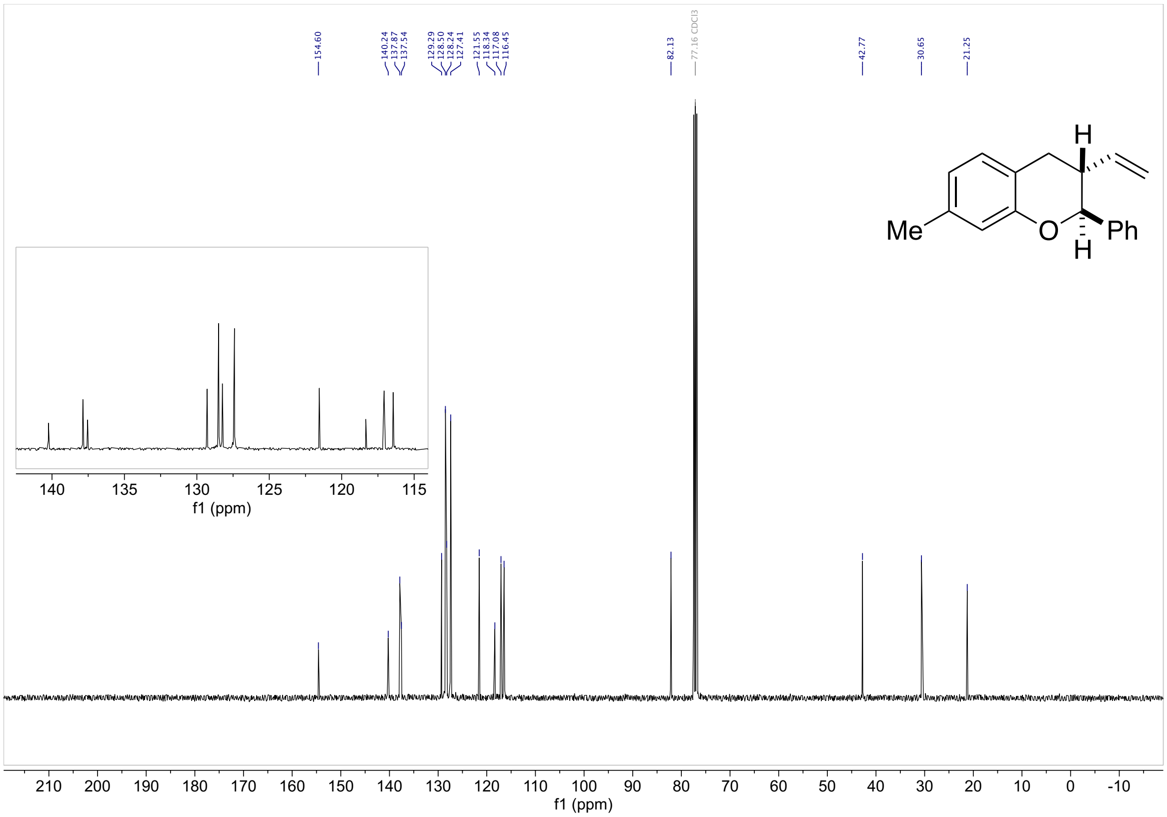

Compound 2i ^{1}H NMR (500 MHz, $CDCl_3$, 298 K)

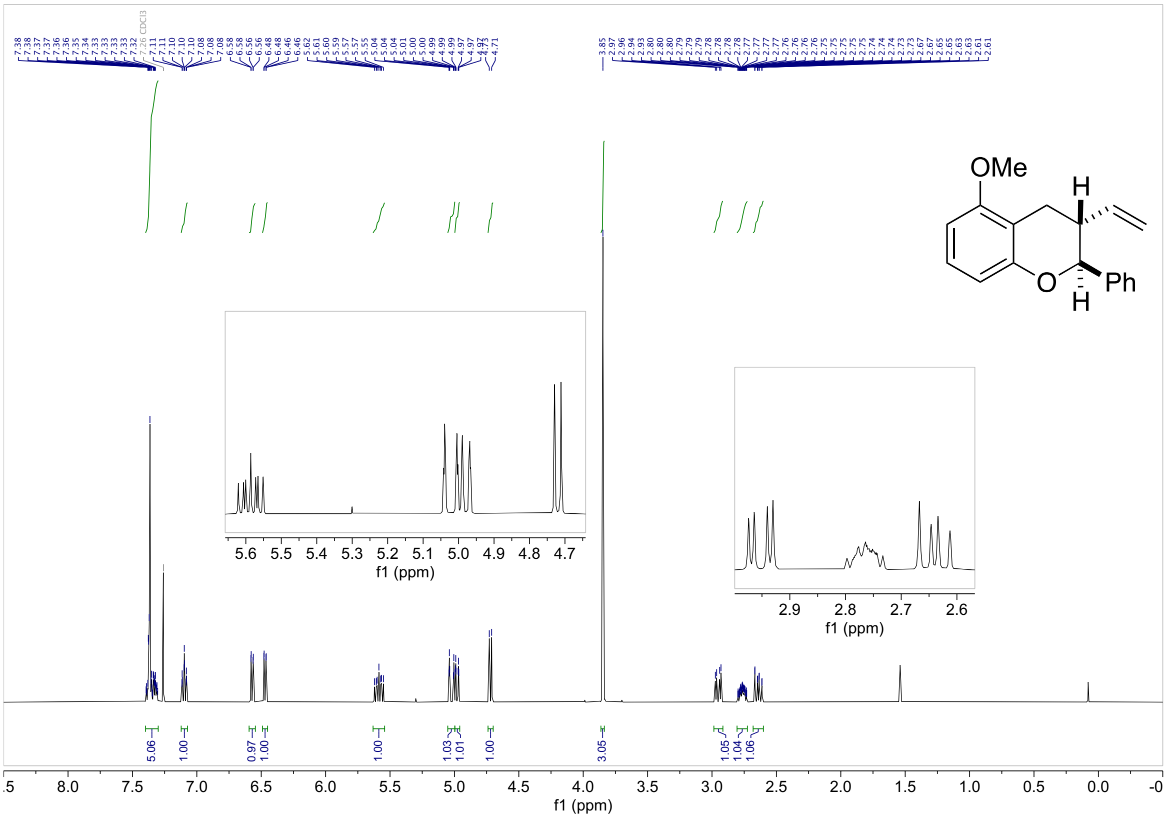

Compound 2i ^{13}C {^{1}H} NMR (126 MHz, $CDCl_3$, 298 K)

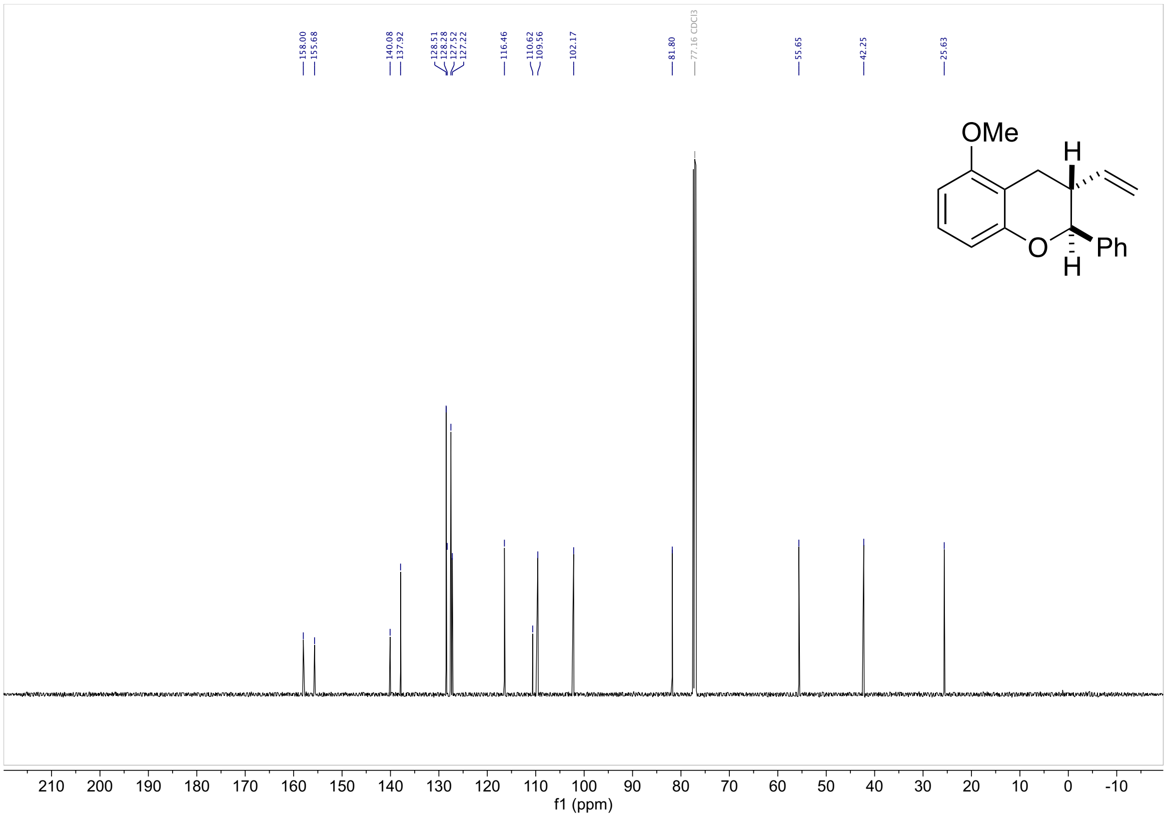

Compound 2j ^{1}H NMR (500 MHz, $CDCl_3$, 298 K)

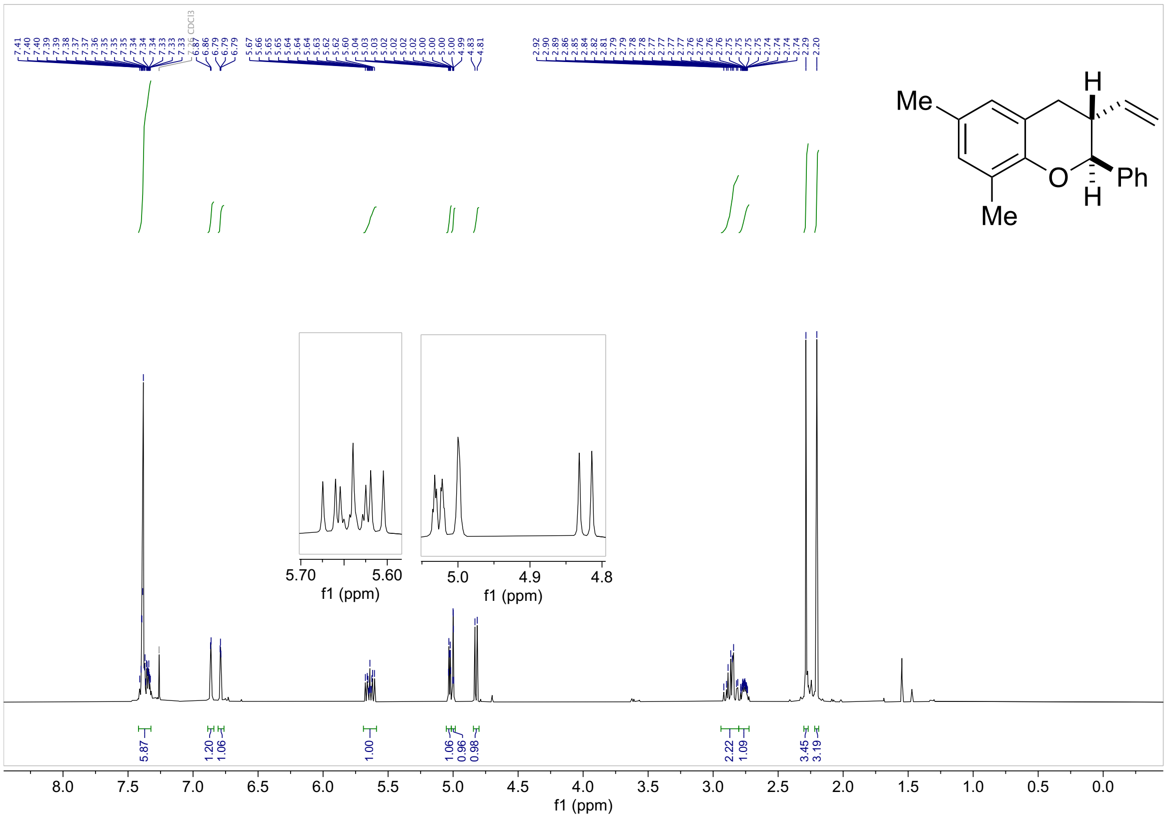

Compound 2j ^{13}C {^{1}H} NMR (126 MHz, $CDCl_3$, 298 K)

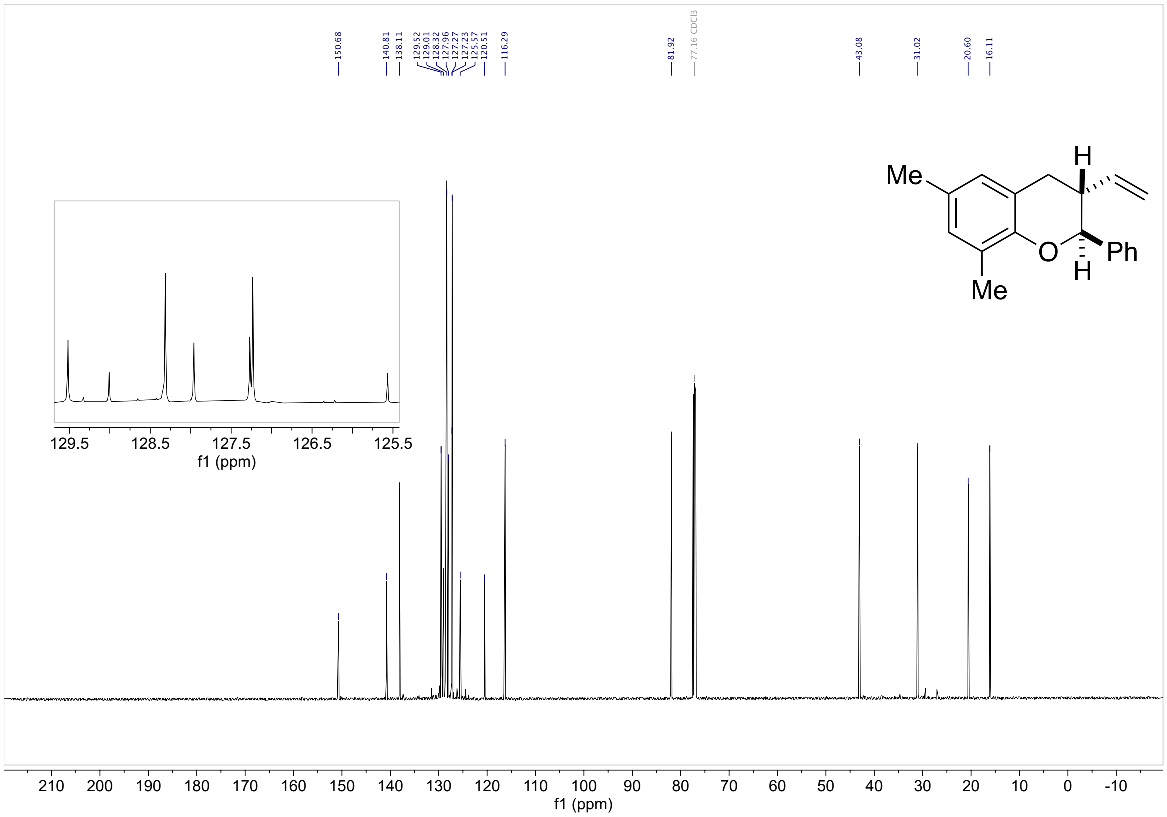

Compound 2k ^{1}H NMR (500 MHz, CDCl$_3$, 298 K)

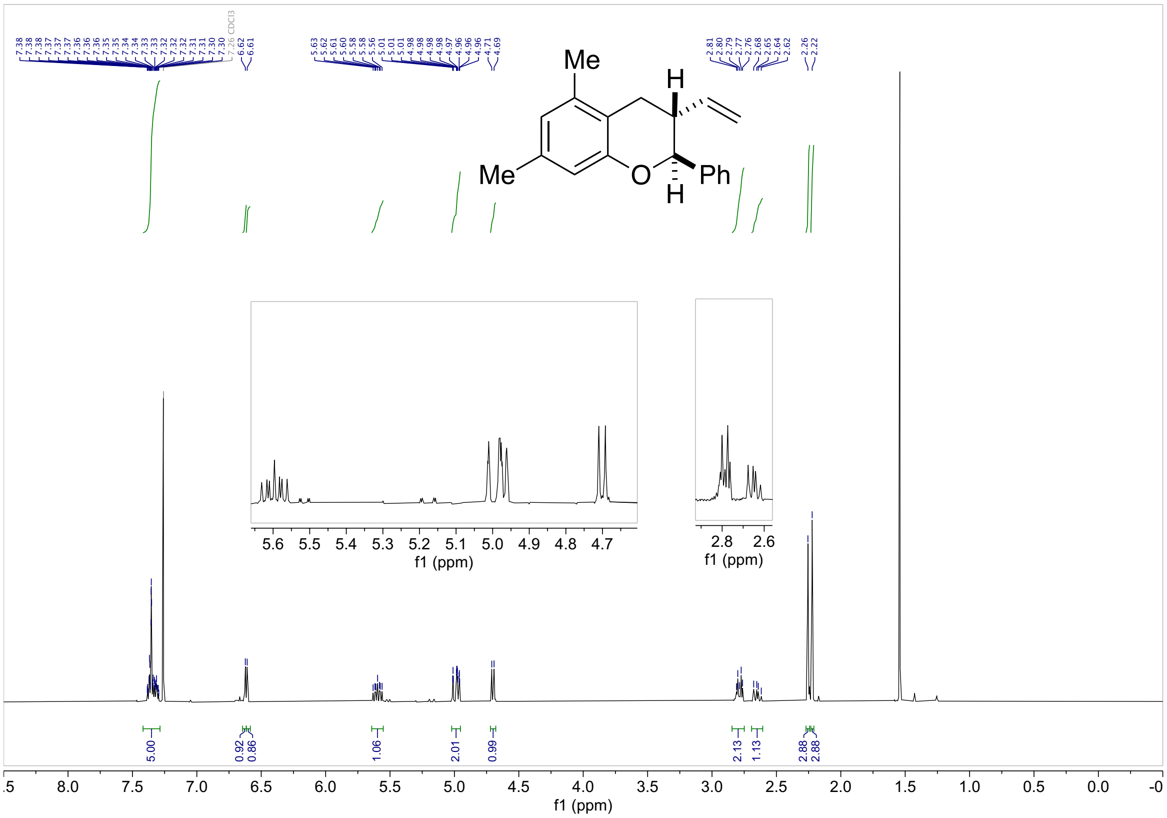

Compound 2k ^{13}C{1H} NMR (126 MHz, CDCl$_3$, 298 K)

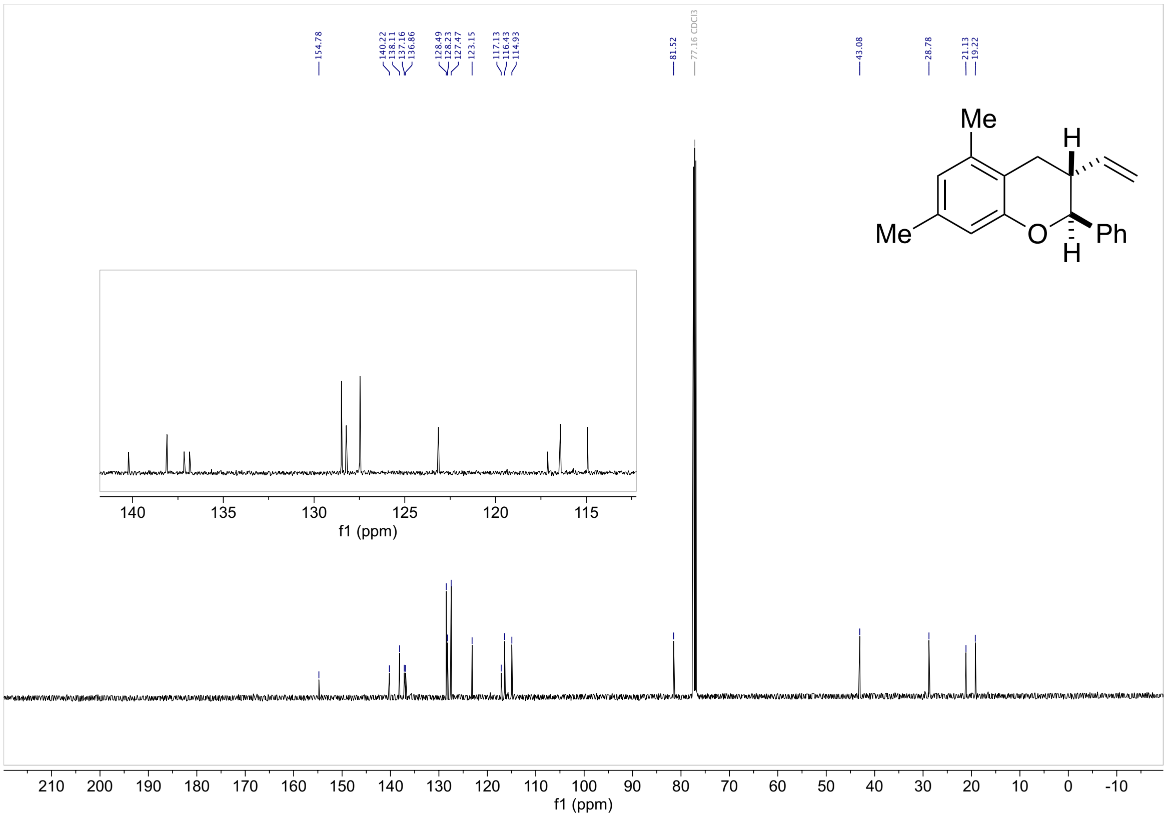

Compound 2l ^{1}H NMR (500 MHz, $CDCl_3$, 298 K)

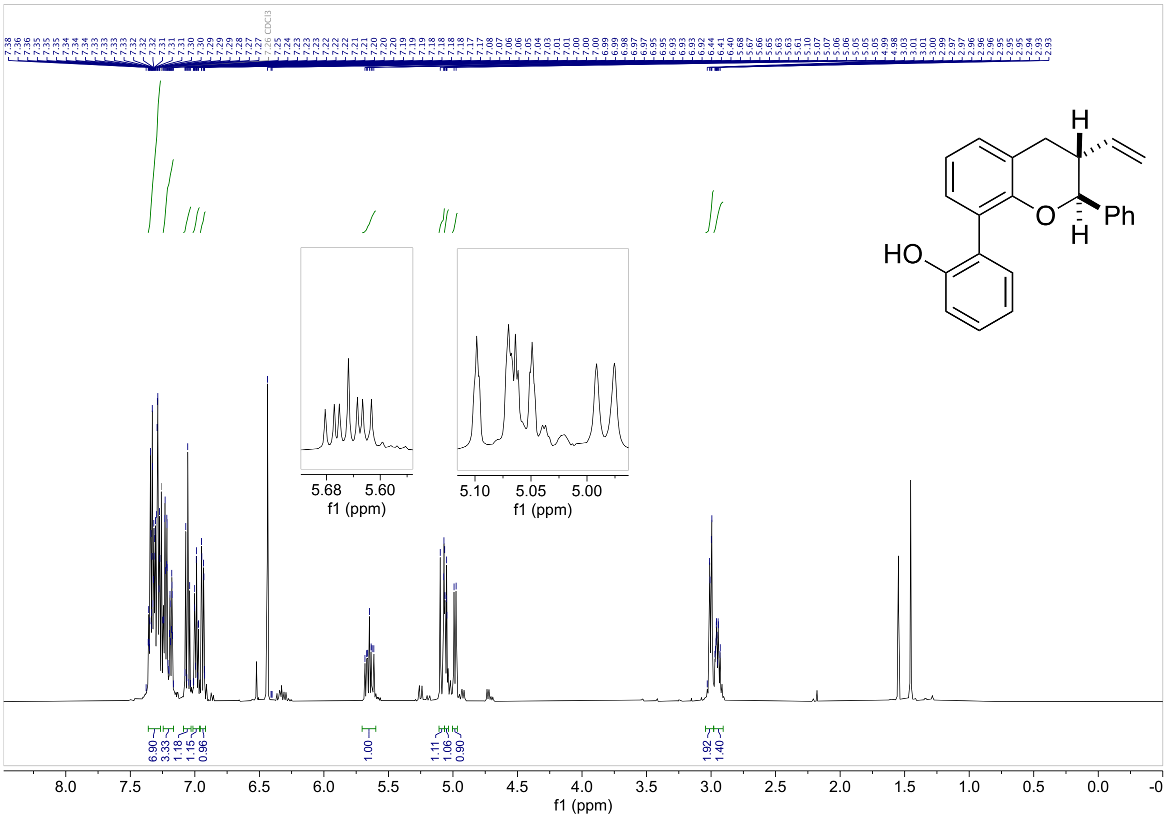

Compound 2l ^{13}C{1H} NMR (126 MHz, $CDCl_3$, 298 K)

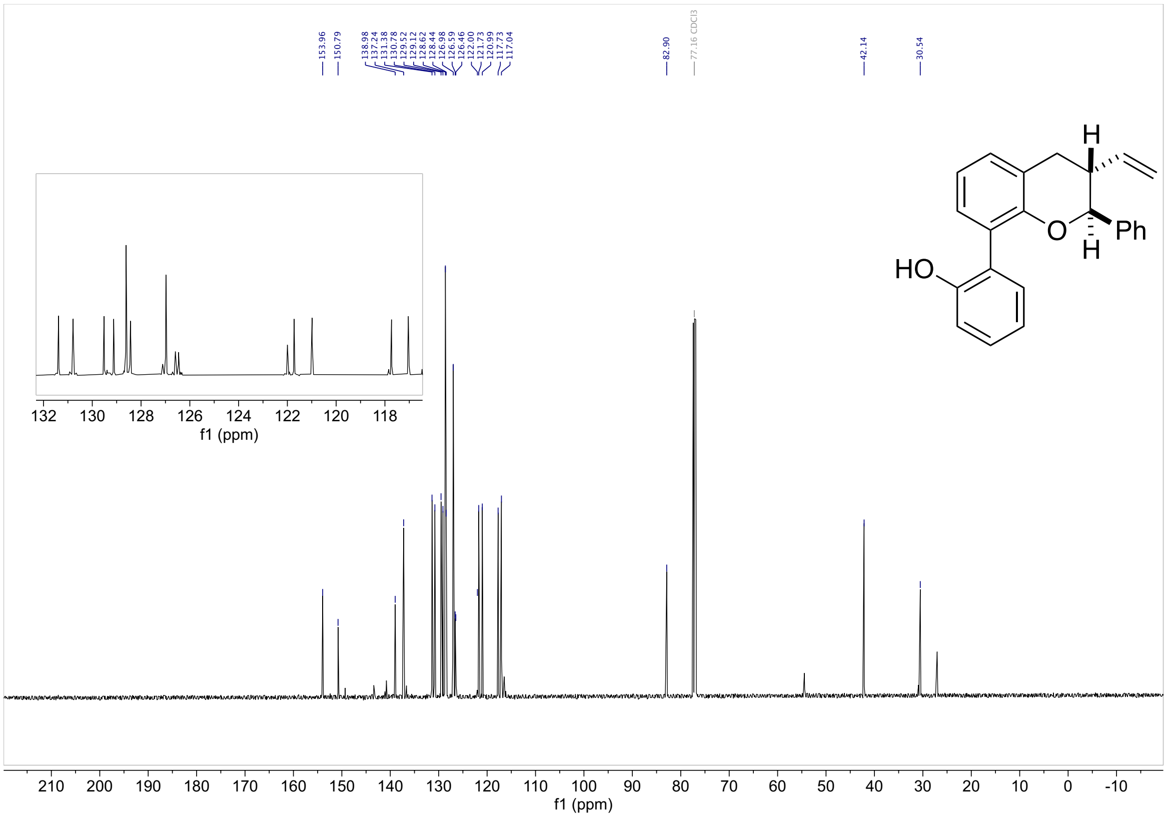

Compound 2m ^{1}H NMR (500 MHz, $CDCl_3$, 298 K)

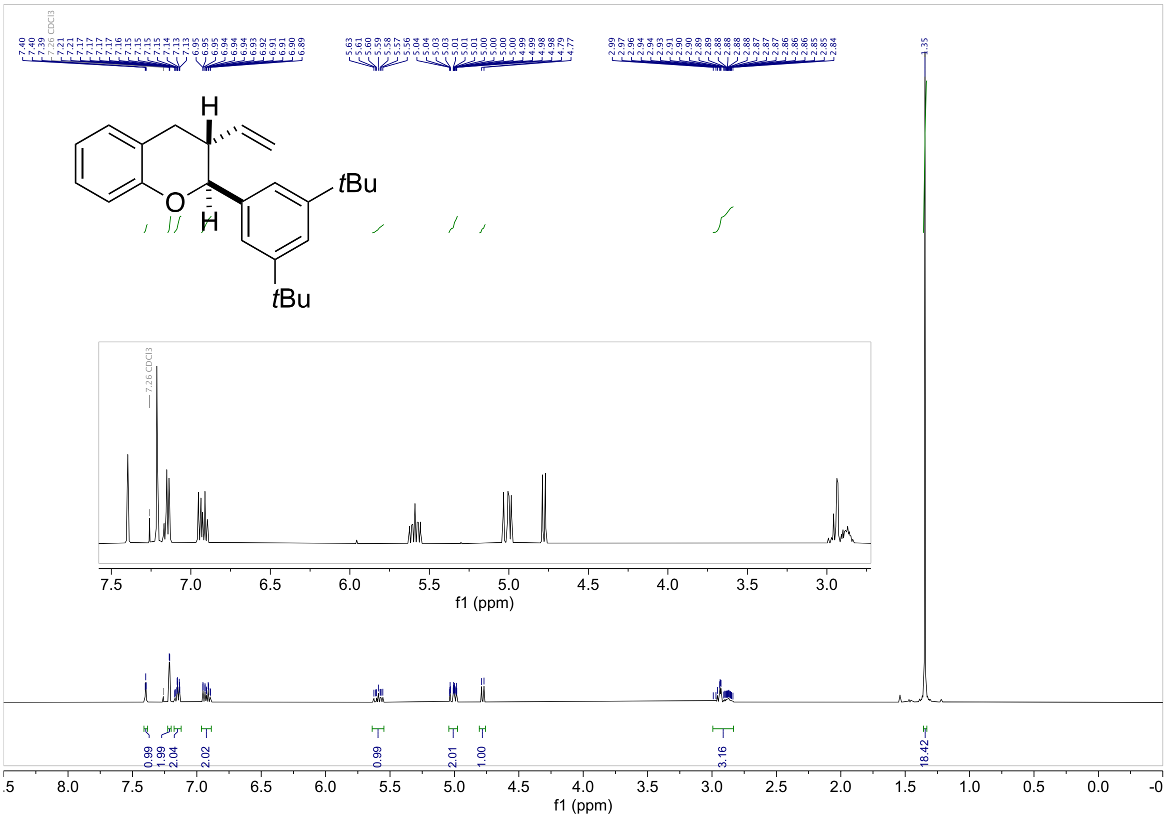

Compound 2m ^{13}C{1H} NMR (126 MHz, $CDCl_3$, 298 K)

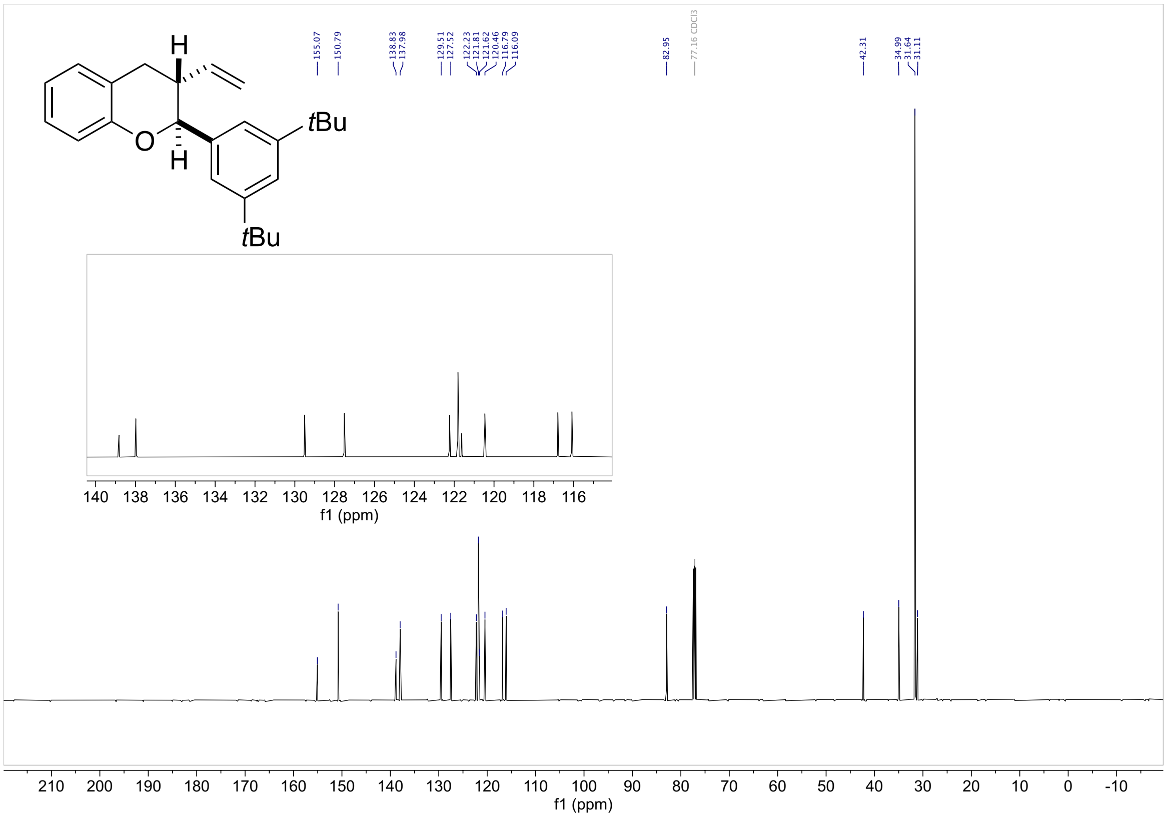

Compound 2n ^{1}H NMR (500 MHz, $CDCl_3$, 298 K)

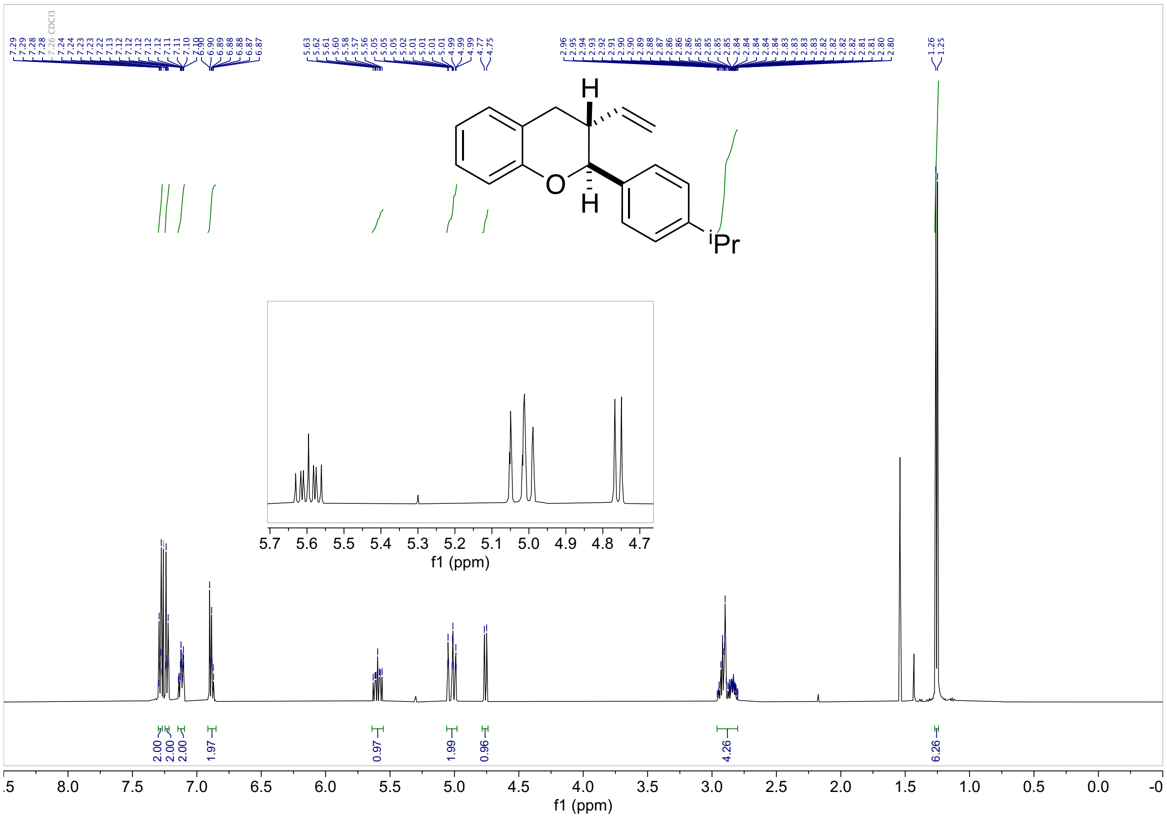

Compound 2n ^{13}C{1H} NMR (126 MHz, $CDCl_3$, 298 K)

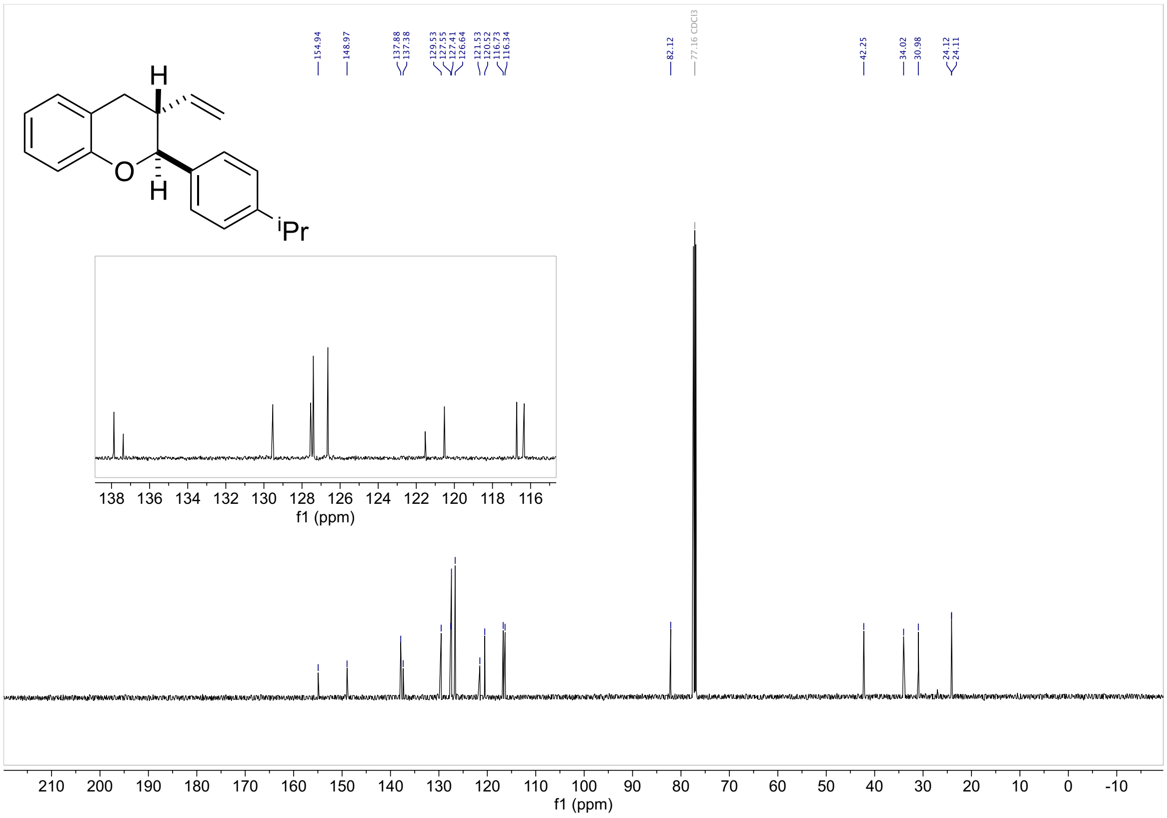

Compound 2o ^{1}H NMR (500 MHz, $CDCl_3$, 298 K)

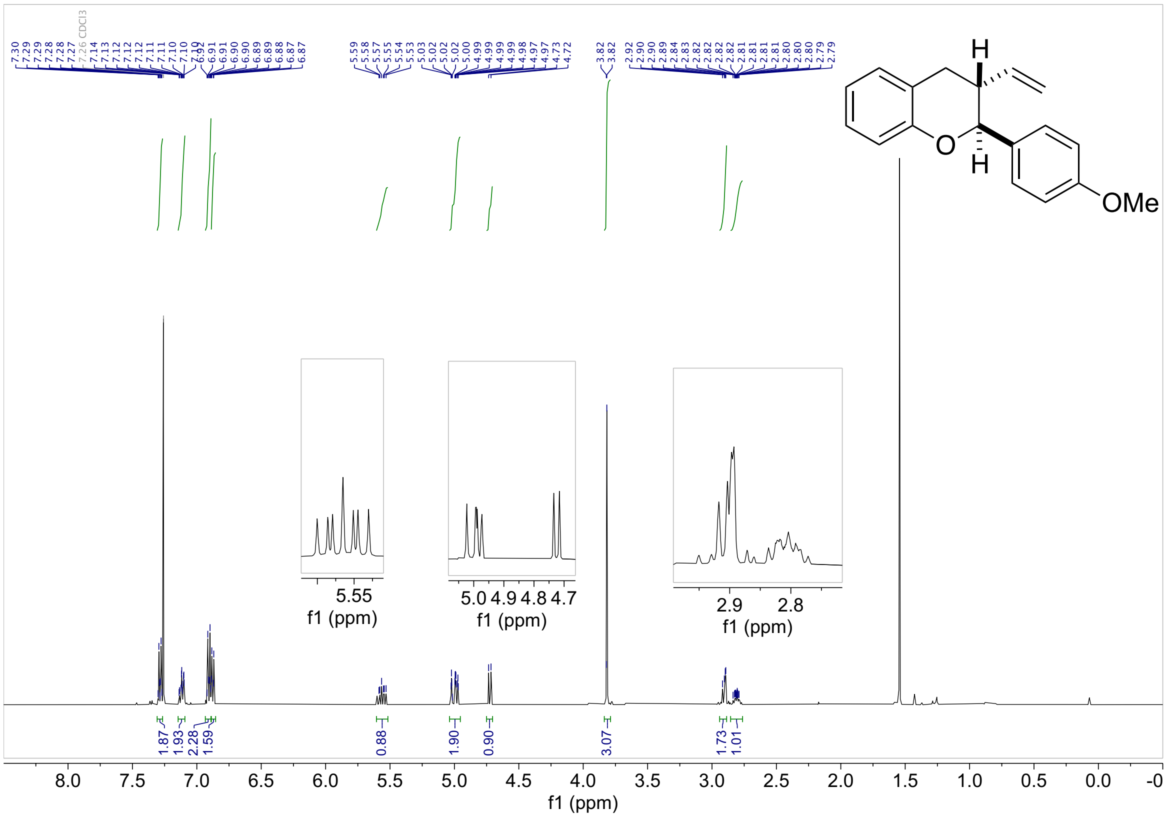

Compound 2o ^{13}C{1H} NMR (126 MHz, $CDCl_3$, 298 K)

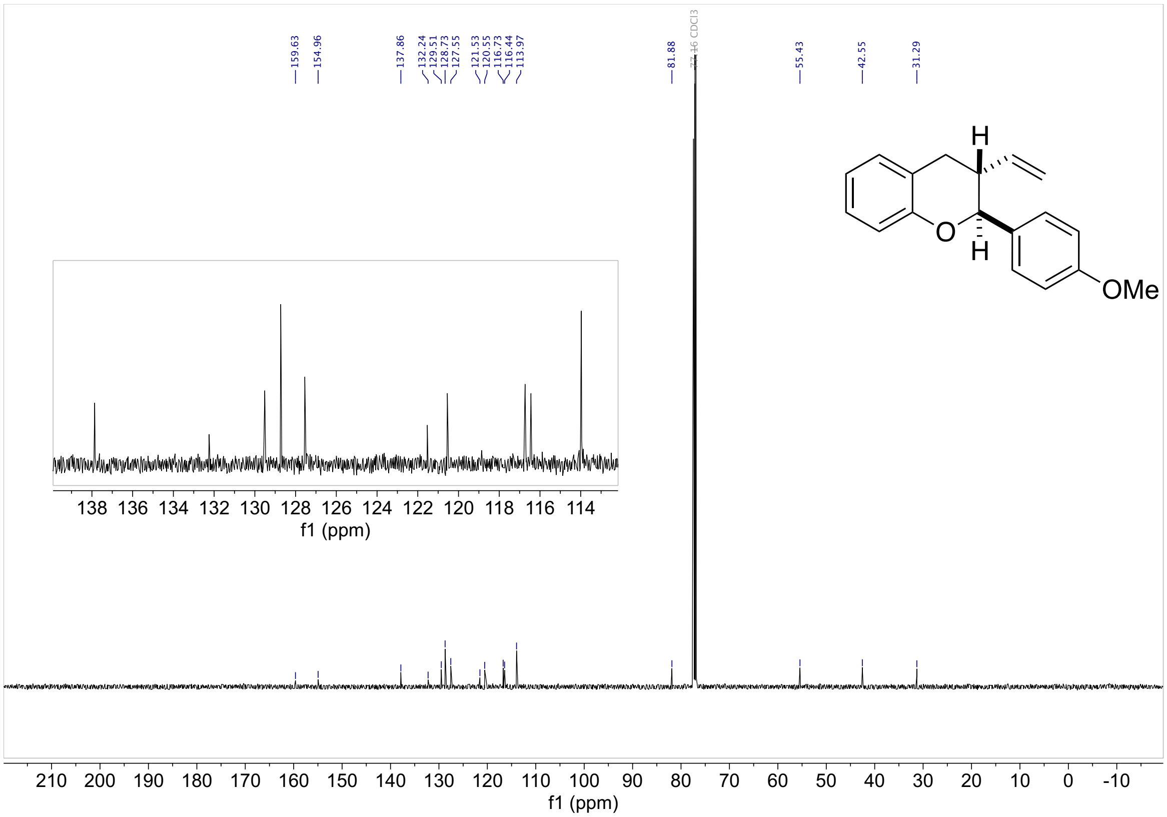

Compound 2p ^{1}H NMR (500 MHz, $CDCl_3$, 298 K)

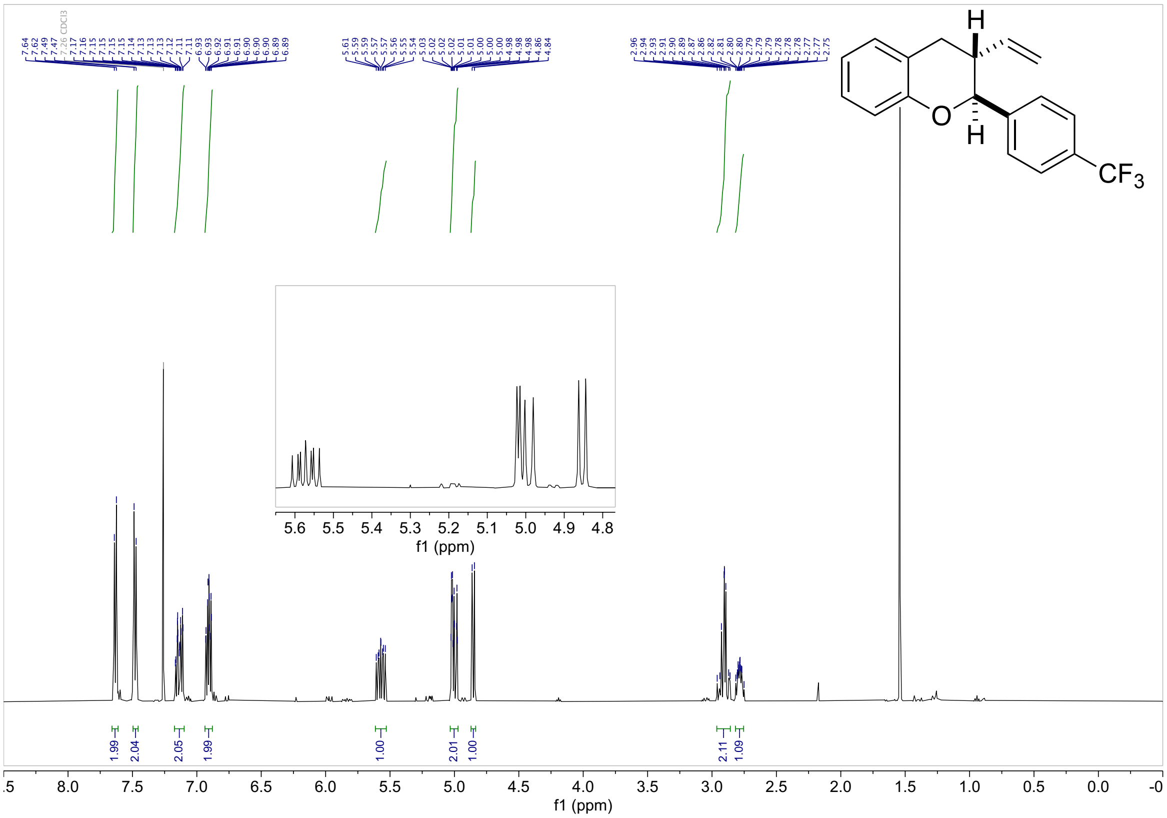

Compound 2p $^{13}C\{^{1}H\}$ NMR (126 MHz, $CDCl_3$, 298 K)

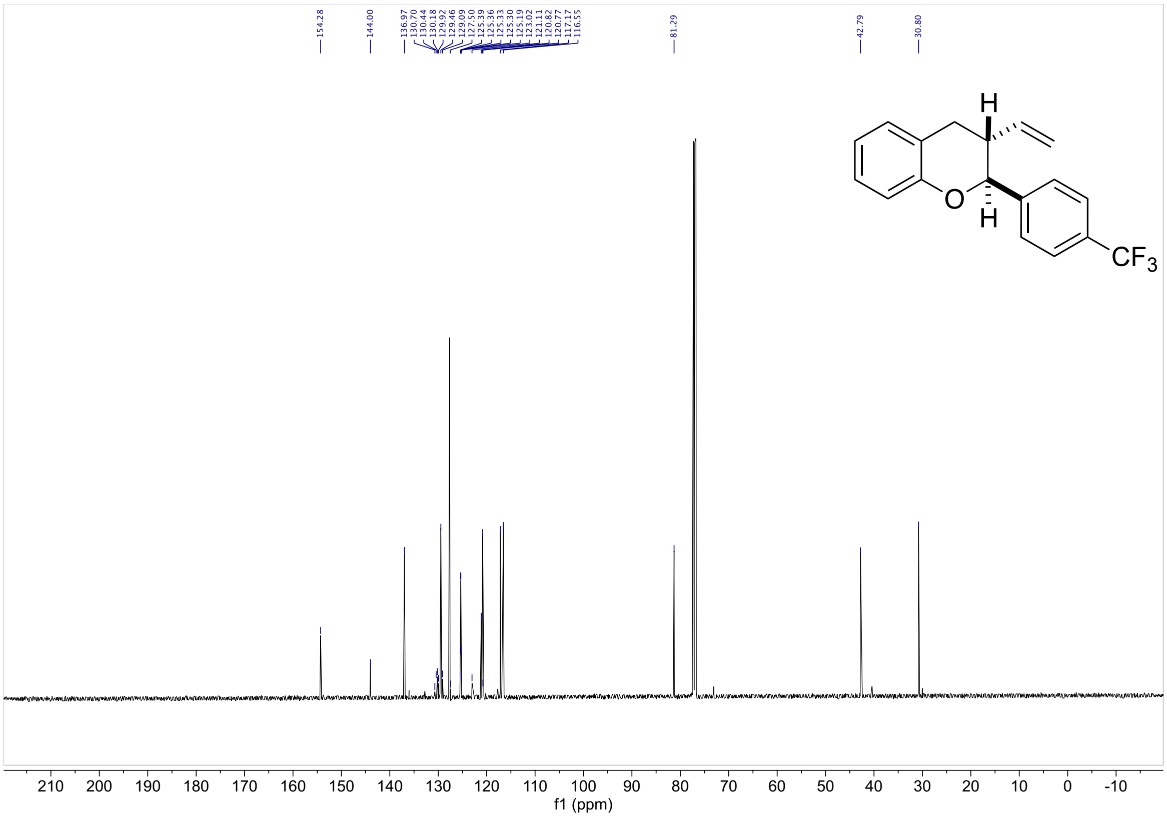

Compound 2p $^{19}F\{^{1}H\}$ NMR (471, $CDCl_3$, 298 K)

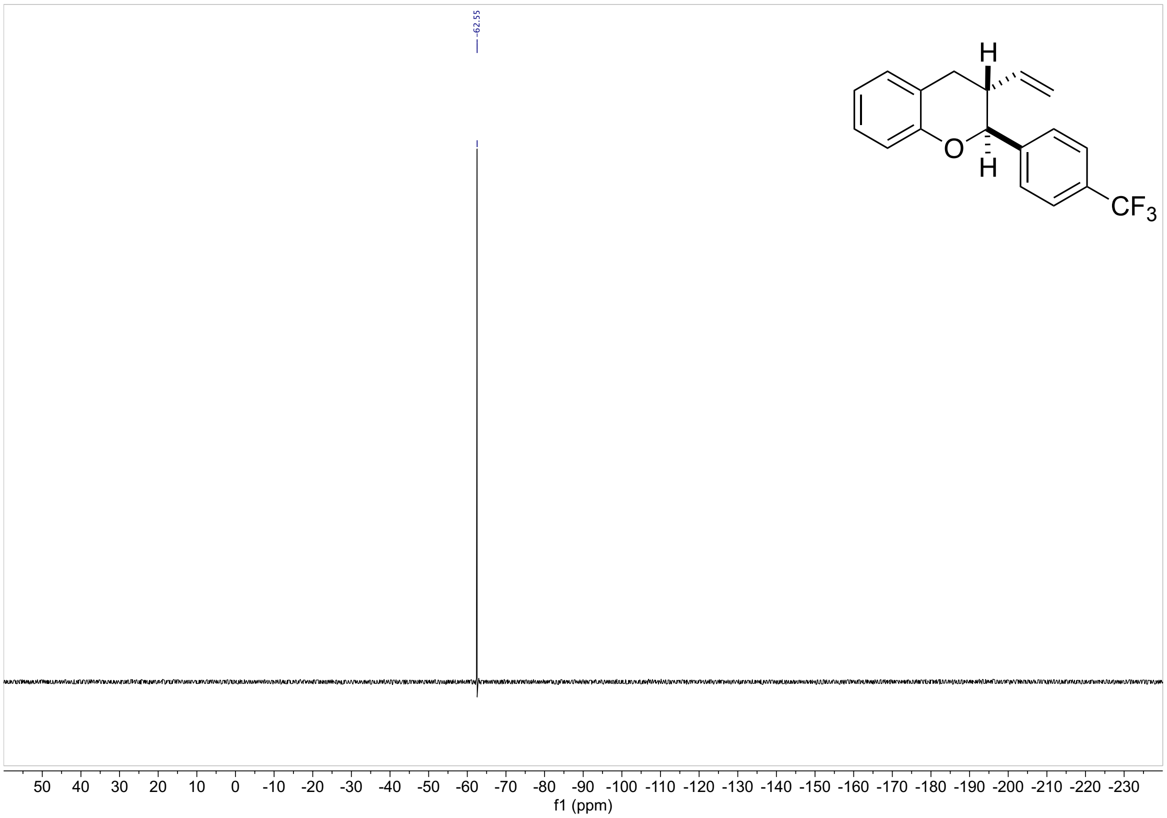

Compound 2q ^{1}H NMR (500 MHz, $CDCl_3$, 298 K)

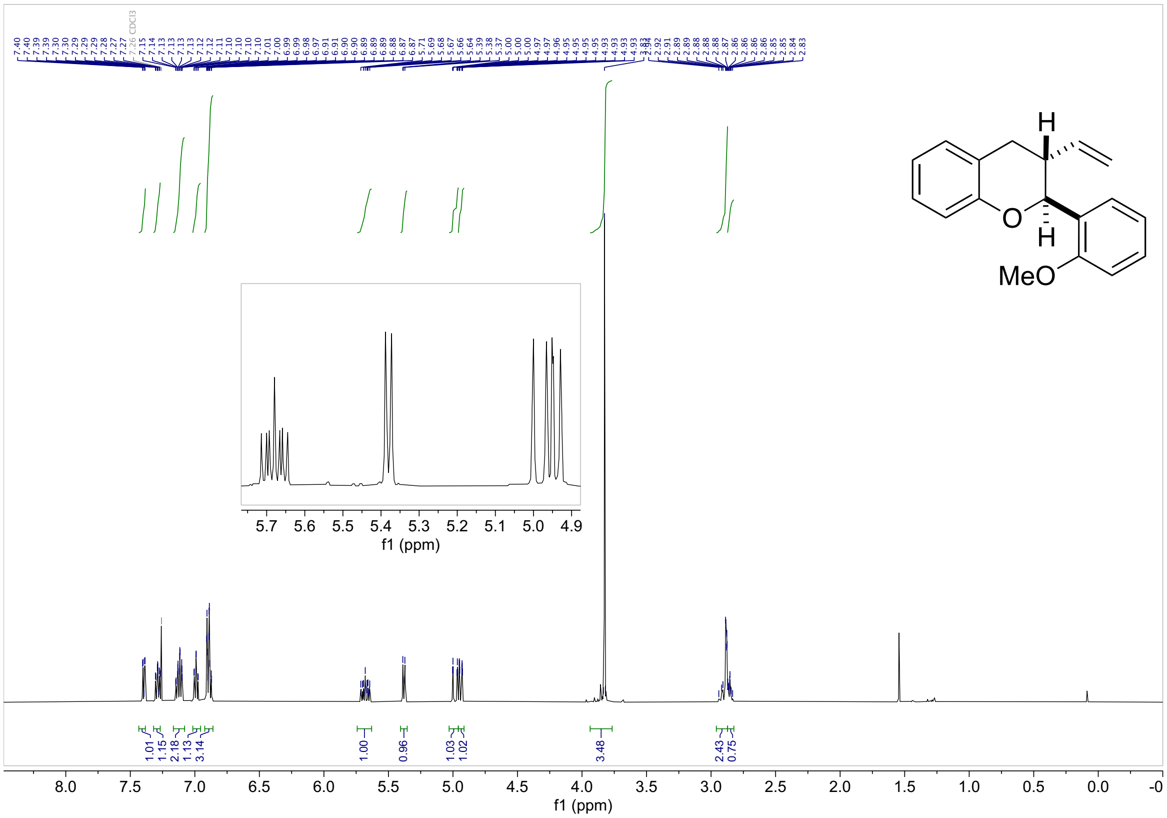

Compound 2q ^{13}C{^{1}H} NMR (126 MHz, $CDCl_3$, 298 K)

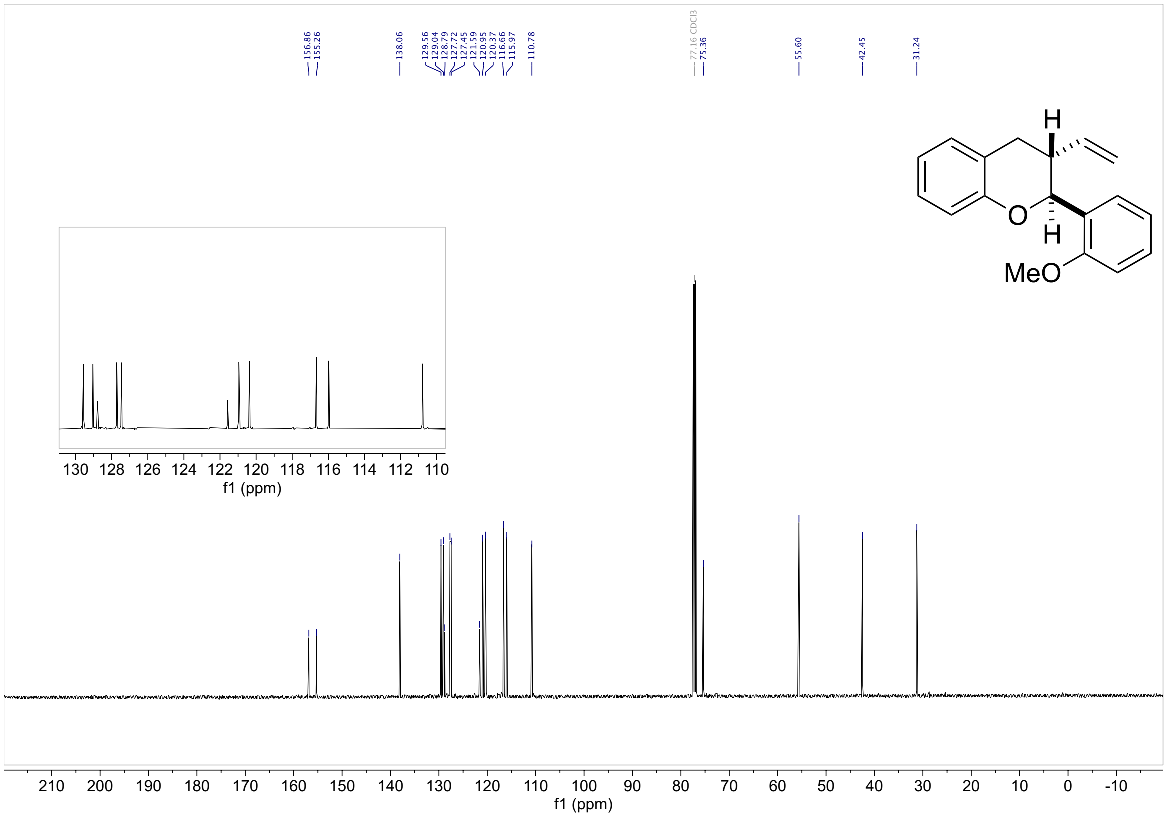

Compound 2r ^{1}H NMR (500 MHz, $CDCl_3$, 298 K)

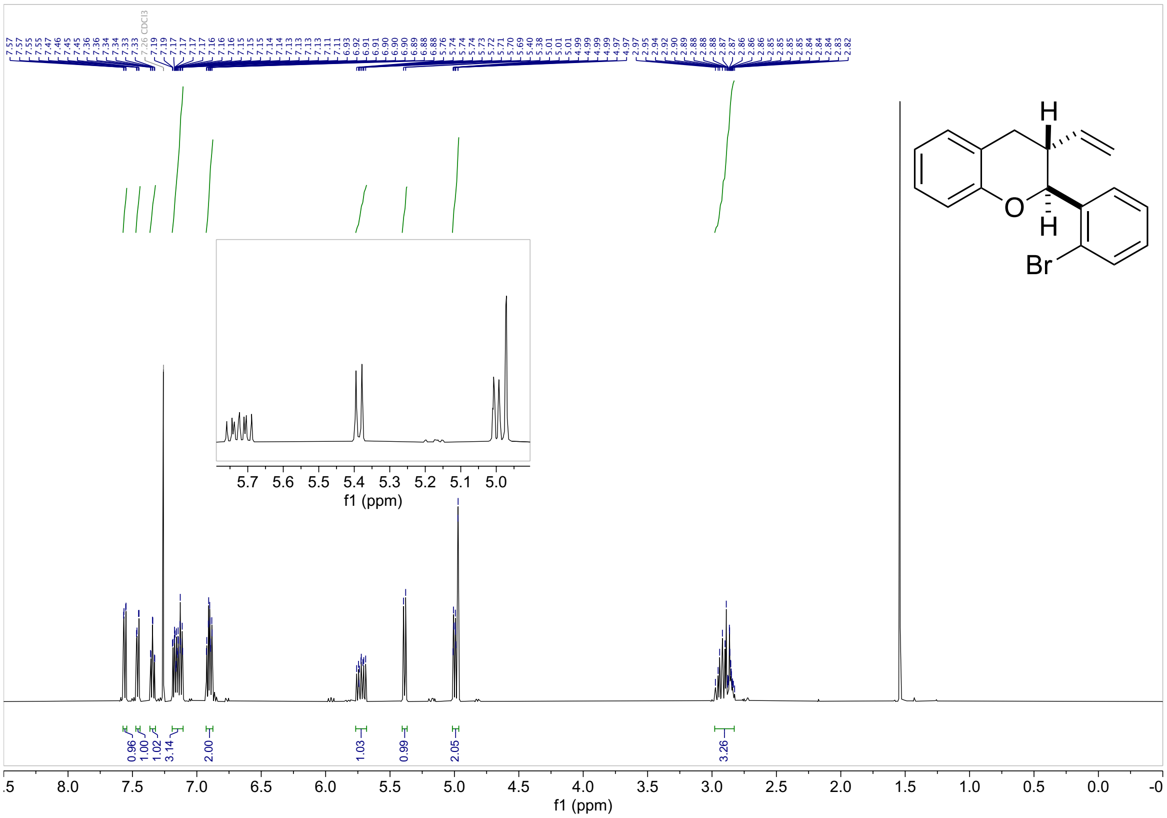

Compound 2r ^{13}C{1H} NMR (126 MHz, $CDCl_3$, 298 K)

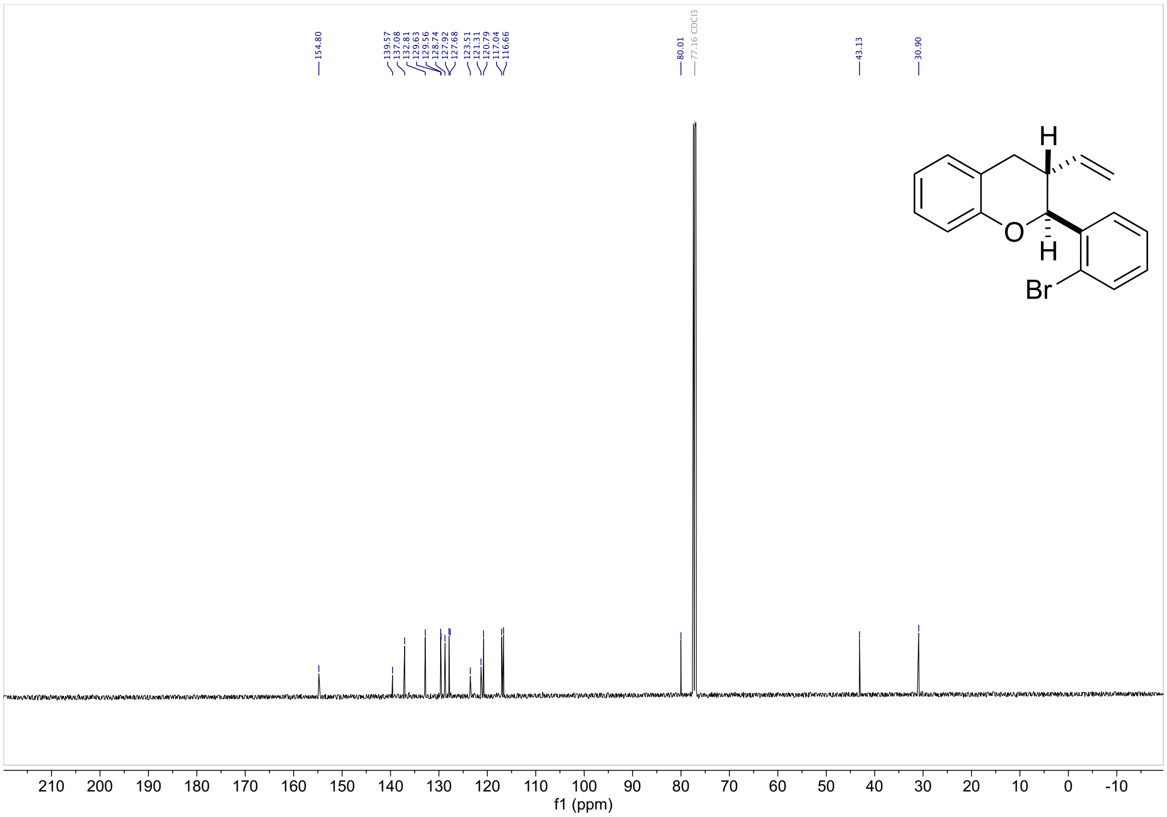

Compound 2s ^{1}H NMR (500 MHz, $CDCl_3$, 298 K)

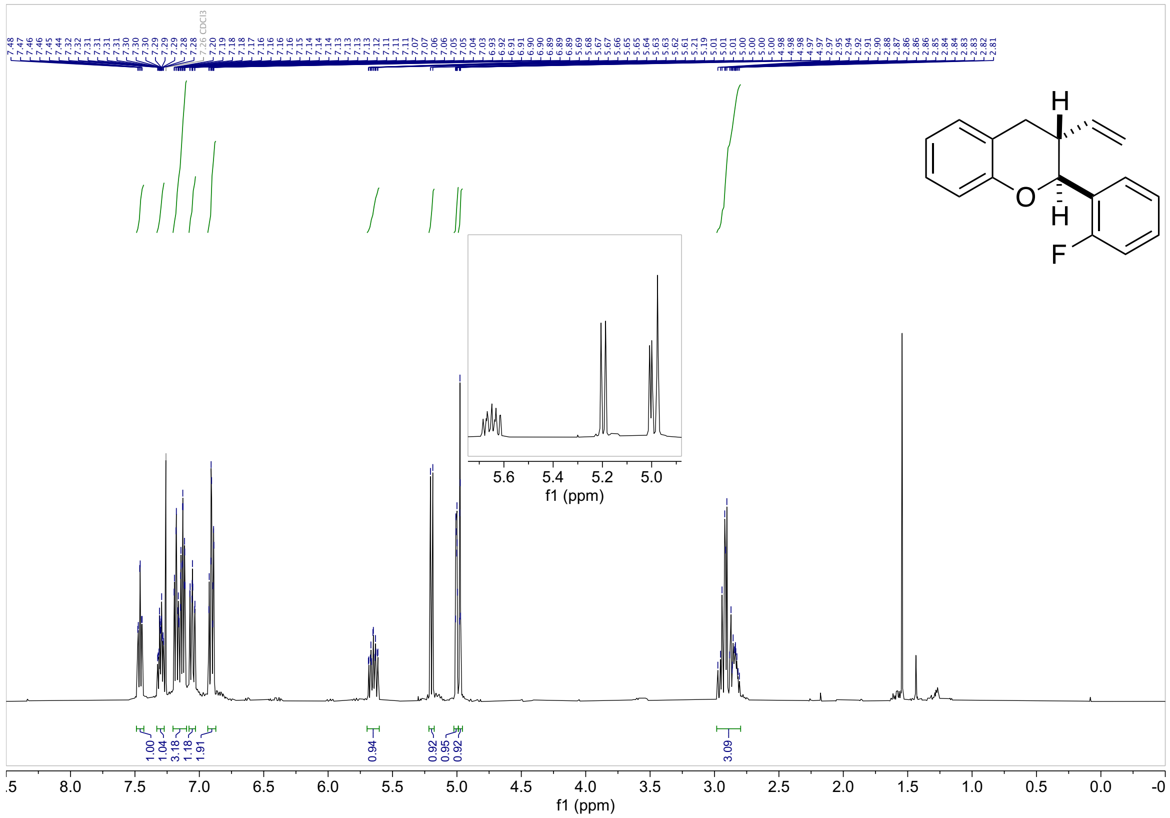

Compound 2s ^{13}C{1H} NMR (126 MHz, $CDCl_3$, 298 K)

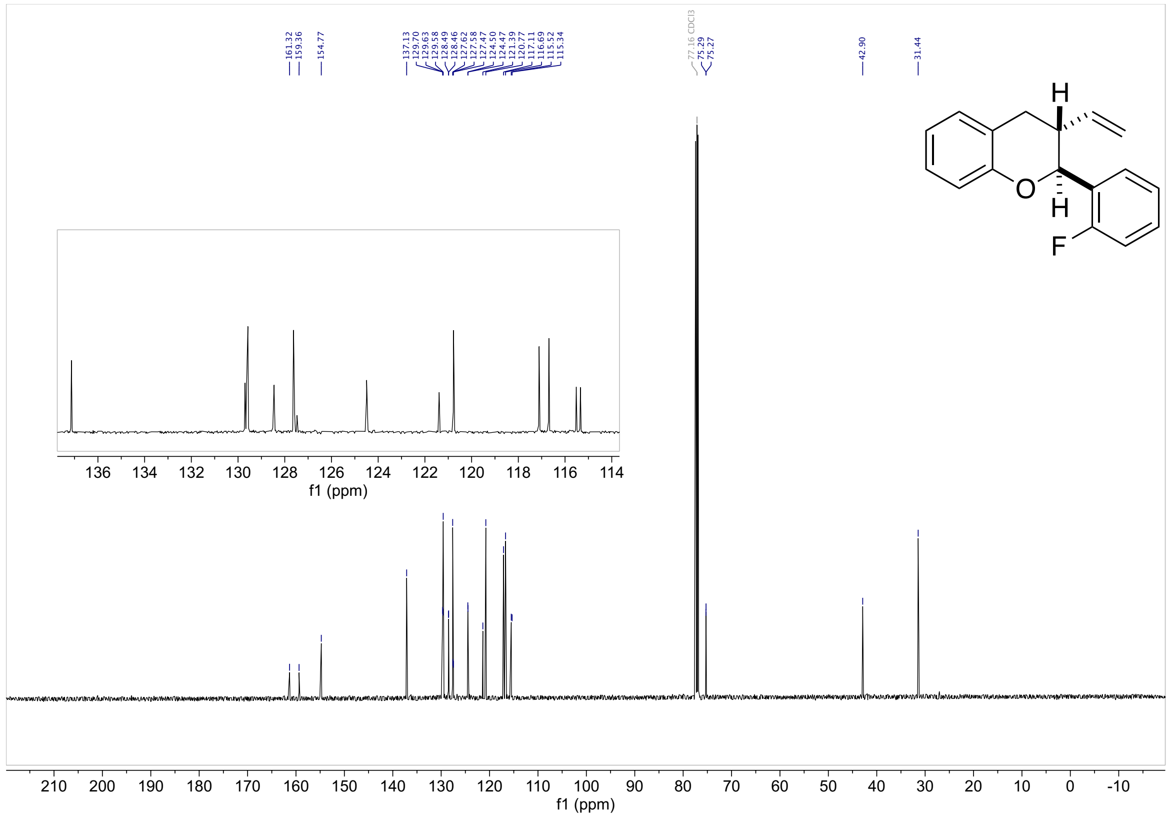

Compound 2s $^{19}F\{1H\}$ NMR (471 MHz, $CDCl_3$, 298 K)

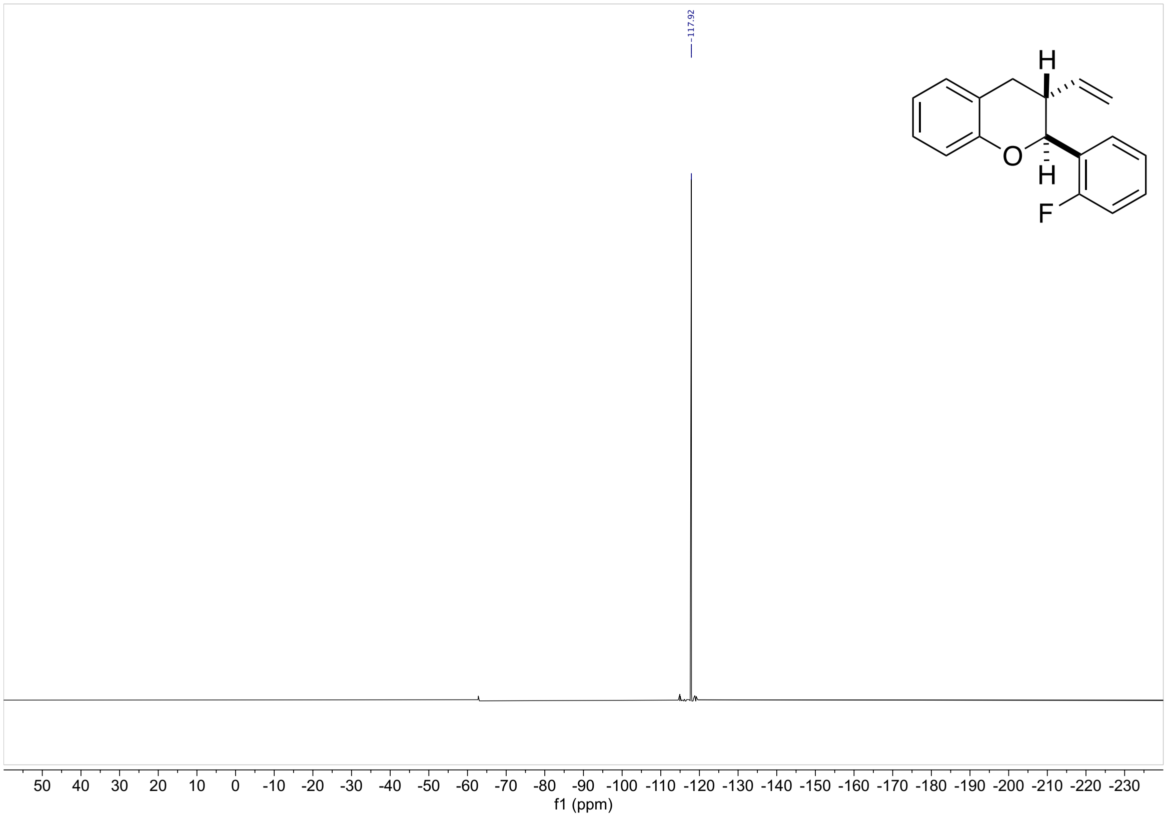

Compound 2t ^{1}H NMR (500 MHz, $CDCl_3$, 298 K)

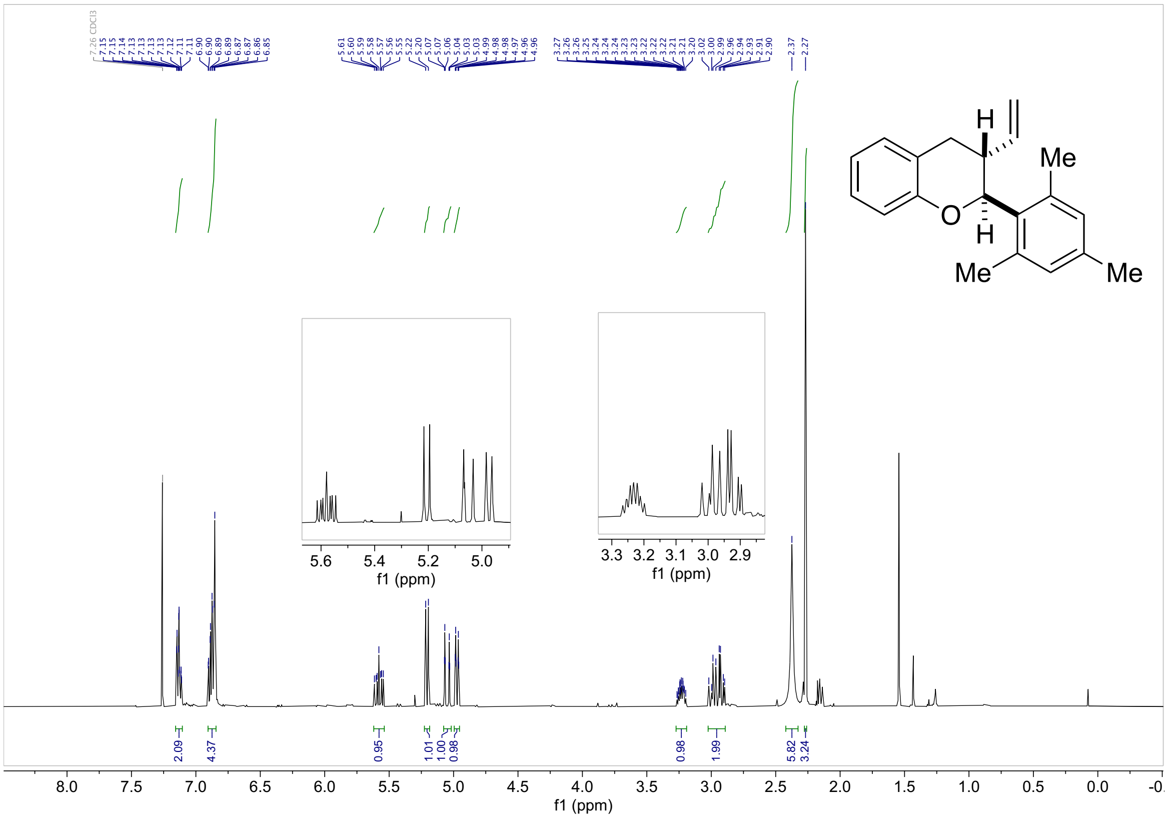

Compound 2t ^{13}C{1H} NMR (126 MHz, $CDCl_3$, 298 K)

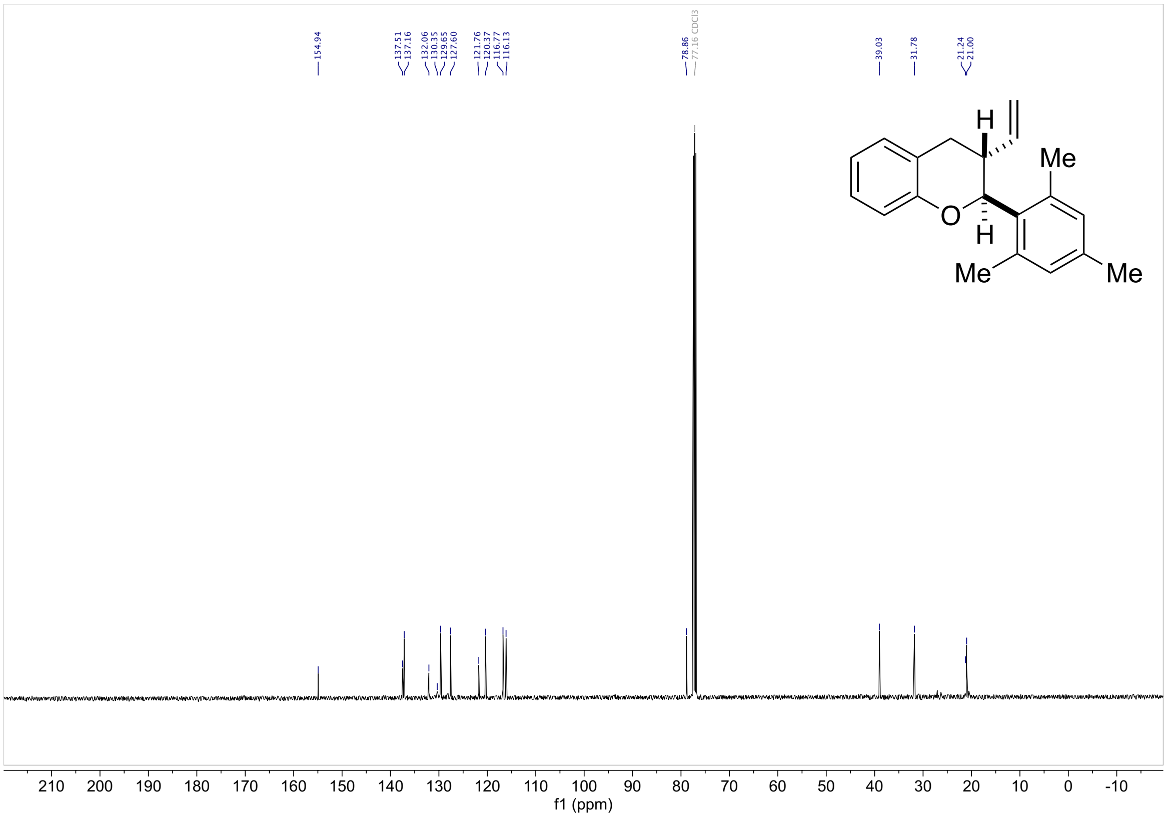

Compound 2u ^{1}H NMR (400 MHz, $CDCl_3$, 298 K)

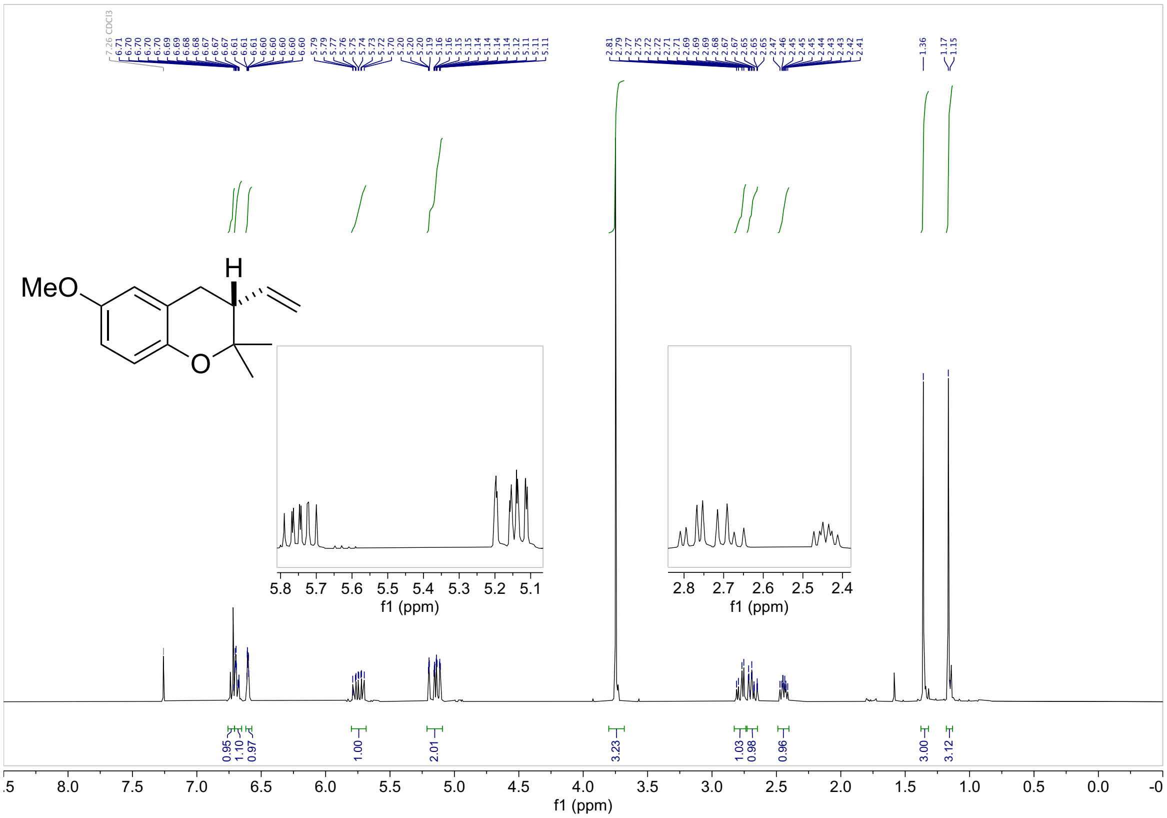

Compound 2u ^{13}C{1H} NMR (101 MHz, $CDCl_3$, 298 K)

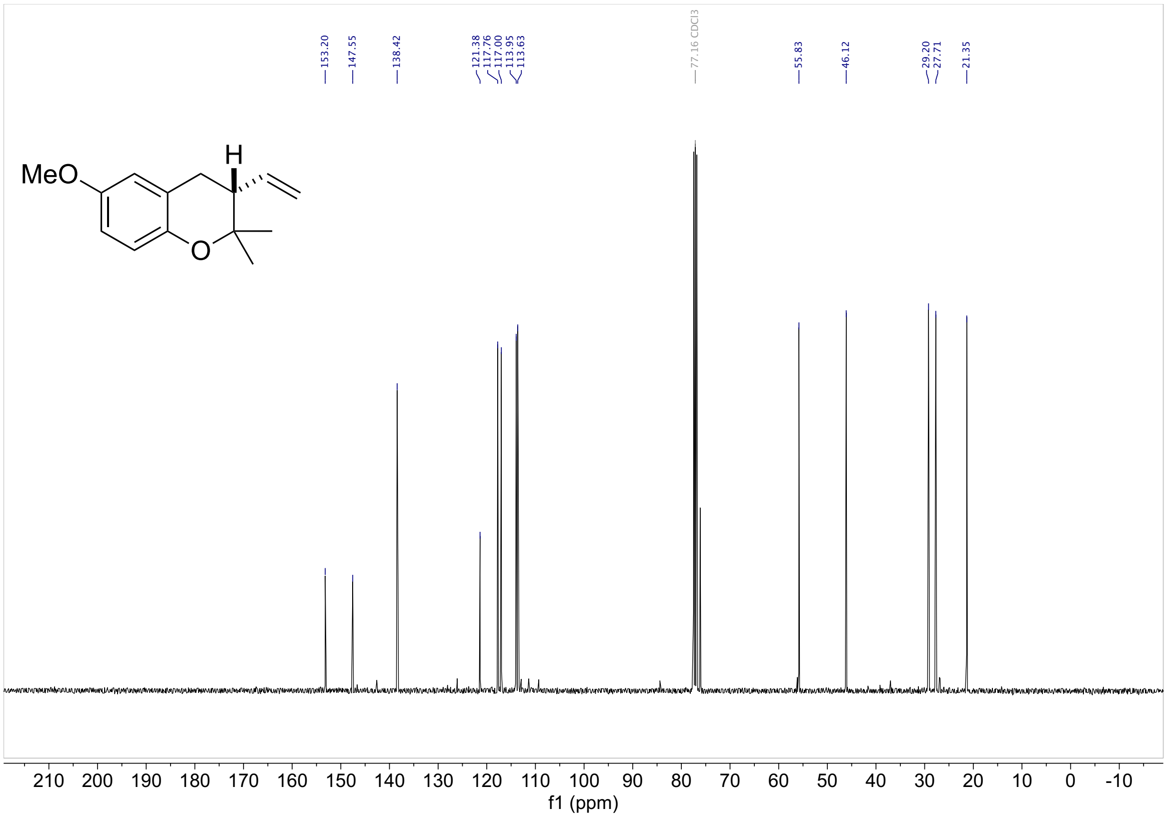

Compound 2v ¹H NMR (500 MHz, CDCl₃, 298 K)

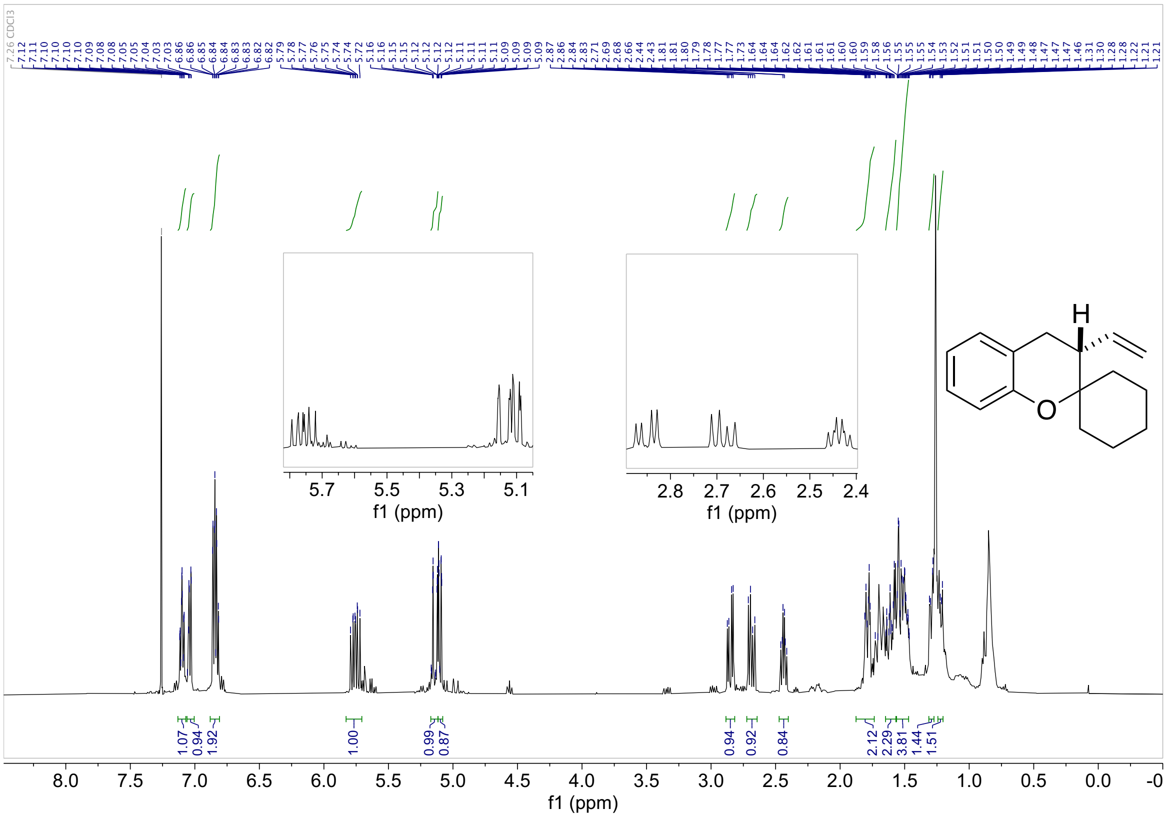

Compound 2v ¹³C{1H} NMR (126 MHz, CDCl₃, 298 K)

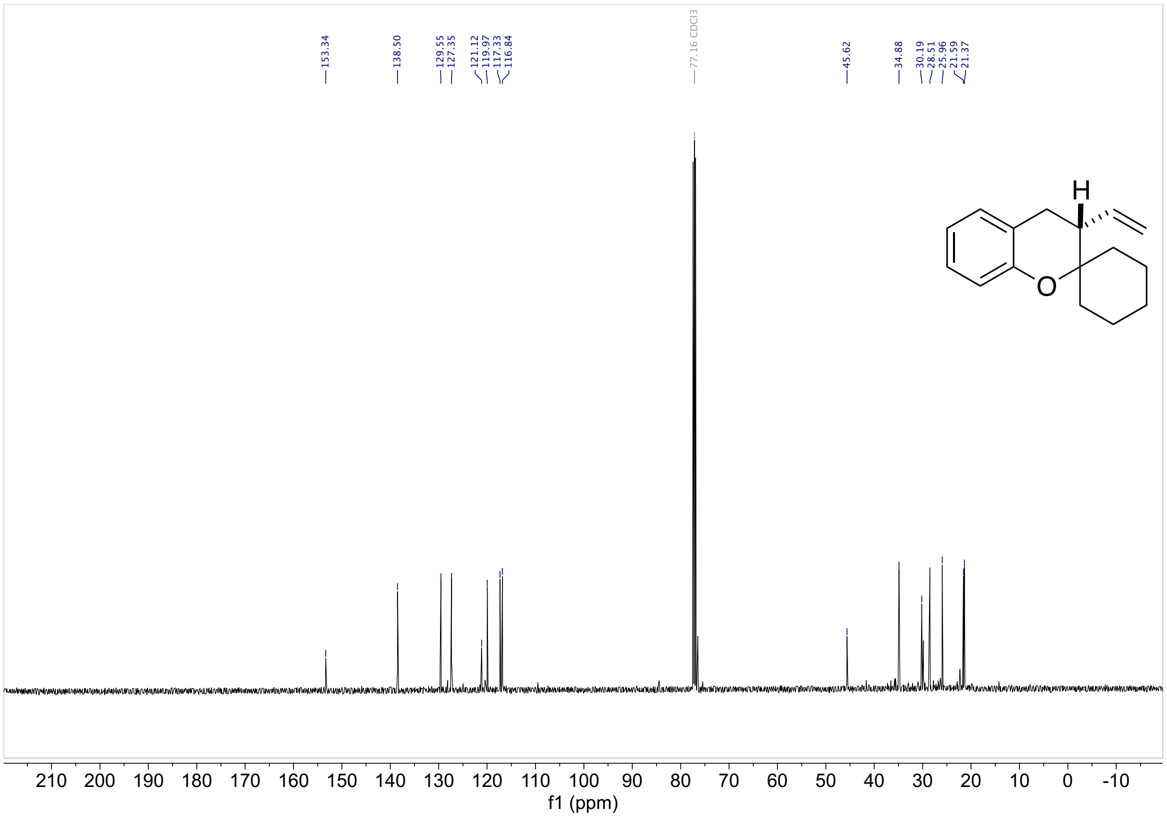

Compound 2w ^{1}H NMR (500 MHz, $CDCl_3$, 298 K)

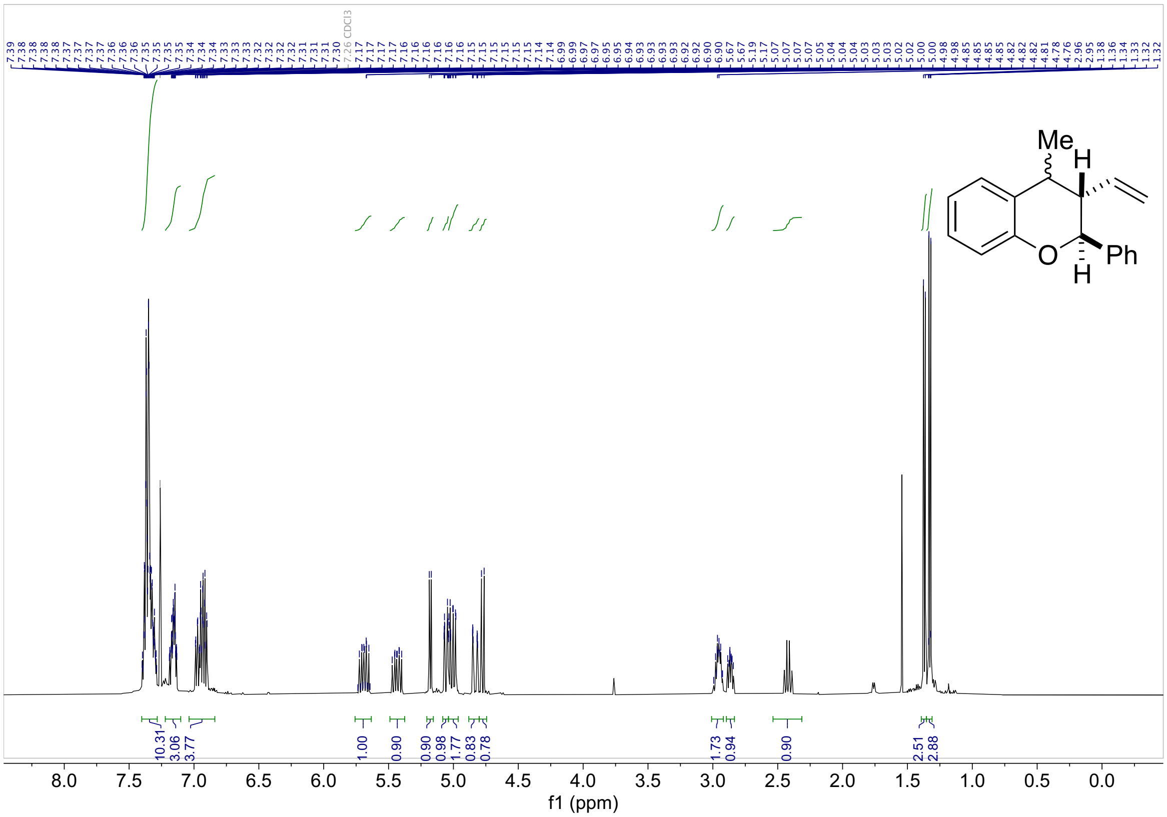

Compound 2w $^{13}C\{1H\}$ NMR (126 MHz, $CDCl_3$, 298 K)

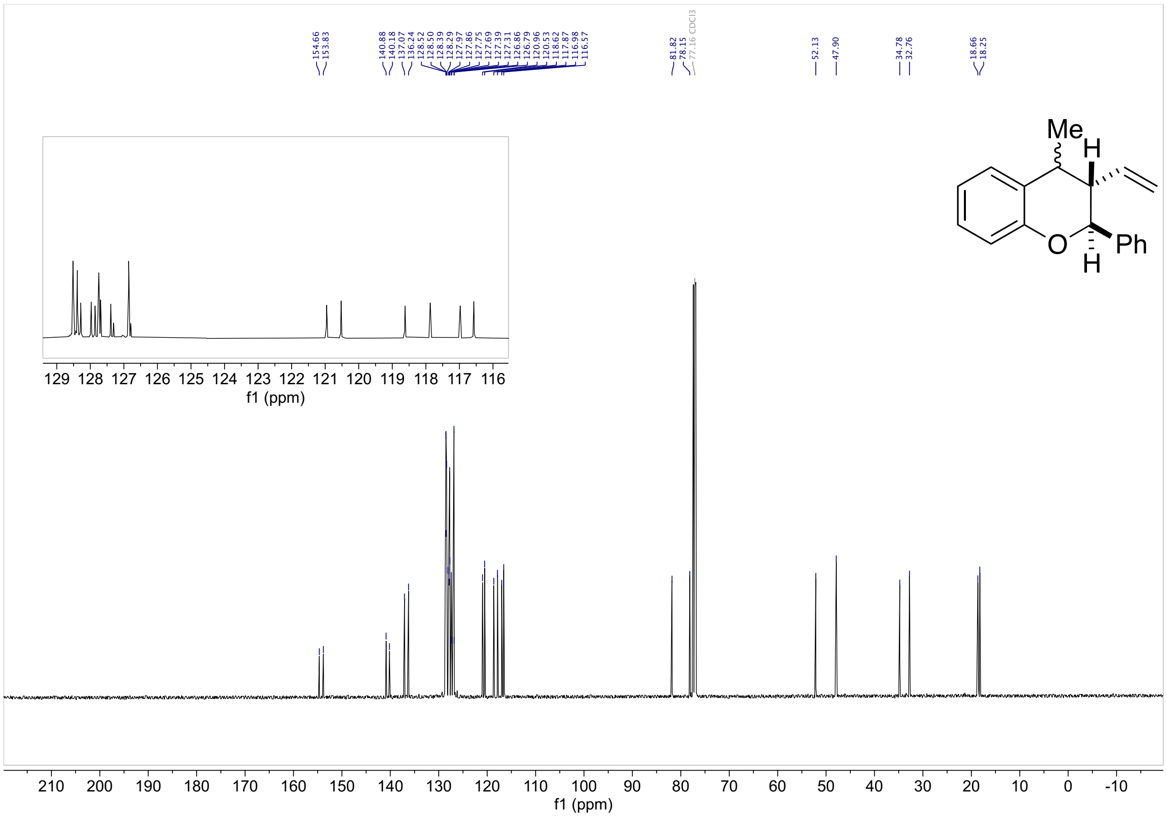

5.3 Suzuki coupling of compound 2d

Compound 2x ^{1}H NMR (500 MHz, $CDCl_3$, 298 K)

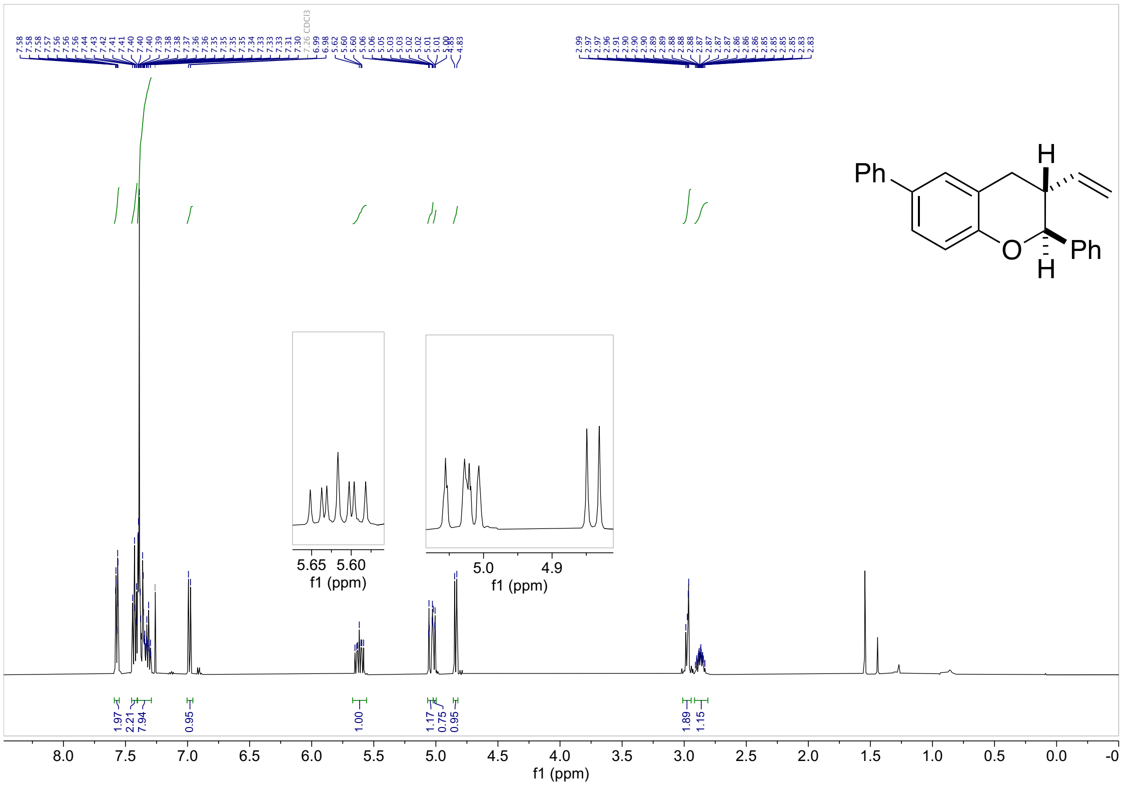

Compound 2x $^{13}C\{1H\}$ NMR (126 MHz, $CDCl_3$, 298 K)

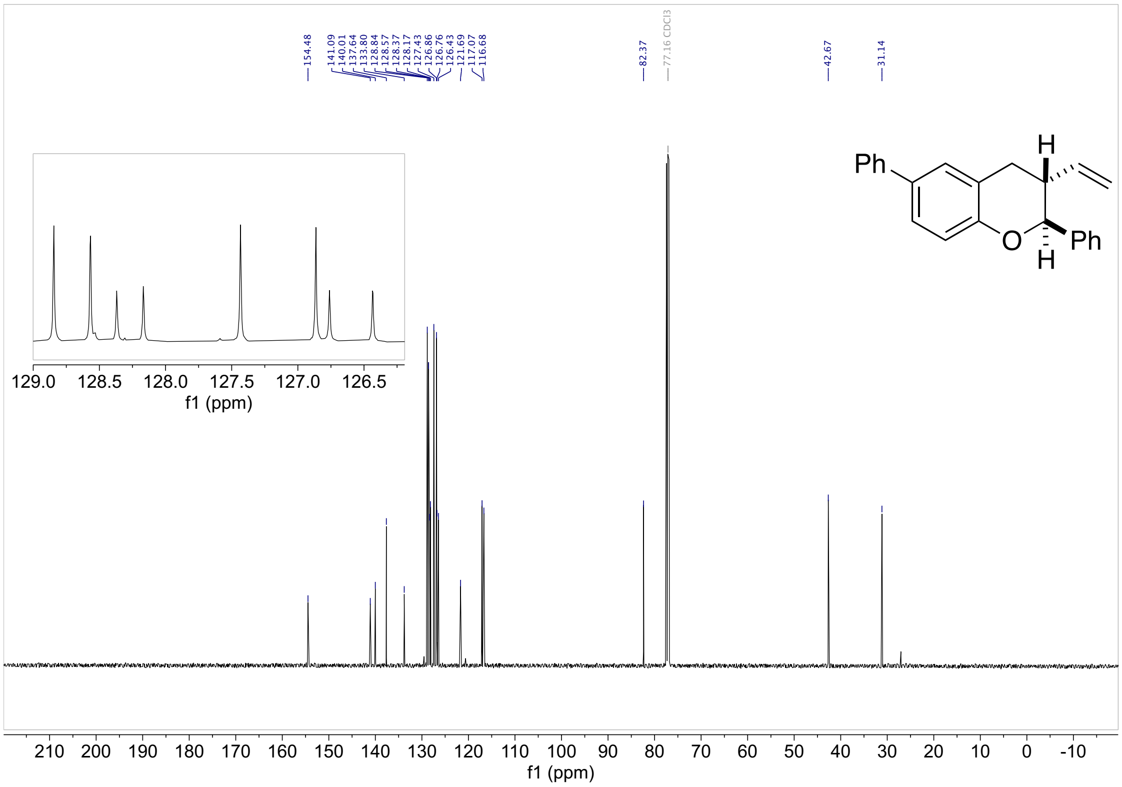

5.4 Late stage functionalization of natural product lapachol

Compound 4 ^{1}H NMR (400 MHz, $CDCl_3$, 298 K)

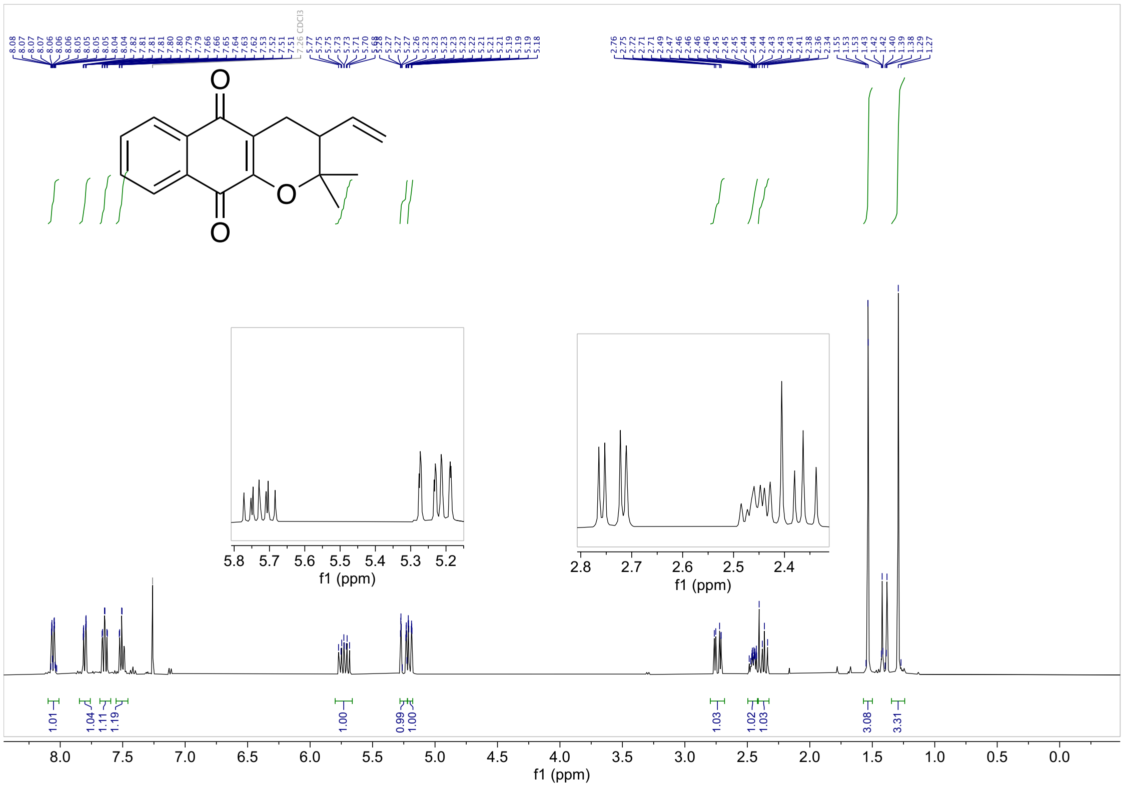

Compound 4 $^{13}C\{1H\}$ NMR (101 MHz, $CDCl_3$, 298 K)

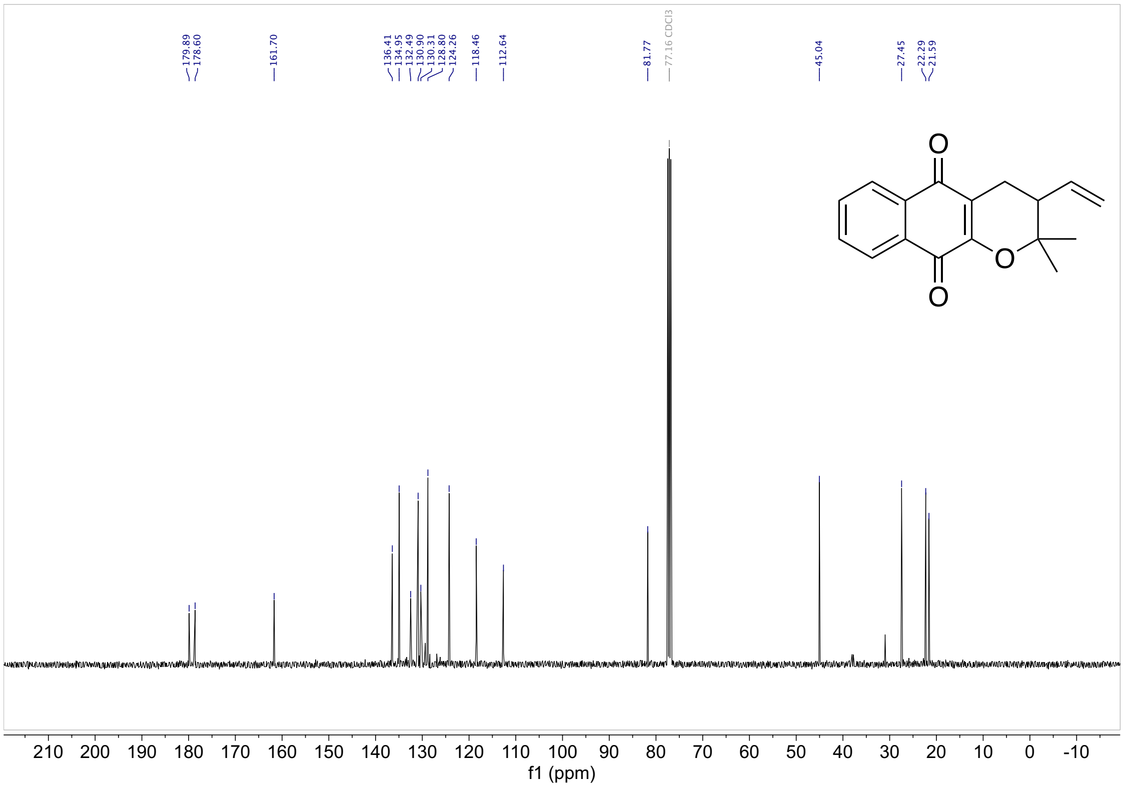

5.5 Diversfication of the product 2a

Compound 5 ^{1}H NMR (400 MHz, $CDCl_3$, 298 K)

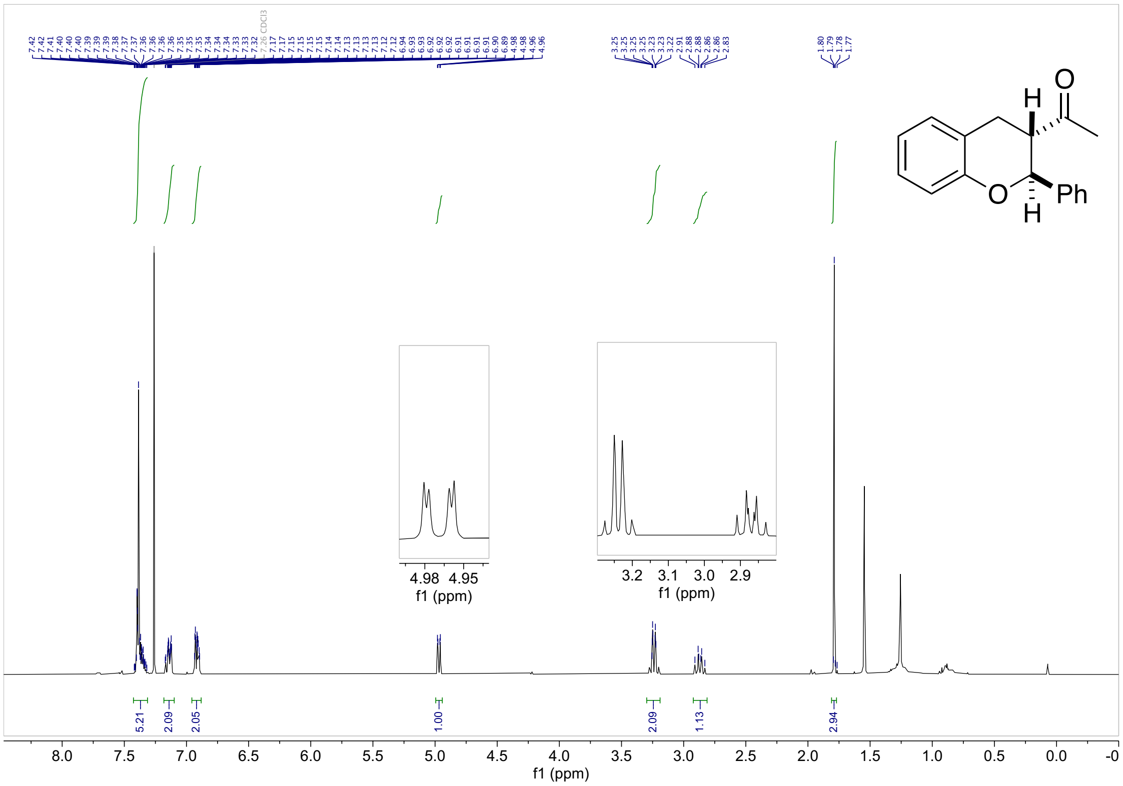

Compound 5 ^{13}C{1H} NMR (101 MHz, $CDCl_3$, 298 K)

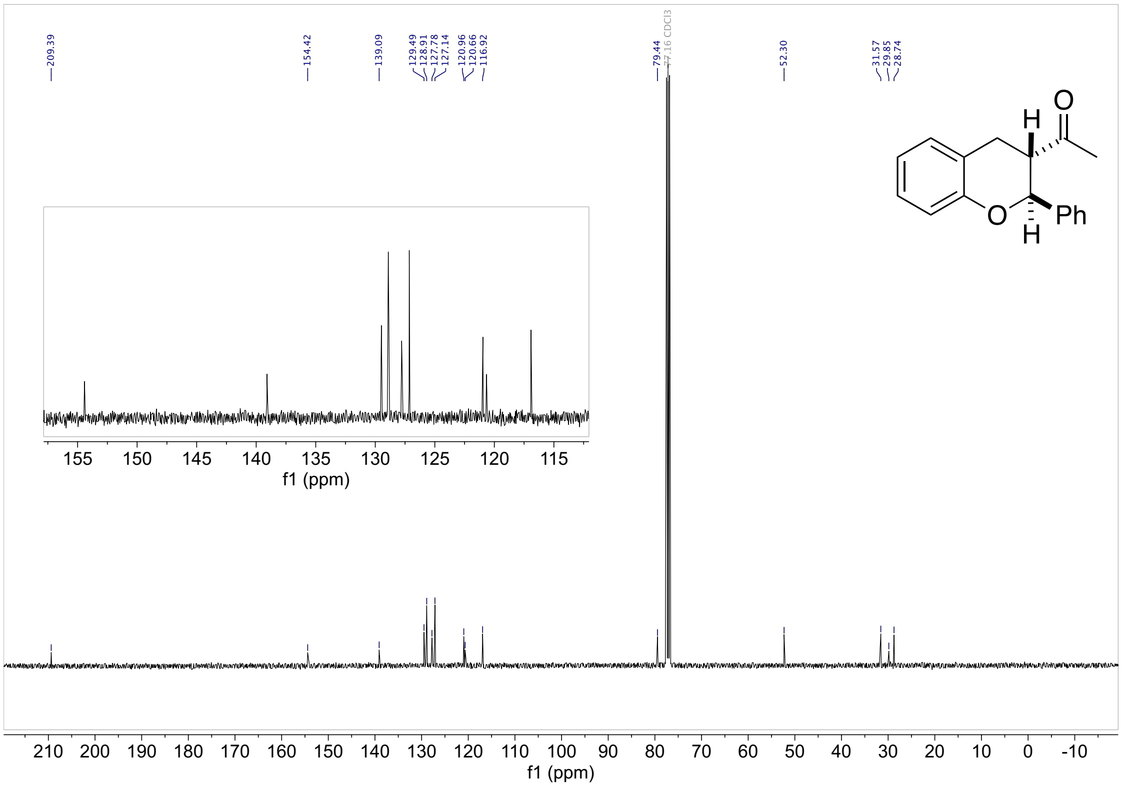

Compound 6 ^{1}H NMR (400 MHz, $CDCl_3$, 298 K)

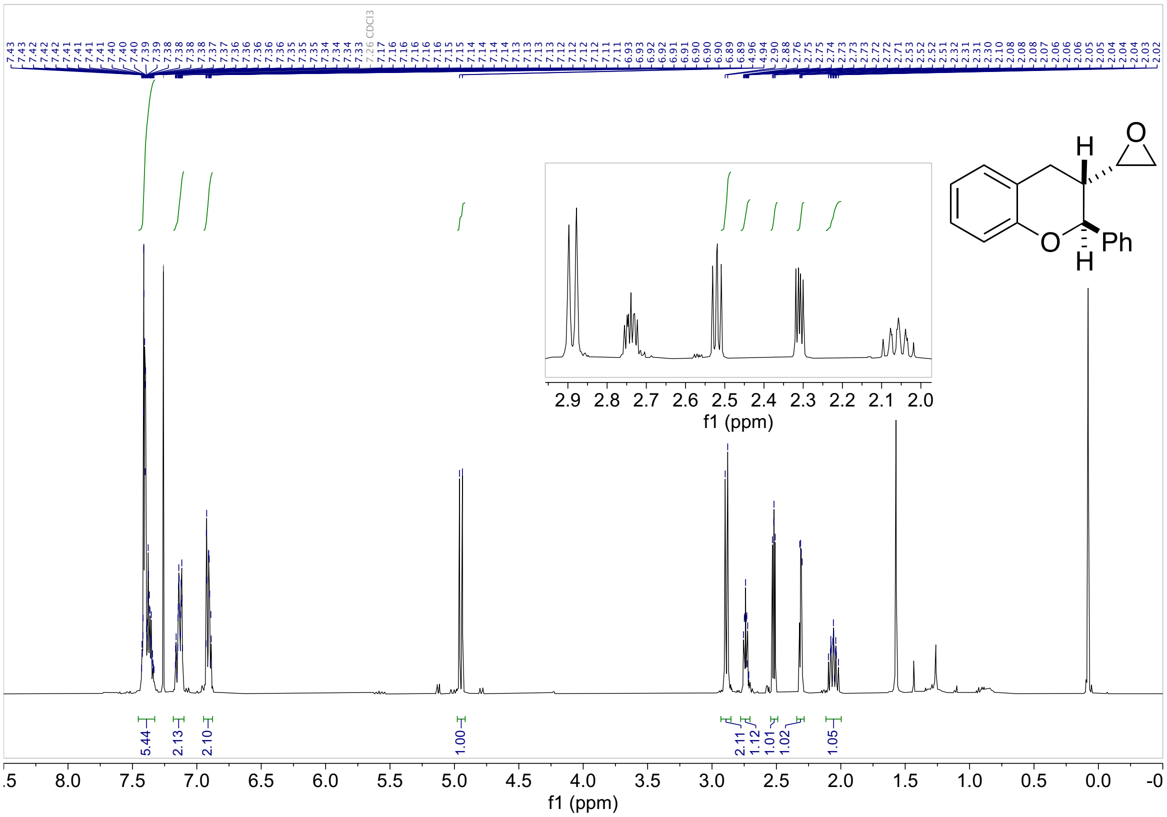

Compound 6 ^{13}C{1H} NMR (126 MHz, $CDCl_3$, 298 K)

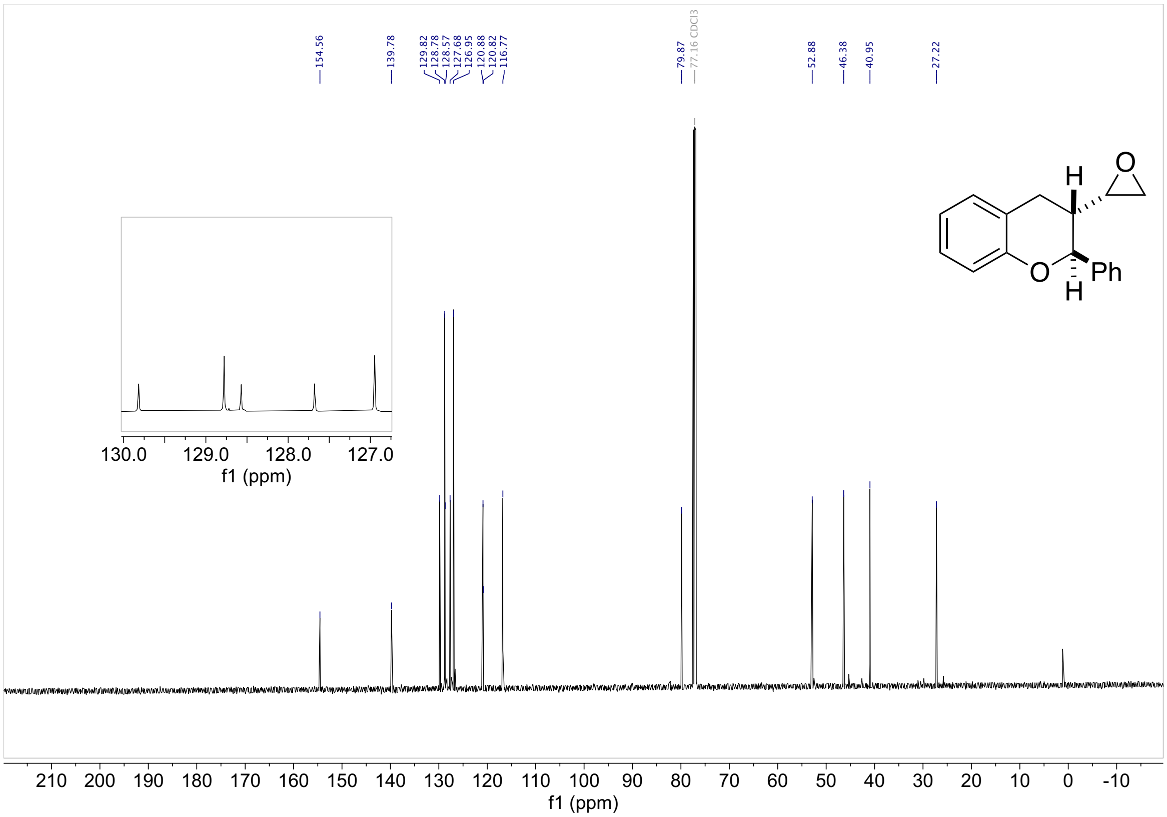

Compound 7 ^{1}H NMR (500 MHz, $CDCl_3$, 298 K)

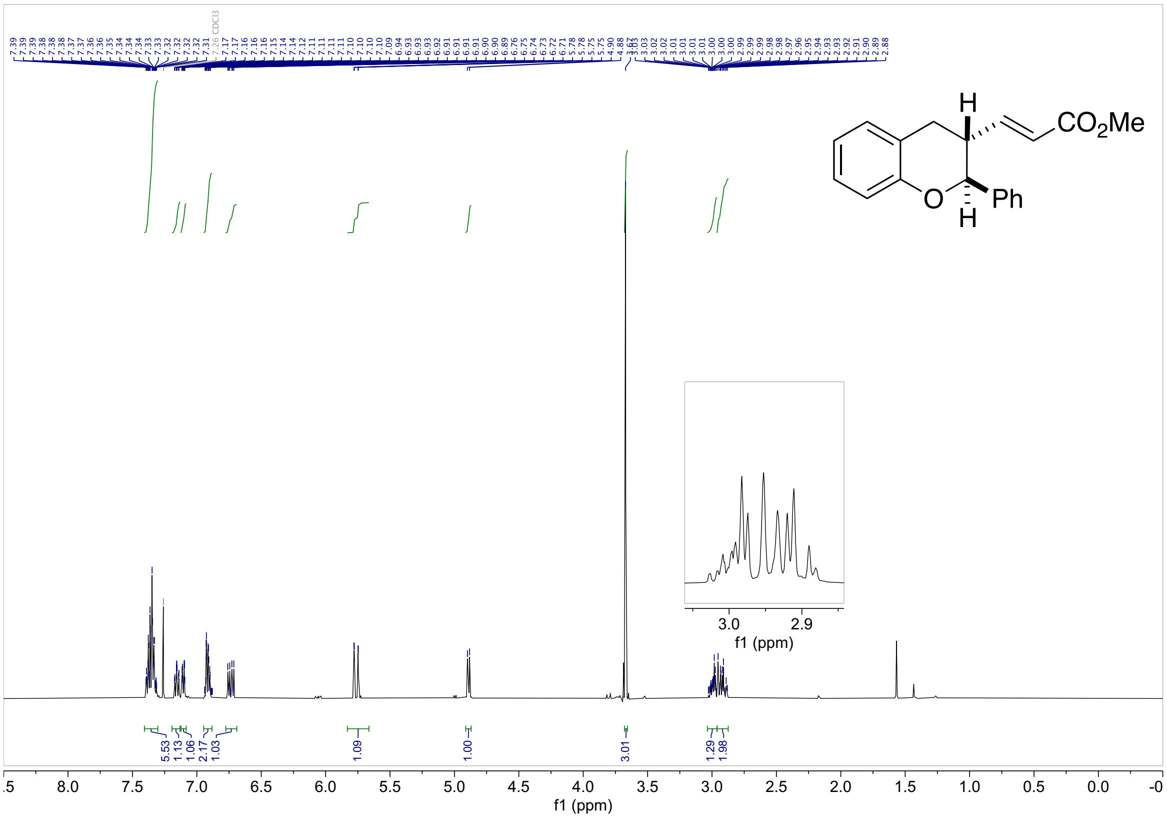

Compound 7 ^{13}C{1H} NMR (126 MHz, $CDCl_3$, 298 K)

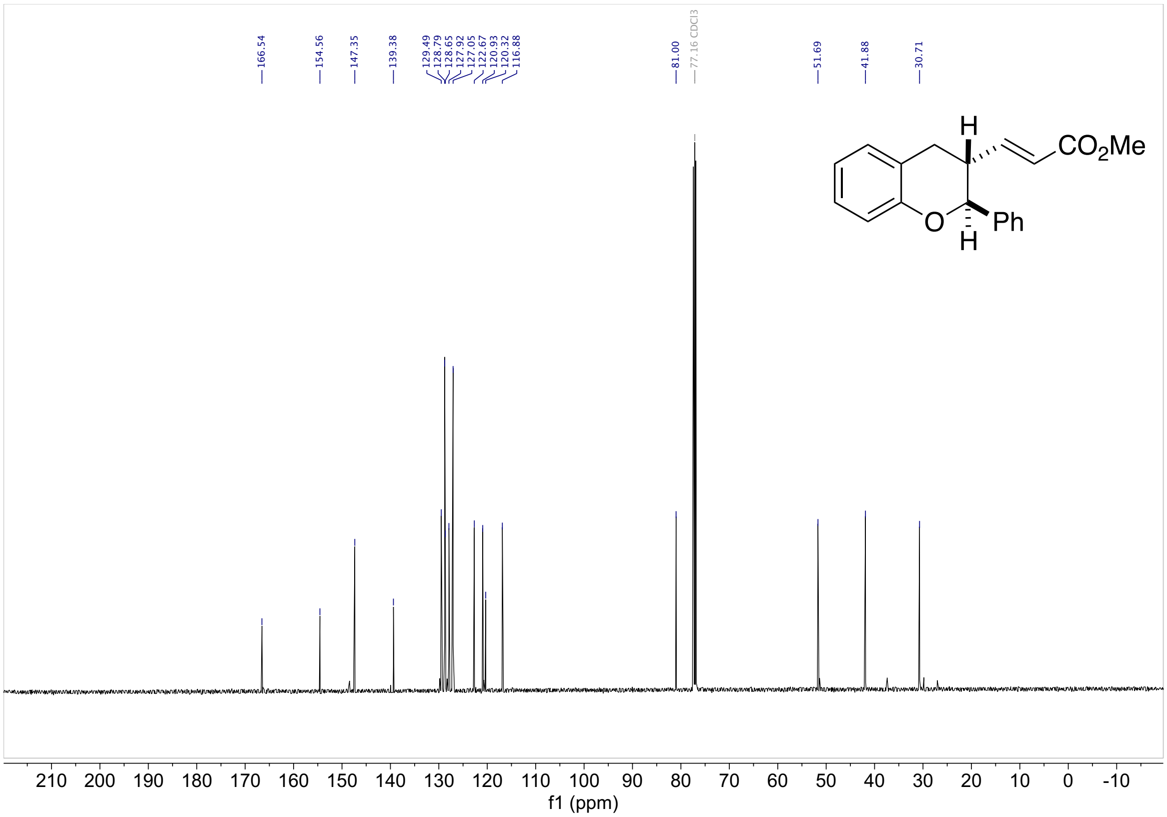

Compound 8 ^{1}H NMR (400 MHz, $CDCl_3$, 298 K)

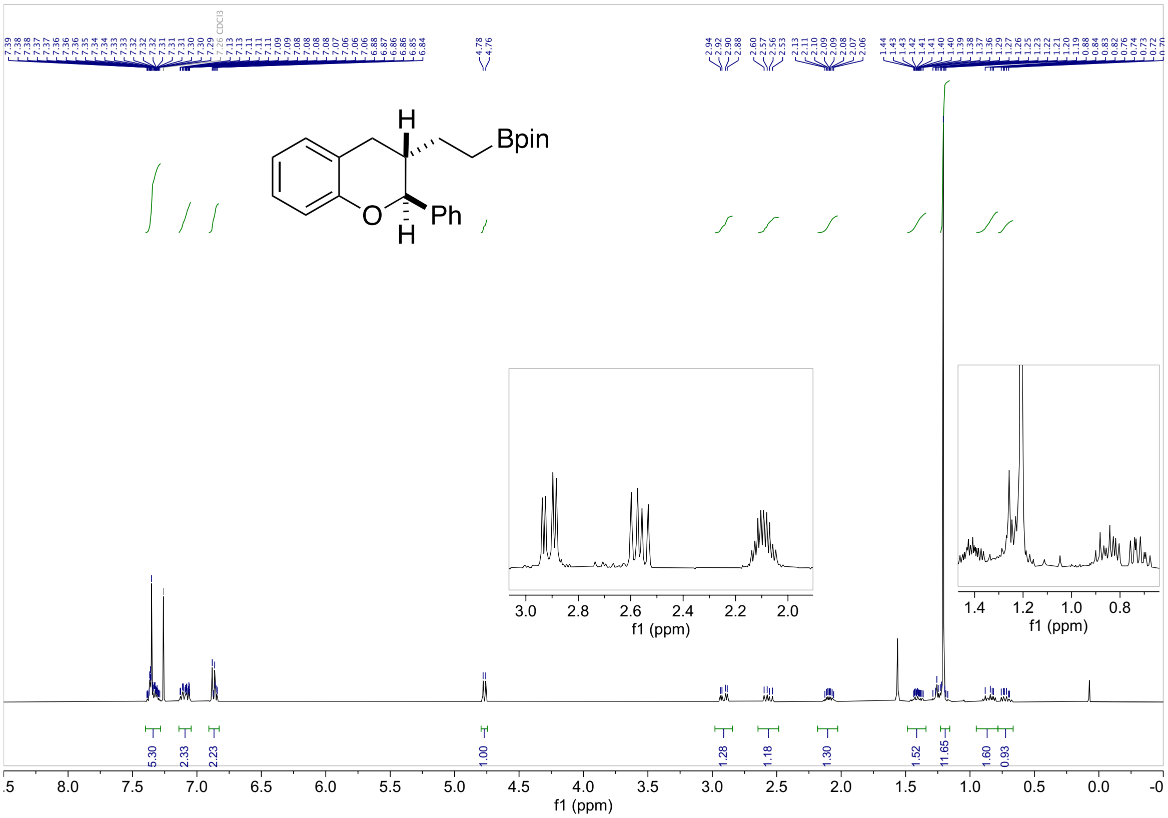

Compound 8 ^{13}C{1H} NMR (126 MHz, $CDCl_3$, 298 K)

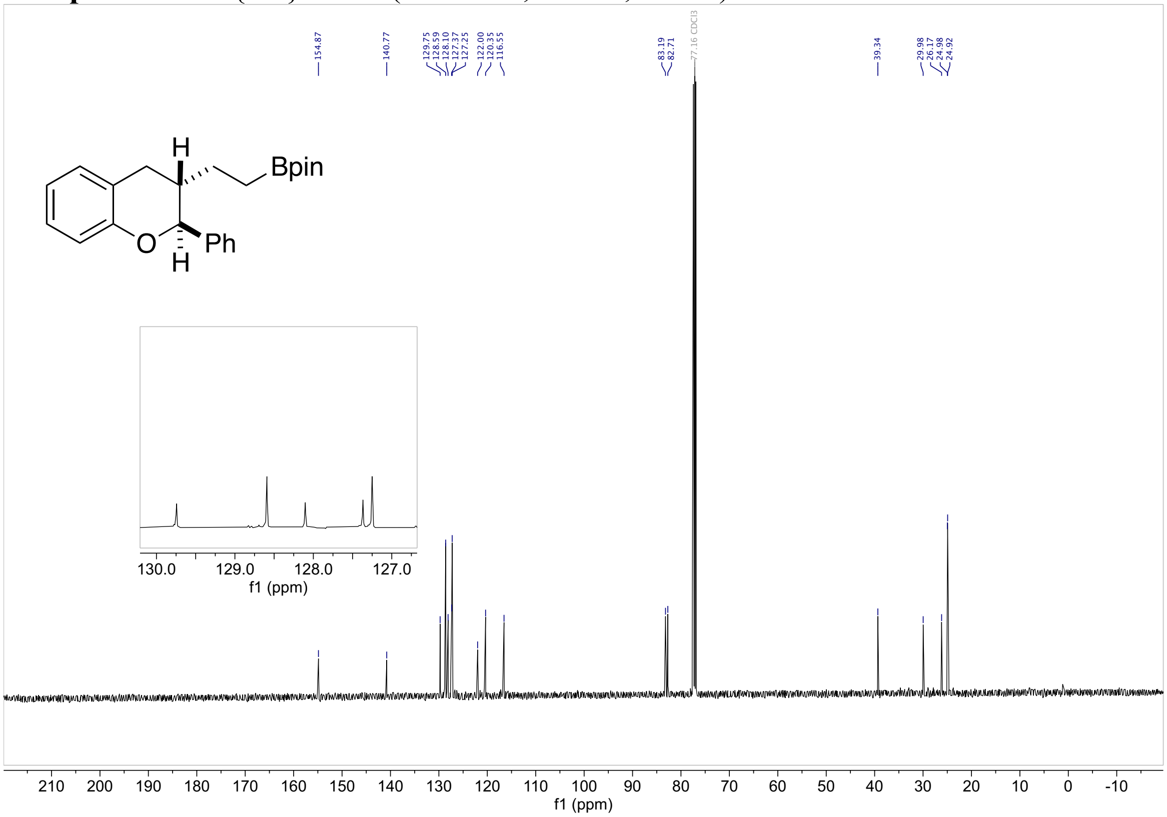

5.6 Chiral Catalyst E

^{1}H NMR (500 MHz, CD_2Cl_2, 298 K)

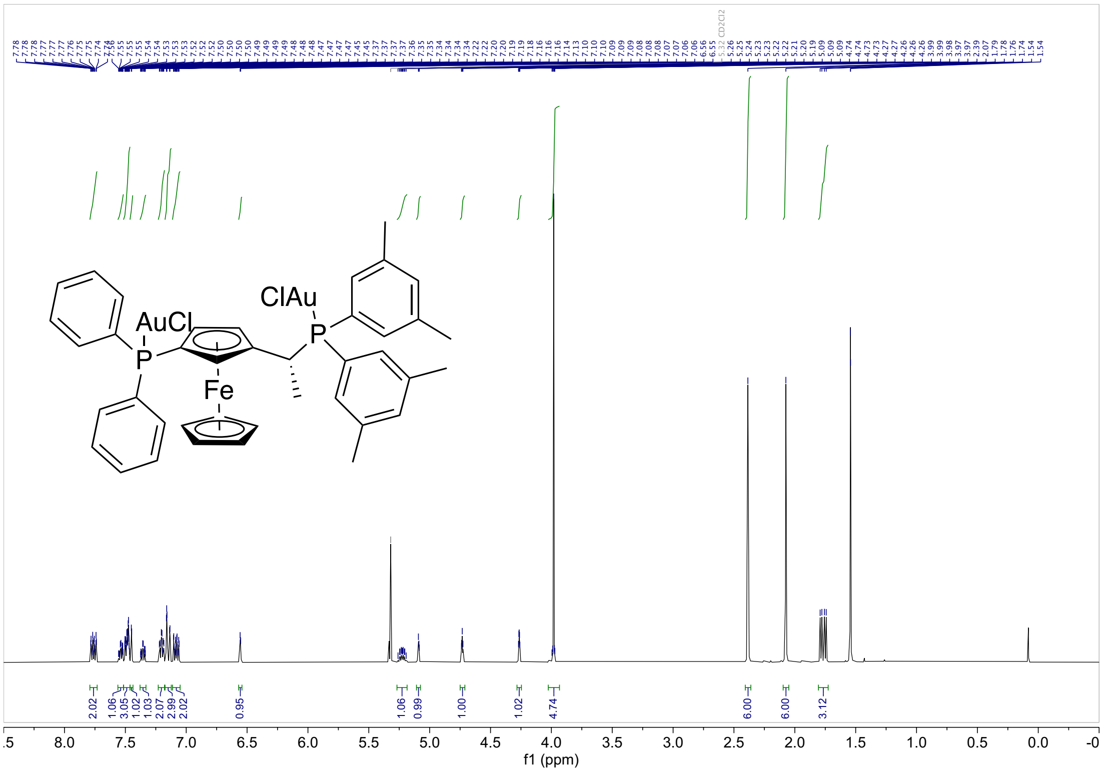

^{13}C {1H} NMR (126 MHz, CD_2Cl_2, 298 K)

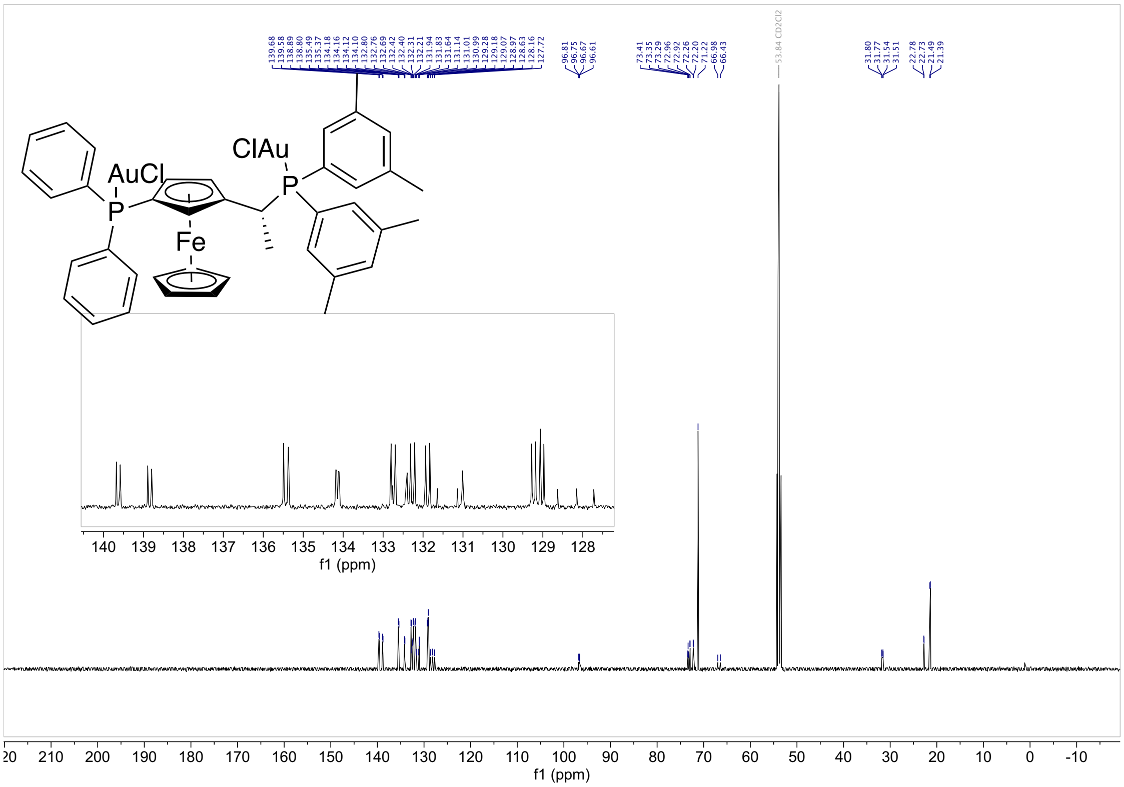

^{31}P {1H} NMR (202 MHz, CD_2Cl_2, 298 K)

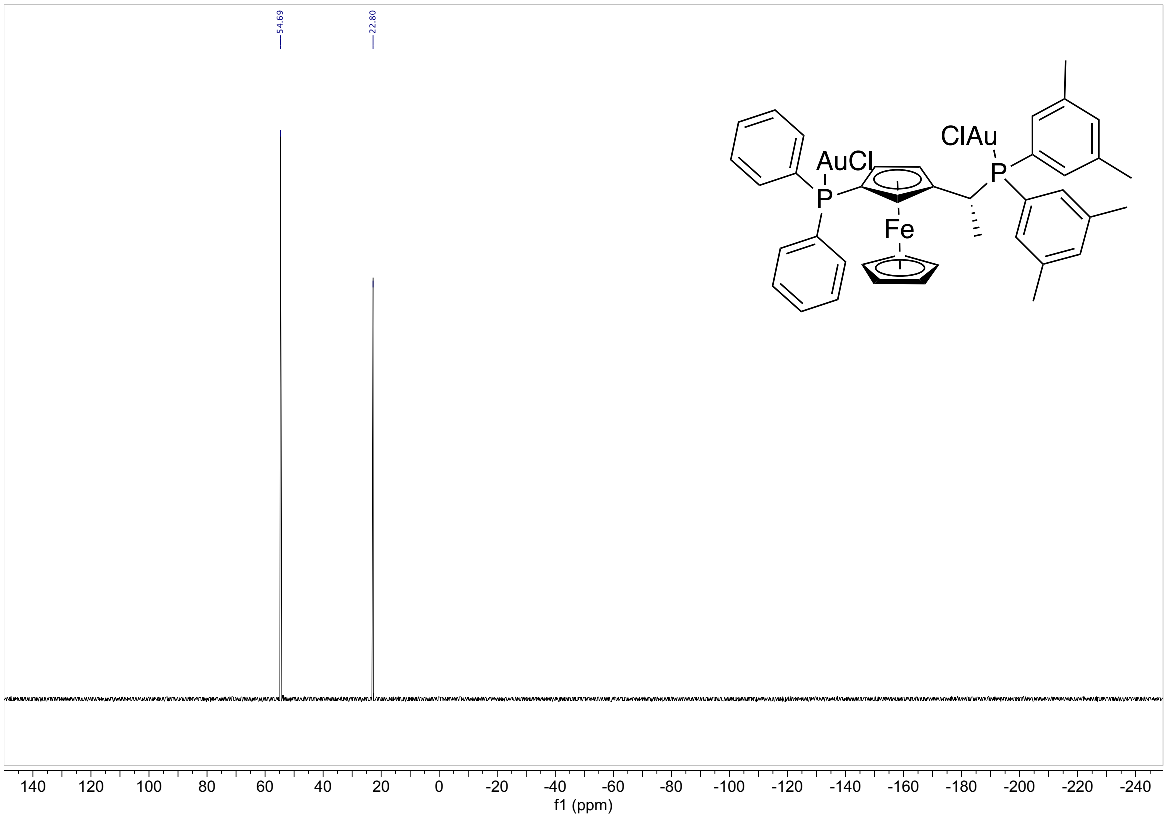

6. X-Ray structures

The supplementary crystallographic data for this paper can be obtained free of charge from The Cambridge Crystallographic Data Centre via www.ccdc.cam.ac.uk/structures.

6.1 6-(*tert*-Butyl)-2-phenyl-3-vinylchromane (Product 2b)

CCDC 2266134

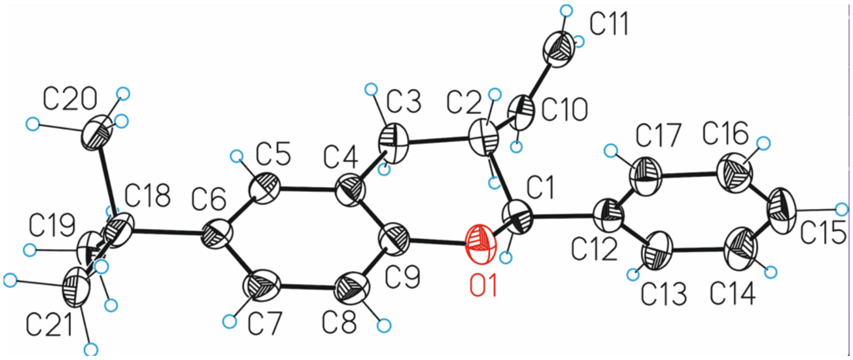

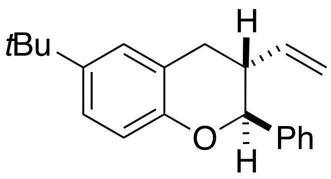

Table S7. Crystal data and structure refinement for TMG-01-840_P2n_J.

Identification code	TMG-01-840_P2n_J	
Empirical formula	C21 H24 O	
Formula weight	292.40	
Temperature	100(2)K	
Wavelength	0.71073 Å	
Crystal system	monoclinic	
Space group	P 2/n	
Unit cell dimensions	a = 14.1122(6)Å	a= 90°.
	b = 5.8601(2)Å	b = 97.726(4)°.
	c = 20.4734(9)Å	g = 90°.
Volume	1677.76(12) $Å^3$	
Z	4	
Density (calculated)	1.158 Mg/m^3	
Absorption coefficient	0.069 mm^{-1}	
F(000)	632	
Crystal size	0.600 x 0.050 x 0.050 mm^3	
Theta range for data collection	3.477 to 28.461°.	
Index ranges	-17<=h<=16,-7<=k<=7,-23<=l<=27	

Reflections collected	17284
Independent reflections	3712[R(int) = 0.0580]
Completeness to theta =28.461°	87.5%
Absorption correction	Multi-scan
Max. and min. transmission	1.00 and 0.42
Refinement method	Full-matrix least-squares on F^2
Data / restraints / parameters	3712/ 330/ 315
Goodness-of-fit on F^2	1.014
Final R indices [I>2sigma(I)]	R1 = 0.0515, wR2 = 0.1221
R indices (all data)	R1 = 0.0915, wR2 = 0.1359
Largest diff. peak and hole	0.191 and -0.202 e.Å^{-3}

Table S8. Bond lengths [Å] and angles [°] for TMG-01-840_P2n_J.

Bond lengths----

O1	C9	1.3804(17)
O1	C1	1.4397(17)
O1	C1'	1.457(13)
C2	C10'	1.439(19)
C2	C1'	1.441(13)
C2	C10	1.492(2)
C2	C1	1.520(2)
C2	C3	1.523(2)
C2	H2	1.0000
C2	H2T	1.0000
C1	C12	1.504(3)
C1	H1	1.0000
C1'	C12'	1.54(3)
C1'	H1'	1.0000
C3	C4	1.5030(19)
C3	H3A	0.9900
C3	H3B	0.9900
C4	C9	1.386(2)
C4	C5	1.392(2)
C5	C6	1.388(2)

C5	H5	0.9500
C6	C7	1.392(2)
C6	C18	1.533(2)
C7	C8	1.378(2)
C7	H7	0.9500
C8	C9	1.385(2)
C8	H8	0.9500
C10	C11	1.306(3)
C10	H10	0.9500
C11	H11A	0.9500
C11	H11B	0.9500
C10'	C11'	1.36(3)
C10'	H10'	0.9500
C11'	H11X	0.9500
C11'	H11Y	0.9500
C12	C17	1.387(3)
C12	C13	1.392(3)
C13	C14	1.387(3)
C13	H13	0.9500
C14	C15	1.393(4)
C14	H14	0.9500
C15	C16	1.395(3)
C15	H15	0.9500
C16	C17	1.390(3)
C16	H16	0.9500
C17	H17	0.9500
C12'	C13'	1.30(3)
C12'	C17'	1.31(3)
C13'	C14'	1.34(3)
C13'	H13'	0.9500
C14'	C15'	1.36(4)
C14'	H14'	0.9500
C15'	C16'	1.33(3)
C15'	H15'	0.9500
C16'	C17'	1.31(3)
C16'	H16'	0.9500
C17'	H17'	0.9500

C18	C19	1.299(3)
C18	C20'	1.383(3)
C18	C20	1.626(3)
C18	C21	1.629(3)
C18	C21'	1.653(3)
C18	C19'	1.660(3)
C19	H19A	0.9800
C19	H19B	0.9800
C19	H19T	0.9800
C20	H20A	0.9800
C20	H20B	0.9800
C20	H20C	0.9800
C21	H21A	0.9800
C21	H21B	0.9800
C21	H21C	0.9800
C19'	H19C	0.9800
C19'	H19D	0.9800
C19'	H19E	0.9800
C20'	H20D	0.9800
C20'	H20E	0.9800
C20'	H20F	0.9800
C21'	H21D	0.9800
C21'	H21E	0.9800
C21'	H21F	0.9800

Angles----------

C9	O1	C1	116.60(11)
C9	O1	C1'	116.8(6)
C10'	C2	C1'	119.4(9)
C10	C2	C1	112.16(13)
C10'	C2	C3	118.6(7)
C1'	C2	C3	120.3(5)
C10	C2	C3	111.41(13)
C1	C2	C3	110.12(12)
C10	C2	H2	107.6
C1	C2	H2	107.6
C3	C2	H2	107.6
C10'	C2	H2T	94.3

C1'	C2	H2T	94.3
C3	C2	H2T	94.3
O1	C1	C12	105.91(13)
O1	C1	C2	112.08(12)
C12	C1	C2	113.75(15)
O1	C1	H1	108.3
C12	C1	H1	108.3
C2	C1	H1	108.3
C2	C1'	O1	115.8(9)
C2	C1'	C12'	120.0(14)
O1	C1'	C12'	108.5(13)
C2	C1'	H1'	103.4
O1	C1'	H1'	103.4
C12'	C1'	H1'	103.4
C4	C3	C2	113.11(12)
C4	C3	H3A	109.0
C2	C3	H3A	109.0
C4	C3	H3B	109.0
C2	C3	H3B	109.0
H3A	C3	H3B	107.8
C9	C4	C5	117.88(13)
C9	C4	C3	120.67(12)
C5	C4	C3	121.46(13)
C6	C5	C4	122.96(15)
C6	C5	H5	118.5
C4	C5	H5	118.5
C5	C6	C7	117.01(14)
C5	C6	C18	121.19(15)
C7	C6	C18	121.79(14)
C8	C7	C6	121.61(14)
C8	C7	H7	119.2
C6	C7	H7	119.2
C7	C8	C9	119.73(15)
C7	C8	H8	120.1
C9	C8	H8	120.1
O1	C9	C8	116.40(13)
O1	C9	C4	122.78(12)

C8	C9	C4	120.81(13)
C11	C10	C2	126.7(2)
C11	C10	H10	116.6
C2	C10	H10	116.6
C10	C11	H11A	120.0
C10	C11	H11B	120.0
H11A	C11	H11B	120.0
C11'	C10'	C2	134(2)
C11'	C10'	H10'	113.0
C2	C10'	H10'	113.0
C10'	C11'	H11X	120.0
C10'	C11'	H11Y	120.0
H11X	C11'	H11Y	120.0
C17	C12	C13	119.4(2)
C17	C12	C1	120.8(2)
C13	C12	C1	119.8(2)
C14	C13	C12	120.4(2)
C14	C13	H13	119.8
C12	C13	H13	119.8
C13	C14	C15	120.0(2)
C13	C14	H14	120.0
C15	C14	H14	120.0
C14	C15	C16	119.8(2)
C14	C15	H15	120.1
C16	C15	H15	120.1
C17	C16	C15	119.7(2)
C17	C16	H16	120.2
C15	C16	H16	120.2
C12	C17	C16	120.7(2)
C12	C17	H17	119.6
C16	C17	H17	119.6
C13'	C12'	C17'	120(2)
C13'	C12'	C1'	115(3)
C17'	C12'	C1'	124(2)
C12'	C13'	C14'	121(2)
C12'	C13'	H13'	119.3
C14'	C13'	H13'	119.3

C13'	C14'	C15'	117(3)
C13'	C14'	H14'	121.4
C15'	C14'	H14'	121.4
C16'	C15'	C14'	116(3)
C16'	C15'	H15'	122.0
C14'	C15'	H15'	122.0
C17'	C16'	C15'	120(3)
C17'	C16'	H16'	120.1
C15'	C16'	H16'	120.1
C16'	C17'	C12'	119(2)
C16'	C17'	H17'	120.6
C12'	C17'	H17'	120.6
C19	C18	C6	112.88(18)
C20'	C18	C6	116.18(19)
C19	C18	C20	116.4(2)
C6	C18	C20	102.61(16)
C19	C18	C21	115.4(2)
C6	C18	C21	108.15(17)
C20	C18	C21	99.80(19)
C20'	C18	C21'	110.7(2)
C6	C18	C21'	107.96(14)
C20'	C18	C19'	108.94(19)
C6	C18	C19'	114.25(15)
C21'	C18	C19'	97.13(19)
C18	C19	H19A	109.5
C18	C19	H19B	109.5
H19A	C19	H19B	109.5
C18	C19	H19T	109.5
H19A	C19	H19T	109.5
H19B	C19	H19T	109.5
C18	C20	H20A	109.5
C18	C20	H20B	109.5
H20A	C20	H20B	109.5
C18	C20	H20C	109.5
H20A	C20	H20C	109.5
H20B	C20	H20C	109.5
C18	C21	H21A	109.5

C18	C21	H21B	109.5
H21A	C21	H21B	109.5
C18	C21	H21C	109.5
H21A	C21	H21C	109.5
H21B	C21	H21C	109.5
C18	C19'	H19C	109.5
C18	C19'	H19D	109.5
H19C	C19'	H19D	109.5
C18	C19'	H19E	109.5
H19C	C19'	H19E	109.5
H19D	C19'	H19E	109.5
C18	C20'	H20D	109.5
C18	C20'	H20E	109.5
H20D	C20'	H20E	109.5
C18	C20'	H20F	109.5
H20D	C20'	H20F	109.5
H20E	C20'	H20F	109.5
C18	C21'	H21D	109.5
C18	C21'	H21E	109.5
H21D	C21'	H21E	109.5
C18	C21'	H21F	109.5
H21D	C21'	H21F	109.5
H21E	C21'	H21F	109.5

6.2 (2*S*,3*R*)-4,4,5,5-Tetramethyl-2-(2-phenyl-3-vinylchroman-6-yl)-1,3,2-dioxaborolane (Product 2d)

CCDC 2266135

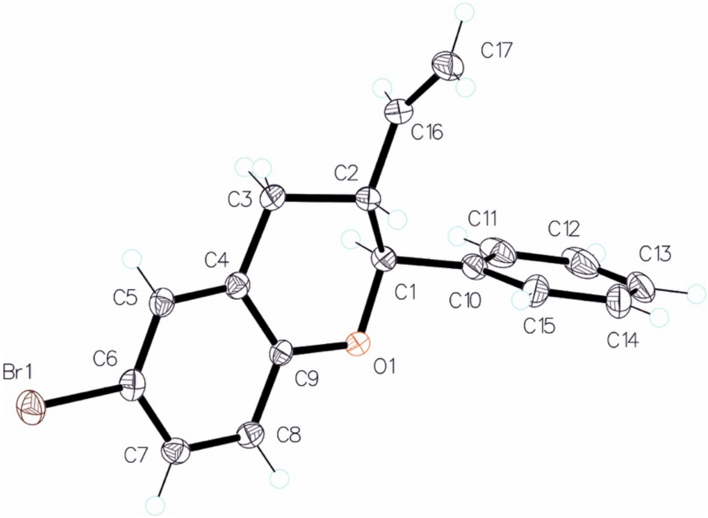

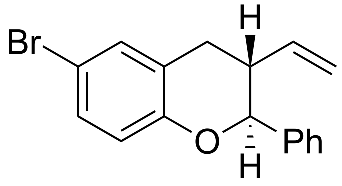

Table S9. Crystal data and structure refinement for TMG01909F_PB.

Identification code	TMG01909F_PB
Empirical formula	$C_{68}H_{60}Br_4O_4$
Formula weight	1260.80
Temperature/K	100
Crystal system	triclinic
Space group	P1
a/Å	9.7946(3)
b/Å	13.0686(3)
c/Å	13.1761(3)
α/°	108.073(2)
β/°	97.027(2)
γ/°	111.729(3)
Volume/Å^3	1434.69(7)

Z	1
ρ_{calc}g/cm^3	1.459
μ/mm^{-1}	2.854
F(000)	640.0
Crystal size/mm^3	0.08 × 0.06 × 0.02
Radiation	MoKα (λ = 0.71073)
2Θ range for data collection/°	3.636 to 64.746
Index ranges	-14 ≤ h ≤ 14, -19 ≤ k ≤ 19, -19 ≤ l ≤ 19
Reflections collected	66225
Independent reflections	18761 [R_{int} = 0.0465, R_{sigma} = 0.0414]
Data/restraints/parameters	18761/3/685
Goodness-of-fit on F^2	1.070
Final R indexes [I>=2σ (I)]	R_1 = 0.0406, wR_2 = 0.0992
Final R indexes [all data]	R_1 = 0.0480, wR_2 = 0.1019
Largest diff. peak/hole / e Å^{-3}	0.95/-0.53
Flack parameter	-0.003(3)

Table S10. Fractional Atomic Coordinates (×10^4) and Equivalent Isotropic Displacement Parameters (Å^2×10^3) for TMG01909F_PB. U_{eq} is defined as 1/3 of the trace of the orthogonalised U_{IJ} tensor.

Atom	*x*	*y*	*z*	U(eq)
Br1C	1054.3(5)	4472.1(4)	8952.5(4)	29.34(11)
Br1	8874.1(5)	5407.3(4)	1087.1(4)	26.81(10)
Br1D	4221.1(6)	8361.8(4)	8784.0(4)	31.75(11)
Br1B	5582.8(5)	1198.2(4)	811.0(3)	24.34(10)

Atom	*x*	*y*	*z*	U(eq)
C6	8958(5)	6386(4)	2527(4)	20.3(8)
O1C	-85(4)	1222(3)	4235(2)	19.2(6)
C4	18766(5)	6608(4)	4387(3)	17.7(7)
C10C	-411(5)	643(3)	2316(3)	17.2(7)
C10D	5190(5)	2286(4)	5507(3)	18.2(7)
C14D	3478(5)	419(4)	4016(4)	22.7(8)
O1B	4723(3)	5429(3)	3580(2)	17.8(5)
C3D	5139(7)	4437(5)	8274(4)	29.7(10)
O1D	4660(4)	3966(3)	5923(2)	25.2(7)
C1B	5090(4)	6455(3)	3265(3)	15.2(7)
C14C	1086(6)	-170(5)	1294(4)	27.3(9)
C10B	4671(5)	7305(4)	4091(3)	15.3(7)
C17	9613(6)	6475(4)	8031(4)	28.6(10)
C8C	227(5)	1567(4)	6124(4)	22.7(8)
C12D	6164(5)	976(4)	4465(4)	22.8(8)
C14	11545(7)	10776(4)	9042(4)	33.2(11)
C15	10972(6)	9777(4)	8053(4)	25.4(9)
C10	9407(5)	9166(4)	7553(3)	19.9(8)
C13B	3915(6)	8854(4)	5656(4)	25.7(9)
C14B	2783(6)	7801(4)	4856(4)	26.4(9)
C9	9165(4)	7817(3)	4634(3)	15.8(7)
C4B	4966(5)	4314(4)	1814(3)	16.1(7)

Atom	*x*	*y*	*z*	U(eq)
C3C	257(5)	3625(4)	4610(4)	21.0(8)
C15D	3718(5)	1472(4)	4855(4)	20.4(8)
C5B	5170(5)	3328(4)	1189(3)	17.7(7)
C5D	4653(5)	6237(4)	8399(3)	22.7(8)
C5C	673(5)	3918(4)	6639(3)	19.9(8)
C8D	4260(5)	5669(4)	6136(4)	21.0(8)
C7	9337(5)	7582(4)	2776(4)	20.1(8)
C5	8674(5)	5890(4)	3319(3)	18.9(7)
C16	8735(5)	6630(4)	7299(4)	23.0(8)
C12C	-1629(7)	-1131(4)	633(4)	32.9(11)
C9C	173(5)	2010(4)	5291(3)	18.1(7)
C17C	799(6)	3525(4)	1967(4)	27.6(9)
O1	9362(3)	8609(2)	5665(2)	17.9(5)
C3	8495(5)	6112(4)	5276(4)	22.5(8)
C1	8798(5)	8114(4)	6461(3)	17.6(7)
C7C	488(6)	2295(4)	7214(4)	23.9(9)
C17B	3567(7)	7390(5)	1311(4)	31.9(10)
C8	9430(5)	8301(4)	3828(4)	18.8(8)
C9B	4907(4)	4484(4)	2903(3)	15.8(7)
C2C	449(5)	2846(4)	3544(3)	18.9(8)
C6C	700(5)	3471(4)	7450(3)	21.9(8)
C11B	5798(5)	8363(4)	4884(3)	16.9(7)

Atom	*x*	*y*	*z*	U(eq)
C2B	4280(5)	6050(3)	2045(3)	16.7(7)
C3B	4810(5)	5185(4)	1325(3)	19.4(8)
C12B	5444(5)	9142(4)	5680(4)	21.9(8)
C8B	5001(5)	3672(4)	3358(3)	18.0(7)
C13	10565(8)	11159(5)	9535(4)	37.3(13)
C4D	4778(5)	5219(4)	7756(3)	21.5(8)
C11C	-1732(5)	-283(4)	1530(4)	24.1(9)
C9D	4573(5)	4941(4)	6628(3)	20.2(8)
C4C	385(4)	3185(3)	5533(3)	17.0(7)
C7B	5189(5)	2688(3)	2735(3)	17.6(7)
C13C	-226(7)	-1080(5)	521(4)	32.9(11)
C16D	5735(8)	2684(5)	7922(4)	39.7(13)
C6B	5292(5)	2534(3)	1661(3)	18.1(7)
C11	8444(7)	9562(5)	8061(4)	28.4(10)
C16B	4612(6)	7113(4)	1719(3)	23.4(8)
C2	9250(5)	7105(4)	6442(3)	18.8(7)
C12	9016(8)	10554(5)	9048(4)	37.4(13)
C16C	-79(5)	3127(4)	2582(4)	22.0(8)
C7D	4147(5)	6679(4)	6782(4)	23.3(8)
C1D	5452(5)	3426(4)	6421(3)	20.2(8)
C2D	4895(6)	3237(4)	7414(4)	24.6(9)
C15B	3148(5)	7022(4)	4080(4)	22.0(8)

Atom	*x*	*y*	*z*	U(eq)
C6D	4352(5)	6956(4)	7908(4)	23.2(8)
C11D	6419(5)	2032(4)	5307(4)	21.4(8)
C1C	-517(4)	1533(3)	3314(3)	16.2(7)
C15C	1001(5)	693(4)	2188(3)	21.6(8)
C13D	4704(6)	173(4)	3815(4)	22.9(9)
C17D	5151(11)	1703(7)	8069(6)	58(2)

This compound crystallized in the triclinic chiral space group *P*1 with four identical molecules (same stereochemistry) in the asymmetric unit. The absolute structure could be determined reliable with a Flack value of 0.007(7) and Flack value based on Parsons' quotients of -0.003(3)[11]; Flack X determined using 7121 quotients [(I+)-(I-)]/[(I+)+(I-)]). The Flack (Parsons) parameter value for the correct absolute structure determination should be 0; the inverted structure would give 1; always taking in account the standard deviation. The absolute configuration based on the absolute structure of the measured crystals was determined with *S*(C1) and *R*(C2) respectively for molecules A to D. The structure presents a R1 value of 4.06 %.

7. SFC

Compound 2a

SFC (IG (100 × 3 mm, 3 µm), CO_2:MeOH 80:20, 1.2 mL/min, 25 ºC, BPR 150 bar, 210 nm): en1 (minor, 16%) min 0.90, en2 (major, 84%) min 0.98.

Racemic

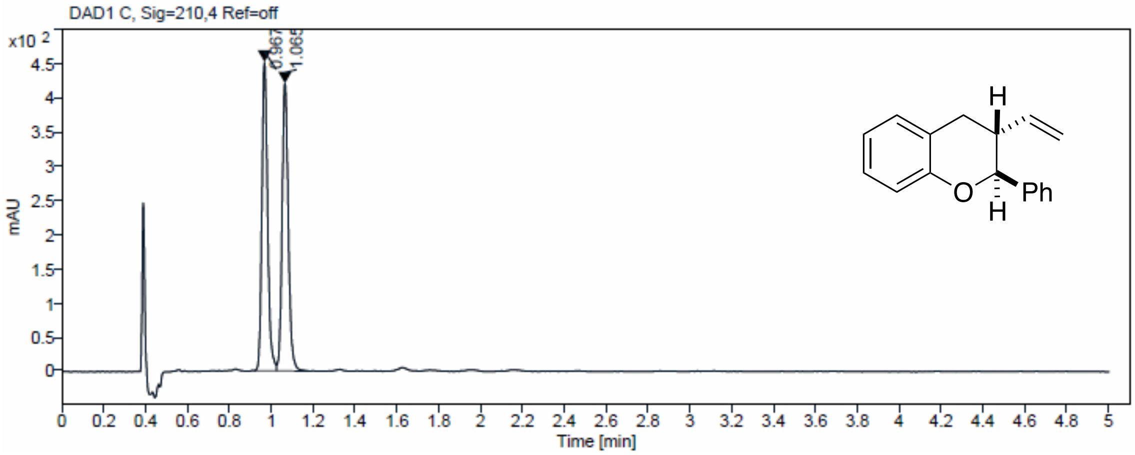

Signal: DAD1 C, Sig=210,4 Ref=off

RT [min]	Type	Width [min]	Area	Height	Area%	Name
0.967	BV	0.0291	856.7393	452.6874	49.7014	
1.065	VB	0.0315	867.0322	421.5662	50.2986	
		Sum	1723.7715			

Enantioenriched

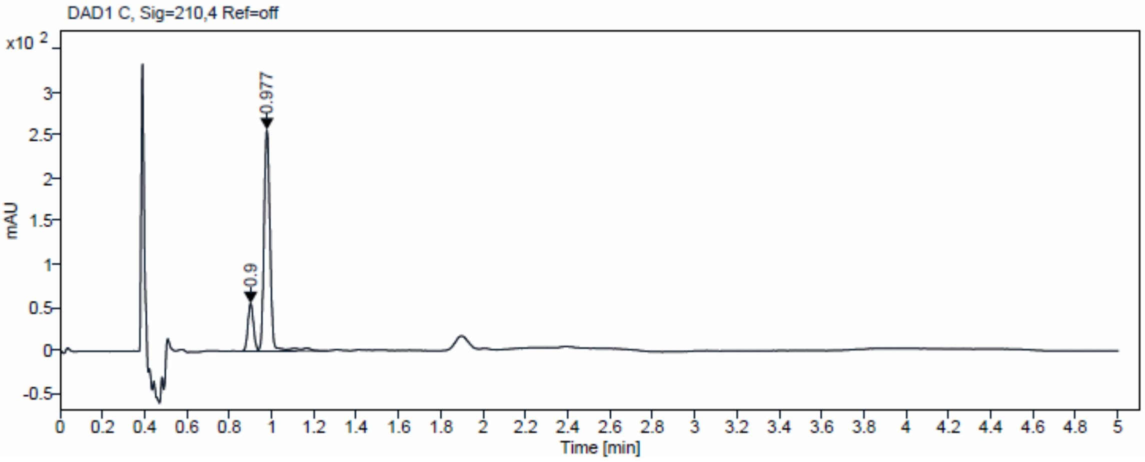

Signal: DAD1 C, Sig=210,4 Ref=off

RT [min]	Type	Width [min]	Area	Height	Area%	Name
0.900	BV	0.0289	101.5963	55.2693	16.4061	
0.977	VV R	0.0308	517.6627	257.3665	83.5939	
		Sum	619.2590			

Compound 2d

SFC (OJ (100 × 3 mm, 3 μm), CO_2:MeOH 70:30, 1.2 mL/min, 25 ºC, BPR 150 bar, 210 nm): en1 (major, 87%) min 0.90, en2 (minor, 13%) min 1.02.

Racemic

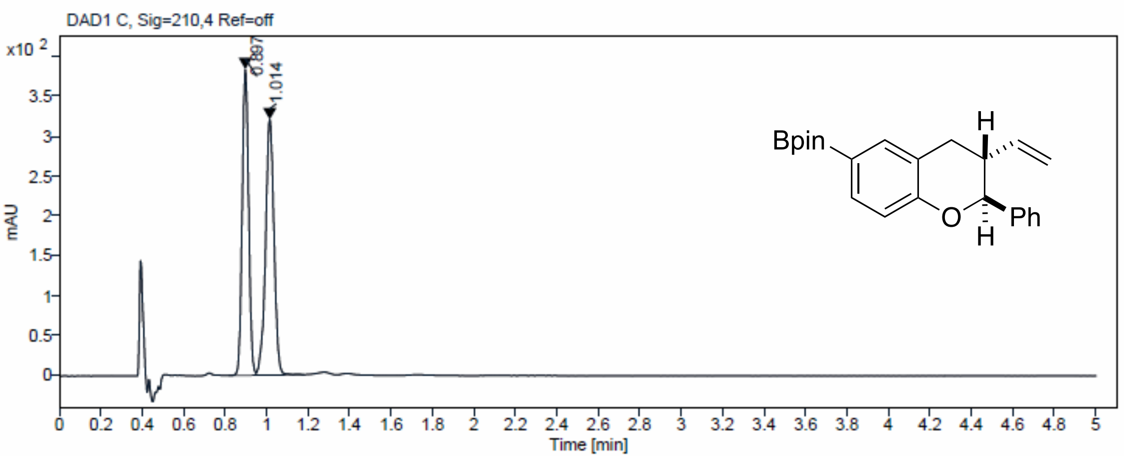

Signal: DAD1 C, Sig=210,4 Ref=off

RT [min]	Type	Width [min]	Area	Height	Area%	Name
0.897	BV	0.0337	830.7938	384.2666	47.6943	
1.014	VV R	0.0441	911.1212	320.8382	52.3057	
		Sum	1741.9150			

Enantioenriched

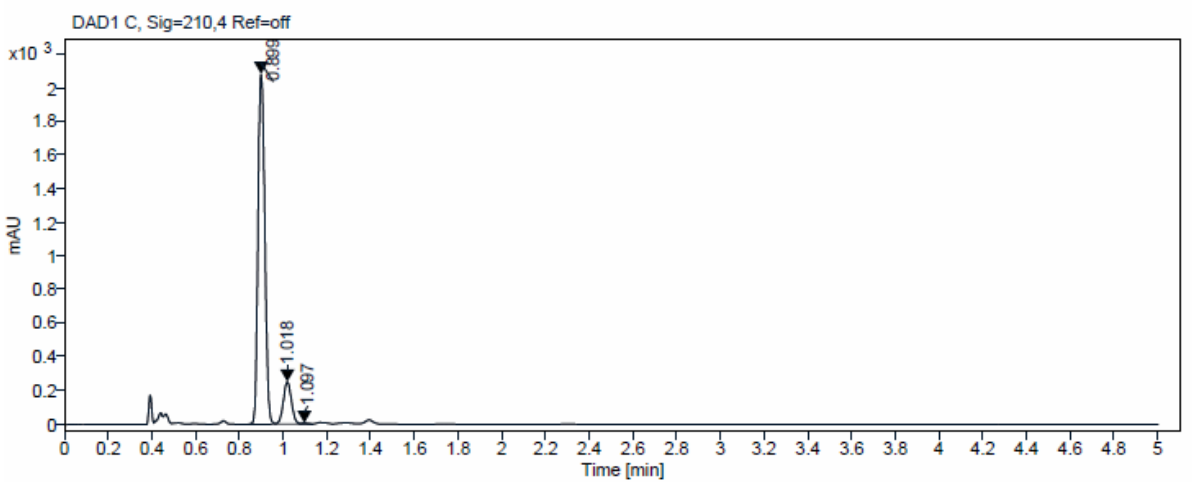

Signal: DAD1 C, Sig=210,4 Ref=off

RT [min]	Type	Width [min]	Area	Height	Area%	Name
0.899	BV	0.0346	4485.7573	2084.7520	87.2390	
1.018	VV R	0.0401	647.2815	250.9901	12.5883	
1.097	VB E	0.0309	8.8808	4.5204	0.1727	
		Sum	5141.9196			

Compound 2e

SFC (IB-N (100 × 3 mm, 3 μm), CO_2:MeOH 95:5, 1.2 mL/min, 25 ºC, BPR 150 bar, 210 nm): en1 (major, 87%) min 0.90, en2 (minor, 13%) min 1.02.

Racemic

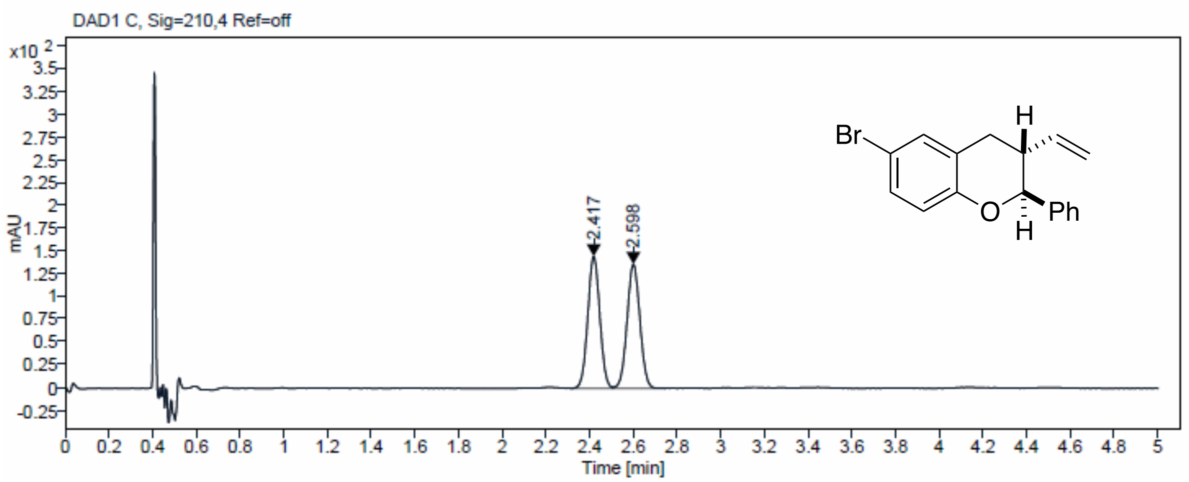

Signal: DAD1 C, Sig=210,4 Ref=off

RT [min]	Type	Width [min]	Area	Height	Area%	Name
2.417	BV	0.0597	554.4973	144.7156	49.9231	
2.598	VV R	0.0637	556.2057	136.1148	50.0769	
		Sum	1110.7031			

Enantioenriched

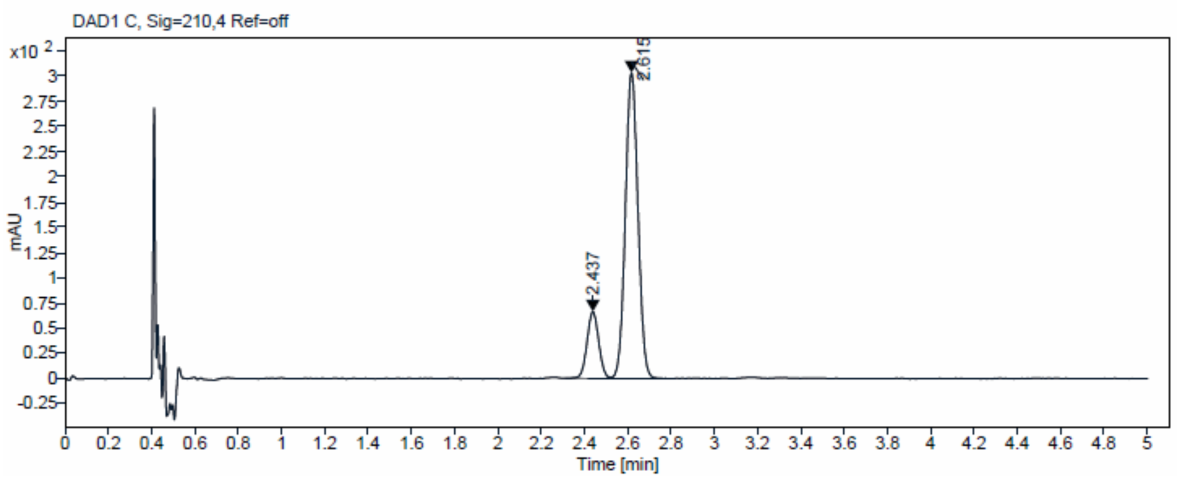

Signal: DAD1 C, Sig=210,4 Ref=off

RT [min]	Type	Width [min]	Area	Height	Area%	Name
2.437	VV R	0.0585	249.7388	66.2278	16.8586	
2.615	VV R	0.0627	1231.6366	304.2320	83.1414	
		Sum	1481.3754			

Compound 2m

SFC (OD (100 × 3 mm, 3 µm), CO_2:i-PrOH 95:5, 1.2 mL/min, 35 ºC, BPR 150 bar, 210 nm): en1 (minor, 77%) min 0.96, en2 (major, 23%) min 1.12.

Racemic

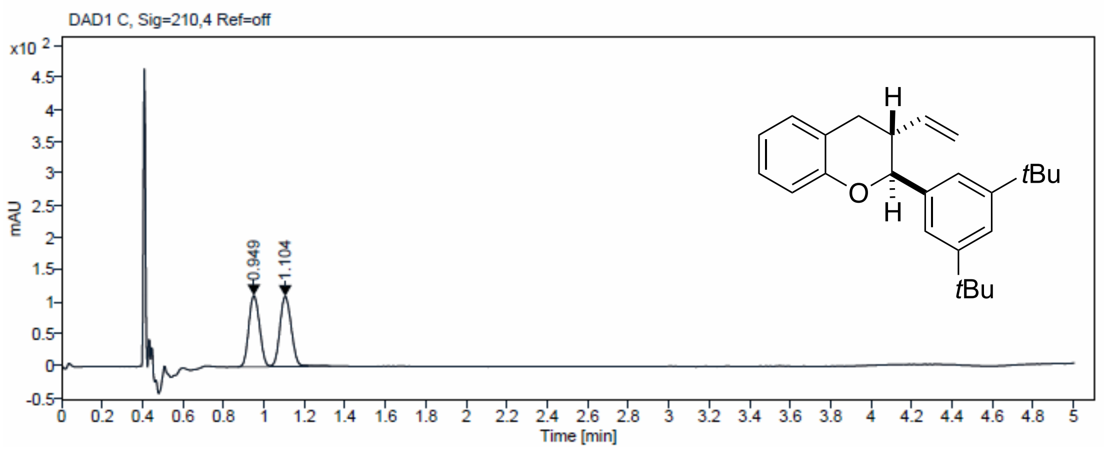

Signal: DAD1 C, Sig=210,4 Ref=off

RT [min]	Type	Width [min]	Area	Height	Area%	Name
0.949	BV	0.0591	409.9019	110.9811	48.8746	
1.104	VV R	0.0628	428.7789	109.2305	51.1254	
		Sum	838.6808			

Enantioenriched

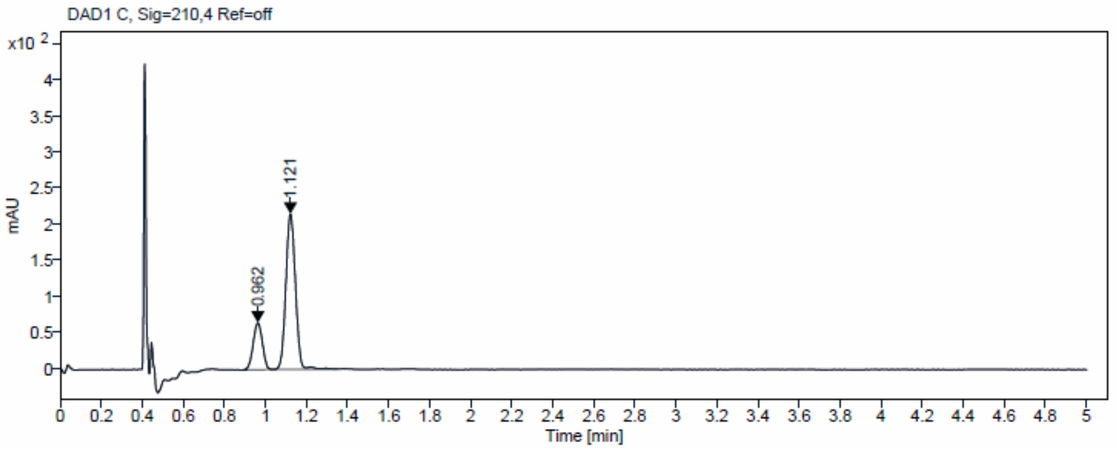

Signal: DAD1 C, Sig=210,4 Ref=off

RT [min]	Type	Width [min]	Area	Height	Area%	Name
0.962	BB	0.0505	202.2861	64.6701	22.5299	
1.121	BV R	0.0499	695.5703	214.7293	77.4701	
		Sum	897.8564			

8. References

1. Karki, R.; Thapa, P.; Kang, M. J.; Jeong, T. C.; Nam, J. M.; Kim, H.-L.; Na, Y.; Cho, W.-J.; Kwon, Y.; Lee, E.-S. Synthesis, Topoisomerase I and II Inhibitory Activity, Cytotoxicity, and Structure–Activity Relationship Study of Hydroxylated 2,4-Diphenyl-6-aryl Pyridines *Bioorg. Med. Chem.* **2010**, *18*, 3066–3077.

2. Yuan, H.; Chen, H.; Jin, H.; Li, B.; Yue, R.; Ye, J.; Shen, Y.; Shan, L.; Sun, Q.; Zhang, W. Deoxygenation of α,β-Unsaturated Acylphenols through Ethyl *o*-Acylphenylcarbonates with Luche Reduction *Tetrahedron Lett.* **2013**, *54*, 2776–2780.

3. Mino, T.; Kogure, T.; Abe, T.; Koizumi, T.; Fujita, T.; Sakamoto, M. Palladium-Catalyzed Allylic Arylation of Allylic Ethers with Arylboronic Acids Using Hydrazone Ligands *Eur. J. Org. Chem.* **2013**, 2013, 1501–1505.

4. Jurd, L.; Stevens, K.; Manners, G. Acid-Catalyzed and Thermal Rearrangements of Obtusaquinol and Related 3,3-Diarylpropenes *Tetrahedron* **1973**, *29*, 2347–2353.

5. Zhang, W.; Haight, A. R.; Hsu, M. C. Palladium-Catalyzed Coupling of Alkynes with Alcohols and Carboxylic Acids *Tetrahedron Lett.* **2002**, *43*, 6575–6578.

6. Magolan, J.; Jentsch, N.; Zhang, X.; Piotrowski, M.; Darveau, P.; Fragis, M.; Johnson, J.; Ritchie, N.; Kaul, A. Processes for the Preparation of *Ortho*-Allylated Hydroxy Aryl Compounds, Patent WO2021237371A1, **2021**.

7. Zhang, H.; Lin, S.; Jacobsen, E. N. Enantioselective Selenocyclization via Dynamic Kinetic Resolution of Seleniranium Ions by Hydrogen-Bond Donor Catalysts *J. Am. Chem. Soc.* **2014**, *136*, 16485–16488.

8. Denmark, S. E.; Kornfilt, D. J. P. Catalytic, Enantioselective, Intramolecular Sulfenofunctionalization of Alkenes with Phenols *J. Org. Chem.* **2017**, *82*, 3192–3222.

9. Vyvyan, J. R.; Dimmitt, H. E.; Griffith, J. K.; Steffens, L. D.; Swanson, R. A. Gold-catalyzed Rearrangement of Substituted Allyl Aryl Ethers *Tetrahedron Lett.* **2010**, *51*, 6666–6669.

10. Trivedi, R.; Tunge, J. A. Regioselective Iron-Catalyzed Decarboxylative Allylic Etherification *Org. Lett.* **2009**, *11*, 5650–5652.

11. (a) Flack, H. D. On Enantiomorph-Polarity Estimation *Acta Cryst.* **1983**, 876. (b) Parsons, S.; Flack, H. Precise Absolute-Structure Determination in Light-Atom Crystals *Acta Cryst.* **2004**, 61. (c) Flack, H. D.; Wagner, H. Use of Intensity Quotients and Differences in Absolute Structure Refinement *Acta Cryst.* **2013**, 249–259. (d) Escudero-Adán, E. C.; Benet-Buchholz, J.; Ballester, P. The Use of Mo Kα Radiation in the Assignment of the Absolute Configuration of Light-Atom Molecules; the Importance of High-Resolution Data *Acta Cryst.* **2014**, 660–668.
